# Characteristics of retracted research papers before and during the COVID-19 pandemic

**DOI:** 10.3389/fmed.2023.1288014

**Published:** 2024-01-10

**Authors:** Yuki Furuse

**Affiliations:** Nagasaki University Graduate School of Biomedical Sciences, Nagasaki, Japan

**Keywords:** COVID-19, SARS-CoV-2, publication, retraction, research integrity

## Abstract

**Objectives:**

During the COVID-19 pandemic, a large number of research papers were published, and some of them were retracted. The present study aims to reveal the characteristics of retracted papers before and during the pandemic.

**Methods:**

The study investigated 24,542,394 publications from 1999 to 2022 and analyzed the profiles of retracted papers from the perspectives of year, disease category, country, and journal.

**Results:**

Retraction rates were generally increasing at least until 2019, and were the highest for “Neoplasms.” The number of publications for “Infections” and “Respiratory Tract Diseases” dramatically rose during the COVID-19 pandemic; however, the retraction rates in the two categories or of COVID-19-related papers were not especially high compared to other diseases. The association with retraction was strongest for China in most disease categories, whereas for COVID-19 papers, other countries showed higher retraction rates than China. In recent years, retracted papers have become less likely to be published in high-impact journals.

**Conclusion:**

The COVID-19 pandemic does not seem to affect the retractions of research papers much. We should keep monitoring retractions and analyze the effects of pandemics for better science.

## 1 Introduction

Medical research plays an important role in advancing knowledge and in improving health. The hypotheses, findings, and reviews of biomedical research are widely shared among research communities and the general public through publications. Yet, “to err is human” (1). Some publications were retracted due to honest errors or misconduct.

During the COVID-19 pandemic, research communities have been eager to study this disease in order to mitigate its impact on public health. Thanks to these efforts, we successfully identified the causative agent, SARS-CoV-2, characterized its clinical and epidemiological features, and developed remedies and vaccines. However, unfortunately, some studies were later retracted, causing chaos. Such eventually retracted studies might result in mistakes in patient care that could cause possible harm. For example, several studies have reported the effectiveness of ivermectin in treating COVID-19, but were eventually retracted (2). These retractions affected the results of a meta-analysis that initially supported the use of the medicine. After excluding data from retracted and questionable studies, the effect did not reach statistical significance (3). In actuality, the administration of the drug aiming to prevent or treat COVID-19 has caused severe adverse events in some people (4, 5).

Researchers have rushed to conduct studies on COVID-19 and publish their results. There might have been pressure to publish results quickly to combat the pandemic, possibly leading to careless or poor research that was later revealed to be incorrect. Furthermore, a flood of submissions and acceleration of the review process during the pandemic might affect the quality of research papers regarding not only COVID-19 but also other diseases (6). As such, the pandemic may have inflated the number of retractions and also made an impact on various aspects of retractions (7). This study analyzed the profiles of retracted research papers and compared them before and during the COVID-19 pandemic. Understanding them would help conduct better research activities, minimizing the chance of future retractions even under crisis situations such as a pandemic.

## 2 Methods

### 2.1 Publication records

The number of publications by retraction status, publication year, disease category, authors’ affiliated country, and journal was obtained via PubMed, accessed between 1 June 2023 and 15 October 2023.^1^ Data were retrieved using the Rentrez package in R (8).

The disease category and microbial etiology of research papers were searched using MeSH terms (9). Information on the Journal Impact Factor 2021 (published in 2022), Journal Impact Factor 2018 (published in 2019), and Scimago Journal Rank (SJR) indicator 2022 was used to identify high-impact and “good” journals. All query terms used in the present study can be found in Supplementary Table S1.

When a research paper could be assigned to multiple disease categories or authors’ affiliations, that paper was counted multiple times in different classifications. Thereby, affiliations were analyzed for all coauthors, not exclusively for the first or corresponding author.

### 2.2 Statistical analysis

The numbers of total publications, retracted papers, and retraction rates were counted and calculated by publication year and disease category. Disease categories were ranked based on the retraction rates by 3-year-periods from 1999 to 2022.

The association of affiliated countries with retracted papers was evaluated using odds ratio by the 3-year-periods. The odds ratio was calculated as follows: (“number of retracted papers from a country of interest”/“number of retracted papers from other countries”)/(“number of non-retracted publications from a country of interest”/“number of non-retracted publications from other countries”).

The increasing trend of retraction rates was tested using the Kendall rank correlation coefficient. A multivariable regression analysis, assuming a quasi-Poisson distribution, was performed to analyze the associations of publication year, disease category, and affiliated country with retraction counts, using the number of total publications as an offset. Statistical significance was set at *p* < 0.05.

## 3 Results

### 3.1 Disease category of retracted papers

**Figure 1:**
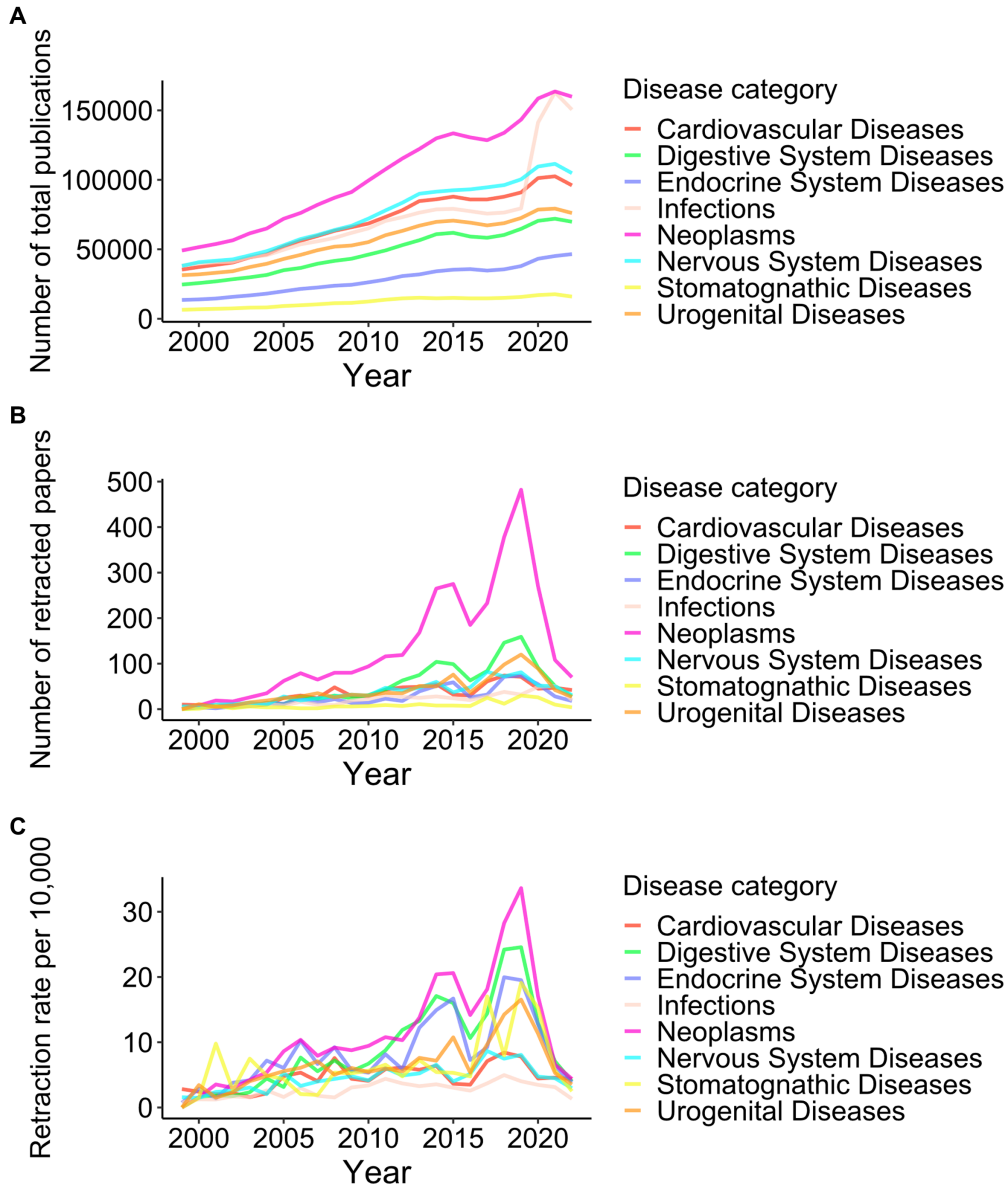
Trend of retractions among eight disease categories from 1999 to 2022. Yearly numbers of total publications **(A)**, retracted papers **(B)**, and retraction rates per 10,000 publications **(C)** in eight disease categories, which are among the top 5 diseases in publication counts or retraction rates, are shown. The results of all 18 disease categories can be found in Supplementary Figure S1.

Between 1999 and 2022, 24,542,394 publications were indexed in PubMed; of these, 14,717 were retracted (6.0 in 10,000 papers). Among the 18 disease categories, “Neoplasms” had the highest number of retracted papers (no. = 3,234), followed by “Digestive System Diseases” (1,187), “Urogenital Diseases” (959), “Nervous System Diseases” (866), and “Cardiovascular Diseases” (834) (Figure 1; Supplementary Figures S1–S3). In terms of retraction rates, which were calculated as the number of retracted papers per total publications, the highest rate was observed for “Neoplasms” (13.1 in 10,000 papers), followed by “Digestive System Diseases” (10.4), “Endocrine System Diseases” (9.3), “Urogenital Diseases” (7.1), and “Stomatognathic Diseases” (7.0).

Both the total number of publications and the number of retracted papers increased from 1999 to 2019. During this period, statistically significant increases in the retraction rate were found for 16/18 disease categories. Although the total number of publications continued to increase after 2019, the retraction counts and rates declined (Figures 1B,C; Supplementary Figures S1–S3). The yearly number of retractions and retraction rates were analyzed based on not their retraction date but the publication date. The decrease of retractions after 2019 must be because this study was conducted in 2023; it still takes time to realize concerns and investigate them for papers published in 2020 or later. Therefore, comparing retraction rates in recent years to past years is difficult.

**Figure 2:**
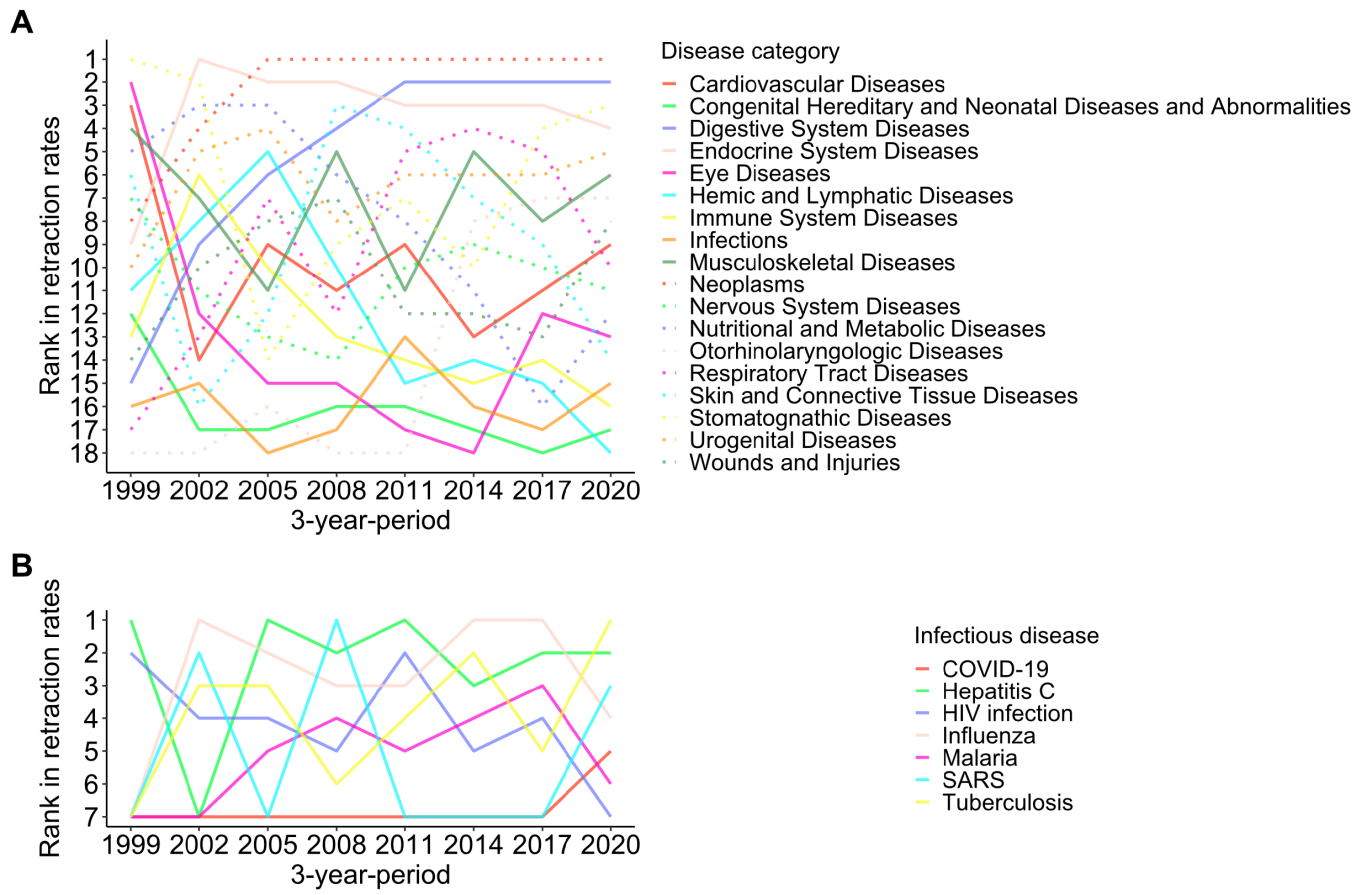
Ranking of retraction rates among different disease categories from 1999 to 2022. Ranking of retraction rates in 18 disease categories **(A)** and seven infectious diseases **(B)** in 3-year-periods are shown. Because there were no retractions for research papers on Ebola, Polio, and Zika, the three diseases were not shown in panel **(B)**.

To address this issue, we compared the retraction rates among different disease categories using contemporary data. Figure 2A illustrates the trend of the contemporary ranking of retraction rates by disease category over 24 years by 3-year-periods. “Neoplasms” ranked the top in 6 out of 8 periods. “Respiratory Tract Diseases” was situated in the top 4 or 5 in the rank in 2011–2019, but it dropped to 10th in 2020–2022. “Infections” kept low positions, 13th or below, throughout the study periods, even during the COVID-19 pandemic.

### 3.2 Infectious diseases in retracted papers

**Figure 3:**
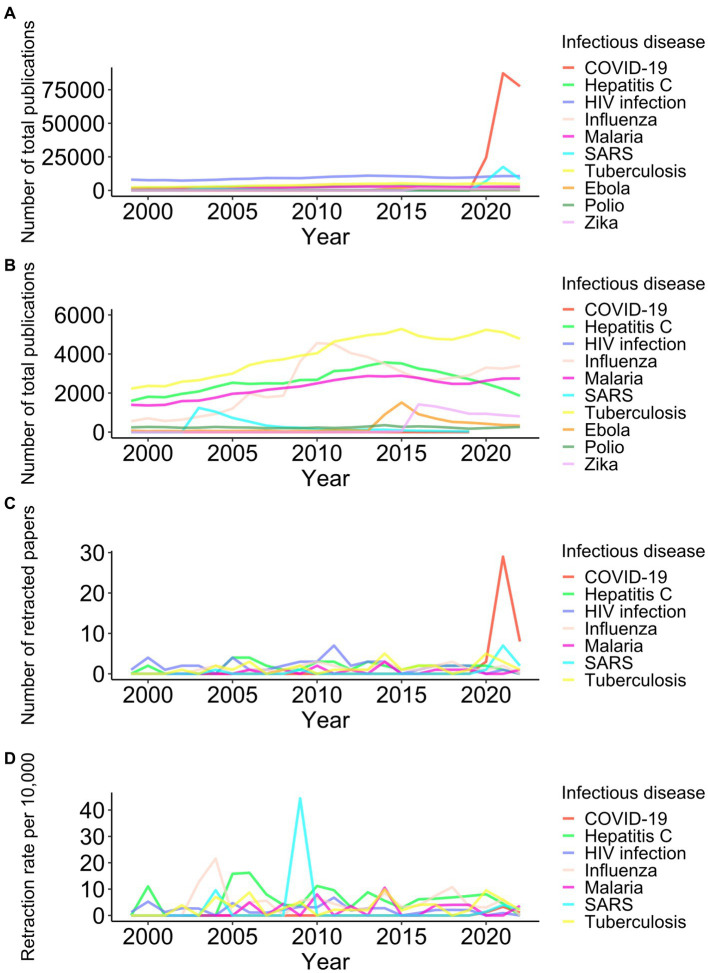
Trend of retractions among 10 infectious diseases from 1999 to 2022. Yearly numbers of total publications (**A** and **B** in different scales on the *y*-axis), retracted papers **(C)**, and retraction rates per 10,000 publications **(D)** for ten infectious diseases are shown. Because there were no retractions for research papers on Ebola, Polio, and Zika, the three diseases were excluded in panels **(C, D)**.

I then investigated retracted papers related to the top five intensively studied infectious diseases identified in a previous study (10): hepatitis C, HIV infection, influenza, malaria, and tuberculosis, along with five trendy infectious diseases over the past 20 years: COVID-19, Ebola virus disease, poliomyelitis, SARS, and Zika fever. Those diseases were declared a Public Health Emergency of International Concern by the World Health Organization (11). The number of publications on such infectious diseases rose after big outbreaks, that is, around 2003 for SARS, 2009 for influenza, 2014 for Ebola, 2016 for Zika, and 2020 for COVID-19 (Figures 3A,B; Supplementary Figure S4).

From 2020 to 2022, 40 papers regarding COVID-19 were retracted (Figure 3C). Ten retracted papers on SARS in the corresponding period must be associated with studies comparing COVID-19 with SARS. Disease outbreaks did not lead to an increase in the retractions of research papers about influenza, Ebola, or Zika (Figures 2B, 3C,D). The peaks of SARS-related retractions in 2004 and 2009 were formed by one retracted paper each year. During the 2020–2022 COVID-19 pandemic, the retraction rate of COVID-19 papers was lower than that of publications on tuberculosis, hepatitis C, or influenza (Figure 2B).

### 3.3 Retracted papers and authors’ affiliated country

**Figure 4:**
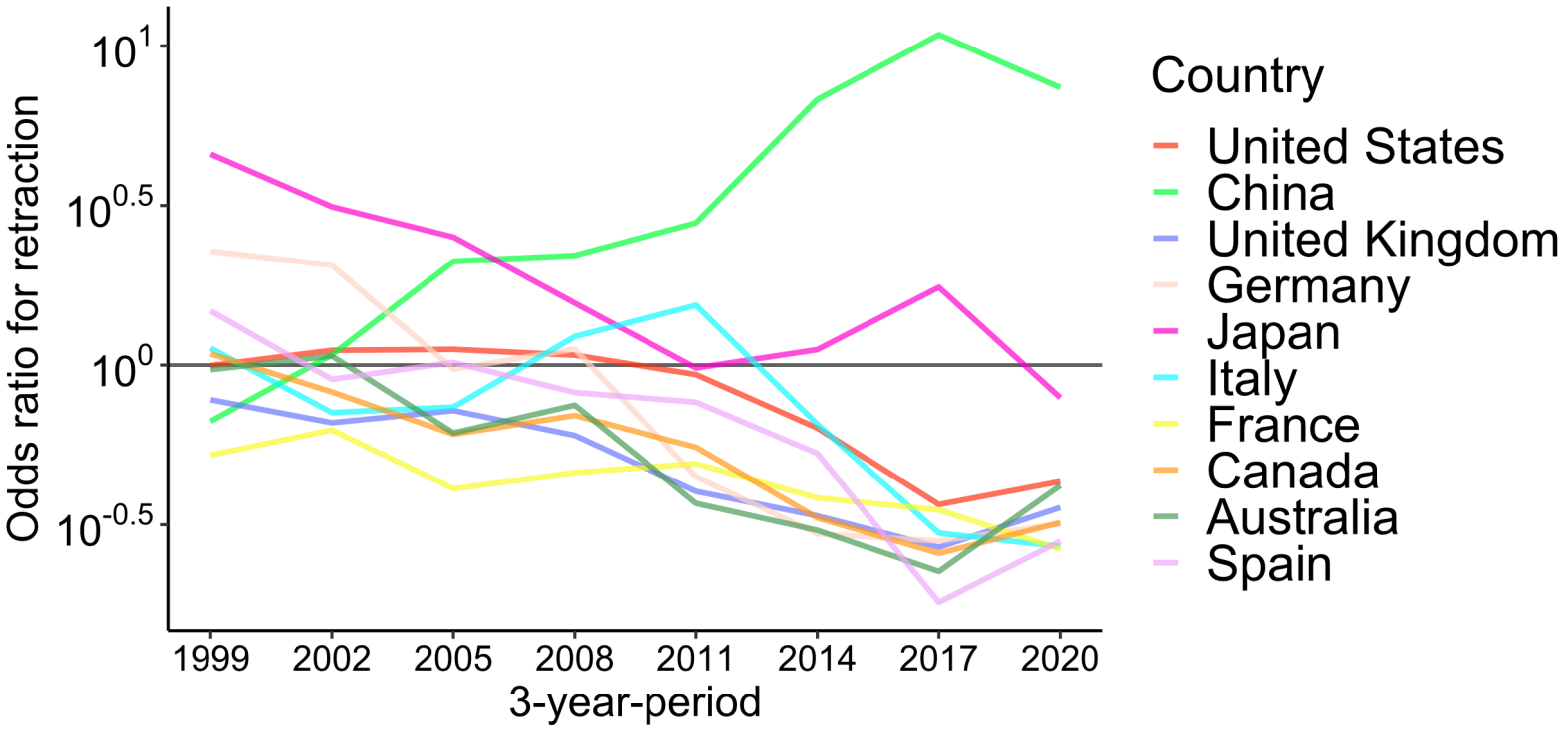
Association of affiliated countries with retraction from 1999 to 2022 in top 10 publishing countries. Odds ratios for retraction in 3-year-periods are shown for the top 10 countries with the highest publication counts. The results of the top 20 publishing countries can be found in Supplementary Figure S5.

Next, I analyzed data on countries affiliated with the authors of retracted papers in the top 20 countries with the largest number of publications. The association between retracted papers and affiliated countries was assessed by calculating the odds ratio in 3-year-periods (Figure 4; Supplementary Figure S5). During 2020–2022, a positive association for retractions was observed for China, India, and Iran. The odds ratios were high before, but recently decreased in Germany, Japan, and South Korea. The United Kingdom, France, the Netherlands, Brazil, Turkey, Switzerland, and Belgium have always shown a negative association with retractions from 1999 to 2022.

**Figure 5:**
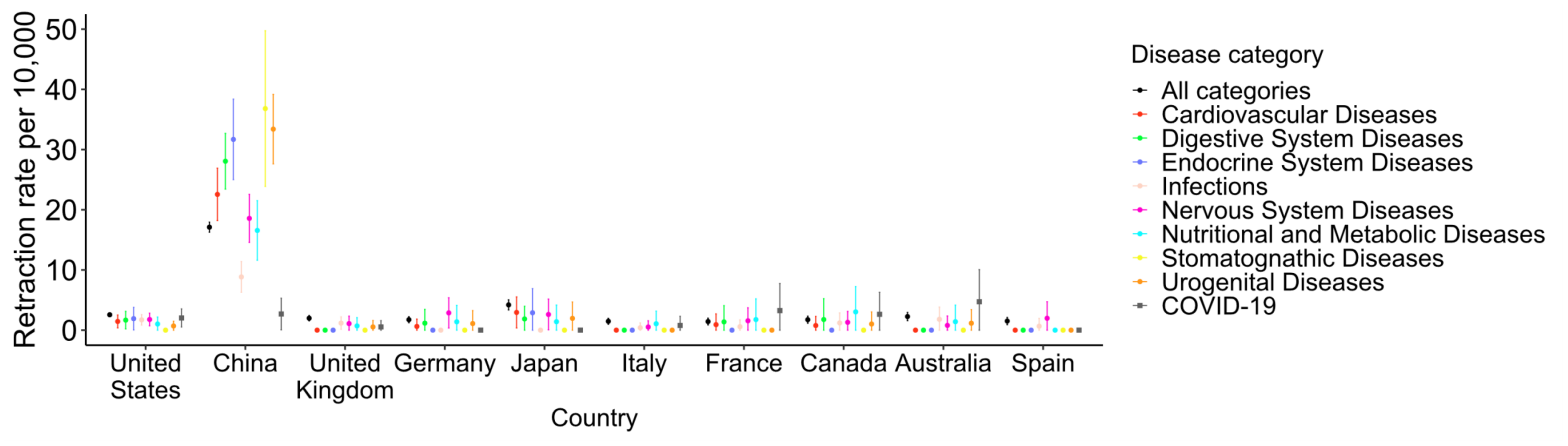
Retraction rates by country and disease category in 2020–2022, in top 10 publishing countries for eight disease categories plus COVID-19. The retraction rates per 10,000 papers in 2020–2022 are shown for eight disease categories, as determined in Figure 1, and COVID-19-related papers in the top 10 countries with the highest publication counts. Vertical lines indicate 95% confidence intervals. The results of the top 20 countries for all 18 disease categories can be found in Supplementary Figure S6.

Figure 5 and Supplementary Figures S6–S8 show the retraction rates in 2020–2022 by the 18 disease categories plus COVID-19 and affiliated countries. The retraction rates of COVID-19-related papers were comparable with other diseases in most countries. While retraction rates were generally high in China, India, Iran, Turkey, and Japan (>4.0 per 10,000 publications), the retraction rates of COVID-19 papers from those countries were lower than other diseases except in India. China had the highest retraction rates in 16 out of 18 disease categories and the second highest in the remaining two categories. However, when it comes to COVID-19, seven countries (India, South Korea, Australia, Belgium, Sweden, France, and Iran) showed higher retraction rates than China.

### 3.4 Multivariable analysis for retraction counts

Factors significantly associated with retraction count by a multivariable regression analysis are listed in Table 1. As indicated by descriptive observations so far, recent publications, particular disease categories (e.g., “Neoplasms,” “Endocrine System Diseases,” and “Digestive System Diseases”), and specific countries (e.g., China, Iran, India, and Japan) showed significant associations with retraction after statistical adjustments among those variables. The analysis found that publications in “Infections” are significantly less likely to be retracted compared with “Cardiovascular diseases,” which is situated in the middle in retraction rates among 18 disease categories (incidence rate ratio, 0.56; *p* value <0.001).

**Table 1:** Multivariable regression analysis for retraction count.

Variable	Category^*1^	Adjusted incidence rate ratio (95% confidence interval)^*2^	*p* value^*2^
Publication year	2017–2019	3.59 (2.82–4.67)	<0.001
	2014–2016	2.78 (2.17–3.63)	<0.001
	2011–2013	2.48 (1.92–3.24)	<0.001
	2008–2010	2.46 (1.90–3.24)	<0.001
	2005–2007	2.57 (1.98–3.40)	<0.001
	2002–2004	1.47 (1.09–2.00)	0.013
	1999–2001	Reference	
Disease category	Neoplasms	1.97 (1.73–2.25)	<0.001
	Endocrine system diseases	1.63 (1.37–1.95)	<0.001
	Digestive system diseases	1.51 (1.30–1.76)	<0.001
	Musculoskeletal diseases	1.34 (1.10–1.62)	0.003
	Urogenital diseases	1.28 (1.09–1.51)	0.003
	Skin and connective tissue diseases	1.23 (1.01–1.48)	0.035
	Cardiovascular diseases	Reference	
	Immune system diseases	0.79 (0.63–0.97)	0.031
	Eye diseases	0.71 (0.51–0.97)	0.037
	Infections	0.56 (0.46–0.68)	<0.001
	Congenital, hereditary, and neonatal diseases and abnormalities	0.47 (0.34–0.63)	<0.001
Affiliated country	China	17.57 (14.23–22.03)	<0.001
	Iran	7.98 (5.87–10.86)	<0.001
	India	5.15 (3.93–6.78)	<0.001
	Japan	4.95 (3.92–6.32)	<0.001
	United States	2.45 (1.96–3.10)	<0.001
	South Korea	1.77 (1.13–2.68)	0.009
	Italy	1.67 (1.25–2.24)	0.001
	Canada	1.47 (1.05–2.06)	0.024
	United Kingdom	Reference	

*^1^Only items with statistical significance are shown. ^*2^Adjusted incidence rate ratios and *p* values were calculated using a quasi-Poisson regression model.

### 3.5 Retracted papers in high-impact journals

**Figure 6:**
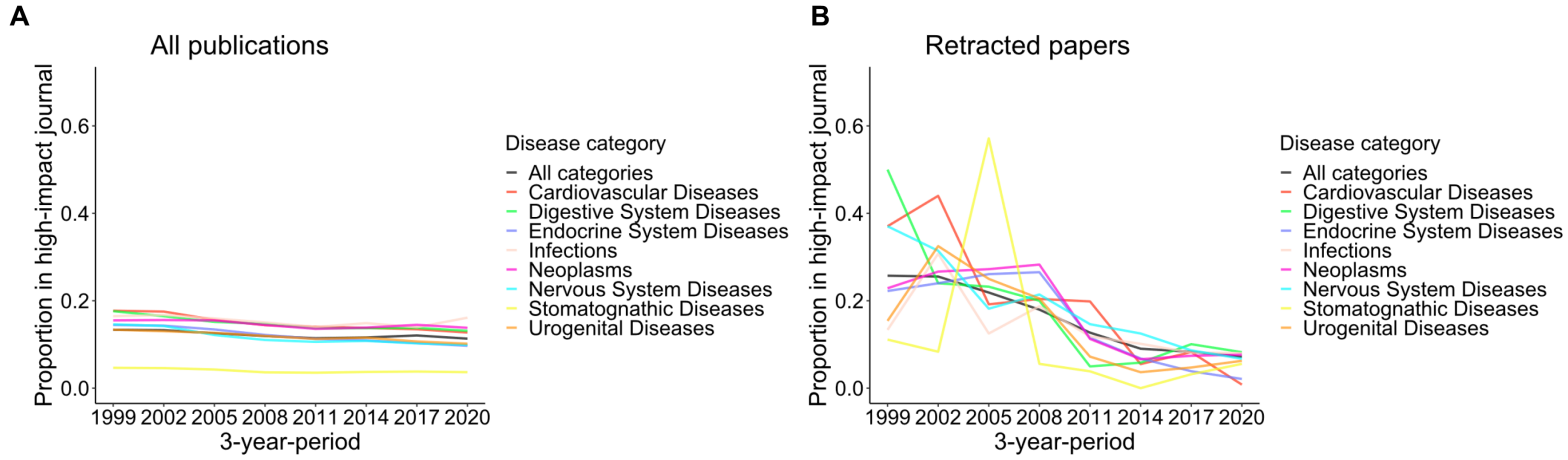
Proportion of papers published in high-impact journals among eight disease categories from 1999 to 2022. The proportions of papers published in high-impact journals (Impact Factor 2021 > 10) for total publications **(A)** and retracted papers **(B)** in eight disease categories from Figure 1 are shown in 3-year-periods. The results of all 18 disease categories can be found in Supplementary Figure S9.

Finally, the impact of the retracted papers was explored by checking the journals in which they were published. The proportion of all publications in high-impact journals (Journal Impact Factor 2021 > 10) has gradually decreased in most disease categories since 1999, except for the bounce of “Infections” and “Respiratory Tract Diseases” in 2020–2022 (Figure 6A; Supplementary Figure S9A).

**Figure 7:**
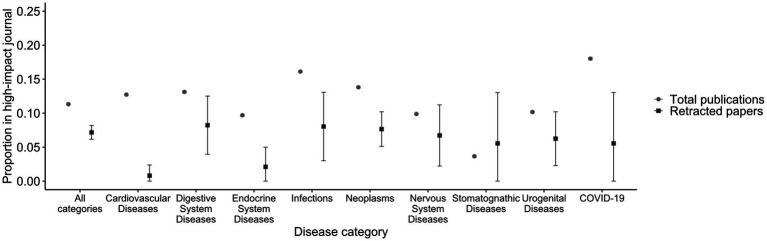
Proportion of papers published in high-impact journals in 2020–2022 among eight disease categories plus COVID-19. The proportions of papers published in high-impact journals (Impact Factor 2021 > 10) for total publications and retracted papers in 2020–2022 are depicted for eight disease categories and COVID-19. Vertical lines indicate 95% confidence intervals. The results of all 18 disease categories using different metrics can be found in Supplementary Figure S10.

The proportion of publications in high-impact journals was high for retracted papers before 2011 (Figure 6B; Supplementary Figure S9B). They have been decreasing recently, and in 2020–2022, retracted papers were less likely to be published in high-impact journals than the total publications in 17/18 disease categories (Figure 7; Supplementary Figure S10). The proportion of the total publications on COVID-19 published in high-impact journals was 18.0%, whereas 5.6% of the retracted COVID-19 papers were published in such journals. That proportion of retracted COVID-19 papers in high-impact journals was as low as other disease categories.

Because the COVID-19 pandemic drastically influenced the Journal Impact Factor (12), the same analysis was performed using the metric in 2018, i.e., the pre-COVID-19 period. Although the proportions of both total publications and retracted papers in high-impact journals slightly lessened when using the information of Journal Impact Factor 2018, their trend and relationship did not differ substantially (Supplementary Figures S9C,D, S10).

Similar sensitivity analyses using a different threshold (Impact Factor > 5) and indicator (SJR Q1 rank) also confirmed the decreasing trend of the proportion of retracted papers in so-called “good” journals (Supplementary Figures S9E–H). Still, there are differences in the results between analyses of high-impact journals and “good” journals. The proportion of retracted papers published in such “good” journals was higher than that of total publications in some disease categories (Supplementary Figure S10). For example, 75.0% of retracted papers about COVID-19 were published in Q1 journals, while the proportion in Q1 journals for total COVID-19 publications was 63.4%.

## 4 Discussion

This study found that (1) the retraction rates of medical research papers were increasing; (2) the retraction rates differed by year, disease category, country, and journal; and (3) the surge in research on COVID-19 led to a large number of retractions, but did not result in a high retraction rate. Although some previous studies investigated retracted publications, they did not focus on the disease categories of retracted papers (13–15). The skewed retraction rates in particular research areas and countries found in the present study suggest the existence of systemic problems in specific environments, such as the lack of research ethics education, insufficient research capability, or high pressure to publish research outcomes. However, this study does not mean to blame particular research areas or countries.

Retractions of COVID-19 studies were conspicuous during the pandemic period. Yet, this study did not find a high retraction rate for COVID-19 papers. The retraction rate of publications in “Infections” did not increase during the pandemic period either. The number of publications on COVID-19 was simply enormous. Notably, China showed a low retraction rate for COVID-19-related papers unlike other diseases. Further investigation on its mechanisms could provide clues for reducing retractions in the country.

The reasons for retraction vary. Honest errors can occur because of mistakes in handling samples or data, skewed statistical analyses, inaccuracies or unverifiable information, and irreproducibility. Misconduct includes plagiarism, data fabrication or manipulation, lack of adherence to ethical protocols, undisclosed conflicts of interest, and duplicate submissions. Although the present study did not analyze the reasons for retractions because such data were unavailable in the PubMed database, Shi et al. reported that COVID-19 papers were retracted more often without detailed explanations or due to “non-misconduct-related concerns” than non-COVID-19 studies (7).

One of the limitations of this study is that it relied solely on the data from PubMed. The database does not always include information on the time of submission or retraction. Changes in the time difference between submission and publication and between publication and retraction during the pandemic should be of interest and explored in future research. Furthermore, PubMed does not index some preprints that played a significant role in rapidly sharing research findings during the COVID-19 pandemic. More retractions can be found in other resources, for example, the Retraction Watch Database (6, 7).

The retraction counts and rates in recent years analyzed in this study must be underestimated. Because new publications with possible concerns are still under investigation or unrealized, some more papers may be retracted in the future. Consequently, it is difficult to directly compare the retraction rates in recent years to past years and determine if the COVID-19 pandemic has affected the retraction rates of non-COVID-19 research papers. Still, the relative changes in the retraction rates among different disease categories can be discussed (Figure 2). The retraction rates of “Neoplasms,” “Digestive System Diseases,” and “Endocrine System Diseases” remained high in both before and during the COVID-19 pandemic periods compared with other disease categories. There was no evident increase in the (relative) retraction rates of “Infections” or “Respiratory Tract Diseases” during the pandemic. Those findings imply marginal, if any, effects of the pandemic on retractions.

The importance of awareness, education, and compliance with research integrity increases as retraction rates continue to grow. We should attempt to reduce errors and misconduct in research activities. However, it is virtually impossible to completely eliminate errors or misconduct. Additionally, an increase in retraction rates in recent years may indicate improvement in research transparency, information sharing, and constructive criticism in research communities. The decreasing trend of retracted papers in high-impact journals also suggests a rigorous peer-review process.

We have to keep an eye on retractions and analyze how pandemics affect them to find and address issues for creating better science. Infectious disease pandemics could cause errors or misconduct in research due to pressure to rapidly publish research findings in a high-profile field. However, this study found that outbreaks of past emerging infectious diseases did not lead to an increase of retraction probability. In a crisis situation such as a pandemic, we should keep conducting research carefully and honestly to confront adversity.

## Data availability statement

Publicly available datasets were analyzed in this study. This data can be found here: https://pubmed.ncbi.nlm.nih.gov.

## Author contributions

YF: Conceptualization, Data curation, Formal analysis, Funding acquisition, Investigation, Methodology, Project administration, Visualization, Writing – original draft, Writing – review & editing.

## Footnotes

^1^ https://pubmed.ncbi.nlm.nih.gov/
