# Supplementary material for: Characteristics of retracted research papers before and during the COVID-19 pandemic
Source: Front Med (Lausanne). 2024 Jan 10;10:1288014. doi: 10.3389/fmed.2023.1288014 (PMC10806159; doi:10.3389/fmed.2023.1288014)
Supplement: Supplementary file 1 [file Data_Sheet_1.pdf]

Supplementary Materials

Supplementary Figures

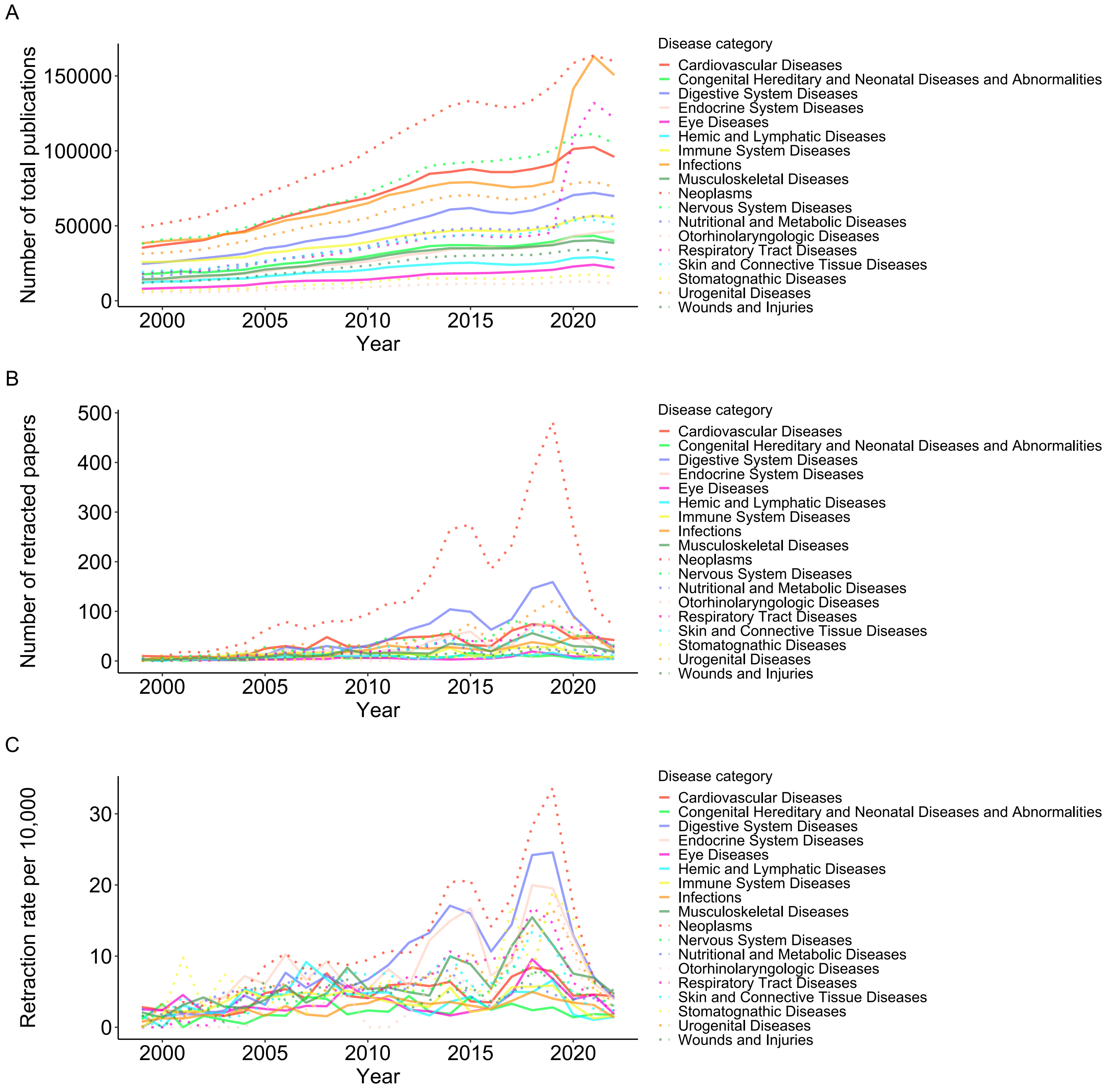

Supplementary Figure S1 Trend of retractions among 18 disease categories from 1999 to 2022.

Yearly numbers of total publications (A), retracted papers (B), and retraction rates per 10,000 publications (C) in 18 disease categories are shown.

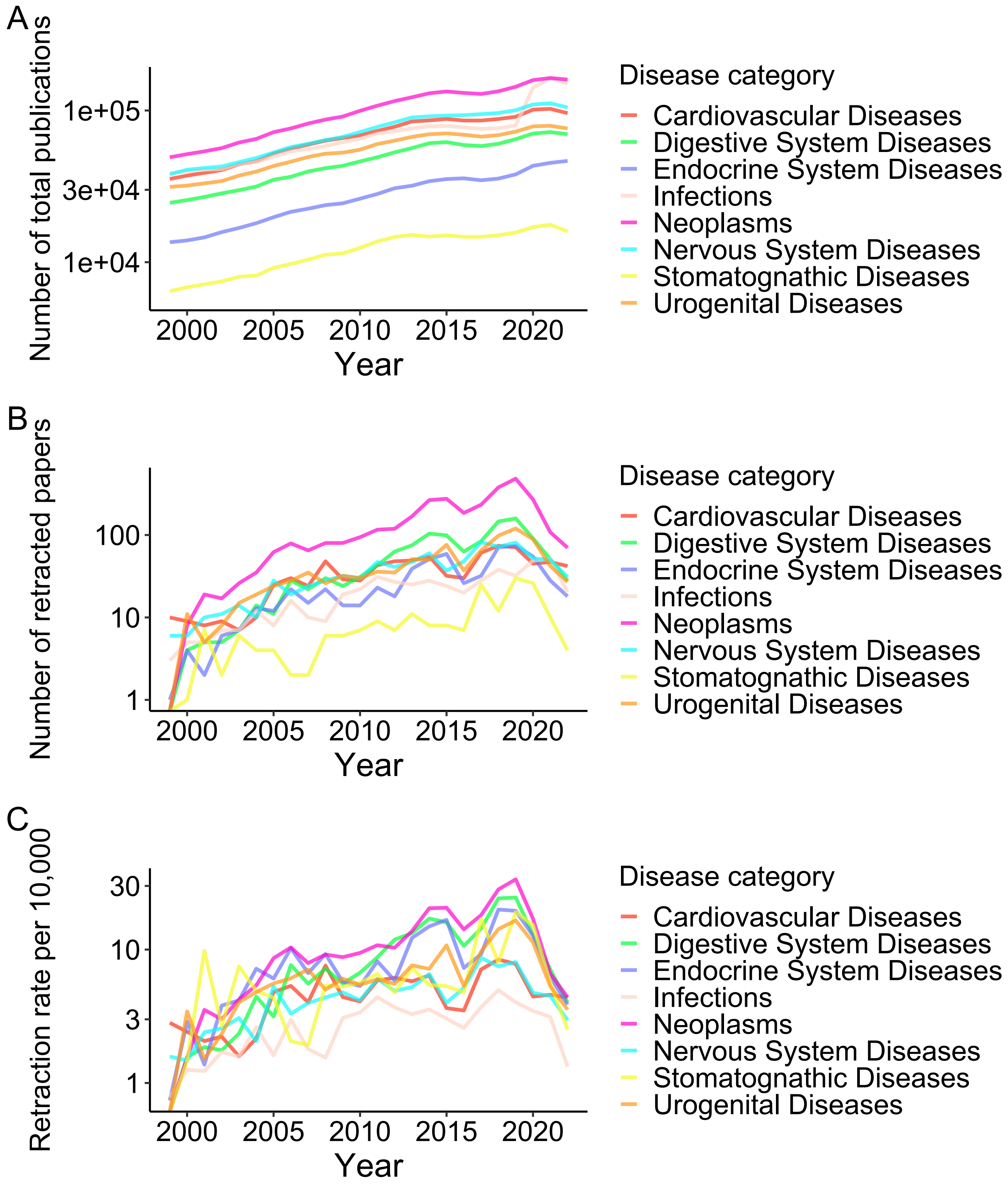

Supplementary Figure S2 Trend of retractions among eight disease categories from 1999 to 2022 (log-scale).

The same data in Figure 1 are shown in a logarithmic scale on the y-axis.

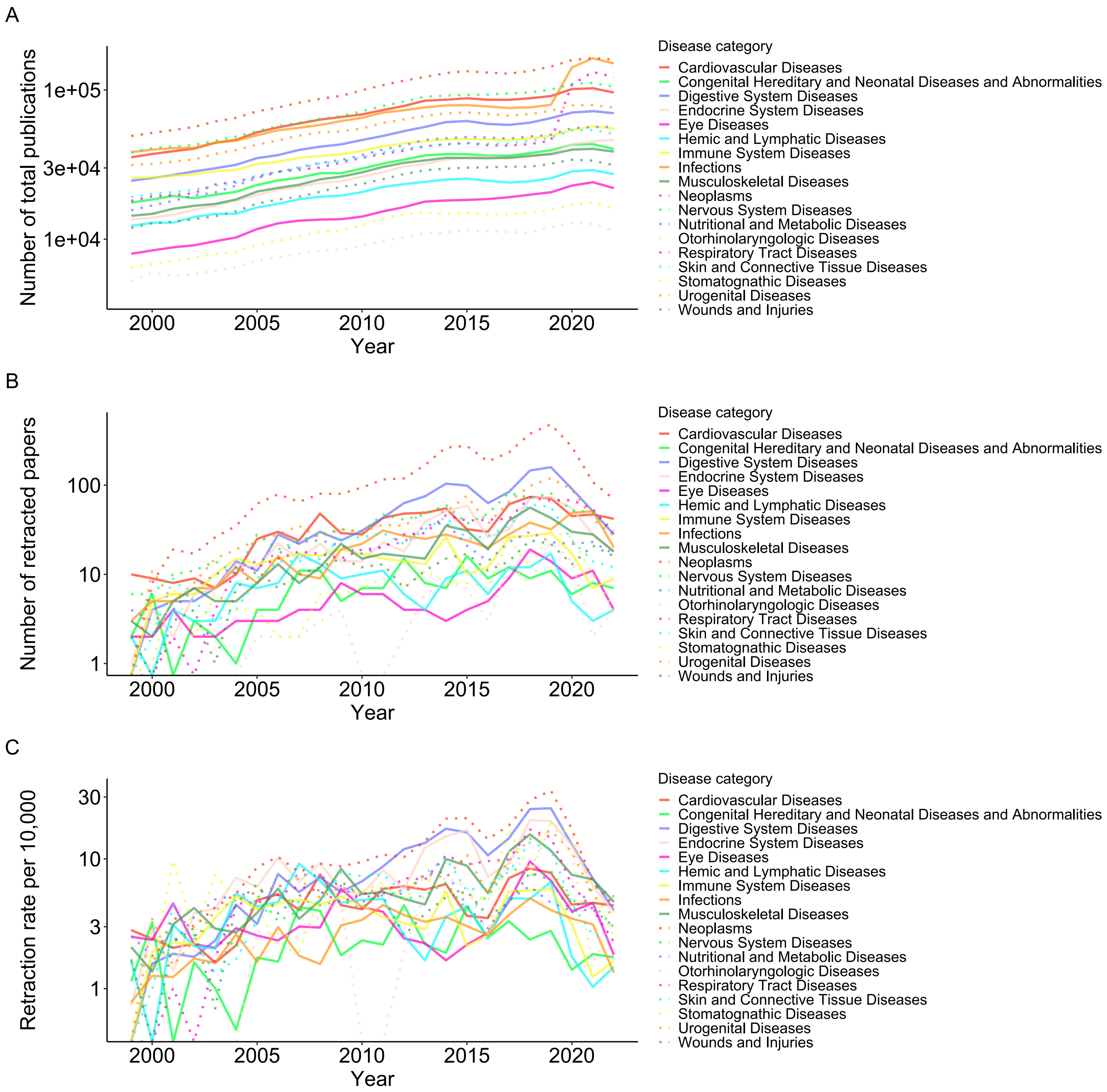

Supplementary Figure S3 Trend of retractions among 18 disease categories from 1999 to 2022 (log-scale).

The same data in Supplementary Figure S1 are shown in a logarithmic scale on the y-axis.

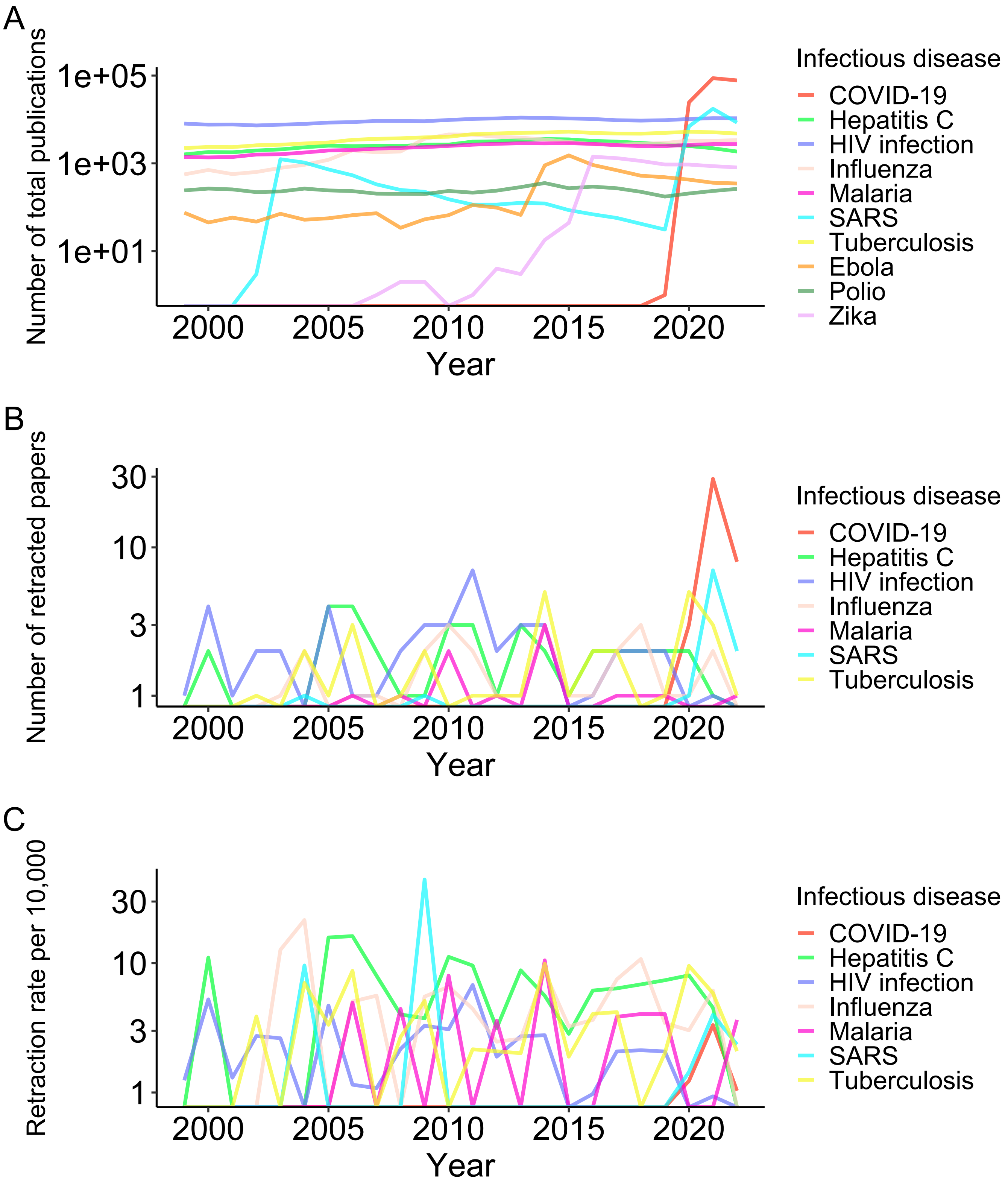

Supplementary Figure S4 Trend of retractions among ten infectious diseases from 1999 to 2022 (log-scale).

The same data in Figure 3 are shown in a logarithmic scale on the y-axis.

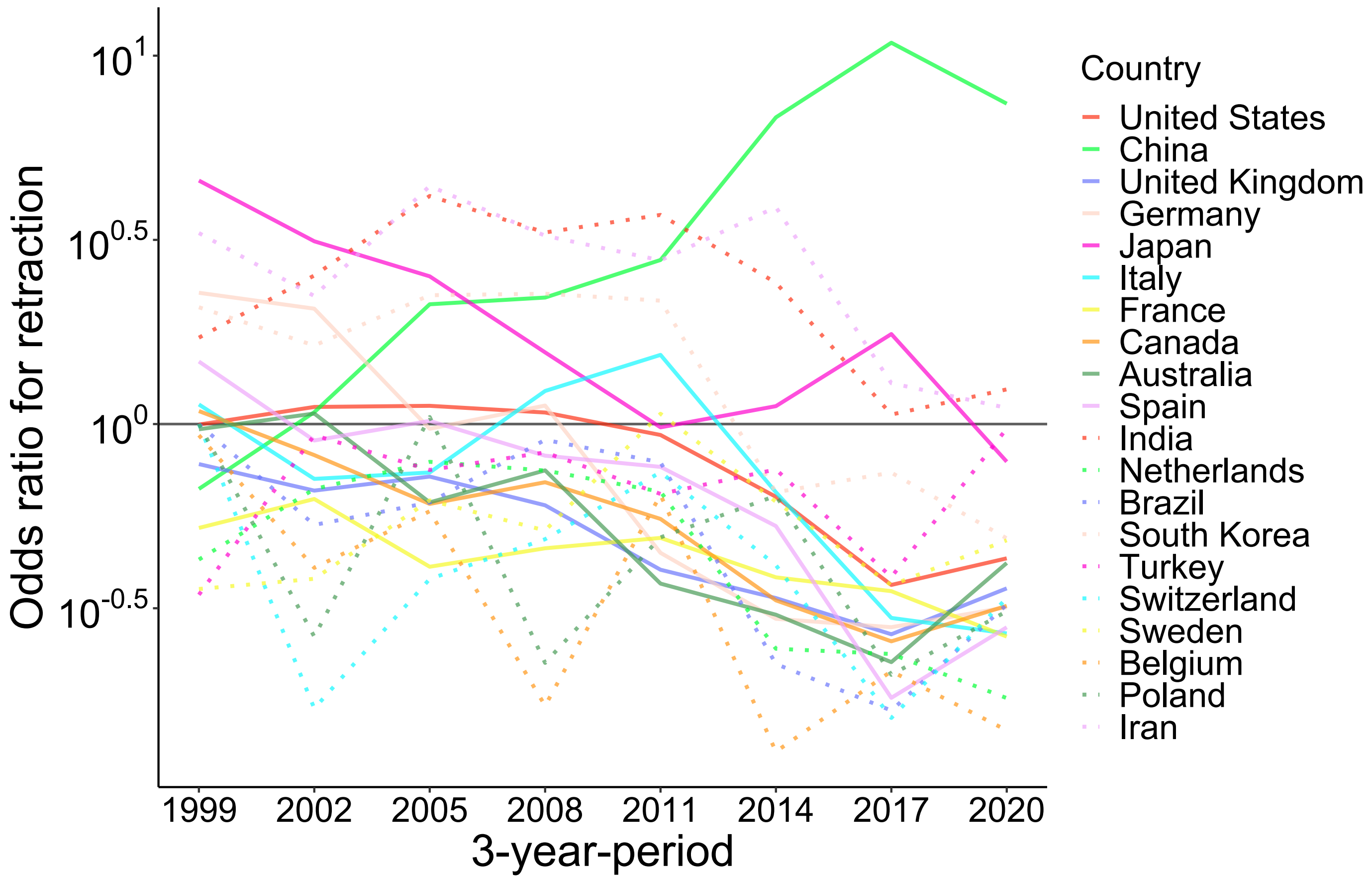

Supplementary Figure S5 Association of affiliated countries with retraction from 1999 to 2022 in top 20 publishing countries.

Odds ratios for retraction in 3-year-periods are shown for the top 20 countries with the highest publication counts.

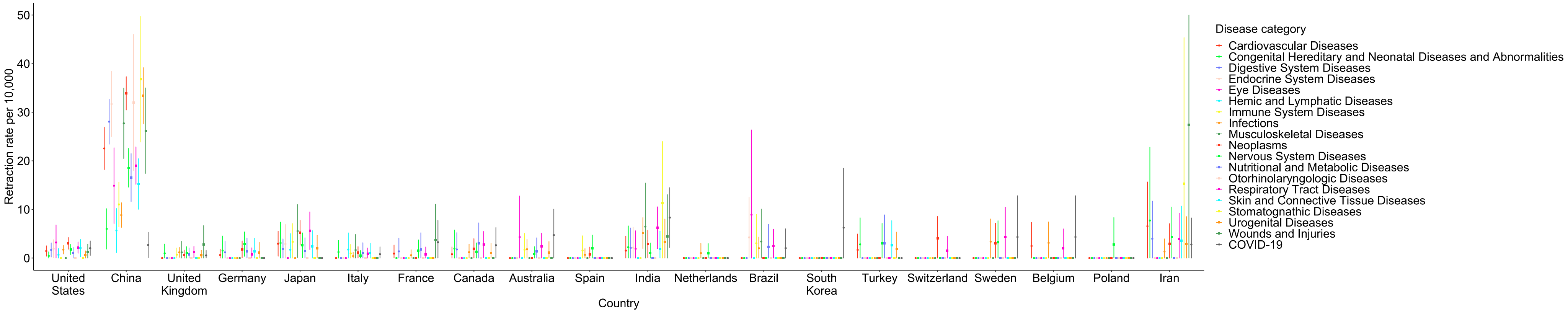

Supplementary Figure S6 Retraction rates by country and disease category in 2020–2022 , in top 20 publishing countries for 18+1 disease categories.

The retraction rates per 10,000 papers in 2020–2022 are shown for 18 disease categories plus COVID-19 in the top 20 countries with the highest publication counts. Vertical lines indicate 95% confidence intervals.

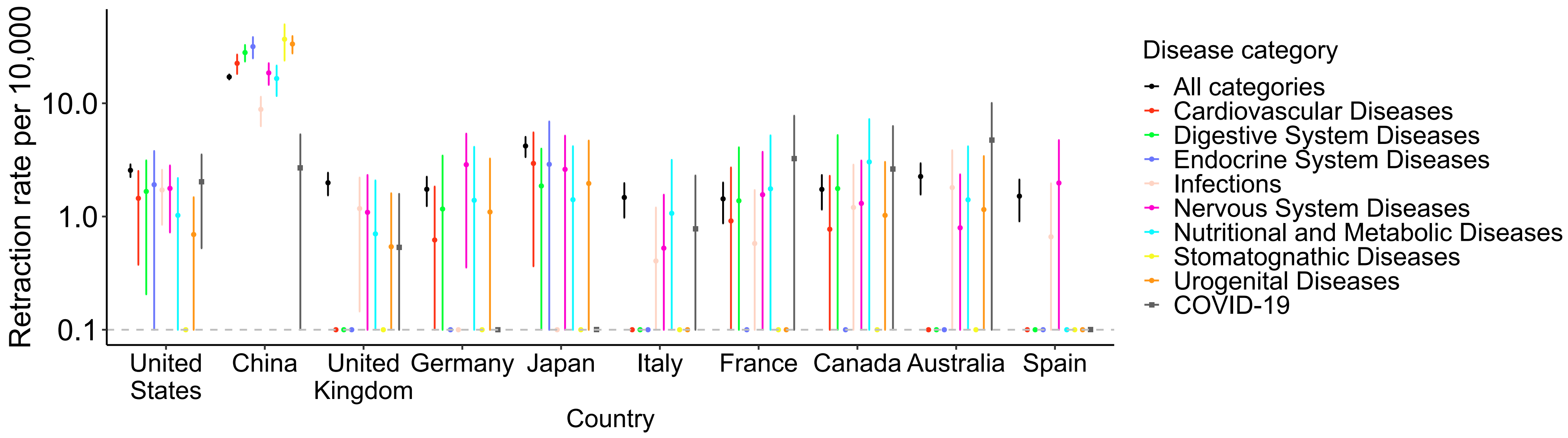

Supplementary Figure S7 Retraction rates by country and disease category in 2020–2022, in top 10 publishing countries for eight disease categories plus COVID-19 (log-scale).

The same data in Figure 5 are shown in a logarithmic scale on the y-axis. Where retraction numbers are zero, dots are on a broken line at the bottom.

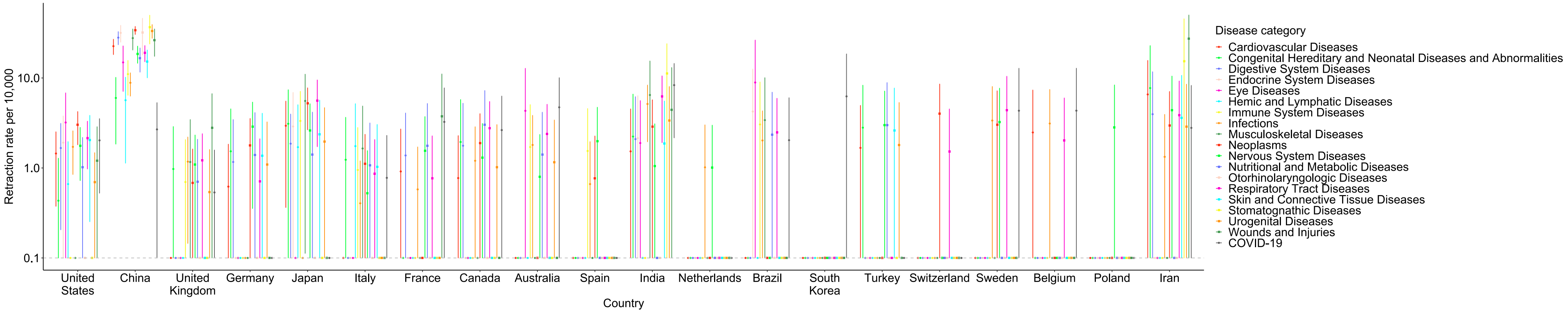

Supplementary Figure S8 Retraction rates by country and disease category in 2020–2022 , in top 20 publishing countries for 18+1 disease categories (log-scale).

The same data in Supplementary Figure S6 are shown in a logarithmic scale on the y-axis. Where retraction numbers are zero, dots are on a broken line at the bottom.

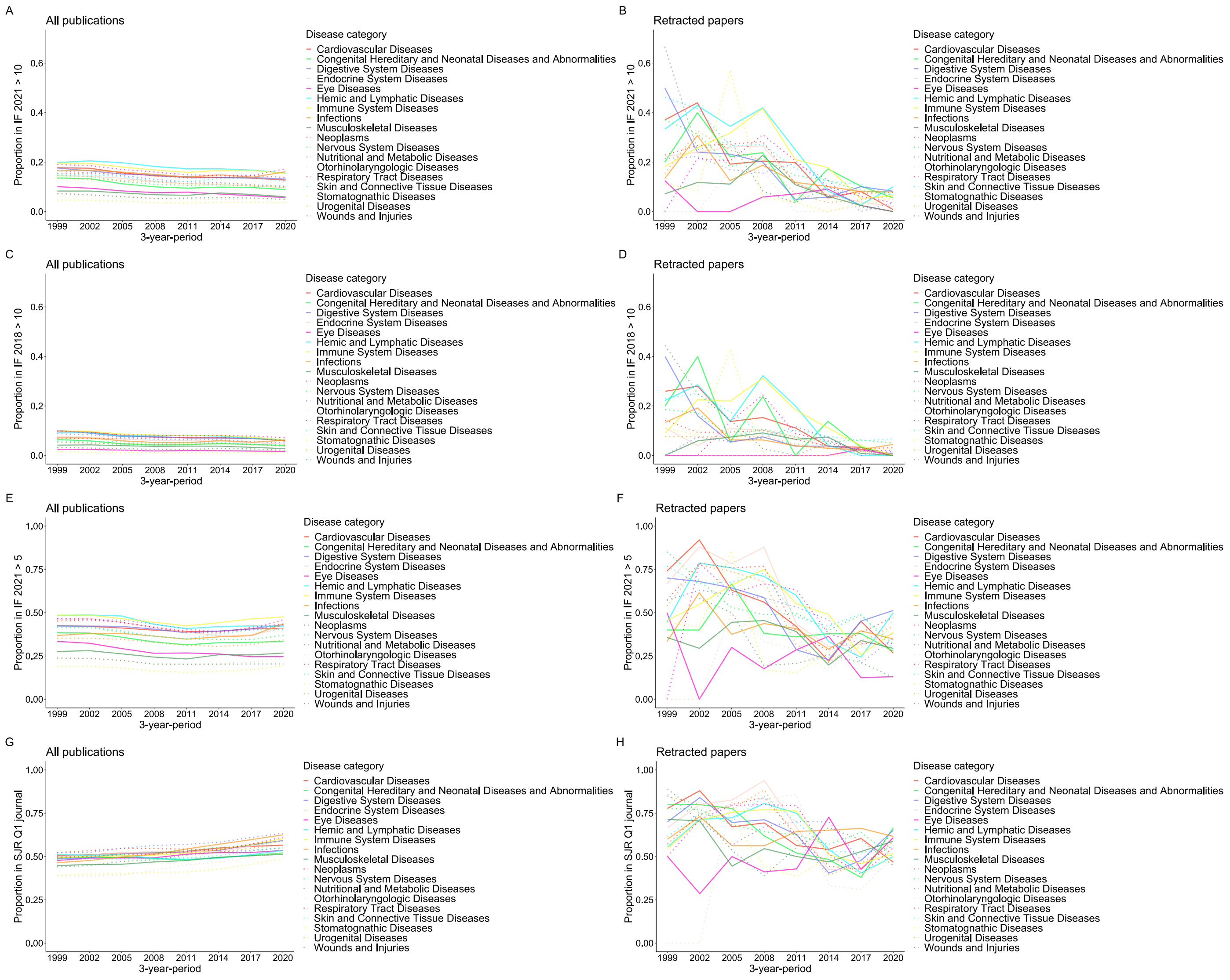

Supplementary Figure S9 Proportion of papers published in high-impact or "good" journals among 18 disease categories from 1999 to 2022.

The proportions of papers published in high-impact or "good" journals for total publications (A,C,E,G) and retracted papers (B,D,F,H) in 18 disease categories are shown in 3-year-periods. A and B) Impact Factor 2021 >10, C and D) Impact Factor 2018 >10, E and F) Impact Factor 2021 >5, G and H) SJR Q1 rank.

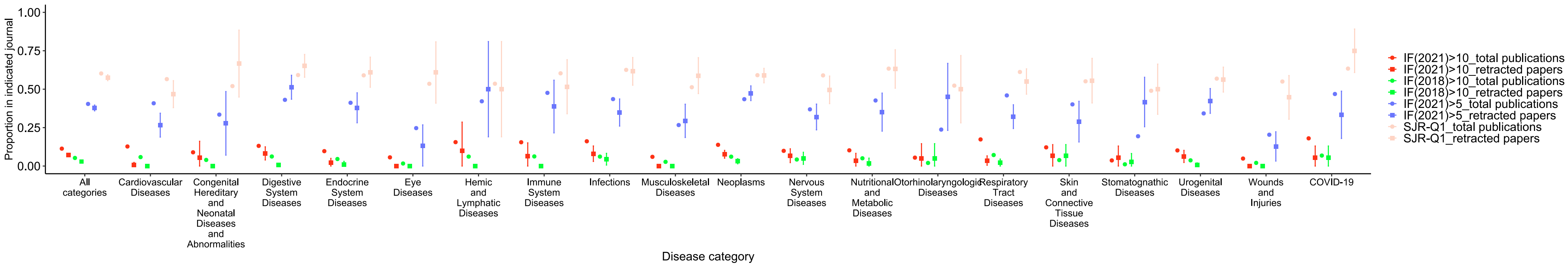

Supplementary Figure S10 Proportion of papers published in high-impact or "good" journals in 2000–2022 among 18+1 disease categories.

The proportions of papers published in high-impact or "good" journals (IF2021>10, IF2018>10, IF2021>5, or SJR Q1) for total publications and retracted papers in 2000–2022 are depicted for 18 disease categories plus COVID-19. Vertical lines indicate 95% confidence intervals.

Supplementary Table S1 Query terms used in the study.

Category	Query term
Retraction	("Retracted Publication"[Publication Type])
Publication year (e.g., 2020)	(2020[DP])
Cardiovascular Diseases	("Cardiovascular Diseases"[MeSH Major Topic])
Congenital, Hereditary, and Neonatal Diseases and Abnormalities	("Congenital, Hereditary, and Neonatal Diseases and Abnormalities"[MeSH Major Topic])
Digestive System Diseases	("Digestive System Diseases"[MeSH Major Topic])
Endocrine System Diseases	("Endocrine System Diseases"[MeSH Major Topic])
Eye Diseases	("Eye Diseases"[MeSH Major Topic])
Hemic and Lymphatic Diseases	("Hemic and Lymphatic Diseases"[MeSH Major Topic])
Immune System Diseases	("Immune System Diseases"[MeSH Major Topic])
Infections	("Infections"[MeSH Major Topic])
Musculoskeletal Diseases	("Musculoskeletal Diseases"[MeSH Major Topic])
Neoplasms	("Neoplasms"[MeSH Major Topic])
Nervous System Diseases	("Nervous System Diseases"[MeSH Major Topic])
Nutritional and Metabolic Diseases	("Nutritional and Metabolic Diseases"[MeSH Major Topic])
Otorhinolaryngologic Diseases	("Otorhinolaryngologic Diseases"[MeSH Major Topic])
Respiratory Tract Diseases	("Respiratory Tract Diseases"[MeSH Major Topic])
Skin and Connective Tissue Diseases	("Skin and Connective Tissue Diseases"[MeSH Major Topic])
Stomatognathic Diseases	("Stomatognathic Diseases"[MeSH Major Topic])
Urogenital Diseases	("Urogenital Diseases"[MeSH Major Topic])
Wounds and Injuries	("Wounds and Injuries"[MeSH Major Topic])
COVID-19	("SARS-CoV-2"[MeSH Major Topic] OR "COVID-19"[MeSH Major Topic])
Ebola	("Hemorrhagic Fever, Ebola"[MeSH Major Topic] OR "Ebolavirus"[MeSH Major Topic])
Hepatitis C	("Hepatitis C"[MeSH Major Topic] OR "Hepacivirus"[MeSH Major Topic])
HIV infection	("HIV Infections"[MeSH Major Topic] OR "HIV"[MeSH Major Topic])
Influenza	("Influenza, Human"[MeSH Major Topic] OR "Influenza A virus"[MeSH Major Topic])
Malaria	("Malaria"[MeSH Major Topic] OR "Plasmodium"[MeSH Major Topic])
Polio	("Poliomyelitis"[MeSH Major Topic] OR "Poliovirus"[MeSH Major Topic])
SARS	("Severe acute respiratory syndrome-related coronavirus"[MeSH Major Topic] OR "Severe Acute Respiratory Syndrome"[MeSH Major Topic])
Tuberculosis	("Tuberculosis"[MeSH Major Topic] OR "Mycobacterium tuberculosis"[MeSH Major Topic])
Zika	("Zika Virus Infection"[MeSH Major Topic] OR "Zika Virus"[MeSH Major Topic])
United States	("USA."[AD] OR "United States of America."[AD] OR "United States."[AD])
China	("China."[AD])

United Kingdom	("UK."[AD] OR "United Kingdom."[AD] OR "Britain."[AD] OR "Northern Ireland."[AD] OR "England."[AD] OR "Scotland."[AD] OR "Wales."[AD])
Germany	("Germany."[AD])
Japan	("Japan."[AD])
Italy	("Italy."[AD])
France	("France."[AD])
Canada	("Canada."[AD])
Australia	("Australia."[AD])
Spain	("Spain."[AD])
India	("India."[AD])
Netherlands	("Netherlands."[AD] OR "Netherland."[AD] OR "Holland."[AD])
Brazil	("Brazil."[AD])
South Korea	("South Korea."[AD] OR "Republic of Korea."[AD])
Turkey	("Turkey."[AD])
Switzerland	("Switzerland."[AD])
Sweden	("Sweden."[AD])
Belgium	("Belgium."[AD])
Poland	("Poland."[AD])
Iran	("Iran."[AD])
Impact factor (2021) >10	("0007-9235"[IS] OR "1542-4863"[IS] OR "0140-6736"[IS] OR "1474-547X"[IS] OR "0028-4793"[IS] OR "1533-4406"[IS] OR "0098-7484"[IS] OR "1538-3598"[IS] OR "1471-0072"[IS] OR "1471-0080"[IS] OR "1474-1776"[IS] OR "1474-1784"[IS] OR "1474-1733"[IS] OR "1474-1741"[IS] OR "2213-2600"[IS] OR "N/A"[IS] OR "0959-535X"[IS] OR "1756-1833"[IS] OR "1078-8956"[IS] OR "1546-170X"[IS] OR "N/A"[IS] OR "2666-5247"[IS] OR "1723-8617"[IS] OR "2051-5545"[IS] OR "1740-1526"[IS] OR "1740-1534"[IS] OR "2215-0374"[IS] OR "N/A"[IS] OR "2058-8437"[IS] OR "2058-8437"[IS] OR "1759-5045"[IS] OR "1759-5053"[IS] OR "2468-2667"[IS] OR "2468-2667"[IS] OR "0009-2665"[IS] OR "1520-6890"[IS] OR "1473-3099"[IS] OR "1474-4457"[IS] OR "1474-175X"[IS] OR "1474-1768"[IS] OR "0028-0836"[IS] OR "1476-4687"[IS] OR "1087-0156"[IS] OR "1546-1696"[IS] OR "2058-7546"[IS] OR "2058-7546"[IS] OR "0092-8674"[IS] OR "1097-4172"[IS] OR "2056-676X"[IS] OR "2056-676X"[IS] OR "1759-4774"[IS] OR "1759-4782"[IS] OR "0036-8075"[IS] OR "1095-9203"[IS] OR "0306-0012"[IS] OR "1460-4744"[IS] OR "1474-4422"[IS] OR "1474-4465"[IS] OR "1471-0056"[IS] OR "1471-0064"[IS] OR "1529-1006"[IS] OR "1539-6053"[IS] OR "1470-2045"[IS] OR "1474-5488"[IS] OR "0923-7534"[IS] OR "1569-8041"[IS] OR "0003-4819"[IS] OR "1539-3704"[IS] OR "0732-183X"[IS] OR "1527-7755"[IS] OR "0034-6861"[IS] OR "1539-0756"[IS] OR "0893-8512"[IS] OR "1098-6618"[IS] OR "1759-5002"[IS] OR "1759-5010"[IS] OR "0079-6425"[IS] OR "1873-2208"[IS] OR "1548-7091"[IS] OR "1548-7105"[IS] OR "1476-1122"[IS] OR "1476-4660"[IS] OR "1759-5029"[IS] OR "1759-5037"[IS] OR "0031-9333"[IS] OR "1522-1210"[IS] OR "1001-0602"[IS] OR "1748-7838"[IS] OR "2542-4351"[IS] OR "2542-4351"[IS] OR "N/A"[IS] OR "2468-1253"[IS] OR "2213-8587"[IS] OR "2213-8595"[IS] OR "1759-4758"[IS] OR "1759-4766"[IS] OR "2168-6106"[IS] OR "2168-6114"[IS] OR "1074-7613"[IS] OR "1097-4180"[IS] OR "2367-3613"[IS] OR "1433-8351"[IS] OR "1759-5061"[IS] OR "1759-507X"[IS] OR "0342-4642"[IS] OR "1432-1238"[IS] OR "N/A"[IS] OR "1476-4598"[IS] OR "1061-4036"[IS] OR "1546-1718"[IS] OR "2520-1158"[IS] OR "2520-1158"[IS] OR "1748-3387"[IS] OR "1748-3395"[IS] OR "0009-7322"[IS] OR "1524-4539"[IS] OR "1749-4885"[IS] OR "1749-4893"[IS] OR "1754-5692"[IS] OR "1754-5706"[IS] OR "1195-1982"[IS] OR "1708-8305"[IS] OR "2214-109X"[IS] OR "2214-109X"[IS] OR "1471-003X"[IS] OR "1471-0048"[IS] OR "0163-4453"[IS] OR "1532-2742"[IS] OR "1535-6108"[IS] OR "1878-3686"[IS] OR "2159-8274"[IS] OR "2159-8290"[IS] OR "2095-9907"[IS] OR "2059-3635"[IS] OR "N/A"[IS] OR "2056-5968"[IS] OR "2352-4642"[IS] OR "2352-4642"[IS] OR "1077-291X"[IS] OR "N/A"[IS] OR "0066-4146"[IS] OR "1545-4282"[IS] OR "N/A"[IS] OR "2662-138X"[IS] OR "N/A"[IS] OR "2589-7500"[IS] OR "1057-5987"[IS] OR "1545-8601"[IS] OR "N/A"[IS] OR "2522-5820"[IS] OR "0195-668X"[IS] OR "1522-9645"[IS] OR "0935-4956"[IS] OR "1432-0754"[IS] OR "2665-9913"[IS] OR "2665-9913"[IS] OR "0360-1285"[IS] OR "1873-216X"[IS] OR "0149-2195"[IS] OR "1545-861X"[IS] OR "2095-7467"[IS] OR "2054-9369"[IS] OR "N/A"[IS] OR "2397-3358"[IS] OR "0016-5085"[IS] OR "1528-0012"[IS] OR "N/A"[IS] OR "1553-877X"[IS] OR "0903-1936"[IS] OR "1399-3003"[IS] OR "0927-796X"[IS] OR "1879-212X"[IS] OR "2520-1131"[IS] OR "2520-1131"[IS] OR "2374-2437"[IS] OR "2374-2445"[IS] OR "2520-8489"[IS] OR "2520-8136"[IS] OR "0732-0582"[IS] OR "1545-3278"[IS] OR "1553-4006"[IS] OR "1553-4014"[IS] OR "1759-4790"[IS] OR "1759-4804"[IS] OR "0935-9648"[IS] OR "1521-4095"[IS] OR "0017-5749"[IS] OR "1468-3288"[IS] OR "1550-4131"[IS] OR "1932-7420"[IS] OR "1931-3128"[IS] OR "1934-6069"[IS] OR "0079-6700"[IS] OR "1873-1619"[IS] OR "1529-2908"[IS] OR "1529-2916"[IS] OR "2058-5276"[IS] OR "2058-5276"[IS] OR "2470-9468"[IS] OR "2470-9468"[IS] OR "1073-449X"[IS] OR "1535-4970"[IS] OR "0370-1573"[IS] OR "1873-6270"[IS] OR "2380-6583"[IS] OR "2380-6591"[IS] OR "2352-3026"[IS] OR "N/A"[IS] OR "0168-8278"[IS] OR "1600-0641"[IS] OR "2168-

	6149"[IS] OR "2168-6157"[IS] OR "1614-6832"[IS] OR "1614-6840"[IS] OR "2157-846X"[IS] OR "2157-846X"[IS] OR "0033-8419"[IS] OR "N/A"[IS] OR "1545-8636"[IS] OR "1545-8636"[IS] OR "1097-6256"[IS] OR "1546-1726"[IS] OR "N/A"[IS] OR "2542-5196"[IS] OR "2367-3648"[IS] OR "1614-4961"[IS] OR "1758-678X"[IS] OR "1758-6798"[IS] OR "1543-5008"[IS] OR "1545-2123"[IS] OR "1465-7392"[IS] OR "1476-4679"[IS] OR "0003-4967"[IS] OR "1468-2060"[IS] OR "0066-4308"[IS] OR "1545-2085"[IS] OR "2470-9476"[IS] OR "2470-9476"[IS] OR "0066-4154"[IS] OR "1545-4509"[IS] OR "0735-1097"[IS] OR "1558-3597"[IS] OR "2398-9629"[IS] OR "2398-9629"[IS] OR "2043-8206"[IS] OR "2043-8214"[IS] OR "1369-7021"[IS] OR "1873-4103"[IS] OR "2168-6203"[IS] OR "2168-6211"[IS] OR "2168-622X"[IS] OR "2168-6238"[IS] OR "N/A"[IS] OR "2522-5839"[IS] OR "2451-9294"[IS] OR "2451-9294"[IS] OR "0166-0616"[IS] OR "1872-9797"[IS] OR "0033-3190"[IS] OR "1423-0348"[IS] OR "0006-4971"[IS] OR "1528-0020"[IS] OR "0066-4189"[IS] OR "1545-4479"[IS] OR "1934-5909"[IS] OR "1875-9777"[IS] OR "0163-769X"[IS] OR "1945-7189"[IS] OR "8755-1209"[IS] OR "1944-9208"[IS] OR "1560-2745"[IS] OR "1878-9129"[IS] OR "1674-2818"[IS] OR "2049-3169"[IS] OR "0010-8545"[IS] OR "1873-3840"[IS] OR "N/A"[IS] OR "2567-3165"[IS] OR "1943-8206"[IS] OR "N/A"[IS] OR "1364-6613"[IS] OR "1879-307X"[IS] OR "0001-4842"[IS] OR "1520-4898"[IS] OR "0926-3373"[IS] OR "1873-3883"[IS] OR "0162-8828"[IS] OR "1939-3539"[IS] OR "1755-4330"[IS] OR "1755-4349"[IS] OR "0302-2838"[IS] OR "1873-7560"[IS] OR "2397-3374"[IS] OR "2397-3374"[IS] OR "2380-8195"[IS] OR "2380-8195"[IS] OR "1947-5454"[IS] OR "1947-5462"[IS] OR "0001-8732"[IS] OR "1460-6976"[IS] OR "2311-6706"[IS] OR "2150-5551"[IS] OR "0009-7330"[IS] OR "1524-4571"[IS] OR "2095-5138"[IS] OR "2053-714X"[IS] OR "N/A"[IS] OR "2662-1347"[IS] OR "N/A"[IS] OR "1756-8722"[IS] OR "0033-2909"[IS] OR "1939-1455"[IS] OR "1368-7646"[IS] OR "1532-2084"[IS] OR "0066-4170"[IS] OR "1545-4487"[IS] OR "N/A"[IS] OR "2589-5974"[IS] OR "0066-4278"[IS] OR "1545-1585"[IS] OR "1548-5943"[IS] OR "1548-5951"[IS] OR "1672-7681"[IS] OR "2042-0226"[IS] OR "1360-1385"[IS] OR "1878-4372"[IS] OR "1674-2052"[IS] OR "1752-9867"[IS] OR "0167-7799"[IS] OR "1879-3096"[IS] OR "0163-7525"[IS] OR "1545-2093"[IS] OR "N/A"[IS] OR "2637-9368"[IS] OR "1752-0894"[IS] OR "1752-0908"[IS] OR "0140-525X"[IS] OR "1469-1825"[IS] OR "1025-496X"[IS] OR "1560-7917"[IS] OR "0962-8924"[IS] OR "1879-3088"[IS] OR "1058-4838"[IS] OR "1537-6591"[IS] OR "2405-8297"[IS] OR "2405-8297"[IS] OR "0146-6615"[IS] OR "1096-9071"[IS] OR "0169-5347"[IS] OR "1872-8383"[IS] OR "2095-9273"[IS] OR "2095-9281"[IS] OR "1477-8939"[IS] OR "1873-0442"[IS] OR "N/A"[IS] OR "2662-1355"[IS] OR "2047-7538"[IS] OR "2047-7538"[IS] OR "1556-0864"[IS] OR "1556-1380"[IS] OR "2590-2393"[IS] OR "2590-2385"[IS] OR "1616-301X"[IS] OR "1616-3028"[IS] OR "N/A"[IS] OR "2522-5812"[IS] OR "N/A"[IS] OR "2662-1991"[IS] OR "1936-122X"[IS] OR "1936-1238"[IS] OR "1471-4906"[IS] OR "1471-4981"[IS] OR "1350-9462"[IS] OR "1873-1635"[IS] OR "1745-2473"[IS] OR "1745-2481"[IS] OR "N/A"[IS] OR "2222-1751"[IS] OR "1931-9401"[IS] OR "1931-9401"[IS] OR "0021-9738"[IS] OR "1558-8238"[IS] OR "1364-8535"[IS] OR "1466-609X"[IS] OR "1097-2765"[IS] OR "1097-4164"[IS] OR "1946-6234"[IS] OR "1946-6242"[IS] OR "0002-953X"[IS] OR "1535-7228"[IS] OR "1941-6520"[IS] OR "1941-6067"[IS] OR "0889-1591"[IS] OR "1090-2139"[IS] OR "2405-8025"[IS] OR "2405-8033"[IS] OR "0305-1048"[IS] OR "1362-4962"[IS] OR "2168-2267"[IS] OR "2168-2275"[IS] OR "2397-334X"[IS] OR "2397-334X"[IS] OR "2211-2855"[IS] OR "2211-3282"[IS] OR "0033-5533"[IS] OR "1531-4650"[IS] OR "0085-2538"[IS] OR "1523-1755"[IS] OR "1748-0132"[IS] OR "1878-044X"[IS] OR "0268-4012"[IS] OR "1873-4707"[IS] OR "0031-6997"[IS] OR "1521-0081"[IS] OR "N/A"[IS] OR "1750-1326"[IS] OR "2374-7943"[IS] OR "2374-7951"[IS] OR "0896-6273"[IS] OR "1097-4199"[IS] OR "0306-3674"[IS] OR "1473-0480"[IS] OR "1545-9993"[IS] OR "1545-9985"[IS] OR "0966-842X"[IS] OR "1878-4380"[IS] OR "1936-0851"[IS] OR "1936-086X"[IS] OR "0141-0768"[IS] OR "1758-1095"[IS] OR "1543-5938"[IS] OR "1545-2050"[IS] OR "1474-760X"[IS] OR "1474-760X"[IS] OR "0169-409X"[IS] OR "1872-8294"[IS] OR "0034-4885"[IS] OR "1361-6633"[IS] OR "0163-8998"[IS] OR "1545-4134"[IS] OR "N/A"[IS] OR "2041-1723"[IS] OR "0734-9750"[IS] OR "1873-1899"[IS] OR "1359-6101"[IS] OR "1879-0305"[IS] OR "0165-6147"[IS] OR "1873-3735"[IS] OR "0022-1007"[IS] OR "1540-9538"[IS] OR "1566-2535"[IS] OR "1872-6305"[IS] OR "N/A"[IS] OR "2198-3844"[IS] OR "0065-2156"[IS] OR "N/A"[IS] OR "1568-9972"[IS] OR "1873-0183"[IS] OR "2055-026X"[IS] OR "2055-0278"[IS] OR "1388-9842"[IS] OR "1879-0844"[IS] OR "N/A"[IS] OR "2001-3078"[IS] OR "0270-9139"[IS] OR "1527-3350"[IS] OR "1355-4786"[IS] OR "1460-2369"[IS] OR "1389-5567"[IS] OR "1873-2739"[IS] OR "0149-5992"[IS] OR "1935-5548"[IS] OR "N/A"[IS] OR "2589-5370"[IS] OR "1754-2189"[IS] OR "1750-2799"[IS] OR "1044-579X"[IS] OR "1096-3650"[IS] OR "2666-3791"[IS] OR "2666-3791"[IS] OR "0166-2236"[IS] OR "1878-108X"[IS] OR "N/A"[IS] OR "2452-199X"[IS] OR "0820-3946"[IS] OR "1488-2329"[IS] OR "2049-2618"[IS] OR "2049-2618"[IS] OR "1433-7851"[IS] OR "1521-3773"[IS] OR "1364-0321"[IS] OR "1879-0690"[IS] OR "1385-8947"[IS] OR "1873-3212"[IS] OR "2168-6254"[IS] OR "2168-6262"[IS] OR "1552-5260"[IS] OR "1552-5279"[IS] OR "1941-1405"[IS] OR "1941-0611"[IS] OR "2077-7000"[IS] OR "2077-7000"[IS] OR "1089-778X"[IS] OR "1941-0026"[IS] OR "0146-2806"[IS] OR "1535-6280"[IS] OR "0362-1642"[IS] OR "1545-4304"[IS] OR "1759-4812"[IS] OR "1759-4820"[IS] OR "0002-7863"[IS] OR "1520-5126"[IS] OR "0003-066X"[IS] OR "1935-990X"[IS] OR "0098-2997"[IS] OR "1872-9452"[IS] OR "0084-6597"[IS] OR "1545-4495"[IS] OR "0066-4227"[IS] OR "1545-3251"[IS] OR "1552-

	4450"[IS] OR "1552-4469"[IS] OR "1088-8683"[IS] OR "1532-7957"[IS] OR "1080-6040"[IS] OR "1080-6059"[IS] OR "2352-3018"[IS] OR "2352-3018"[IS] OR "1936-878X"[IS] OR "1876-7591"[IS] OR "0066-4219"[IS] OR "1545-326X"[IS] OR "1538-7933"[IS] OR "1538-7836"[IS] OR "0924-2244"[IS] OR "1879-3053"[IS] OR "0001-6322"[IS] OR "1432-0533"[IS] OR "1226-4601"[IS] OR "2055-7124"[IS] OR "2515-2459"[IS] OR "2515-2467"[IS] OR "1541-4337"[IS] OR "1541-4337"[IS] OR "0950-6608"[IS] OR "1743-2804"[IS] OR "2051-6347"[IS] OR "2051-6355"[IS] OR "2397-3366"[IS] OR "2397-3366"[IS] OR "0962-4929"[IS] OR "1474-0508"[IS] OR "0147-006X"[IS] OR "1545-4126"[IS] OR "0190-9622"[IS] OR "1097-6787"[IS] OR "2326-5191"[IS] OR "2326-5205"[IS] OR "0924-8579"[IS] OR "1872-7913"[IS] OR "2366-9608"[IS] OR "2366-9608"[IS] OR "0022-2429"[IS] OR "1547-7185"[IS] OR "2398-6352"[IS] OR "2398-6352"[IS] OR "1674-800X"[IS] OR "1674-8018"[IS] OR "0142-9612"[IS] OR "1878-5905"[IS] OR "N/A"[IS] OR "2523-3548"[IS] OR "1471-4914"[IS] OR "1471-499X"[IS] OR "1756-994X"[IS] OR "1756-994X"[IS] OR "0006-8950"[IS] OR "1460-2156"[IS] OR "1053-5888"[IS] OR "1558-0792"[IS] OR "0001-8686"[IS] OR "1873-3727"[IS] OR "0168-6445"[IS] OR "1574-6976"[IS] OR "1613-6810"[IS] OR "1613-6829"[IS] OR "0265-0568"[IS] OR "1460-4752"[IS] OR "1046-6673"[IS] OR "1533-3450"[IS] OR "2375-2548"[IS] OR "2375-2548"[IS] OR "2590-3330"[IS] OR "2590-3322"[IS] OR "0013-0095"[IS] OR "1944-8287"[IS] OR "0018-9219"[IS] OR "1558-2256"[IS] OR "0165-9936"[IS] OR "1879-3142"[IS] OR "0092-0703"[IS] OR "1552-7824"[IS] OR "2211-3835"[IS] OR "2211-3843"[IS] OR "1941-1413"[IS] OR "1941-1421"[IS] OR "0105-4538"[IS] OR "1398-9995"[IS] OR "0963-8687"[IS] OR "1873-1198"[IS] OR "1526-5161"[IS] OR "1536-0075"[IS] OR "0163-7827"[IS] OR "1873-2194"[IS] OR "2369-2960"[IS] OR "2369-2960"[IS] OR "2213-6657"[IS] OR "2213-6665"[IS] OR "1558-3724"[IS] OR "1558-3716"[IS] OR "2050-7488"[IS] OR "2050-7496"[IS] OR "0896-8411"[IS] OR "1095-9157"[IS] OR "1386-6532"[IS] OR "1873-5967"[IS] OR "2160-3308"[IS] OR "2160-3308"[IS] OR "N/A"[IS] OR "2057-3995"[IS] OR "1464-7931"[IS] OR "1469-185X"[IS] OR "1543-592X"[IS] OR "1545-2069"[IS] OR "0360-0300"[IS] OR "1557-7341"[IS] OR "0091-6749"[IS] OR "1097-6825"[IS] OR "1569-1705"[IS] OR "1572-9826"[IS] OR "0161-6420"[IS] OR "1549-4713"[IS] OR "0968-0004"[IS] OR "1362-4326"[IS] OR "2327-056X"[IS] OR "2327-0578"[IS] OR "1757-4676"[IS] OR "1757-4684"[IS] OR "2162-237X"[IS] OR "2162-2388"[IS] OR "0304-3894"[IS] OR "1873-3336"[IS] OR "0001-0782"[IS] OR "1557-7317"[IS] OR "0004-3702"[IS] OR "1872-7921"[IS] OR "1460-2725"[IS] OR "1460-2393"[IS] OR "2095-1779"[IS] OR "2214-0883"[IS] OR "0261-4189"[IS] OR "1460-2075"[IS] OR "1467-5463"[IS] OR "1477-4054"[IS] OR "1949-3045"[IS] OR "1949-3045"[IS] OR "1531-7331"[IS] OR "1545-4118"[IS] OR "0026-0495"[IS] OR "1532-8600"[IS] OR "2473-2397"[IS] OR "2168-6831"[IS] OR "1876-2018"[IS] OR "1876-2026"[IS] OR "0363-7425"[IS] OR "1930-3807"[IS] OR "0034-4257"[IS] OR "1879-0704"[IS] OR "0042-9686"[IS] OR "1564-0604"[IS] OR "1361-8415"[IS] OR "1361-8423"[IS] OR "0066-4197"[IS] OR "1545-2948"[IS] OR "1078-0432"[IS] OR "1557-3265"[IS] OR "0003-4932"[IS] OR "1528-1140"[IS] OR "1540-9295"[IS] OR "1540-9309"[IS] OR "0921-3449"[IS] OR "1879-0658"[IS] OR "0887-6185"[IS] OR "1873-7897"[IS] OR "2155-5435"[IS] OR "2155-5435"[IS] OR "2590-1168"[IS] OR "2590-1168"[IS] OR "0022-3050"[IS] OR "1468-330X"[IS] OR "2214-1588"[IS] OR "2214-1588"[IS] OR "1610-3653"[IS] OR "1610-3661"[IS] OR "1556-6072"[IS] OR "1556-6080"[IS] OR "0305-7372"[IS] OR "1532-1967"[IS] OR "0161-4940"[IS] OR "1520-5703"[IS] OR "2095-4956"[IS] OR "2095-4956"[IS] OR "N/A"[IS] OR "2577-5421"[IS] OR "1542-3565"[IS] OR "1542-7714"[IS] OR "1053-2498"[IS] OR "1557-3117"[IS] OR "0034-6543"[IS] OR "1935-1046"[IS] OR "1362-0347"[IS] OR "1468-960X"[IS] OR "0149-2063"[IS] OR "1557-1211"[IS] OR "N/A"[IS] OR "2575-0356"[IS] OR "1359-4184"[IS] OR "1476-5578"[IS] OR "0167-5729"[IS] OR "1879-274X"[IS] OR "1534-5807"[IS] OR "1878-1551"[IS] OR "1743-9191"[IS] OR "1743-9159"[IS] OR "0163-7258"[IS] OR "1879-016X"[IS] OR "0043-1354"[IS] OR "1879-2448"[IS] OR "1554-8627"[IS] OR "1554-8635"[IS] OR "0920-5691"[IS] OR "1573-1405"[IS] OR "2588-8420"[IS] OR "2588-8420"[IS] OR "2095-4700"[IS] OR "2095-6231"[IS] OR "2574-3805"[IS] OR "2574-3805"[IS] OR "0160-4120"[IS] OR "1873-6750"[IS] OR "2165-8102"[IS] OR "2165-8110"[IS] OR "2472-3428"[IS] OR "2472-3428"[IS] OR "0008-5472"[IS] OR "1538-7445"[IS] OR "1198-743X"[IS] OR "1469-0691"[IS] OR "1554-0669"[IS] OR "1554-0677"[IS] OR "0361-8609"[IS] OR "1096-8652"[IS] OR "1467-7644"[IS] OR "1467-7652"[IS] OR "1354-1013"[IS] OR "1365-2486"[IS] OR "0883-9026"[IS] OR "1873-2003"[IS] OR "0890-8567"[IS] OR "1527-5418"[IS] OR "0008-6363"[IS] OR "1755-3245"[IS] OR "0733-8716"[IS] OR "1558-0008"[IS] OR "2095-2546"[IS] OR "2213-2961"[IS] OR "0954-6820"[IS] OR "1365-2796"[IS] OR "1744-4292"[IS] OR "1744-4292"[IS] OR "1092-2172"[IS] OR "1098-5557"[IS] OR "1522-8517"[IS] OR "1523-5866"[IS] OR "2524-7921"[IS] OR "2524-793X"[IS] OR "0253-9837"[IS] OR "1872-2067"[IS] OR "1525-0016"[IS] OR "1525-0024"[IS] OR "0022-0515"[IS] OR "2328-8175"[IS] OR "0003-2409"[IS] OR "1365-2044"[IS] OR "0890-9369"[IS] OR "1549-5477"[IS] OR "0887-6924"[IS] OR "1476-5551"[IS] OR "0261-5177"[IS] OR "1879-3193"[IS] OR "1359-0286"[IS] OR "1879-0348"[IS] OR "0160-7383"[IS] OR "1873-7722"[IS] OR "2095-8099"[IS] OR "2096-0026"[IS] OR "2090-1232"[IS] OR "2090-1224"[IS] OR "0006-3223"[IS] OR "1873-2402"[IS] OR "0002-0729"[IS] OR "1468-2834"[IS] OR "2096-2797"[IS] OR "2468-0257"[IS] OR "0027-8424"[IS] OR "1091-6490"[IS] OR "1536-1284"[IS] OR "1558-0687"[IS] OR "0025-729X"[IS] OR "1326-5377"[IS] OR "1021-7770"[IS] OR "1423-0127"[IS] OR "1540-

	1405"[IS] OR "1540-1413"[IS] OR "N/A"[IS] OR "1756-9966"[IS] OR "2327-0608"[IS] OR "2327-0616"[IS] OR "0956-5663"[IS] OR "1873-4235"[IS] OR "2213-1779"[IS] OR "2213-1787"[IS] OR "0001-8392"[IS] OR "1930-3815"[IS] OR "N/A"[IS] OR "2051-1426"[IS] OR "0003-1224"[IS] OR "1939-8271"[IS] OR "0393-2990"[IS] OR "1573-7284"[IS] OR "2235-1795"[IS] OR "1664-5553"[IS] OR "0146-6410"[IS] OR "1873-2224"[IS] OR "0300-8428"[IS] OR "1435-1803"[IS] OR "1936-1327"[IS] OR "1936-1335"[IS] OR "0198-6325"[IS] OR "1098-1128"[IS] OR "0302-282X"[IS] OR "1423-0224"[IS] OR "1530-6984"[IS] OR "1530-6992"[IS] OR "N/A"[IS] OR "2057-3960"[IS] OR "1063-6706"[IS] OR "1941-0034"[IS] OR "0906-6713"[IS] OR "1600-0757"[IS] OR "N/A"[IS] OR "2567-3173"[IS] OR "2059-7037"[IS] OR "2059-7037"[IS] OR "0009-9147"[IS] OR "1530-8561"[IS] OR "1226-3613"[IS] OR "2092-6413"[IS] OR "1323-1316"[IS] OR "1440-1819"[IS] OR "0017-8012"[IS] OR "0017-8012"[IS] OR "1040-4651"[IS] OR "1532-298X"[IS] OR "0001-8791"[IS] OR "1095-9084"[IS] OR "0742-3098"[IS] OR "1600-079X"[IS] OR "1094-2939"[IS] OR "1545-1577"[IS] OR "1201-9712"[IS] OR "1878-3511"[IS] OR "1350-9047"[IS] OR "1476-5403"[IS] OR "2190-5991"[IS] OR "2190-6009"[IS] OR "0002-9270"[IS] OR "1572-0241"[IS] OR "0012-8252"[IS] OR "1872-6828"[IS] OR "2326-6066"[IS] OR "2326-6074"[IS] OR "N/A"[IS] OR "2397-4621"[IS] OR "1469-493X"[IS] OR "1361-6137"[IS] OR "0008-8846"[IS] OR "1873-3948"[IS] OR "2573-5144"[IS] OR "2573-5144"[IS] OR "0112-1642"[IS] OR "1179-2035"[IS] OR "1081-0706"[IS] OR "1530-8995"[IS] OR "0960-8524"[IS] OR "1873-2976"[IS] OR "1548-9213"[IS] OR "1548-9221"[IS] OR "0168-9525"[IS] OR "1362-4555"[IS] OR "2168-6068"[IS] OR "2168-6084"[IS] OR "0027-8874"[IS] OR "1460-2105"[IS] OR "1866-3508"[IS] OR "1866-3516"[IS] OR "2213-9567"[IS] OR "2213-9567"[IS] OR "2522-0128"[IS] OR "2522-0136"[IS] OR "0021-9010"[IS] OR "1939-1854"[IS] OR "0028-3878"[IS] OR "1526-632X"[IS] OR "1568-1637"[IS] OR "1872-9649"[IS] OR "0924-2716"[IS] OR "1872-8235"[IS] OR "1863-2297"[IS] OR "1863-2300"[IS] OR "1064-3389"[IS] OR "1547-6537"[IS] OR "0007-0912"[IS] OR "1471-6771"[IS] OR "2467-964X"[IS] OR "2452-414X"[IS] OR "2055-6756"[IS] OR "2055-6764"[IS] OR "0008-1256"[IS] OR "2162-8564"[IS] OR "0095-1137"[IS] OR "1098-660X"[IS] OR "0031-5850"[IS] OR "N/A"[IS] OR "1551-3203"[IS] OR "1941-0050"[IS] OR "2214-8604"[IS] OR "2214-7810"[IS] OR "1745-6916"[IS] OR "1745-6924"[IS] OR "1549-1277"[IS] OR "1549-1676"[IS] OR "1838-7640"[IS] OR "1838-7640"[IS] OR "2161-8313"[IS] OR "2156-5376"[IS] OR "0006-3568"[IS] OR "1525-3244"[IS] OR "0090-0036"[IS] OR "1541-0048"[IS] OR "2328-8930"[IS] OR "N/A"[IS] OR "1557-1874"[IS] OR "1557-1882"[IS] OR "2226-4108"[IS] OR "2227-8508"[IS] OR "0196-8904"[IS] OR "1879-2227"[IS] OR "0268-005X"[IS] OR "1873-7137"[IS] OR "1759-0876"[IS] OR "1759-0884"[IS] OR "0002-8282"[IS] OR "1944-7981"[IS] OR "2168-2216"[IS] OR "2168-2232"[IS] OR "0168-3659"[IS] OR "1873-4995"[IS] OR "2524-7972"[IS] OR "2524-7867"[IS] OR "0306-2619"[IS] OR "1872-9118"[IS] OR "0012-6667"[IS] OR "1179-1950"[IS] OR "0304-419X"[IS] OR "1879-2561"[IS] OR "1087-0792"[IS] OR "1532-2955"[IS] OR "0272-7358"[IS] OR "1873-7811"[IS] OR "0166-4972"[IS] OR "1879-2383"[IS] OR "2332-7812"[IS] OR "2332-7812"[IS] OR "0013-936X"[IS] OR "1520-5851"[IS] OR "1461-6688"[IS] OR "1470-1340"[IS] OR "0008-3976"[IS] OR "1744-7976"[IS] OR "0003-6900"[IS] OR "2379-0407"[IS] OR "N/A"[IS] OR "2688-4062"[IS] OR "1523-9829"[IS] OR "1545-4274"[IS] OR "1359-8368"[IS] OR "1879-1069"[IS] OR "1094-9968"[IS] OR "1520-6653"[IS] OR "0008-6223"[IS] OR "1873-3891"[IS] OR "0033-0620"[IS] OR "1873-1740"[IS] OR "0364-5134"[IS] OR "1531-8249"[IS] OR "1461-023X"[IS] OR "1461-0248"[IS] OR "1359-8546"[IS] OR "1758-6852"[IS] OR "0925-5273"[IS] OR "1873-7579"[IS] OR "0166-3615"[IS] OR "1872-6194"[IS] OR "0165-1781"[IS] OR "1872-7123"[IS] OR "2530-7614"[IS] OR "2444-569X"[IS] OR "1751-7362"[IS] OR "1751-7370"[IS] OR "0011-9164"[IS] OR "1873-4464"[IS] OR "1040-8398"[IS] OR "1549-7852"[IS] OR "2352-3964"[IS] OR "2352-3964"[IS] OR "0022-4359"[IS] OR "1873-3271"[IS] OR "0360-1315"[IS] OR "1873-782X"[IS] OR "1040-8436"[IS] OR "1547-6561"[IS] OR "2055-6837"[IS] OR "2055-6845"[IS] OR "N/A"[IS] OR "2639-4979"[IS] OR "0959-3780"[IS] OR "1872-9495"[IS] OR "1741-7015"[IS] OR "1741-7015"[IS] OR "0007-1323"[IS] OR "1365-2168"[IS] OR "0007-0963"[IS] OR "1365-2133"[IS] OR "0025-6196"[IS] OR "1942-5546"[IS] OR "0047-2506"[IS] OR "1478-6990"[IS] OR "2192-2640"[IS] OR "2192-2659"[IS] OR "2405-4712"[IS] OR "2405-4720"[IS] OR "0161-5505"[IS] OR "1535-5667"[IS] OR "1936-8798"[IS] OR "1876-7605"[IS] OR "0959-6526"[IS] OR "1879-1786"[IS] OR "0272-6386"[IS] OR "1523-6838"[IS] OR "1077-5552"[IS] OR "1077-5552"[IS] OR "1758-5082"[IS] OR "1758-5090"[IS] OR "0390-6078"[IS] OR "N/A"[IS] OR "1052-9276"[IS] OR "1099-1654"[IS] OR "0002-9297"[IS] OR "1537-6605"[IS] OR "1057-7149"[IS] OR "1941-0042"[IS] OR "0278-0062"[IS] OR "1558-254X"[IS] OR "N/A"[IS] OR "2639-5274"[IS] OR "0091-6765"[IS] OR "1552-9924"[IS] OR "1463-9262"[IS] OR "1463-9270"[IS] OR "2213-2198"[IS] OR "2213-2201"[IS] OR "2542-5293"[IS] OR "2542-5293"[IS] OR "1939-1374"[IS] OR "1939-1374"[IS] OR "1474-9718"[IS] OR "1474-9726"[IS] OR "0105-2896"[IS] OR "1600-065X"[IS] OR "0001-4273"[IS] OR "1948-0989"[IS] OR "2198-6436"[IS] OR "2198-6436"[IS] OR "0969-6989"[IS] OR "1873-1384"[IS] OR "0148-2963"[IS] OR "1873-7978"[IS] OR "1745-2759"[IS] OR "1745-2767"[IS] OR "1863-8880"[IS] OR "1863-8899"[IS] OR "1082-989X"[IS] OR "1939-1463"[IS] OR "0960-9822"[IS] OR "1879-0445"[IS] OR "0301-0082"[IS] OR "1873-5118"[IS] OR "0040-1625"[IS] OR "1873-5509"[IS] OR "1467-7881"[IS] OR "1467-789X"[IS] OR "0066-4286"[IS] OR "1545-2107"[IS] OR "N/A"[IS] OR "2196-1115"[IS] OR "1080-0549"[IS] OR "1559-0267"[IS] OR "0964-

	4733"[IS] OR "1099-0836"[IS] OR "2213-2317"[IS] OR "2213-2317"[IS] OR "0363-9762"[IS] OR "1536-0229"[IS] OR "1884-4049"[IS] OR "1884-4057"[IS] OR "2590-0064"[IS] OR "2590-0064"[IS] OR "N/A"[IS] OR "2056-6387"[IS] OR "0048-9697"[IS] OR "1879-1026"[IS] OR "1449-2288"[IS] OR "1449-2288"[IS] OR "0144-8617"[IS] OR "1879-1344"[IS] OR "1836-9553"[IS] OR "1836-9561"[IS] OR "1367-5788"[IS] OR "1872-9088"[IS] OR "2210-6707"[IS] OR "2210-6715"[IS] OR "0002-9378"[IS] OR "1097-6868"[IS] OR "N/A"[IS] OR "2380-6761"[IS] OR "2214-9937"[IS] OR "2214-9937"[IS] OR "1044-5323"[IS] OR "1096-3618"[IS] OR "0007-1250"[IS] OR "1472-1465"[IS] OR "1053-4822"[IS] OR "1873-7889"[IS] OR "0969-6970"[IS] OR "1573-7209"[IS] OR "2334-2536"[IS] OR "2334-2536"[IS] OR "1742-7061"[IS] OR "1878-7568"[IS] OR "0268-960X"[IS] OR "1532-1681"[IS] OR "1753-5123"[IS] OR "1753-5131"[IS] OR "1555-9041"[IS] OR "1555-905X"[IS] OR "0033-2917"[IS] OR "1469-8978"[IS] OR "1043-2760"[IS] OR "1879-3061"[IS] OR "1478-0887"[IS] OR "1478-0895"[IS] OR "0007-6813"[IS] OR "1873-6068"[IS] OR "0376-7388"[IS] OR "1873-3123"[IS] OR "1471-4922"[IS] OR "1471-5007"[IS] OR "0926-5805"[IS] OR "1872-7891"[IS] OR "N/A"[IS] OR "2397-7132"[IS] OR "0098-7921"[IS] OR "1728-4457"[IS] OR "1079-5642"[IS] OR "1524-4636"[IS] OR "0897-4756"[IS] OR "1520-5002"[IS] OR "2367-2617"[IS] OR "2367-0983"[IS] OR "2157-6904"[IS] OR "2157-6912"[IS] OR "2095-5162"[IS] OR "2049-9957"[IS] OR "0020-7640"[IS] OR "1741-2854"[IS] OR "0012-186X"[IS] OR "1432-0428"[IS] OR "1941-3289"[IS] OR "1941-3297"[IS] OR "0945-053X"[IS] OR "1569-1802"[IS] OR "0278-4319"[IS] OR "1873-4693"[IS] OR "N/A"[IS] OR "1880-8190"[IS] OR "1569-9056"[IS] OR "1878-1500"[IS] OR "0049-3848"[IS] OR "1879-2472"[IS] OR "2330-8249"[IS] OR "2330-8257"[IS] OR "N/A"[IS] OR "1744-8603"[IS] OR "0016-5107"[IS] OR "1097-6779"[IS] OR "1944-8244"[IS] OR "1944-8252"[IS] OR "1674-7305"[IS] OR "1869-1889"[IS] OR "1043-6618"[IS] OR "1096-1186"[IS] OR "0079-6727"[IS] OR "1873-1627"[IS] OR "0890-6955"[IS] OR "1879-2170"[IS] OR "0378-7206"[IS] OR "1872-7530"[IS] OR "0028-646X"[IS] OR "1469-8137"[IS] OR "1005-0302"[IS] OR "1941-1162"[IS] OR "2110-5820"[IS] OR "2110-5820"[IS] OR "0890-8044"[IS] OR "1558-156X"[IS] OR "0958-1669"[IS] OR "1879-0429"[IS] OR "1949-3053"[IS] OR "1949-3061"[IS] OR "1684-1182"[IS] OR "1995-9133"[IS] OR "1998-0124"[IS] OR "1998-0000"[IS] OR "2210-6502"[IS] OR "2210-6510"[IS] OR "0012-3692"[IS] OR "1931-3543"[IS] OR "2327-4662"[IS] OR "2327-4662"[IS] OR "1726-2135"[IS] OR "1684-8799"[IS] OR "1747-938X"[IS] OR "1878-0385"[IS] OR "1521-6616"[IS] OR "1521-7035"[IS] OR "0144-1647"[IS] OR "1464-5327"[IS] OR "2162-2531"[IS] OR "2162-2531"[IS] OR "2040-7122"[IS] OR "2040-7130"[IS] OR "0956-7976"[IS] OR "1467-9280"[IS] OR "1931-5244"[IS] OR "1878-1810"[IS] OR "0039-2499"[IS] OR "1524-4628"[IS] OR "1674-7291"[IS] OR "1869-1870"[IS] OR "0065-3195"[IS] OR "1436-5030"[IS] OR "1449-4035"[IS] OR "1839-3373"[IS] OR "0166-3542"[IS] OR "1872-9096"[IS] OR "0736-5845"[IS] OR "1879-2537"[IS] OR "N/A"[IS] OR "2397-768X"[IS] OR "0894-3796"[IS] OR "1099-1379"[IS] OR "1757-7780"[IS] OR "1757-7799"[IS] OR "1755-263X"[IS] OR "1755-263X"[IS] OR "1093-9687"[IS] OR "1467-8667"[IS] OR "0020-9996"[IS] OR "1536-0210"[IS] OR "1619-7070"[IS] OR "1619-7089"[IS] OR "1094-6705"[IS] OR "1552-7379"[IS] OR "2195-1071"[IS] OR "2195-1071"[IS] OR "1366-5545"[IS] OR "1878-5794"[IS] OR "2196-5404"[IS] OR "2196-5404"[IS] OR "2631-8644"[IS] OR "2631-7990"[IS] OR "0360-0572"[IS] OR "1545-2115"[IS] OR "1873-9946"[IS] OR "1876-4479"[IS] OR "2211-467X"[IS] OR "2211-4688"[IS] OR "0959-8049"[IS] OR "1879-0852"[IS])
Impact factor (2018) >10	("0007-9235"[IS] OR "1542-4863"[IS] OR "2058-8437"[IS] OR "2058-8437"[IS] OR "0028-4793"[IS] OR "1533-4406"[IS] OR "0140-6736"[IS] OR "1474-547X"[IS] OR "1474-1776"[IS] OR "1474-1784"[IS] OR "0009-2665"[IS] OR "1520-6890"[IS] OR "2058-7546"[IS] OR "2058-7546"[IS] OR "1474-175X"[IS] OR "1474-1768"[IS] OR "0098-7484"[IS] OR "1538-3598"[IS] OR "1474-1733"[IS] OR "1474-1741"[IS] OR "1471-0056"[IS] OR "1471-0064"[IS] OR "1471-0072"[IS] OR "1471-0080"[IS] OR "0028-0836"[IS] OR "1476-4687"[IS] OR "0036-8075"[IS] OR "1095-9203"[IS] OR "0306-0012"[IS] OR "1460-4744"[IS] OR "1476-1122"[IS] OR "1476-4660"[IS] OR "0034-6861"[IS] OR "1539-0756"[IS] OR "0092-8674"[IS] OR "1097-4172"[IS] OR "1470-2045"[IS] OR "1474-5488"[IS] OR "1740-1526"[IS] OR "1740-1534"[IS] OR "1759-4774"[IS] OR "1759-4782"[IS] OR "1723-8617"[IS] OR "2051-5545"[IS] OR "1723-8617"[IS] OR "2051-5545"[IS] OR "1748-3387"[IS] OR "1748-3395"[IS] OR "1754-5692"[IS] OR "1754-5706"[IS] OR "1471-003X"[IS] OR "1471-0048"[IS] OR "0066-4146"[IS] OR "1545-4282"[IS] OR "2056-676X"[IS] OR "2056-676X"[IS] OR "1087-0156"[IS] OR "1546-1696"[IS] OR "1749-4885"[IS] OR "1749-4893"[IS] OR "1078-8956"[IS] OR "1546-170X"[IS] OR "N/A"[IS] OR "2397-3358"[IS] OR "1474-4422"[IS] OR "1474-4465"[IS] OR "1548-7091"[IS] OR "1548-7105"[IS] OR "0370-1573"[IS] OR "1873-6270"[IS] OR "0732-183X"[IS] OR "1527-7755"[IS] OR "2367-3613"[IS] OR "1433-8351"[IS] OR "0959-535X"[IS] OR "1756-1833"[IS] OR "1473-3099"[IS] OR "1474-4457"[IS] OR "0066-4154"[IS] OR "1545-4509"[IS] OR "0360-1285"[IS] OR "1873-216X"[IS] OR "2159-8274"[IS] OR "2159-8290"[IS] OR "0001-8732"[IS] OR "1460-6976"[IS] OR "0935-9648"[IS] OR "1521-4095"[IS] OR "1061-4036"[IS] OR "1546-1718"[IS] OR "1614-6832"[IS] OR "1614-6840"[IS] OR "1759-5029"[IS] OR "1759-5037"[IS] OR "2213-8587"[IS] OR "2213-8595"[IS] OR "0079-6700"[IS] OR "1873-1619"[IS] OR "1369-7021"[IS] OR "1873-4103"[IS] OR "0031-9333"[IS] OR "1522-1210"[IS] OR "1535-6108"[IS] OR "1878-3686"[IS] OR "0079-6425"[IS] OR "1873-2208"[IS] OR "1759-5045"[IS] OR "1759-5053"[IS] OR "1529-2908"[IS] OR "1529-2916"[IS] OR "0195-

	668X"[IS] OR "1522-9645"[IS] OR "1755-4330"[IS] OR "1755-4349"[IS] OR "0009-7322"[IS] OR "1524-4539"[IS] OR "2213-2600"[IS] OR "N/A"[IS] OR "N/A"[IS] OR "1553-877X"[IS] OR "2374-2437"[IS] OR "2374-2445"[IS] OR "1550-4131"[IS] OR "1932-7420"[IS] OR "0927-796X"[IS] OR "1879-212X"[IS] OR "1529-1006"[IS] OR "1539-6053"[IS] OR "1758-678X"[IS] OR "1758-6798"[IS] OR "1758-678X"[IS] OR "1758-6798"[IS] OR "0001-4842"[IS] OR "1520-4898"[IS] OR "1074-7613"[IS] OR "1097-4180"[IS] OR "1934-5909"[IS] OR "1875-9777"[IS] OR "0732-0582"[IS] OR "1545-3278"[IS] OR "1759-4758"[IS] OR "1759-4766"[IS] OR "1097-6256"[IS] OR "1546-1726"[IS] OR "0950-6608"[IS] OR "1743-2804"[IS] OR "2168-6106"[IS] OR "2168-6114"[IS] OR "1745-2473"[IS] OR "1745-2481"[IS] OR "0066-4308"[IS] OR "1545-2085"[IS] OR "0066-4308"[IS] OR "1545-2085"[IS] OR "1759-5061"[IS] OR "1759-507X"[IS] OR "2470-9476"[IS] OR "2470-9476"[IS] OR "0003-4819"[IS] OR "1539-3704"[IS] OR "0016-5085"[IS] OR "1528-0012"[IS] OR "0342-4642"[IS] OR "1432-1238"[IS] OR "0168-8278"[IS] OR "1600-0641"[IS] OR "1543-5008"[IS] OR "1545-2123"[IS] OR "0031-6997"[IS] OR "1521-0081"[IS] OR "0735-1097"[IS] OR "1558-3597"[IS] OR "1759-4790"[IS] OR "1759-4804"[IS] OR "2215-0374"[IS] OR "N/A"[IS] OR "2215-0374"[IS] OR "N/A"[IS] OR "2451-9294"[IS] OR "2451-9294"[IS] OR "0001-6322"[IS] OR "1432-0533"[IS] OR "0017-5749"[IS] OR "1468-3288"[IS] OR "0066-4278"[IS] OR "1545-1585"[IS] OR "1001-0602"[IS] OR "1748-7838"[IS] OR "0893-8512"[IS] OR "1098-6618"[IS] OR "0162-8828"[IS] OR "1939-3539"[IS] OR "1465-7392"[IS] OR "1476-4679"[IS] OR "1759-5002"[IS] OR "1759-5010"[IS] OR "0302-2838"[IS] OR "1873-7560"[IS] OR "0066-4189"[IS] OR "1545-4479"[IS] OR "0140-525X"[IS] OR "1469-1825"[IS] OR "0140-525X"[IS] OR "1469-1825"[IS] OR "1946-6234"[IS] OR "1946-6242"[IS] OR "2157-846X"[IS] OR "2157-846X"[IS] OR "0968-0004"[IS] OR "1362-4326"[IS] OR "1531-7331"[IS] OR "1545-4118"[IS] OR "8755-1209"[IS] OR "1944-9208"[IS] OR "0034-4885"[IS] OR "1361-6633"[IS] OR "0962-8924"[IS] OR "1879-3088"[IS] OR "1748-0132"[IS] OR "1878-044X"[IS] OR "0006-4971"[IS] OR "1528-0020"[IS] OR "1073-449X"[IS] OR "1535-4970"[IS] OR "0033-2909"[IS] OR "1939-1455"[IS] OR "0033-2909"[IS] OR "1939-1455"[IS] OR "2380-8195"[IS] OR "2380-8195"[IS] OR "1364-6613"[IS] OR "1879-307X"[IS] OR "1364-6613"[IS] OR "1879-307X"[IS] OR "2168-622X"[IS] OR "2168-6238"[IS] OR "2168-622X"[IS] OR "2168-6238"[IS] OR "2214-109X"[IS] OR "2214-109X"[IS] OR "2214-109X"[IS] OR "2214-109X"[IS] OR "0009-7330"[IS] OR "1524-4571"[IS] OR "N/A"[IS] OR "2198-3844"[IS] OR "1931-3128"[IS] OR "1934-6069"[IS] OR "1616-301X"[IS] OR "1616-3028"[IS] OR "1560-2745"[IS] OR "1878-9129"[IS] OR "1947-5454"[IS] OR "1947-5462"[IS] OR "2211-2855"[IS] OR "2211-3282"[IS] OR "0169-409X"[IS] OR "1872-8294"[IS] OR "0149-5992"[IS] OR "1935-5548"[IS] OR "1092-2172"[IS] OR "1098-5557"[IS] OR "0169-5347"[IS] OR "1872-8383"[IS] OR "1941-1405"[IS] OR "1941-0611"[IS] OR "0742-3098"[IS] OR "1600-079X"[IS] OR "0163-769X"[IS] OR "1945-7189"[IS] OR "0935-4956"[IS] OR "1432-0754"[IS] OR "0270-9139"[IS] OR "1527-3350"[IS] OR "0149-2195"[IS] OR "1545-861X"[IS] OR "0167-5729"[IS] OR "1879-274X"[IS] OR "0737-4038"[IS] OR "1537-1719"[IS] OR "2352-3018"[IS] OR "2352-3018"[IS] OR "0002-7863"[IS] OR "1520-5126"[IS] OR "2367-3648"[IS] OR "1614-4961"[IS] OR "1097-2765"[IS] OR "1097-4164"[IS] OR "1752-0894"[IS] OR "1752-0908"[IS] OR "1552-5260"[IS] OR "1552-5279"[IS] OR "0896-6273"[IS] OR "1097-4199"[IS] OR "2051-6347"[IS] OR "2051-6355"[IS] OR "2058-5276"[IS] OR "2058-5276"[IS] OR "0003-4967"[IS] OR "1468-2060"[IS] OR "0926-3373"[IS] OR "1873-3883"[IS] OR "0923-7534"[IS] OR "1569-8041"[IS] OR "0091-6749"[IS] OR "1097-6825"[IS] OR "1548-5943"[IS] OR "1548-5951"[IS] OR "1548-5943"[IS] OR "1548-5951"[IS] OR "1474-760X"[IS] OR "1474-760X"[IS] OR "1360-1385"[IS] OR "1878-4372"[IS] OR "2047-7538"[IS] OR "2047-7538"[IS] OR "1388-9842"[IS] OR "1879-0844"[IS] OR "1943-8206"[IS] OR "N/A"[IS] OR "1936-0851"[IS] OR "1936-086X"[IS] OR "1553-4006"[IS] OR "1553-4014"[IS] OR "0167-7799"[IS] OR "1879-3096"[IS] OR "0033-3190"[IS] OR "1423-0348"[IS] OR "0033-3190"[IS] OR "1423-0348"[IS] OR "0002-953X"[IS] OR "1535-7228"[IS] OR "0002-953X"[IS] OR "1535-7228"[IS] OR "0010-8545"[IS] OR "1873-3840"[IS] OR "2055-026X"[IS] OR "2055-0278"[IS] OR "1932-4529"[IS] OR "1941-0115"[IS] OR "2095-5138"[IS] OR "2053-714X"[IS] OR "1471-4906"[IS] OR "1471-4981"[IS] OR "1355-4786"[IS] OR "1460-2369"[IS] OR "N/A"[IS] OR "2468-1253"[IS] OR "2374-7943"[IS] OR "2374-7951"[IS] OR "0734-9750"[IS] OR "1873-1899"[IS] OR "2375-2548"[IS] OR "2375-2548"[IS] OR "1931-9401"[IS] OR "1931-9401"[IS] OR "0163-7827"[IS] OR "1873-2194"[IS] OR "1556-0864"[IS] OR "1556-1380"[IS] OR "2168-6149"[IS] OR "2168-6157"[IS] OR "0166-2236"[IS] OR "1878-108X"[IS] OR "1941-6520"[IS] OR "1941-6067"[IS] OR "0021-9738"[IS] OR "1558-8238"[IS] OR "1530-6984"[IS] OR "1530-6992"[IS] OR "1433-7851"[IS] OR "1521-3773"[IS] OR "1523-9829"[IS] OR "1545-4274"[IS] OR "2155-5435"[IS] OR "2155-5435"[IS] OR "2160-3308"[IS] OR "2160-3308"[IS] OR "1936-122X"[IS] OR "1936-1238"[IS] OR "1552-4450"[IS] OR "1552-4469"[IS] OR "1545-9993"[IS] OR "1545-9985"[IS] OR "0362-1642"[IS] OR "1545-4304"[IS] OR "0147-006X"[IS] OR "1545-4126"[IS] OR "2168-6203"[IS] OR "2168-6211"[IS] OR "2352-3026"[IS] OR "N/A"[IS] OR "0966-842X"[IS] OR "1878-4380"[IS] OR "1359-4184"[IS] OR "1476-5578"[IS] OR "N/A"[IS] OR "2041-1723"[IS] OR "0265-0568"[IS] OR "1460-4752"[IS] OR "2380-6583"[IS] OR "2380-6591"[IS] OR "0006-8950"[IS] OR "1460-2156"[IS] OR "0903-1936"[IS] OR "1399-3003"[IS] OR "0066-4170"[IS] OR "1545-4487"[IS] OR "0033-5533"[IS] OR "1531-4650"[IS] OR "1350-9462"[IS] OR "1873-1635"[IS] OR "1368-7646"[IS] OR "1532-2084"[IS] OR "2162-

	237X"[IS] OR "2162-2388"[IS] OR "1548-7660"[IS] OR "1548-7660"[IS] OR "0306-3674"[IS] OR "1473-0480"[IS] OR "2468-2667"[IS] OR "2468-2667"[IS] OR "2468-2667"[IS] OR "2468-2667"[IS] OR "0168-6445"[IS] OR "1574-6976"[IS] OR "0165-6147"[IS] OR "1873-3735"[IS] OR "0006-3223"[IS] OR "1873-2402"[IS] OR "1754-2189"[IS] OR "1750-2799"[IS] OR "0105-2896"[IS] OR "1600-065X"[IS] OR "0261-4189"[IS] OR "1460-2075"[IS] OR "0305-1048"[IS] OR "1362-4962"[IS] OR "1554-8627"[IS] OR "1554-8635"[IS] OR "1549-1277"[IS] OR "1549-1676"[IS] OR "1571-0645"[IS] OR "1873-1457"[IS] OR "1471-4914"[IS] OR "1471-499X"[IS] OR "1536-1284"[IS] OR "1558-0687"[IS] OR "N/A"[IS] OR "2001-3078"[IS] OR "1936-878X"[IS] OR "1876-7591"[IS] OR "2397-334X"[IS] OR "2397-334X"[IS] OR "1866-3508"[IS] OR "1866-3516"[IS] OR "1540-9295"[IS] OR "1540-9309"[IS] OR "0022-1007"[IS] OR "1540-9538"[IS] OR "1756-994X"[IS] OR "1756-994X"[IS] OR "1543-592X"[IS] OR "1545-2069"[IS] OR "1613-6810"[IS] OR "1613-6829"[IS] OR "1081-0706"[IS] OR "1530-8995"[IS] OR "1674-2052"[IS] OR "1752-9867"[IS] OR "0163-7525"[IS] OR "1545-2093"[IS] OR "0163-7525"[IS] OR "1545-2093"[IS] OR "0146-6410"[IS] OR "1873-2224"[IS] OR "2190-5991"[IS] OR "2190-6009"[IS] OR "2050-7488"[IS] OR "2050-7496"[IS] OR "1566-2535"[IS] OR "1872-6305"[IS] OR "0018-9219"[IS] OR "1558-2256"[IS] OR "N/A"[IS] OR "1476-4598"[IS] OR "2168-6254"[IS] OR "2168-6262"[IS] OR "0065-2156"[IS] OR "N/A"[IS] OR "0301-0082"[IS] OR "1873-5118"[IS] OR "0363-7425"[IS] OR "1930-3807"[IS] OR "0168-9525"[IS] OR "1362-4555"[IS] OR "1757-4676"[IS] OR "1757-4684"[IS] OR "2397-3374"[IS] OR "2397-3374"[IS] OR "2397-3374"[IS] OR "2397-3374"[IS] OR "1364-0321"[IS] OR "1879-0690"[IS] OR "2470-9468"[IS] OR "2470-9468"[IS] OR "1087-0792"[IS] OR "1532-2955"[IS] OR "2397-3366"[IS] OR "2397-3366"[IS] OR "1949-3053"[IS] OR "1949-3061"[IS] OR "2049-2618"[IS] OR "2049-2618"[IS] OR "0959-3780"[IS] OR "1872-9495"[IS] OR "0959-3780"[IS] OR "1872-9495"[IS] OR "1389-5567"[IS] OR "1873-2739"[IS] OR "1568-1637"[IS] OR "1872-9649"[IS] OR "2168-2267"[IS] OR "2168-2275"[IS] OR "0163-6804"[IS] OR "1558-1896"[IS] OR "1464-7931"[IS] OR "1469-185X"[IS] OR "0142-9612"[IS] OR "1878-5905"[IS] OR "1047-840X"[IS] OR "1532-7965"[IS] OR "1063-5157"[IS] OR "1076-836X"[IS] OR "0066-4227"[IS] OR "1545-3251"[IS] OR "0002-9270"[IS] OR "1572-0241"[IS] OR "0027-8874"[IS] OR "1460-2105"[IS] OR "0066-4286"[IS] OR "1545-2107"[IS] OR "0144-235X"[IS] OR "1366-591X"[IS] OR "0897-4756"[IS] OR "1520-5002"[IS] OR "0066-4219"[IS] OR "1545-326X"[IS] OR "1522-8517"[IS] OR "1523-5866"[IS])
Impact factor (2021) >5	("0007-9235"[IS] OR "1542-4863"[IS] OR "0140-6736"[IS] OR "1474-547X"[IS] OR "0028-4793"[IS] OR "1533-4406"[IS] OR "0098-7484"[IS] OR "1538-3598"[IS] OR "1471-0072"[IS] OR "1471-0080"[IS] OR "1474-1776"[IS] OR "1474-1784"[IS] OR "1474-1733"[IS] OR "1474-1741"[IS] OR "2213-2600"[IS] OR "N/A"[IS] OR "0959-535X"[IS] OR "1756-1833"[IS] OR "1078-8956"[IS] OR "1546-170X"[IS] OR "N/A"[IS] OR "2666-5247"[IS] OR "1723-8617"[IS] OR "2051-5545"[IS] OR "1740-1526"[IS] OR "1740-1534"[IS] OR "2215-0374"[IS] OR "N/A"[IS] OR "2058-8437"[IS] OR "2058-8437"[IS] OR "1759-5045"[IS] OR "1759-5053"[IS] OR "2468-2667"[IS] OR "2468-2667"[IS] OR "0009-2665"[IS] OR "1520-6890"[IS] OR "1473-3099"[IS] OR "1474-4457"[IS] OR "1474-175X"[IS] OR "1474-1768"[IS] OR "0028-0836"[IS] OR "1476-4687"[IS] OR "1087-0156"[IS] OR "1546-1696"[IS] OR "2058-7546"[IS] OR "2058-7546"[IS] OR "0092-8674"[IS] OR "1097-4172"[IS] OR "2056-676X"[IS] OR "2056-676X"[IS] OR "1759-4774"[IS] OR "1759-4782"[IS] OR "0036-8075"[IS] OR "1095-9203"[IS] OR "0306-0012"[IS] OR "1460-4744"[IS] OR "1474-4422"[IS] OR "1474-4465"[IS] OR "1471-0056"[IS] OR "1471-0064"[IS] OR "1529-1006"[IS] OR "1539-6053"[IS] OR "1470-2045"[IS] OR "1474-5488"[IS] OR "0923-7534"[IS] OR "1569-8041"[IS] OR "0003-4819"[IS] OR "1539-3704"[IS] OR "0732-183X"[IS] OR "1527-7755"[IS] OR "0034-6861"[IS] OR "1539-0756"[IS] OR "0893-8512"[IS] OR "1098-6618"[IS] OR "1759-5002"[IS] OR "1759-5010"[IS] OR "0079-6425"[IS] OR "1873-2208"[IS] OR "1548-7091"[IS] OR "1548-7105"[IS] OR "1476-1122"[IS] OR "1476-4660"[IS] OR "1759-5029"[IS] OR "1759-5037"[IS] OR "0031-9333"[IS] OR "1522-1210"[IS] OR "1001-0602"[IS] OR "1748-7838"[IS] OR "2542-4351"[IS] OR "2542-4351"[IS] OR "N/A"[IS] OR "2468-1253"[IS] OR "2213-8587"[IS] OR "2213-8595"[IS] OR "1759-4758"[IS] OR "1759-4766"[IS] OR "2168-6106"[IS] OR "2168-6114"[IS] OR "1074-7613"[IS] OR "1097-4180"[IS] OR "2367-3613"[IS] OR "1433-8351"[IS] OR "1759-5061"[IS] OR "1759-507X"[IS] OR "0342-4642"[IS] OR "1432-1238"[IS] OR "N/A"[IS] OR "1476-4598"[IS] OR "1061-4036"[IS] OR "1546-1718"[IS] OR "2520-1158"[IS] OR "2520-1158"[IS] OR "1748-3387"[IS] OR "1748-3395"[IS] OR "0009-7322"[IS] OR "1524-4539"[IS] OR "1749-4885"[IS] OR "1749-4893"[IS] OR "1754-5692"[IS] OR "1754-5706"[IS] OR "1195-1982"[IS] OR "1708-8305"[IS] OR "2214-109X"[IS] OR "2214-109X"[IS] OR "1471-003X"[IS] OR "1471-0048"[IS] OR "0163-4453"[IS] OR "1532-2742"[IS] OR "1535-6108"[IS] OR "1878-3686"[IS] OR "2159-8274"[IS] OR "2159-8290"[IS] OR "2095-9907"[IS] OR "2059-3635"[IS] OR "N/A"[IS] OR "2056-5968"[IS] OR "2352-4642"[IS] OR "2352-4642"[IS] OR "1077-291X"[IS] OR "N/A"[IS] OR "0066-4146"[IS] OR "1545-4282"[IS] OR "N/A"[IS] OR "2662-138X"[IS] OR "N/A"[IS] OR "2589-7500"[IS] OR "1057-5987"[IS] OR "1545-8601"[IS] OR "N/A"[IS] OR "2522-5820"[IS] OR "0195-668X"[IS] OR "1522-9645"[IS] OR "0935-4956"[IS] OR "1432-0754"[IS] OR "2665-9913"[IS] OR "2665-9913"[IS] OR "0360-1285"[IS] OR "1873-216X"[IS] OR "0149-2195"[IS] OR "1545-861X"[IS] OR "2095-7467"[IS] OR "2054-9369"[IS] OR "N/A"[IS] OR "2397-3358"[IS] OR "0016-5085"[IS] OR "1528-0012"[IS] OR "N/A"[IS] OR "1553-877X"[IS] OR "0903-1936"[IS] OR "1399-3003"[IS]

	OR "0927-796X"[IS] OR "1879-212X"[IS] OR "2520-1131"[IS] OR "2520-1131"[IS] OR "2374-2437"[IS] OR "2374-2445"[IS] OR "2520-8489"[IS] OR "2520-8136"[IS] OR "0732-0582"[IS] OR "1545-3278"[IS] OR "1553-4006"[IS] OR "1553-4014"[IS] OR "1759-4790"[IS] OR "1759-4804"[IS] OR "0935-9648"[IS] OR "1521-4095"[IS] OR "0017-5749"[IS] OR "1468-3288"[IS] OR "1550-4131"[IS] OR "1932-7420"[IS] OR "1931-3128"[IS] OR "1934-6069"[IS] OR "0079-6700"[IS] OR "1873-1619"[IS] OR "1529-2908"[IS] OR "1529-2916"[IS] OR "2058-5276"[IS] OR "2058-5276"[IS] OR "2470-9468"[IS] OR "2470-9468"[IS] OR "1073-449X"[IS] OR "1535-4970"[IS] OR "0370-1573"[IS] OR "1873-6270"[IS] OR "2380-6583"[IS] OR "2380-6591"[IS] OR "2352-3026"[IS] OR "N/A"[IS] OR "0168-8278"[IS] OR "1600-0641"[IS] OR "2168-6149"[IS] OR "2168-6157"[IS] OR "1614-6832"[IS] OR "1614-6840"[IS] OR "2157-846X"[IS] OR "2157-846X"[IS] OR "0033-8419"[IS] OR "N/A"[IS] OR "1545-8636"[IS] OR "1545-8636"[IS] OR "1097-6256"[IS] OR "1546-1726"[IS] OR "N/A"[IS] OR "2542-5196"[IS] OR "2367-3648"[IS] OR "1614-4961"[IS] OR "1758-678X"[IS] OR "1758-6798"[IS] OR "1543-5008"[IS] OR "1545-2123"[IS] OR "1465-7392"[IS] OR "1476-4679"[IS] OR "0003-4967"[IS] OR "1468-2060"[IS] OR "0066-4308"[IS] OR "1545-2085"[IS] OR "2470-9476"[IS] OR "2470-9476"[IS] OR "0066-4154"[IS] OR "1545-4509"[IS] OR "0735-1097"[IS] OR "1558-3597"[IS] OR "2398-9629"[IS] OR "2398-9629"[IS] OR "2043-8206"[IS] OR "2043-8214"[IS] OR "1369-7021"[IS] OR "1873-4103"[IS] OR "2168-6203"[IS] OR "2168-6211"[IS] OR "2168-622X"[IS] OR "2168-6238"[IS] OR "N/A"[IS] OR "2522-5839"[IS] OR "2451-9294"[IS] OR "2451-9294"[IS] OR "0166-0616"[IS] OR "1872-9797"[IS] OR "0033-3190"[IS] OR "1423-0348"[IS] OR "0006-4971"[IS] OR "1528-0020"[IS] OR "0066-4189"[IS] OR "1545-4479"[IS] OR "1934-5909"[IS] OR "1875-9777"[IS] OR "0163-769X"[IS] OR "1945-7189"[IS] OR "8755-1209"[IS] OR "1944-9208"[IS] OR "1560-2745"[IS] OR "1878-9129"[IS] OR "1674-2818"[IS] OR "2049-3169"[IS] OR "0010-8545"[IS] OR "1873-3840"[IS] OR "N/A"[IS] OR "2567-3165"[IS] OR "1943-8206"[IS] OR "N/A"[IS] OR "1364-6613"[IS] OR "1879-307X"[IS] OR "0001-4842"[IS] OR "1520-4898"[IS] OR "0926-3373"[IS] OR "1873-3883"[IS] OR "0162-8828"[IS] OR "1939-3539"[IS] OR "1755-4330"[IS] OR "1755-4349"[IS] OR "0302-2838"[IS] OR "1873-7560"[IS] OR "2397-3374"[IS] OR "2397-3374"[IS] OR "2380-8195"[IS] OR "2380-8195"[IS] OR "1947-5454"[IS] OR "1947-5462"[IS] OR "0001-8732"[IS] OR "1460-6976"[IS] OR "2311-6706"[IS] OR "2150-5551"[IS] OR "0009-7330"[IS] OR "1524-4571"[IS] OR "2095-5138"[IS] OR "2053-714X"[IS] OR "N/A"[IS] OR "2662-1347"[IS] OR "N/A"[IS] OR "1756-8722"[IS] OR "0033-2909"[IS] OR "1939-1455"[IS] OR "1368-7646"[IS] OR "1532-2084"[IS] OR "0066-4170"[IS] OR "1545-4487"[IS] OR "N/A"[IS] OR "2589-5974"[IS] OR "0066-4278"[IS] OR "1545-1585"[IS] OR "1548-5943"[IS] OR "1548-5951"[IS] OR "1672-7681"[IS] OR "2042-0226"[IS] OR "1360-1385"[IS] OR "1878-4372"[IS] OR "1674-2052"[IS] OR "1752-9867"[IS] OR "0167-7799"[IS] OR "1879-3096"[IS] OR "0163-7525"[IS] OR "1545-2093"[IS] OR "N/A"[IS] OR "2637-9368"[IS] OR "1752-0894"[IS] OR "1752-0908"[IS] OR "0140-525X"[IS] OR "1469-1825"[IS] OR "1025-496X"[IS] OR "1560-7917"[IS] OR "0962-8924"[IS] OR "1879-3088"[IS] OR "1058-4838"[IS] OR "1537-6591"[IS] OR "2405-8297"[IS] OR "2405-8297"[IS] OR "0146-6615"[IS] OR "1096-9071"[IS] OR "0169-5347"[IS] OR "1872-8383"[IS] OR "2095-9273"[IS] OR "2095-9281"[IS] OR "1477-8939"[IS] OR "1873-0442"[IS] OR "N/A"[IS] OR "2662-1355"[IS] OR "2047-7538"[IS] OR "2047-7538"[IS] OR "1556-0864"[IS] OR "1556-1380"[IS] OR "2590-2393"[IS] OR "2590-2385"[IS] OR "1616-301X"[IS] OR "1616-3028"[IS] OR "N/A"[IS] OR "2522-5812"[IS] OR "N/A"[IS] OR "2662-1991"[IS] OR "1936-122X"[IS] OR "1936-1238"[IS] OR "1471-4906"[IS] OR "1471-4981"[IS] OR "1350-9462"[IS] OR "1873-1635"[IS] OR "1745-2473"[IS] OR "1745-2481"[IS] OR "N/A"[IS] OR "2222-1751"[IS] OR "1931-9401"[IS] OR "1931-9401"[IS] OR "0021-9738"[IS] OR "1558-8238"[IS] OR "1364-8535"[IS] OR "1466-609X"[IS] OR "1097-2765"[IS] OR "1097-4164"[IS] OR "1946-6234"[IS] OR "1946-6242"[IS] OR "0002-953X"[IS] OR "1535-7228"[IS] OR "1941-6520"[IS] OR "1941-6067"[IS] OR "0889-1591"[IS] OR "1090-2139"[IS] OR "2405-8025"[IS] OR "2405-8033"[IS] OR "0305-1048"[IS] OR "1362-4962"[IS] OR "2168-2267"[IS] OR "2168-2275"[IS] OR "2397-334X"[IS] OR "2397-334X"[IS] OR "2211-2855"[IS] OR "2211-3282"[IS] OR "0033-5533"[IS] OR "1531-4650"[IS] OR "0085-2538"[IS] OR "1523-1755"[IS] OR "1748-0132"[IS] OR "1878-044X"[IS] OR "0268-4012"[IS] OR "1873-4707"[IS] OR "0031-6997"[IS] OR "1521-0081"[IS] OR "N/A"[IS] OR "1750-1326"[IS] OR "2374-7943"[IS] OR "2374-7951"[IS] OR "0896-6273"[IS] OR "1097-4199"[IS] OR "0306-3674"[IS] OR "1473-0480"[IS] OR "1545-9993"[IS] OR "1545-9985"[IS] OR "0966-842X"[IS] OR "1878-4380"[IS] OR "1936-0851"[IS] OR "1936-086X"[IS] OR "0141-0768"[IS] OR "1758-1095"[IS] OR "1543-5938"[IS] OR "1545-2050"[IS] OR "1474-760X"[IS] OR "1474-760X"[IS] OR "0169-409X"[IS] OR "1872-8294"[IS] OR "0034-4885"[IS] OR "1361-6633"[IS] OR "0163-8998"[IS] OR "1545-4134"[IS] OR "N/A"[IS] OR "2041-1723"[IS] OR "0734-9750"[IS] OR "1873-1899"[IS] OR "1359-6101"[IS] OR "1879-0305"[IS] OR "0165-6147"[IS] OR "1873-3735"[IS] OR "0022-1007"[IS] OR "1540-9538"[IS] OR "1566-2535"[IS] OR "1872-6305"[IS] OR "N/A"[IS] OR "2198-3844"[IS] OR "0065-2156"[IS] OR "N/A"[IS] OR "1568-9972"[IS] OR "1873-0183"[IS] OR "2055-026X"[IS] OR "2055-0278"[IS] OR "1388-9842"[IS] OR "1879-0844"[IS] OR "N/A"[IS] OR "2001-3078"[IS] OR "0270-9139"[IS] OR "1527-3350"[IS] OR "1355-4786"[IS] OR "1460-2369"[IS] OR "1389-5567"[IS] OR "1873-2739"[IS] OR "0149-5992"[IS] OR "1935-5548"[IS] OR "N/A"[IS] OR "2589-5370"[IS] OR "1754-2189"[IS] OR "1750-2799"[IS] OR "1044-579X"[IS] OR "1096-3650"[IS] OR "2666-3791"[IS] OR "2666-3791"[IS] OR

	"0166-2236"[IS] OR "1878-108X"[IS] OR "N/A"[IS] OR "2452-199X"[IS] OR "0820-3946"[IS] OR "1488-2329"[IS] OR "2049-2618"[IS] OR "2049-2618"[IS] OR "1433-7851"[IS] OR "1521-3773"[IS] OR "1364-0321"[IS] OR "1879-0690"[IS] OR "1385-8947"[IS] OR "1873-3212"[IS] OR "2168-6254"[IS] OR "2168-6262"[IS] OR "1552-5260"[IS] OR "1552-5279"[IS] OR "1941-1405"[IS] OR "1941-0611"[IS] OR "2077-7000"[IS] OR "2077-7000"[IS] OR "1089-778X"[IS] OR "1941-0026"[IS] OR "0146-2806"[IS] OR "1535-6280"[IS] OR "0362-1642"[IS] OR "1545-4304"[IS] OR "1759-4812"[IS] OR "1759-4820"[IS] OR "0002-7863"[IS] OR "1520-5126"[IS] OR "0003-066X"[IS] OR "1935-990X"[IS] OR "0098-2997"[IS] OR "1872-9452"[IS] OR "0084-6597"[IS] OR "1545-4495"[IS] OR "0066-4227"[IS] OR "1545-3251"[IS] OR "1552-4450"[IS] OR "1552-4469"[IS] OR "1088-8683"[IS] OR "1532-7957"[IS] OR "1080-6040"[IS] OR "1080-6059"[IS] OR "2352-3018"[IS] OR "2352-3018"[IS] OR "1936-878X"[IS] OR "1876-7591"[IS] OR "0066-4219"[IS] OR "1545-326X"[IS] OR "1538-7933"[IS] OR "1538-7836"[IS] OR "0924-2244"[IS] OR "1879-3053"[IS] OR "0001-6322"[IS] OR "1432-0533"[IS] OR "1226-4601"[IS] OR "2055-7124"[IS] OR "2515-2459"[IS] OR "2515-2467"[IS] OR "1541-4337"[IS] OR "1541-4337"[IS] OR "0950-6608"[IS] OR "1743-2804"[IS] OR "2051-6347"[IS] OR "2051-6355"[IS] OR "2397-3366"[IS] OR "2397-3366"[IS] OR "0962-4929"[IS] OR "1474-0508"[IS] OR "0147-006X"[IS] OR "1545-4126"[IS] OR "0190-9622"[IS] OR "1097-6787"[IS] OR "2326-5191"[IS] OR "2326-5205"[IS] OR "0924-8579"[IS] OR "1872-7913"[IS] OR "2366-9608"[IS] OR "2366-9608"[IS] OR "0022-2429"[IS] OR "1547-7185"[IS] OR "2398-6352"[IS] OR "2398-6352"[IS] OR "1674-800X"[IS] OR "1674-8018"[IS] OR "0142-9612"[IS] OR "1878-5905"[IS] OR "N/A"[IS] OR "2523-3548"[IS] OR "1471-4914"[IS] OR "1471-499X"[IS] OR "1756-994X"[IS] OR "1756-994X"[IS] OR "0006-8950"[IS] OR "1460-2156"[IS] OR "1053-5888"[IS] OR "1558-0792"[IS] OR "0001-8686"[IS] OR "1873-3727"[IS] OR "0168-6445"[IS] OR "1574-6976"[IS] OR "1613-6810"[IS] OR "1613-6829"[IS] OR "0265-0568"[IS] OR "1460-4752"[IS] OR "1046-6673"[IS] OR "1533-3450"[IS] OR "2375-2548"[IS] OR "2375-2548"[IS] OR "2590-3330"[IS] OR "2590-3322"[IS] OR "0013-0095"[IS] OR "1944-8287"[IS] OR "0018-9219"[IS] OR "1558-2256"[IS] OR "0165-9936"[IS] OR "1879-3142"[IS] OR "0092-0703"[IS] OR "1552-7824"[IS] OR "2211-3835"[IS] OR "2211-3843"[IS] OR "1941-1413"[IS] OR "1941-1421"[IS] OR "0105-4538"[IS] OR "1398-9995"[IS] OR "0963-8687"[IS] OR "1873-1198"[IS] OR "1526-5161"[IS] OR "1536-0075"[IS] OR "0163-7827"[IS] OR "1873-2194"[IS] OR "2369-2960"[IS] OR "2369-2960"[IS] OR "2213-6657"[IS] OR "2213-6665"[IS] OR "1558-3724"[IS] OR "1558-3716"[IS] OR "2050-7488"[IS] OR "2050-7496"[IS] OR "0896-8411"[IS] OR "1095-9157"[IS] OR "1386-6532"[IS] OR "1873-5967"[IS] OR "2160-3308"[IS] OR "2160-3308"[IS] OR "N/A"[IS] OR "2057-3995"[IS] OR "1464-7931"[IS] OR "1469-185X"[IS] OR "1543-592X"[IS] OR "1545-2069"[IS] OR "0360-0300"[IS] OR "1557-7341"[IS] OR "0091-6749"[IS] OR "1097-6825"[IS] OR "1569-1705"[IS] OR "1572-9826"[IS] OR "0161-6420"[IS] OR "1549-4713"[IS] OR "0968-0004"[IS] OR "1362-4326"[IS] OR "2327-056X"[IS] OR "2327-0578"[IS] OR "1757-4676"[IS] OR "1757-4684"[IS] OR "2162-237X"[IS] OR "2162-2388"[IS] OR "0304-3894"[IS] OR "1873-3336"[IS] OR "0001-0782"[IS] OR "1557-7317"[IS] OR "0004-3702"[IS] OR "1872-7921"[IS] OR "1460-2725"[IS] OR "1460-2393"[IS] OR "2095-1779"[IS] OR "2214-0883"[IS] OR "0261-4189"[IS] OR "1460-2075"[IS] OR "1467-5463"[IS] OR "1477-4054"[IS] OR "1949-3045"[IS] OR "1949-3045"[IS] OR "1531-7331"[IS] OR "1545-4118"[IS] OR "0026-0495"[IS] OR "1532-8600"[IS] OR "2473-2397"[IS] OR "2168-6831"[IS] OR "1876-2018"[IS] OR "1876-2026"[IS] OR "0363-7425"[IS] OR "1930-3807"[IS] OR "0034-4257"[IS] OR "1879-0704"[IS] OR "0042-9686"[IS] OR "1564-0604"[IS] OR "1361-8415"[IS] OR "1361-8423"[IS] OR "0066-4197"[IS] OR "1545-2948"[IS] OR "1078-0432"[IS] OR "1557-3265"[IS] OR "0003-4932"[IS] OR "1528-1140"[IS] OR "1540-9295"[IS] OR "1540-9309"[IS] OR "0921-3449"[IS] OR "1879-0658"[IS] OR "0887-6185"[IS] OR "1873-7897"[IS] OR "2155-5435"[IS] OR "2155-5435"[IS] OR "2590-1168"[IS] OR "2590-1168"[IS] OR "0022-3050"[IS] OR "1468-330X"[IS] OR "2214-1588"[IS] OR "2214-1588"[IS] OR "1610-3653"[IS] OR "1610-3661"[IS] OR "1556-6072"[IS] OR "1556-6080"[IS] OR "0305-7372"[IS] OR "1532-1967"[IS] OR "0161-4940"[IS] OR "1520-5703"[IS] OR "2095-4956"[IS] OR "2095-4956"[IS] OR "N/A"[IS] OR "2577-5421"[IS] OR "1542-3565"[IS] OR "1542-7714"[IS] OR "1053-2498"[IS] OR "1557-3117"[IS] OR "0034-6543"[IS] OR "1935-1046"[IS] OR "1362-0347"[IS] OR "1468-960X"[IS] OR "0149-2063"[IS] OR "1557-1211"[IS] OR "N/A"[IS] OR "2575-0356"[IS] OR "1359-4184"[IS] OR "1476-5578"[IS] OR "0167-5729"[IS] OR "1879-274X"[IS] OR "1534-5807"[IS] OR "1878-1551"[IS] OR "1743-9191"[IS] OR "1743-9159"[IS] OR "0163-7258"[IS] OR "1879-016X"[IS] OR "0043-1354"[IS] OR "1879-2448"[IS] OR "1554-8627"[IS] OR "1554-8635"[IS] OR "0920-5691"[IS] OR "1573-1405"[IS] OR "2588-8420"[IS] OR "2588-8420"[IS] OR "2095-4700"[IS] OR "2095-6231"[IS] OR "2574-3805"[IS] OR "2574-3805"[IS] OR "0160-4120"[IS] OR "1873-6750"[IS] OR "2165-8102"[IS] OR "2165-8110"[IS] OR "2472-3428"[IS] OR "2472-3428"[IS] OR "0008-5472"[IS] OR "1538-7445"[IS] OR "1198-743X"[IS] OR "1469-0691"[IS] OR "1554-0669"[IS] OR "1554-0677"[IS] OR "0361-8609"[IS] OR "1096-8652"[IS] OR "1467-7644"[IS] OR "1467-7652"[IS] OR "1354-1013"[IS] OR "1365-2486"[IS] OR "0883-9026"[IS] OR "1873-2003"[IS] OR "0890-8567"[IS] OR "1527-5418"[IS] OR "0008-6363"[IS] OR "1755-3245"[IS] OR "0733-8716"[IS] OR "1558-0008"[IS] OR "2095-2546"[IS] OR "2213-2961"[IS] OR "0954-6820"[IS] OR "1365-2796"[IS] OR "1744-4292"[IS] OR "1744-4292"[IS] OR "1092-2172"[IS] OR "1098-5557"[IS] OR "1522-

	8517"[IS] OR "1523-5866"[IS] OR "2524-7921"[IS] OR "2524-793X"[IS] OR "0253-9837"[IS] OR "1872-2067"[IS] OR "1525-0016"[IS] OR "1525-0024"[IS] OR "0022-0515"[IS] OR "2328-8175"[IS] OR "0003-2409"[IS] OR "1365-2044"[IS] OR "0890-9369"[IS] OR "1549-5477"[IS] OR "0887-6924"[IS] OR "1476-5551"[IS] OR "0261-5177"[IS] OR "1879-3193"[IS] OR "1359-0286"[IS] OR "1879-0348"[IS] OR "0160-7383"[IS] OR "1873-7722"[IS] OR "2095-8099"[IS] OR "2096-0026"[IS] OR "2090-1232"[IS] OR "2090-1224"[IS] OR "0006-3223"[IS] OR "1873-2402"[IS] OR "0002-0729"[IS] OR "1468-2834"[IS] OR "2096-2797"[IS] OR "2468-0257"[IS] OR "0027-8424"[IS] OR "1091-6490"[IS] OR "1536-1284"[IS] OR "1558-0687"[IS] OR "0025-729X"[IS] OR "1326-5377"[IS] OR "1021-7770"[IS] OR "1423-0127"[IS] OR "1540-1405"[IS] OR "1540-1413"[IS] OR "N/A"[IS] OR "1756-9966"[IS] OR "2327-0608"[IS] OR "2327-0616"[IS] OR "0956-5663"[IS] OR "1873-4235"[IS] OR "2213-1779"[IS] OR "2213-1787"[IS] OR "0001-8392"[IS] OR "1930-3815"[IS] OR "N/A"[IS] OR "2051-1426"[IS] OR "0003-1224"[IS] OR "1939-8271"[IS] OR "0393-2990"[IS] OR "1573-7284"[IS] OR "2235-1795"[IS] OR "1664-5553"[IS] OR "0146-6410"[IS] OR "1873-2224"[IS] OR "0300-8428"[IS] OR "1435-1803"[IS] OR "1936-1327"[IS] OR "1936-1335"[IS] OR "0198-6325"[IS] OR "1098-1128"[IS] OR "0302-282X"[IS] OR "1423-0224"[IS] OR "1530-6984"[IS] OR "1530-6992"[IS] OR "N/A"[IS] OR "2057-3960"[IS] OR "1063-6706"[IS] OR "1941-0034"[IS] OR "0906-6713"[IS] OR "1600-0757"[IS] OR "N/A"[IS] OR "2567-3173"[IS] OR "2059-7037"[IS] OR "2059-7037"[IS] OR "0009-9147"[IS] OR "1530-8561"[IS] OR "1226-3613"[IS] OR "2092-6413"[IS] OR "1323-1316"[IS] OR "1440-1819"[IS] OR "0017-8012"[IS] OR "0017-8012"[IS] OR "1040-4651"[IS] OR "1532-298X"[IS] OR "0001-8791"[IS] OR "1095-9084"[IS] OR "0742-3098"[IS] OR "1600-079X"[IS] OR "1094-2939"[IS] OR "1545-1577"[IS] OR "1201-9712"[IS] OR "1878-3511"[IS] OR "1350-9047"[IS] OR "1476-5403"[IS] OR "2190-5991"[IS] OR "2190-6009"[IS] OR "0002-9270"[IS] OR "1572-0241"[IS] OR "0012-8252"[IS] OR "1872-6828"[IS] OR "2326-6066"[IS] OR "2326-6074"[IS] OR "N/A"[IS] OR "2397-4621"[IS] OR "1469-493X"[IS] OR "1361-6137"[IS] OR "0008-8846"[IS] OR "1873-3948"[IS] OR "2573-5144"[IS] OR "2573-5144"[IS] OR "0112-1642"[IS] OR "1179-2035"[IS] OR "1081-0706"[IS] OR "1530-8995"[IS] OR "0960-8524"[IS] OR "1873-2976"[IS] OR "1548-9213"[IS] OR "1548-9221"[IS] OR "0168-9525"[IS] OR "1362-4555"[IS] OR "2168-6068"[IS] OR "2168-6084"[IS] OR "0027-8874"[IS] OR "1460-2105"[IS] OR "1866-3508"[IS] OR "1866-3516"[IS] OR "2213-9567"[IS] OR "2213-9567"[IS] OR "2522-0128"[IS] OR "2522-0136"[IS] OR "0021-9010"[IS] OR "1939-1854"[IS] OR "0028-3878"[IS] OR "1526-632X"[IS] OR "1568-1637"[IS] OR "1872-9649"[IS] OR "0924-2716"[IS] OR "1872-8235"[IS] OR "1863-2297"[IS] OR "1863-2300"[IS] OR "1064-3389"[IS] OR "1547-6537"[IS] OR "0007-0912"[IS] OR "1471-6771"[IS] OR "2467-964X"[IS] OR "2452-414X"[IS] OR "2055-6756"[IS] OR "2055-6764"[IS] OR "0008-1256"[IS] OR "2162-8564"[IS] OR "0095-1137"[IS] OR "1098-660X"[IS] OR "0031-5850"[IS] OR "N/A"[IS] OR "1551-3203"[IS] OR "1941-0050"[IS] OR "2214-8604"[IS] OR "2214-7810"[IS] OR "1745-6916"[IS] OR "1745-6924"[IS] OR "1549-1277"[IS] OR "1549-1676"[IS] OR "1838-7640"[IS] OR "1838-7640"[IS] OR "2161-8313"[IS] OR "2156-5376"[IS] OR "0006-3568"[IS] OR "1525-3244"[IS] OR "0090-0036"[IS] OR "1541-0048"[IS] OR "2328-8930"[IS] OR "N/A"[IS] OR "1557-1874"[IS] OR "1557-1882"[IS] OR "2226-4108"[IS] OR "2227-8508"[IS] OR "0196-8904"[IS] OR "1879-2227"[IS] OR "0268-005X"[IS] OR "1873-7137"[IS] OR "1759-0876"[IS] OR "1759-0884"[IS] OR "0002-8282"[IS] OR "1944-7981"[IS] OR "2168-2216"[IS] OR "2168-2232"[IS] OR "0168-3659"[IS] OR "1873-4995"[IS] OR "2524-7972"[IS] OR "2524-7867"[IS] OR "0306-2619"[IS] OR "1872-9118"[IS] OR "0012-6667"[IS] OR "1179-1950"[IS] OR "0304-419X"[IS] OR "1879-2561"[IS] OR "1087-0792"[IS] OR "1532-2955"[IS] OR "0272-7358"[IS] OR "1873-7811"[IS] OR "0166-4972"[IS] OR "1879-2383"[IS] OR "2332-7812"[IS] OR "2332-7812"[IS] OR "0013-936X"[IS] OR "1520-5851"[IS] OR "1461-6688"[IS] OR "1470-1340"[IS] OR "0008-3976"[IS] OR "1744-7976"[IS] OR "0003-6900"[IS] OR "2379-0407"[IS] OR "N/A"[IS] OR "2688-4062"[IS] OR "1523-9829"[IS] OR "1545-4274"[IS] OR "1359-8368"[IS] OR "1879-1069"[IS] OR "1094-9968"[IS] OR "1520-6653"[IS] OR "0008-6223"[IS] OR "1873-3891"[IS] OR "0033-0620"[IS] OR "1873-1740"[IS] OR "0364-5134"[IS] OR "1531-8249"[IS] OR "1461-023X"[IS] OR "1461-0248"[IS] OR "1359-8546"[IS] OR "1758-6852"[IS] OR "0925-5273"[IS] OR "1873-7579"[IS] OR "0166-3615"[IS] OR "1872-6194"[IS] OR "0165-1781"[IS] OR "1872-7123"[IS] OR "2530-7614"[IS] OR "2444-569X"[IS] OR "1751-7362"[IS] OR "1751-7370"[IS] OR "0011-9164"[IS] OR "1873-4464"[IS] OR "1040-8398"[IS] OR "1549-7852"[IS] OR "2352-3964"[IS] OR "2352-3964"[IS] OR "0022-4359"[IS] OR "1873-3271"[IS] OR "0360-1315"[IS] OR "1873-782X"[IS] OR "1040-8436"[IS] OR "1547-6561"[IS] OR "2055-6837"[IS] OR "2055-6845"[IS] OR "N/A"[IS] OR "2639-4979"[IS] OR "0959-3780"[IS] OR "1872-9495"[IS] OR "1741-7015"[IS] OR "1741-7015"[IS] OR "0007-1323"[IS] OR "1365-2168"[IS] OR "0007-0963"[IS] OR "1365-2133"[IS] OR "0025-6196"[IS] OR "1942-5546"[IS] OR "0047-2506"[IS] OR "1478-6990"[IS] OR "2192-2640"[IS] OR "2192-2659"[IS] OR "2405-4712"[IS] OR "2405-4720"[IS] OR "0161-5505"[IS] OR "1535-5667"[IS] OR "1936-8798"[IS] OR "1876-7605"[IS] OR "0959-6526"[IS] OR "1879-1786"[IS] OR "0272-6386"[IS] OR "1523-6838"[IS] OR "1077-5552"[IS] OR "1077-5552"[IS] OR "1758-5082"[IS] OR "1758-5090"[IS] OR "0390-6078"[IS] OR "N/A"[IS] OR "1052-9276"[IS] OR "1099-1654"[IS] OR "0002-9297"[IS] OR "1537-6605"[IS] OR "1057-7149"[IS] OR "1941-0042"[IS] OR "0278-0062"[IS] OR "1558-254X"[IS] OR "N/A"[IS] OR "2639-5274"[IS] OR "0091-6765"[IS] OR "1552-9924"[IS]

	OR "1463-9262"[IS] OR "1463-9270"[IS] OR "2213-2198"[IS] OR "2213-2201"[IS] OR "2542-5293"[IS] OR "2542-5293"[IS] OR "1939-1374"[IS] OR "1939-1374"[IS] OR "1474-9718"[IS] OR "1474-9726"[IS] OR "0105-2896"[IS] OR "1600-065X"[IS] OR "0001-4273"[IS] OR "1948-0989"[IS] OR "2198-6436"[IS] OR "2198-6436"[IS] OR "0969-6989"[IS] OR "1873-1384"[IS] OR "0148-2963"[IS] OR "1873-7978"[IS] OR "1745-2759"[IS] OR "1745-2767"[IS] OR "1863-8880"[IS] OR "1863-8899"[IS] OR "1082-989X"[IS] OR "1939-1463"[IS] OR "0960-9822"[IS] OR "1879-0445"[IS] OR "0301-0082"[IS] OR "1873-5118"[IS] OR "0040-1625"[IS] OR "1873-5509"[IS] OR "1467-7881"[IS] OR "1467-789X"[IS] OR "0066-4286"[IS] OR "1545-2107"[IS] OR "N/A"[IS] OR "2196-1115"[IS] OR "1080-0549"[IS] OR "1559-0267"[IS] OR "0964-4733"[IS] OR "1099-0836"[IS] OR "2213-2317"[IS] OR "2213-2317"[IS] OR "0363-9762"[IS] OR "1536-0229"[IS] OR "1884-4049"[IS] OR "1884-4057"[IS] OR "2590-0064"[IS] OR "2590-0064"[IS] OR "N/A"[IS] OR "2056-6387"[IS] OR "0048-9697"[IS] OR "1879-1026"[IS] OR "1449-2288"[IS] OR "1449-2288"[IS] OR "0144-8617"[IS] OR "1879-1344"[IS] OR "1836-9553"[IS] OR "1836-9561"[IS] OR "1367-5788"[IS] OR "1872-9088"[IS] OR "2210-6707"[IS] OR "2210-6715"[IS] OR "0002-9378"[IS] OR "1097-6868"[IS] OR "N/A"[IS] OR "2380-6761"[IS] OR "2214-9937"[IS] OR "2214-9937"[IS] OR "1044-5323"[IS] OR "1096-3618"[IS] OR "0007-1250"[IS] OR "1472-1465"[IS] OR "1053-4822"[IS] OR "1873-7889"[IS] OR "0969-6970"[IS] OR "1573-7209"[IS] OR "2334-2536"[IS] OR "2334-2536"[IS] OR "1742-7061"[IS] OR "1878-7568"[IS] OR "0268-960X"[IS] OR "1532-1681"[IS] OR "1753-5123"[IS] OR "1753-5131"[IS] OR "1555-9041"[IS] OR "1555-905X"[IS] OR "0033-2917"[IS] OR "1469-8978"[IS] OR "1043-2760"[IS] OR "1879-3061"[IS] OR "1478-0887"[IS] OR "1478-0895"[IS] OR "0007-6813"[IS] OR "1873-6068"[IS] OR "0376-7388"[IS] OR "1873-3123"[IS] OR "1471-4922"[IS] OR "1471-5007"[IS] OR "0926-5805"[IS] OR "1872-7891"[IS] OR "N/A"[IS] OR "2397-7132"[IS] OR "0098-7921"[IS] OR "1728-4457"[IS] OR "1079-5642"[IS] OR "1524-4636"[IS] OR "0897-4756"[IS] OR "1520-5002"[IS] OR "2367-2617"[IS] OR "2367-0983"[IS] OR "2157-6904"[IS] OR "2157-6912"[IS] OR "2095-5162"[IS] OR "2049-9957"[IS] OR "0020-7640"[IS] OR "1741-2854"[IS] OR "0012-186X"[IS] OR "1432-0428"[IS] OR "1941-3289"[IS] OR "1941-3297"[IS] OR "0945-053X"[IS] OR "1569-1802"[IS] OR "0278-4319"[IS] OR "1873-4693"[IS] OR "N/A"[IS] OR "1880-8190"[IS] OR "1569-9056"[IS] OR "1878-1500"[IS] OR "0049-3848"[IS] OR "1879-2472"[IS] OR "2330-8249"[IS] OR "2330-8257"[IS] OR "N/A"[IS] OR "1744-8603"[IS] OR "0016-5107"[IS] OR "1097-6779"[IS] OR "1944-8244"[IS] OR "1944-8252"[IS] OR "1674-7305"[IS] OR "1869-1889"[IS] OR "1043-6618"[IS] OR "1096-1186"[IS] OR "0079-6727"[IS] OR "1873-1627"[IS] OR "0890-6955"[IS] OR "1879-2170"[IS] OR "0378-7206"[IS] OR "1872-7530"[IS] OR "0028-646X"[IS] OR "1469-8137"[IS] OR "1005-0302"[IS] OR "1941-1162"[IS] OR "2110-5820"[IS] OR "2110-5820"[IS] OR "0890-8044"[IS] OR "1558-156X"[IS] OR "0958-1669"[IS] OR "1879-0429"[IS] OR "1949-3053"[IS] OR "1949-3061"[IS] OR "1684-1182"[IS] OR "1995-9133"[IS] OR "1998-0124"[IS] OR "1998-0000"[IS] OR "2210-6502"[IS] OR "2210-6510"[IS] OR "0012-3692"[IS] OR "1931-3543"[IS] OR "2327-4662"[IS] OR "2327-4662"[IS] OR "1726-2135"[IS] OR "1684-8799"[IS] OR "1747-938X"[IS] OR "1878-0385"[IS] OR "1521-6616"[IS] OR "1521-7035"[IS] OR "0144-1647"[IS] OR "1464-5327"[IS] OR "2162-2531"[IS] OR "2162-2531"[IS] OR "2040-7122"[IS] OR "2040-7130"[IS] OR "0956-7976"[IS] OR "1467-9280"[IS] OR "1931-5244"[IS] OR "1878-1810"[IS] OR "0039-2499"[IS] OR "1524-4628"[IS] OR "1674-7291"[IS] OR "1869-1870"[IS] OR "0065-3195"[IS] OR "1436-5030"[IS] OR "1449-4035"[IS] OR "1839-3373"[IS] OR "0166-3542"[IS] OR "1872-9096"[IS] OR "0736-5845"[IS] OR "1879-2537"[IS] OR "N/A"[IS] OR "2397-768X"[IS] OR "0894-3796"[IS] OR "1099-1379"[IS] OR "1757-7780"[IS] OR "1757-7799"[IS] OR "1755-263X"[IS] OR "1755-263X"[IS] OR "1093-9687"[IS] OR "1467-8667"[IS] OR "0020-9996"[IS] OR "1536-0210"[IS] OR "1619-7070"[IS] OR "1619-7089"[IS] OR "1094-6705"[IS] OR "1552-7379"[IS] OR "2195-1071"[IS] OR "2195-1071"[IS] OR "1366-5545"[IS] OR "1878-5794"[IS] OR "2196-5404"[IS] OR "2196-5404"[IS] OR "2631-8644"[IS] OR "2631-7990"[IS] OR "0360-0572"[IS] OR "1545-2115"[IS] OR "1873-9946"[IS] OR "1876-4479"[IS] OR "2211-467X"[IS] OR "2211-4688"[IS] OR "0959-8049"[IS] OR "1879-0852"[IS] OR "2095-8137"[IS] OR "N/A"[IS] OR "0887-7963"[IS] OR "1532-9496"[IS] OR "0167-9236"[IS] OR "1873-5797"[IS] OR "2643-6515"[IS] OR "2643-6515"[IS] OR "2045-8118"[IS] OR "2045-8118"[IS] OR "0271-678X"[IS] OR "1559-7016"[IS] OR "2574-0962"[IS] OR "N/A"[IS] OR "0964-4563"[IS] OR "1468-3318"[IS] OR "1747-4930"[IS] OR "1747-4949"[IS] OR "1674-0769"[IS] OR "1995-820X"[IS] OR "1748-9326"[IS] OR "1748-9326"[IS] OR "0044-7447"[IS] OR "1654-7209"[IS] OR "1040-7308"[IS] OR "1573-6660"[IS] OR "1527-8999"[IS] OR "1528-0691"[IS] OR "1367-4803"[IS] OR "1460-2059"[IS] OR "0361-476X"[IS] OR "1090-2384"[IS] OR "0008-543X"[IS] OR "1097-0142"[IS] OR "0144-6665"[IS] OR "2044-8309"[IS] OR "0025-7125"[IS] OR "1557-9859"[IS] OR "0003-2670"[IS] OR "1873-4324"[IS] OR "2212-0416"[IS] OR "2212-0416"[IS] OR "1466-822X"[IS] OR "1466-8238"[IS] OR "1097-6647"[IS] OR "1532-429X"[IS] OR "0009-9236"[IS] OR "1532-6535"[IS] OR "0013-7952"[IS] OR "1872-6917"[IS] OR "0167-8140"[IS] OR "1879-0887"[IS] OR "1991-959X"[IS] OR "1991-9603"[IS] OR "N/A"[IS] OR "2397-2106"[IS] OR "1948-7185"[IS] OR "N/A"[IS] OR "0278-3649"[IS] OR "1741-3176"[IS] OR "N/A"[IS] OR "2059-7029"[IS] OR "0047-2778"[IS] OR "1540-627X"[IS] OR "0160-791X"[IS] OR "1879-3274"[IS] OR "0143-5221"[IS] OR "1470-8736"[IS] OR "0360-0564"[IS] OR "2163-0747"[IS] OR "2050-6406"[IS] OR "2050-6414"[IS] OR "0021-8901"[IS] OR "1365-

	2664"[IS] OR "0950-1991"[IS] OR "1477-9129"[IS] OR "2053-1583"[IS] OR "2053-1583"[IS] OR "0022-0663"[IS] OR "1939-2176"[IS] OR "N/A"[IS] OR "2397-4648"[IS] OR "1365-1609"[IS] OR "1873-4545"[IS] OR "0021-9150"[IS] OR "1879-1484"[IS] OR "2167-0811"[IS] OR "2167-082X"[IS] OR "0029-6643"[IS] OR "1753-4887"[IS] OR "0953-7287"[IS] OR "1366-5871"[IS] OR "0960-3166"[IS] OR "1573-5184"[IS] OR "0010-7514"[IS] OR "1366-5812"[IS] OR "1552-3098"[IS] OR "1941-0468"[IS] OR "2165-5979"[IS] OR "2165-5987"[IS] OR "1071-7544"[IS] OR "1521-0464"[IS] OR "0742-2091"[IS] OR "1573-6822"[IS] OR "1011-1344"[IS] OR "1873-2682"[IS] OR "2398-4902"[IS] OR "2398-4902"[IS] OR "2352-250X"[IS] OR "2352-2518"[IS] OR "0906-7590"[IS] OR "1600-0587"[IS] OR "1868-4483"[IS] OR "1868-601X"[IS] OR "1472-8222"[IS] OR "1744-7631"[IS] OR "1001-0742"[IS] OR "1878-7320"[IS] OR "1888-9891"[IS] OR "1989-4600"[IS] OR "N/A"[IS] OR "2199-4730"[IS] OR "1545-5971"[IS] OR "1941-0018"[IS] OR "0735-1933"[IS] OR "1879-0178"[IS] OR "N/A"[IS] OR "1476-0711"[IS] OR "0024-3205"[IS] OR "1879-0631"[IS] OR "1547-5271"[IS] OR "1556-3871"[IS] OR "0020-7403"[IS] OR "1879-2162"[IS] OR "0944-1174"[IS] OR "1435-5922"[IS] OR "2199-1170"[IS] OR "2198-9761"[IS] OR "0308-521X"[IS] OR "1873-2267"[IS] OR "2160-6056"[IS] OR "2160-6064"[IS] OR "0196-0644"[IS] OR "1097-6760"[IS] OR "1226-086X"[IS] OR "1876-794X"[IS] OR "0168-1699"[IS] OR "1872-7107"[IS] OR "0925-5214"[IS] OR "1873-2356"[IS] OR "0033-5770"[IS] OR "1539-7718"[IS] OR "1541-1672"[IS] OR "1941-1294"[IS] OR "0013-9580"[IS] OR "1528-1167"[IS] OR "0007-6503"[IS] OR "1552-4205"[IS] OR "N/A"[IS] OR "2576-604X"[IS] OR "2191-9089"[IS] OR "2191-9097"[IS] OR "1866-7910"[IS] OR "1866-7929"[IS] OR "2366-7486"[IS] OR "2366-7486"[IS] OR "1749-4613"[IS] OR "1878-0253"[IS] OR "0272-6963"[IS] OR "1873-1317"[IS] OR "1945-7707"[IS] OR "1945-7715"[IS] OR "1354-3776"[IS] OR "1744-7674"[IS] OR "0832-610X"[IS] OR "1496-8975"[IS] OR "0270-6474"[IS] OR "1529-2401"[IS] OR "0022-1694"[IS] OR "1879-2707"[IS] OR "N/A"[IS] OR "1758-2652"[IS] OR "N/A"[IS] OR "2072-6643"[IS] OR "2199-4536"[IS] OR "2198-6053"[IS] OR "0010-4825"[IS] OR "1879-0534"[IS] OR "2629-3269"[IS] OR "2629-3277"[IS] OR "0014-2980"[IS] OR "1521-4141"[IS] OR "1362-3613"[IS] OR "1461-7005"[IS] OR "0340-5354"[IS] OR "1432-1459"[IS] OR "0340-6245"[IS] OR "2567-689X"[IS] OR "0305-750X"[IS] OR "1873-5991"[IS] OR "0022-2275"[IS] OR "1539-7262"[IS] OR "0962-8436"[IS] OR "1471-2970"[IS] OR "0011-1384"[IS] OR "1745-9125"[IS] OR "0022-2437"[IS] OR "1547-7193"[IS] OR "2212-0955"[IS] OR "2212-0955"[IS] OR "0944-7113"[IS] OR "1618-095X"[IS] OR "0956-7135"[IS] OR "1873-7129"[IS] OR "1935-9772"[IS] OR "1935-9780"[IS] OR "1813-7253"[IS] OR "1861-6909"[IS] OR "1359-2998"[IS] OR "1468-2052"[IS] OR "0091-6331"[IS] OR "1538-3008"[IS] OR "0959-4752"[IS] OR "1873-3263"[IS] OR "1545-5955"[IS] OR "1558-3783"[IS] OR "2166-532X"[IS] OR "2166-532X"[IS] OR "0300-0729"[IS] OR "0300-0729"[IS] OR "0167-7322"[IS] OR "1873-3166"[IS] OR "0925-4439"[IS] OR "1879-260X"[IS] OR "0340-7004"[IS] OR "1432-0851"[IS] OR "0003-2999"[IS] OR "0003-2999"[IS] OR "1664-462X"[IS] OR "1664-462X"[IS] OR "1110-0168"[IS] OR "2090-2670"[IS] OR "1040-8428"[IS] OR "1879-0461"[IS] OR "1976-7633"[IS] OR "1976-7951"[IS] OR "0962-1083"[IS] OR "1365-294X"[IS] OR "1941-1340"[IS] OR "1941-1359"[IS] OR "1027-5606"[IS] OR "1607-7938"[IS] OR "0965-8564"[IS] OR "1879-2375"[IS] OR "0828-282X"[IS] OR "1916-7075"[IS] OR "1753-0350"[IS] OR "1753-0369"[IS] OR "0020-7489"[IS] OR "1873-491X"[IS] OR "0378-3774"[IS] OR "1873-2283"[IS] OR "0171-8177"[IS] OR "2363-7102"[IS] OR "0178-2762"[IS] OR "1432-0789"[IS] OR "0749-3797"[IS] OR "1873-2607"[IS] OR "0889-3268"[IS] OR "1573-353X"[IS] OR "0263-8223"[IS] OR "1879-1085"[IS] OR "1524-8380"[IS] OR "1552-8324"[IS] OR "2168-6750"[IS] OR "2168-6750"[IS] OR "1071-9164"[IS] OR "1532-8414"[IS] OR "1079-5006"[IS] OR "1758-535X"[IS] OR "2296-861X"[IS] OR "2296-861X"[IS] OR "0045-7825"[IS] OR "1879-2138"[IS] OR "2473-2877"[IS] OR "2473-2877"[IS] OR "0361-803X"[IS] OR "1546-3141"[IS] OR "1549-9618"[IS] OR "1549-9626"[IS] OR "0167-8809"[IS] OR "1873-2305"[IS] OR "1751-7915"[IS] OR "1751-7915"[IS] OR "N/A"[IS] OR "2072-6694"[IS] OR "1613-4125"[IS] OR "1613-4133"[IS] OR "2083-1277"[IS] OR "2353-1827"[IS] OR "1350-6129"[IS] OR "1744-2818"[IS] OR "2058-9565"[IS] OR "2058-9565"[IS] OR "0920-5861"[IS] OR "1873-4308"[IS] OR "0804-4643"[IS] OR "1479-683X"[IS] OR "0021-9355"[IS] OR "1535-1386"[IS] OR "N/A"[IS] OR "2192-1962"[IS] OR "0039-9140"[IS] OR "1873-3573"[IS] OR "2327-0012"[IS] OR "2327-0039"[IS] OR "2542-4653"[IS] OR "2542-4653"[IS] OR "0905-6947"[IS] OR "1600-0668"[IS] OR "0022-538X"[IS] OR "1098-5514"[IS] OR "0018-9286"[IS] OR "1558-2523"[IS] OR "N/A"[IS] OR "2399-3642"[IS] OR "0013-9165"[IS] OR "1552-390X"[IS] OR "0921-8009"[IS] OR "1873-6106"[IS] OR "1540-7489"[IS] OR "1873-2704"[IS] OR "0165-0327"[IS] OR "1573-2517"[IS] OR "1072-7515"[IS] OR "1879-1190"[IS] OR "0091-3367"[IS] OR "1557-7805"[IS] OR "N/A"[IS] OR "2168-1007"[IS] OR "N/A"[IS] OR "1999-4923"[IS] OR "2157-9024"[IS] OR "2157-9024"[IS] OR "0891-1150"[IS] OR "1939-2869"[IS] OR "N/A"[IS] OR "2196-5412"[IS] OR "0899-823X"[IS] OR "1559-6834"[IS] OR "1994-2060"[IS] OR "1997-003X"[IS] OR "2332-7782"[IS] OR "2332-7782"[IS] OR "1347-9032"[IS] OR "1349-7006"[IS] OR "0723-2632"[IS] OR "1434-453X"[IS] OR "N/A"[IS] OR "2050-0068"[IS] OR "0021-9541"[IS] OR "1097-4652"[IS] OR "0272-8087"[IS] OR "1098-8971"[IS] OR "0378-5173"[IS] OR "1873-3476"[IS] OR "1323-3580"[IS] OR "1448-6083"[IS] OR "1050-7256"[IS] OR "1557-9077"[IS] OR "0886-6236"[IS] OR "1944-9224"[IS] OR "0077-8923"[IS] OR "1749-6632"[IS] OR "1354-3784"[IS] OR "1744-7658"[IS] OR "1172-7047"[IS] OR "1179-1934"[IS] OR "2399-3650"[IS] OR "2399-3650"[IS] OR

	"2287-4208"[IS] OR "2287-4690"[IS] OR "N/A"[IS] OR "2056-3744"[IS] OR "1871-6784"[IS] OR "1876-4347"[IS] OR "0090-3752"[IS] OR "1095-9904"[IS] OR "0034-6535"[IS] OR "1530-9142"[IS] OR "1473-9542"[IS] OR "1479-7364"[IS] OR "2040-2392"[IS] OR "2040-2392"[IS] OR "0884-8734"[IS] OR "1525-1497"[IS] OR "1743-9671"[IS] OR "1746-0220"[IS] OR "1882-7616"[IS] OR "2213-6851"[IS] OR "1359-4311"[IS] OR "1873-5606"[IS] OR "N/A"[IS] OR "2296-2565"[IS] OR "0737-0024"[IS] OR "1532-7051"[IS] OR "1549-9634"[IS] OR "1549-9642"[IS] OR "2047-2994"[IS] OR "2047-2994"[IS] OR "0198-9715"[IS] OR "1873-7587"[IS] OR "0926-6690"[IS] OR "1872-633X"[IS] OR "2590-1575"[IS] OR "2590-1575"[IS] OR "1942-0862"[IS] OR "1942-0870"[IS] OR "0022-5223"[IS] OR "1097-685X"[IS] OR "2521-327X"[IS] OR "2521-327X"[IS] OR "0951-6433"[IS] OR "1872-8081"[IS] OR "0165-2370"[IS] OR "1873-250X"[IS] OR "0012-9658"[IS] OR "1939-9170"[IS] OR "1756-2856"[IS] OR "1756-2864"[IS] OR "N/A"[IS] OR "1475-2867"[IS] OR "1078-5884"[IS] OR "1532-2165"[IS] OR "1462-9011"[IS] OR "1873-6416"[IS] OR "0168-1923"[IS] OR "1873-2240"[IS] OR "1937-6448"[IS] OR "N/A"[IS] OR "0105-1873"[IS] OR "1600-0536"[IS] OR "0742-597X"[IS] OR "1943-5479"[IS] OR "0266-2426"[IS] OR "1741-2870"[IS] OR "1672-0229"[IS] OR "2210-3244"[IS] OR "1462-8902"[IS] OR "1463-1326"[IS] OR "0026-3672"[IS] OR "1436-5073"[IS] OR "0898-5626"[IS] OR "1464-5114"[IS] OR "0886-7798"[IS] OR "1878-4364"[IS] OR "0094-6176"[IS] OR "1098-9064"[IS] OR "1548-1603"[IS] OR "1943-7226"[IS] OR "1931-3896"[IS] OR "1931-390X"[IS] OR "1110-9823"[IS] OR "2090-2476"[IS] OR "0925-7535"[IS] OR "1879-1042"[IS] OR "0029-6651"[IS] OR "1475-2719"[IS] OR "0884-0431"[IS] OR "1523-4681"[IS] OR "2196-7350"[IS] OR "2196-7350"[IS] OR "0951-418X"[IS] OR "1099-1573"[IS] OR "0013-189X"[IS] OR "1935-102X"[IS] OR "1556-4959"[IS] OR "1556-4967"[IS] OR "0012-9682"[IS] OR "1468-0262"[IS] OR "2378-0967"[IS] OR "2378-0967"[IS] OR "0022-0477"[IS] OR "1365-2745"[IS] OR "1029-8479"[IS] OR "1029-8479"[IS] OR "2328-8604"[IS] OR "2328-8620"[IS] OR "1076-1551"[IS] OR "1528-3658"[IS] OR "0001-4575"[IS] OR "1879-2057"[IS] OR "0740-0020"[IS] OR "1095-9998"[IS] OR "2199-3971"[IS] OR "2199-398X"[IS] OR "1559-0631"[IS] OR "1559-064X"[IS] OR "0925-8388"[IS] OR "1873-4669"[IS] OR "1469-1930"[IS] OR "1758-7468"[IS] OR "0341-8162"[IS] OR "1872-6887"[IS] OR "0955-2219"[IS] OR "1873-619X"[IS] OR "0377-2217"[IS] OR "1872-6860"[IS] OR "N/A"[IS] OR "2524-6372"[IS] OR "2332-7731"[IS] OR "2332-7731"[IS] OR "2169-1401"[IS] OR "2169-141X"[IS] OR "0268-1161"[IS] OR "1460-2350"[IS] OR "1066-2243"[IS] OR "1066-2243"[IS] OR "1540-1960"[IS] OR "1540-1979"[IS] OR "N/A"[IS] OR "1475-2859"[IS] OR "2468-5925"[IS] OR "2352-8648"[IS] OR "0950-3293"[IS] OR "1873-6343"[IS] OR "1357-633X"[IS] OR "1758-1109"[IS] OR "0743-9156"[IS] OR "1547-7207"[IS] OR "2471-1403"[IS] OR "2471-1403"[IS] OR "0915-5635"[IS] OR "1443-1661"[IS] OR "0969-5893"[IS] OR "1468-2850"[IS] OR "1541-7786"[IS] OR "1557-3125"[IS] OR "0300-2896"[IS] OR "1579-2129"[IS] OR "2368-7959"[IS] OR "2368-7959"[IS] OR "0167-4544"[IS] OR "1573-0697"[IS] OR "1516-4446"[IS] OR "1809-452X"[IS] OR "0266-903X"[IS] OR "1460-2121"[IS] OR "0129-0657"[IS] OR "1793-6462"[IS] OR "0091-7613"[IS] OR "1943-2682"[IS] OR "0033-8222"[IS] OR "1945-5755"[IS] OR "1001-0521"[IS] OR "1867-7185"[IS] OR "2042-6496"[IS] OR "2042-650X"[IS] OR "0009-8981"[IS] OR "1873-3492"[IS] OR "0022-3476"[IS] OR "1097-6833"[IS] OR "0033-8362"[IS] OR "1826-6983"[IS] OR "0161-8105"[IS] OR "1550-9109"[IS] OR "0271-5333"[IS] OR "N/A"[IS] OR "2372-7705"[IS] OR "2372-7705"[IS] OR "1874-9399"[IS] OR "1876-4320"[IS] OR "0065-2423"[IS] OR "2162-9471"[IS] OR "1359-6462"[IS] OR "1872-8456"[IS] OR "0960-1643"[IS] OR "1478-5242"[IS] OR "0166-5162"[IS] OR "1872-7840"[IS] OR "0093-6502"[IS] OR "1552-3810"[IS] OR "0147-5185"[IS] OR "1532-0979"[IS] OR "0195-9131"[IS] OR "1530-0315"[IS] OR "0735-2689"[IS] OR "1549-7836"[IS] OR "1351-5101"[IS] OR "1468-1331"[IS] OR "0143-005X"[IS] OR "1470-2738"[IS] OR "0168-1702"[IS] OR "1872-7492"[IS] OR "1350-7540"[IS] OR "1473-6551"[IS] OR "1476-9255"[IS] OR "1476-9255"[IS] OR "0269-8463"[IS] OR "1365-2435"[IS] OR "0190-6011"[IS] OR "1938-1344"[IS] OR "1534-4843"[IS] OR "1552-6712"[IS] OR "N/A"[IS] OR "2665-9271"[IS] OR "1542-0124"[IS] OR "1937-5913"[IS] OR "2214-157X"[IS] OR "2214-157X"[IS] OR "2238-7854"[IS] OR "2214-0697"[IS] OR "1089-3261"[IS] OR "1557-8224"[IS] OR "1470-160X"[IS] OR "1872-7034"[IS] OR "1662-5099"[IS] OR "1662-5099"[IS] OR "1000-6818"[IS] OR "N/A"[IS] OR "0300-9572"[IS] OR "1873-1570"[IS] OR "0277-0008"[IS] OR "1875-9114"[IS] OR "1868-596X"[IS] OR "1868-8551"[IS] OR "0305-1846"[IS] OR "1365-2990"[IS] OR "1754-1611"[IS] OR "1754-1611"[IS] OR "1081-1206"[IS] OR "1534-4436"[IS] OR "1546-1440"[IS] OR "1558-349X"[IS] OR "0004-6361"[IS] OR "1432-0746"[IS] OR "0018-9545"[IS] OR "1939-9359"[IS] OR "0887-378X"[IS] OR "1468-0009"[IS] OR "0090-4848"[IS] OR "1099-050X"[IS] OR "2468-0249"[IS] OR "2468-0249"[IS] OR "1175-0561"[IS] OR "1179-1888"[IS] OR "0022-3751"[IS] OR "1469-7793"[IS] OR "1756-5383"[IS] OR "1756-5391"[IS] OR "N/A"[IS] OR "2330-6696"[IS] OR "1476-5586"[IS] OR "1476-5586"[IS] OR "1878-5352"[IS] OR "1878-5379"[IS] OR "N/A"[IS] OR "1422-0067"[IS] OR "2196-9744"[IS] OR "2196-9744"[IS] OR "0300-9440"[IS] OR "1873-331X"[IS] OR "0260-8774"[IS] OR "1873-5770"[IS] OR "0039-6257"[IS] OR "1879-3304"[IS] OR "1465-3249"[IS] OR "1477-2566"[IS] OR "0143-5671"[IS] OR "1475-5890"[IS] OR "0264-8377"[IS] OR "1873-5754"[IS] OR "1040-8444"[IS] OR "1547-6898"[IS] OR "1070-5511"[IS] OR "1532-8007"[IS] OR "2044-4753"[IS] OR "2044-4761"[IS] OR "1058-4609"[IS] OR "1091-7675"[IS] OR "1323-7799"[IS] OR "1440-1843"[IS] OR "1674-9782"[IS] OR "2049-1891"[IS] OR "0967-070X"[IS] OR "1879-

	310X"[IS] OR "0025-1909"[IS] OR "1526-5501"[IS] OR "0340-5761"[IS] OR "1432-0738"[IS] OR "N/A"[IS] OR "2288-5048"[IS] OR "0090-6778"[IS] OR "1558-0857"[IS] OR "1549-9596"[IS] OR "1549-960X"[IS] OR "0924-0136"[IS] OR "1873-4774"[IS] OR "1750-8592"[IS] OR "1750-8606"[IS] OR "1053-1858"[IS] OR "1477-9803"[IS] OR "0043-1397"[IS] OR "1944-7973"[IS] OR "1021-9498"[IS] OR "1021-9498"[IS] OR "2001-0370"[IS] OR "2001-0370"[IS] OR "1612-510X"[IS] OR "1612-5118"[IS] OR "1342-937X"[IS] OR "1878-0571"[IS] OR "0022-2836"[IS] OR "1089-8638"[IS] OR "1554-7191"[IS] OR "1555-1938"[IS] OR "0005-1098"[IS] OR "1873-2836"[IS] OR "1537-260X"[IS] OR "N/A"[IS] OR "N/A"[IS] OR "1662-5102"[IS] OR "0378-4290"[IS] OR "1872-6852"[IS] OR "1398-9219"[IS] OR "1600-0854"[IS] OR "1229-9367"[IS] OR "1598-6233"[IS] OR "0275-5319"[IS] OR "1878-3384"[IS] OR "N/A"[IS] OR "2574-0970"[IS] OR "1861-0684"[IS] OR "1861-0692"[IS] OR "1069-2509"[IS] OR "1875-8835"[IS] OR "2468-0230"[IS] OR "2468-0230"[IS] OR "2152-2715"[IS] OR "2152-2723"[IS] OR "0021-972X"[IS] OR "1945-7197"[IS] OR "0366-6999"[IS] OR "2542-5641"[IS] OR "0018-9200"[IS] OR "1558-173X"[IS] OR "2405-500X"[IS] OR "2405-5018"[IS] OR "0969-0239"[IS] OR "1572-882X"[IS] OR "0195-9255"[IS] OR "1873-6432"[IS] OR "2193-8229"[IS] OR "2193-6382"[IS] OR "0081-1750"[IS] OR "N/A"[IS] OR "0955-2863"[IS] OR "1873-4847"[IS] OR "2211-3398"[IS] OR "2211-3398"[IS] OR "1877-8585"[IS] OR "1877-8593"[IS] OR "N/A"[IS] OR "2296-889X"[IS] OR "1527-6465"[IS] OR "1527-6473"[IS] OR "0263-2373"[IS] OR "1873-5681"[IS] OR "N/A"[IS] OR "2589-0042"[IS] OR "N/A"[IS] OR "2047-9980"[IS] OR "1051-0761"[IS] OR "1939-5582"[IS] OR "0006-2952"[IS] OR "1873-2968"[IS] OR "1743-5889"[IS] OR "1748-6963"[IS] OR "0840-6529"[IS] OR "1209-1367"[IS] OR "1098-3058"[IS] OR "1943-4294"[IS] OR "2468-2047"[IS] OR "2468-080X"[IS] OR "1933-7213"[IS] OR "1878-7479"[IS] OR "N/A"[IS] OR "1660-3397"[IS] OR "N/A"[IS] OR "2056-7944"[IS] OR "1040-3590"[IS] OR "1939-134X"[IS] OR "2157-1724"[IS] OR "2157-1716"[IS] OR "1050-8406"[IS] OR "1532-7809"[IS] OR "0169-5002"[IS] OR "1872-8332"[IS] OR "2296-634X"[IS] OR "2296-634X"[IS] OR "0306-9192"[IS] OR "1873-5657"[IS] OR "0264-2751"[IS] OR "1873-6084"[IS] OR "0333-1024"[IS] OR "1468-2982"[IS] OR "1536-1233"[IS] OR "1558-0660"[IS] OR "0194-4363"[IS] OR "1939-0130"[IS] OR "2235-2988"[IS] OR "2235-2988"[IS] OR "1523-7060"[IS] OR "1523-7052"[IS] OR "1043-1802"[IS] OR "1520-4812"[IS] OR "1945-7731"[IS] OR "1945-774X"[IS] OR "2053-9711"[IS] OR "2053-9711"[IS] OR "1359-7345"[IS] OR "1364-548X"[IS] OR "2296-4185"[IS] OR "2296-4185"[IS] OR "N/A"[IS] OR "2218-273X"[IS] OR "N/A"[IS] OR "1664-302X"[IS] OR "0305-9006"[IS] OR "1873-4510"[IS] OR "N/A"[IS] OR "2399-5300"[IS] OR "1673-5374"[IS] OR "1876-7958"[IS] OR "1545-2255"[IS] OR "1545-2263"[IS] OR "0024-9297"[IS] OR "1520-5835"[IS] OR "1469-3062"[IS] OR "1752-7457"[IS] OR "0023-6438"[IS] OR "1096-1127"[IS] OR "1664-2392"[IS] OR "1664-2392"[IS] OR "2451-9022"[IS] OR "2451-9030"[IS] OR "2095-2201"[IS] OR "2095-221X"[IS] OR "0921-5093"[IS] OR "1873-4936"[IS] OR "N/A"[IS] OR "2566-6223"[IS] OR "8755-9129"[IS] OR "1525-6103"[IS] OR "2452-0748"[IS] OR "2452-0748"[IS] OR "1066-2316"[IS] OR "1936-1351"[IS] OR "0022-4405"[IS] OR "1873-3506"[IS] OR "0017-811X"[IS] OR "2161-976X"[IS] OR "1528-4042"[IS] OR "1534-6293"[IS] OR "0098-8472"[IS] OR "1873-7307"[IS] OR "2572-4568"[IS] OR "2572-4568"[IS] OR "0958-5192"[IS] OR "1466-4399"[IS] OR "1553-7404"[IS] OR "1553-7404"[IS] OR "0171-9335"[IS] OR "1618-1298"[IS] OR "1019-6781"[IS] OR "1422-8890"[IS] OR "1751-8253"[IS] OR "1751-7192"[IS] OR "2041-8396"[IS] OR "2041-840X"[IS] OR "2096-0042"[IS] OR "2096-0042"[IS] OR "N/A"[IS] OR "2632-2153"[IS] OR "1040-0605"[IS] OR "1522-1504"[IS] OR "0741-5400"[IS] OR "1938-3673"[IS] OR "0253-6269"[IS] OR "1976-3786"[IS] OR "1535-7163"[IS] OR "1538-8514"[IS] OR "0192-415X"[IS] OR "1793-6853"[IS] OR "1471-9037"[IS] OR "1471-9045"[IS] OR "0079-6638"[IS] OR "N/A"[IS] OR "0036-8326"[IS] OR "1098-237X"[IS] OR "1070-4698"[IS] OR "1559-8985"[IS] OR "0078-3218"[IS] OR "N/A"[IS] OR "1087-2981"[IS] OR "1087-2981"[IS] OR "0927-7765"[IS] OR "1873-4367"[IS] OR "1355-2554"[IS] OR "1758-6534"[IS] OR "1367-5567"[IS] OR "1469-848X"[IS] OR "N/A"[IS] OR "1663-9812"[IS] OR "1615-4150"[IS] OR "1615-4169"[IS] OR "1042-9573"[IS] OR "1096-0473"[IS] OR "0749-8063"[IS] OR "1526-3231"[IS] OR "1368-8375"[IS] OR "1879-0593"[IS] OR "1066-033X"[IS] OR "1941-000X"[IS] OR "1941-1383"[IS] OR "1941-1391"[IS] OR "1464-4096"[IS] OR "1464-410X"[IS] OR "1523-3804"[IS] OR "1534-6242"[IS] OR "0885-8993"[IS] OR "1941-0107"[IS] OR "0399-077X"[IS] OR "1769-6690"[IS] OR "0169-8095"[IS] OR "1873-2895"[IS] OR "0958-8221"[IS] OR "1744-3210"[IS] OR "0033-6807"[IS] OR "1467-9310"[IS] OR "2210-6006"[IS] OR "2210-6014"[IS] OR "1757-1693"[IS] OR "1757-1707"[IS] OR "1755-2966"[IS] OR "1878-0199"[IS] OR "1945-4589"[IS] OR "N/A"[IS] OR "0959-6658"[IS] OR "1460-2423"[IS] OR "1554-351X"[IS] OR "1554-3528"[IS] OR "N/A"[IS] OR "2475-0379"[IS] OR "1741-7597"[IS] OR "1751-7737"[IS] OR "N/A"[IS] OR "2405-4569"[IS] OR "1523-3790"[IS] OR "1534-6269"[IS] OR "1087-2914"[IS] OR "1557-7449"[IS] OR "2095-4719"[IS] OR "2052-3289"[IS] OR "2156-6976"[IS] OR "2156-6976"[IS] OR "0022-2593"[IS] OR "1468-6244"[IS] OR "N/A"[IS] OR "2313-0105"[IS] OR "0066-4804"[IS] OR "1098-6596"[IS] OR "0955-3959"[IS] OR "1873-4758"[IS] OR "0002-9343"[IS] OR "1555-7162"[IS] OR "0306-6800"[IS] OR "1473-4257"[IS] OR "0250-6807"[IS] OR "1421-9697"[IS] OR "0016-7037"[IS] OR "1872-9533"[IS] OR "2092-7193"[IS] OR "2092-7193"[IS] OR "1674-7755"[IS] OR "2589-0417"[IS] OR "N/A"[IS] OR "1743-422X"[IS] OR "0019-0578"[IS] OR "1879-2022"[IS] OR "0168-1605"[IS] OR "1879-3460"[IS] OR "1873-9601"[IS] OR "1873-961X"[IS] OR "0007-1161"[IS] OR "1468-2079"[IS] OR

	"0169-1317"[IS] OR "1872-9053"[IS] OR "0160-6689"[IS] OR "1555-2101"[IS] OR "0891-5520"[IS] OR "1557-9824"[IS] OR "1568-9883"[IS] OR "1878-1470"[IS] OR "1697-2600"[IS] OR "1576-7329"[IS] OR "0193-1849"[IS] OR "1522-1555"[IS] OR "1351-0088"[IS] OR "1479-6821"[IS] OR "0966-6923"[IS] OR "1873-1236"[IS] OR "0021-8561"[IS] OR "1520-5118"[IS] OR "2396-9873"[IS] OR "2396-9881"[IS] OR "2233-6079"[IS] OR "2233-6087"[IS] OR "0265-0487"[IS] OR "1759-3948"[IS] OR "2096-5524"[IS] OR "2522-8552"[IS] OR "1019-6439"[IS] OR "1791-2423"[IS] OR "0018-9529"[IS] OR "1558-1721"[IS] OR "0263-8231"[IS] OR "1879-3223"[IS] OR "0340-6717"[IS] OR "1432-1203"[IS] OR "1743-5390"[IS] OR "1743-5404"[IS] OR "2156-3357"[IS] OR "2156-3365"[IS] OR "1387-1811"[IS] OR "1873-3093"[IS] OR "0969-2126"[IS] OR "1878-4186"[IS] OR "1083-4435"[IS] OR "1941-014X"[IS] OR "0210-5705"[IS] OR "0210-5705"[IS] OR "0037-7732"[IS] OR "1534-7605"[IS] OR "2048-8505"[IS] OR "2048-8513"[IS] OR "1616-5187"[IS] OR "1616-5195"[IS] OR "1051-8215"[IS] OR "1558-2205"[IS] OR "1352-4585"[IS] OR "1477-0970"[IS] OR "1478-7210"[IS] OR "1744-8336"[IS] OR "0929-1903"[IS] OR "1476-5500"[IS] OR "0818-9641"[IS] OR "1440-1711"[IS] OR "1598-2629"[IS] OR "2092-6685"[IS] OR "2214-367X"[IS] OR "2214-3688"[IS] OR "N/A"[IS] OR "2329-0501"[IS] OR "N/A"[IS] OR "2515-7639"[IS] OR "2297-055X"[IS] OR "2297-055X"[IS] OR "1066-5099"[IS] OR "1549-4918"[IS] OR "0004-6280"[IS] OR "1538-3873"[IS] OR "0007-1420"[IS] OR "1471-8391"[IS] OR "0095-0696"[IS] OR "1096-0449"[IS] OR "0266-1144"[IS] OR "1879-3584"[IS] OR "2374-4235"[IS] OR "2374-4243"[IS] OR "1083-7159"[IS] OR "1549-490X"[IS] OR "1383-7621"[IS] OR "1873-6165"[IS] OR "0892-6638"[IS] OR "1530-6860"[IS] OR "N/A"[IS] OR "2000-2297"[IS] OR "0959-3535"[IS] OR "1461-7161"[IS] OR "2452-2627"[IS] OR "2452-2627"[IS] OR "N/A"[IS] OR "1746-4811"[IS] OR "1462-2203"[IS] OR "1469-994X"[IS] OR "2053-1400"[IS] OR "2053-1419"[IS] OR "N/A"[IS] OR "1999-4915"[IS] OR "1179-1349"[IS] OR "1179-1349"[IS] OR "1878-9293"[IS] OR "1878-9307"[IS] OR "2056-5933"[IS] OR "2056-5933"[IS] OR "1994-0416"[IS] OR "1994-0424"[IS] OR "1873-9318"[IS] OR "1873-9326"[IS] OR "0887-3801"[IS] OR "1943-5487"[IS] OR "1099-3460"[IS] OR "1468-2869"[IS] OR "1109-9666"[IS] OR "2241-5955"[IS] OR "0276-3478"[IS] OR "1098-108X"[IS] OR "2196-7822"[IS] OR "2196-7822"[IS] OR "N/A"[IS] OR "2056-3485"[IS] OR "0012-821X"[IS] OR "1385-013X"[IS] OR "1382-6689"[IS] OR "1872-7077"[IS] OR "0022-166X"[IS] OR "1548-8004"[IS] OR "2000-8198"[IS] OR "2000-8066"[IS] OR "N/A"[IS] OR "2575-1077"[IS] OR "1948-7193"[IS] OR "1948-7193"[IS] OR "0925-2312"[IS] OR "1872-8286"[IS] OR "0961-9534"[IS] OR "1873-2909"[IS] OR "0304-4130"[IS] OR "1475-6765"[IS] OR "0265-1335"[IS] OR "1758-6763"[IS] OR "0002-9440"[IS] OR "1525-2191"[IS] OR "0010-2180"[IS] OR "1556-2921"[IS] OR "1385-2256"[IS] OR "1573-1618"[IS] OR "0065-230X"[IS] OR "2162-5557"[IS] OR "1618-8667"[IS] OR "1610-8167"[IS] OR "2056-3418"[IS] OR "2056-3426"[IS] OR "0022-2828"[IS] OR "1095-8584"[IS] OR "1932-4391"[IS] OR "1932-443X"[IS] OR "1567-5394"[IS] OR "1878-562X"[IS] OR "0940-1334"[IS] OR "1433-8491"[IS] OR "0305-7453"[IS] OR "1460-2091"[IS] OR "1475-6366"[IS] OR "1475-6374"[IS] OR "1352-2310"[IS] OR "1873-2844"[IS] OR "0020-8183"[IS] OR "1531-5088"[IS] OR "1723-2007"[IS] OR "1723-2007"[IS] OR "0021-9916"[IS] OR "1460-2466"[IS] OR "1558-6898"[IS] OR "1558-6901"[IS] OR "N/A"[IS] OR "2079-6374"[IS] OR "1612-4758"[IS] OR "1612-4766"[IS] OR "0924-090X"[IS] OR "1573-269X"[IS] OR "1537-1891"[IS] OR "1879-3649"[IS] OR "2234-943X"[IS] OR "2234-943X"[IS] OR "1226-8453"[IS] OR "2093-4947"[IS] OR "1754-8403"[IS] OR "1754-8411"[IS] OR "0009-9104"[IS] OR "1365-2249"[IS] OR "N/A"[IS] OR "2641-0397"[IS] OR "1860-6768"[IS] OR "1860-7314"[IS] OR "1134-3478"[IS] OR "1988-3293"[IS] OR "N/A"[IS] OR "2309-608X"[IS] OR "0926-860X"[IS] OR "1873-3875"[IS] OR "1673-8527"[IS] OR "1873-5533"[IS] OR "1161-0301"[IS] OR "1873-7331"[IS] OR "0014-2972"[IS] OR "1365-2362"[IS] OR "N/A"[IS] OR "2079-4991"[IS] OR "1352-3260"[IS] OR "1743-8764"[IS] OR "1567-5769"[IS] OR "1878-1705"[IS] OR "1366-9516"[IS] OR "1472-4642"[IS] OR "0269-2163"[IS] OR "1477-030X"[IS] OR "0167-4730"[IS] OR "1879-3355"[IS] OR "2543-1536"[IS] OR "2542-6605"[IS] OR "2321-3868"[IS] OR "2321-3876"[IS] OR "1475-9217"[IS] OR "1741-3168"[IS] OR "0018-716X"[IS] OR "1423-0054"[IS] OR "1544-1709"[IS] OR "1544-1717"[IS] OR "1538-6341"[IS] OR "1931-2393"[IS] OR "1663-4365"[IS] OR "1663-4365"[IS] OR "N/A"[IS] OR "2471-254X"[IS] OR "2059-7983"[IS] OR "2059-7983"[IS] OR "1180-4882"[IS] OR "1488-2434"[IS] OR "2168-7161"[IS] OR "2168-7161"[IS] OR "1351-0002"[IS] OR "1743-2928"[IS] OR "N/A"[IS] OR "2572-2611"[IS] OR "1441-2772"[IS] OR "N/A"[IS] OR "0109-5641"[IS] OR "1879-0097"[IS] OR "0208-5216"[IS] OR "0208-5216"[IS] OR "2040-4166"[IS] OR "2040-4174"[IS] OR "1040-8347"[IS] OR "1547-6510"[IS] OR "0003-9985"[IS] OR "1543-2165"[IS] OR "1526-6125"[IS] OR "2212-4616"[IS] OR "1476-0584"[IS] OR "1744-8395"[IS] OR "2054-4642"[IS] OR "2054-4650"[IS] OR "0893-7648"[IS] OR "1559-1182"[IS] OR "N/A"[IS] OR "2590-1567"[IS] OR "1461-1457"[IS] OR "1469-5111"[IS] OR "2363-7005"[IS] OR "1867-0202"[IS] OR "2190-393X"[IS] OR "2190-3948"[IS] OR "1473-7159"[IS] OR "1744-8352"[IS] OR "0954-5395"[IS] OR "1748-8583"[IS] OR "1521-690X"[IS] OR "1532-1908"[IS] OR "0890-765X"[IS] OR "1748-0361"[IS] OR "0143-8166"[IS] OR "1873-0302"[IS] OR "1741-0398"[IS] OR "1758-7409"[IS] OR "0009-3920"[IS] OR "1467-8624"[IS] OR "0964-8410"[IS] OR "1467-8683"[IS] OR "0142-0615"[IS] OR "1879-3517"[IS] OR "0018-7267"[IS] OR "1741-282X"[IS] OR "N/A"[IS] OR "2045-7022"[IS] OR "2029-4913"[IS] OR "2029-4921"[IS] OR "0973-0826"[IS] OR "2352-4669"[IS] OR "2530-0644"[IS] OR "2530-0644"[IS] OR "1046-3283"[IS] OR "1479-277X"[IS] OR

	"1357-2725"[IS] OR "1878-5875"[IS] OR "0032-5910"[IS] OR "1873-328X"[IS] OR "1939-8425"[IS] OR "1939-8433"[IS] OR "1355-8382"[IS] OR "1469-9001"[IS] OR "2215-0382"[IS] OR "2215-0382"[IS] OR "0962-7480"[IS] OR "1471-8405"[IS] OR "0047-2891"[IS] OR "1573-6601"[IS] OR "0959-9851"[IS] OR "1619-1560"[IS] OR "1742-464X"[IS] OR "1742-4658"[IS] OR "1567-4223"[IS] OR "1873-7846"[IS] OR "0014-4886"[IS] OR "1090-2430"[IS] OR "0301-679X"[IS] OR "1879-2464"[IS] OR "0002-9599"[IS] OR "1945-452X"[IS] OR "0007-1234"[IS] OR "1469-2112"[IS] OR "N/A"[IS] OR "2057-1577"[IS] OR "1556-1607"[IS] OR "1556-1615"[IS] OR "1083-8791"[IS] OR "1523-6536"[IS] OR "1520-7439"[IS] OR "1573-8981"[IS] OR "0021-8790"[IS] OR "1365-2656"[IS] OR "1478-6354"[IS] OR "1478-6362"[IS] OR "0749-5978"[IS] OR "1095-9920"[IS] OR "0946-2716"[IS] OR "1432-1440"[IS] OR "1750-2640"[IS] OR "1750-2659"[IS] OR "1740-5025"[IS] OR "1470-7330"[IS] OR "2041-3866"[IS] OR "2041-3874"[IS] OR "0025-5408"[IS] OR "1873-4227"[IS] OR "2516-0230"[IS] OR "2516-0230"[IS] OR "0004-8674"[IS] OR "1440-1614"[IS] OR "0304-324X"[IS] OR "1423-0003"[IS] OR "0194-5998"[IS] OR "1097-6817"[IS] OR "0939-6411"[IS] OR "1873-3441"[IS] OR "1471-2598"[IS] OR "1744-7682"[IS] OR "0025-1747"[IS] OR "1758-6070"[IS] OR "1441-3523"[IS] OR "1839-2083"[IS] OR "2052-2525"[IS] OR "2052-2525"[IS] OR "0898-2104"[IS] OR "1532-2394"[IS] OR "0141-0296"[IS] OR "1873-7323"[IS] OR "0022-5096"[IS] OR "1873-4782"[IS] OR "1935-5130"[IS] OR "1935-5149"[IS] OR "N/A"[IS] OR "2218-1989"[IS] OR "1047-840X"[IS] OR "1532-7965"[IS] OR "0026-4806"[IS] OR "1827-1669"[IS] OR "1740-1445"[IS] OR "1873-6807"[IS] OR "1042-1629"[IS] OR "1556-6501"[IS] OR "2373-8227"[IS] OR "N/A"[IS] OR "0312-5963"[IS] OR "1179-1926"[IS] OR "0094-8276"[IS] OR "1944-8007"[IS] OR "1757-9880"[IS] OR "1757-9899"[IS] OR "0885-3924"[IS] OR "1873-6513"[IS] OR "0040-5752"[IS] OR "1432-2242"[IS] OR "0278-6915"[IS] OR "1873-6351"[IS] OR "2194-7511"[IS] OR "2194-7511"[IS] OR "1861-1125"[IS] OR "1861-1133"[IS] OR "1098-7339"[IS] OR "1532-8651"[IS] OR "1528-3542"[IS] OR "1931-1516"[IS] OR "N/A"[IS] OR "2666-1659"[IS] OR "N/A"[IS] OR "2304-8158"[IS] OR "1360-8185"[IS] OR "1573-675X"[IS] OR "0175-7598"[IS] OR "1432-0614"[IS] OR "1001-604X"[IS] OR "1614-7065"[IS] OR "0278-6133"[IS] OR "1930-7810"[IS] OR "1935-7893"[IS] OR "1938-744X"[IS] OR "0016-8505"[IS] OR "1751-7656"[IS] OR "1673-1581"[IS] OR "1862-1783"[IS] OR "0307-0565"[IS] OR "1476-5497"[IS] OR "1040-2608"[IS] OR "1040-2608"[IS] OR "1208-6053"[IS] OR "1181-8700"[IS] OR "0014-3057"[IS] OR "1873-1945"[IS] OR "0022-3042"[IS] OR "1471-4159"[IS] OR "2296-2646"[IS] OR "2296-2646"[IS] OR "1045-2354"[IS] OR "1095-9955"[IS] OR "N/A"[IS] OR "2161-3311"[IS] OR "N/A"[IS] OR "2504-446X"[IS] OR "0015-8208"[IS] OR "1521-3978"[IS] OR "0272-8842"[IS] OR "1873-3956"[IS] OR "0962-8452"[IS] OR "1471-2954"[IS] OR "0916-9636"[IS] OR "1348-4214"[IS] OR "0883-5993"[IS] OR "1536-0237"[IS] OR "1569-1993"[IS] OR "1873-5010"[IS] OR "1526-0542"[IS] OR "1526-0550"[IS] OR "1075-4253"[IS] OR "1873-0620"[IS] OR "0170-8406"[IS] OR "1741-3044"[IS] OR "0004-637X"[IS] OR "1538-4357"[IS] OR "1877-7171"[IS] OR "1877-718X"[IS] OR "1464-6722"[IS] OR "1364-3703"[IS] OR "0927-7757"[IS] OR "1873-4359"[IS] OR "1939-4551"[IS] OR "1939-4551"[IS] OR "0065-2164"[IS] OR "N/A"[IS] OR "1002-0160"[IS] OR "2210-5107"[IS] OR "N/A"[IS] OR "2056-7936"[IS] OR "1461-6696"[IS] OR "1469-8307"[IS] OR "0929-1393"[IS] OR "1873-0272"[IS] OR "1477-9560"[IS] OR "1477-9560"[IS] OR "0742-6046"[IS] OR "1520-6793"[IS] OR "N/A"[IS] OR "1757-2215"[IS] OR "0023-6837"[IS] OR "1530-0307"[IS] OR "1088-8691"[IS] OR "1532-480X"[IS] OR "1959-0318"[IS] OR "1876-0988"[IS] OR "0158-7919"[IS] OR "1475-0198"[IS] OR "0047-6374"[IS] OR "1872-6216"[IS] OR "0096-3445"[IS] OR "1939-2222"[IS] OR "1867-3880"[IS] OR "1867-3899"[IS] OR "1548-3568"[IS] OR "1548-3576"[IS] OR "1389-1286"[IS] OR "1872-7069"[IS] OR "0355-3140"[IS] OR "1795-990X"[IS] OR "1674-7313"[IS] OR "1869-1897"[IS] OR "0004-6256"[IS] OR "1538-3881"[IS] OR "1047-7047"[IS] OR "1526-5536"[IS] OR "0142-1123"[IS] OR "1879-3452"[IS] OR "0002-9394"[IS] OR "1879-1891"[IS] OR "0213-4853"[IS] OR "1578-1968"[IS] OR "1099-5129"[IS] OR "1532-2092"[IS] OR "0015-7120"[IS] OR "N/A"[IS] OR "N/A"[IS] OR "1083-351X"[IS] OR "1758-8340"[IS] OR "1758-8359"[IS] OR "1076-6332"[IS] OR "1878-4046"[IS] OR "2190-4707"[IS] OR "2190-4715"[IS] OR "0892-6875"[IS] OR "0892-6875"[IS] OR "N/A"[IS] OR "2473-4284"[IS] OR "1751-570X"[IS] OR "1878-7460"[IS] OR "1876-1070"[IS] OR "1876-1089"[IS] OR "1462-2912"[IS] OR "1462-2920"[IS] OR "0260-3594"[IS] OR "1548-9574"[IS] OR "1828-0447"[IS] OR "1970-9366"[IS] OR "1364-8152"[IS] OR "1873-6726"[IS] OR "0031-5826"[IS] OR "1744-6570"[IS] OR "0391-4097"[IS] OR "1720-8386"[IS] OR "N/A"[IS] OR "1756-8935"[IS] OR "0905-6157"[IS] OR "1399-3038"[IS] OR "N/A"[IS] OR "2352-5541"[IS] OR "2168-6777"[IS] OR "2168-6785"[IS] OR "2190-4979"[IS] OR "2190-4987"[IS] OR "1270-9638"[IS] OR "1626-3219"[IS] OR "0094-1190"[IS] OR "1095-9068"[IS] OR "2052-4129"[IS] OR "2052-4129"[IS] OR "1365-7852"[IS] OR "1476-5608"[IS] OR "1060-3743"[IS] OR "1873-1422"[IS] OR "1876-1623"[IS] OR "N/A"[IS] OR "0957-4093"[IS] OR "1758-6550"[IS] OR "0172-4614"[IS] OR "1438-8782"[IS] OR "1388-2481"[IS] OR "1873-1902"[IS] OR "0981-9428"[IS] OR "1873-2690"[IS] OR "N/A"[IS] OR "2214-5818"[IS] OR "0020-1669"[IS] OR "1520-510X"[IS] OR "1863-6683"[IS] OR "1863-6691"[IS] OR "1664-0640"[IS] OR "1664-0640"[IS] OR "0017-9310"[IS] OR "1879-2189"[IS] OR "0049-0172"[IS] OR "1532-866X"[IS] OR "1534-4827"[IS] OR "1539-0829"[IS] OR "2328-9503"[IS] OR "2328-9503"[IS] OR "1811-5209"[IS] OR "1811-5217"[IS] OR "2640-205X"[IS] OR "2640-2068"[IS] OR "0022-3506"[IS] OR "1467-6494"[IS] OR "0969-6997"[IS] OR "1873-

	2089"[IS] OR "2150-5594"[IS] OR "2150-5608"[IS] OR "0968-6673"[IS] OR "1468-0432"[IS] OR "0378-3839"[IS] OR "1872-7379"[IS] OR "2042-6976"[IS] OR "2042-6984"[IS] OR "0022-1767"[IS] OR "1550-6606"[IS] OR "0016-9013"[IS] OR "1758-5341"[IS] OR "1053-4296"[IS] OR "1532-9461"[IS] OR "0022-4537"[IS] OR "1540-4560"[IS] OR "1063-6536"[IS] OR "1558-0865"[IS] OR "1931-7573"[IS] OR "1556-276X"[IS] OR "0924-977X"[IS] OR "1873-7862"[IS] OR "0269-994X"[IS] OR "1464-0597"[IS] OR "0885-6125"[IS] OR "1573-0565"[IS] OR "0732-2399"[IS] OR "1526-548X"[IS] OR "N/A"[IS] OR "2296-665X"[IS] OR "2190-5487"[IS] OR "2190-5495"[IS] OR "0016-7606"[IS] OR "1943-2674"[IS] OR "1470-2118"[IS] OR "1473-4893"[IS] OR "0923-1811"[IS] OR "1873-569X"[IS] OR "0022-3085"[IS] OR "1933-0693"[IS] OR "2470-0010"[IS] OR "2470-0029"[IS] OR "1384-5810"[IS] OR "1573-756X"[IS] OR "2352-4677"[IS] OR "2352-4677"[IS] OR "0954-7894"[IS] OR "1365-2222"[IS] OR "0002-9173"[IS] OR "1943-7722"[IS] OR "2040-6207"[IS] OR "2040-6215"[IS] OR "0084-0173"[IS] OR "1938-5455"[IS] OR "1065-9471"[IS] OR "1097-0193"[IS] OR "0037-7791"[IS] OR "1533-8533"[IS] OR "2373-9878"[IS] OR "2373-9878"[IS] OR "N/A"[IS] OR "1758-5996"[IS] OR "1877-0657"[IS] OR "1877-0665"[IS] OR "1615-9853"[IS] OR "1615-9861"[IS] OR "0251-5350"[IS] OR "1423-0208"[IS] OR "1471-7727"[IS] OR "1873-7919"[IS] OR "0093-7754"[IS] OR "1532-8708"[IS] OR "0041-1337"[IS] OR "1534-6080"[IS] OR "2049-4394"[IS] OR "2049-4394"[IS] OR "0889-3365"[IS] OR "1537-2642"[IS] OR "1526-5900"[IS] OR "1528-8447"[IS] OR "0894-8755"[IS] OR "1520-0442"[IS] OR "0277-9536"[IS] OR "1873-5347"[IS] OR "1007-9327"[IS] OR "2219-2840"[IS] OR "0305-1838"[IS] OR "1365-2907"[IS] OR "0045-7949"[IS] OR "1879-2243"[IS] OR "1535-3893"[IS] OR "1535-3907"[IS] OR "1933-2874"[IS] OR "1876-4789"[IS] OR "1759-9954"[IS] OR "1759-9962"[IS] OR "1543-8384"[IS] OR "1543-8392"[IS] OR "1933-9747"[IS] OR "1933-9747"[IS] OR "0002-9262"[IS] OR "1476-6256"[IS] OR "0168-9452"[IS] OR "1873-2259"[IS] OR "0264-8172"[IS] OR "1873-4073"[IS] OR "0309-1708"[IS] OR "1872-9657"[IS] OR "0885-4122"[IS] OR "1552-6593"[IS] OR "1072-4133"[IS] OR "1099-0968"[IS] OR "N/A"[IS] OR "2214-7829"[IS] OR "2329-7662"[IS] OR "2329-7670"[IS] OR "1011-8934"[IS] OR "1598-6357"[IS] OR "1018-4813"[IS] OR "1476-5438"[IS] OR "1018-8827"[IS] OR "1435-165X"[IS] OR "N/A"[IS] OR "2072-4292"[IS] OR "0785-3890"[IS] OR "1365-2060"[IS] OR "2095-3941"[IS] OR "2095-3941"[IS] OR "0315-162X"[IS] OR "1499-2752"[IS] OR "1366-9877"[IS] OR "1466-4461"[IS] OR "1536-9323"[IS] OR "1558-3457"[IS] OR "1398-5647"[IS] OR "1399-5618"[IS] OR "1137-6821"[IS] OR "2386-5857"[IS] OR "2050-0513"[IS] OR "2050-0521"[IS] OR "1545-598X"[IS] OR "1558-0571"[IS] OR "1525-1578"[IS] OR "1943-7811"[IS] OR "0924-6495"[IS] OR "1572-8641"[IS] OR "0892-9912"[IS] OR "1573-7047"[IS] OR "0307-904X"[IS] OR "1872-8480"[IS] OR "0031-3025"[IS] OR "1465-3931"[IS] OR "2050-7887"[IS] OR "2050-7895"[IS] OR "0306-6150"[IS] OR "1743-9361"[IS] OR "0360-3989"[IS] OR "1468-2958"[IS] OR "1059-0161"[IS] OR "1573-7756"[IS] OR "0018-9456"[IS] OR "1557-9662"[IS] OR "2165-0373"[IS] OR "2165-0381"[IS] OR "1090-5138"[IS] OR "1879-0607"[IS] OR "2096-2754"[IS] OR "2467-9674"[IS] OR "1757-4749"[IS] OR "1757-4749"[IS] OR "1755-4365"[IS] OR "1878-0067"[IS] OR "0065-2776"[IS] OR "1557-8445"[IS] OR "0706-7437"[IS] OR "1497-0015"[IS] OR "0005-7967"[IS] OR "1873-622X"[IS] OR "0019-5456"[IS] OR "0973-7693"[IS] OR "0718-1876"[IS] OR "0718-1876"[IS] OR "2212-4292"[IS] OR "2212-4306"[IS] OR "0954-5794"[IS] OR "1469-2198"[IS] OR "2311-2638"[IS] OR "2311-2638"[IS] OR "1948-5506"[IS] OR "1948-5514"[IS] OR "2397-334X"[IS] OR "2397-334X"[IS] OR "2211-2855"[IS] OR "2211-3282"[IS] OR "0033-5533"[IS] OR "1531-4650"[IS] OR "0085-2538"[IS] OR "1523-1755"[IS] OR "1748-0132"[IS] OR "1878-044X"[IS] OR "0268-4012"[IS] OR "1873-4707"[IS] OR "0031-6997"[IS] OR "1521-0081"[IS] OR "N/A"[IS] OR "1750-1326"[IS] OR "2374-7943"[IS] OR "2374-7951"[IS] OR "0896-6273"[IS] OR "1097-4199"[IS] OR "0306-3674"[IS] OR "1473-0480"[IS] OR "1545-9993"[IS] OR "1545-9985"[IS] OR "0966-842X"[IS] OR "1878-4380"[IS] OR "1936-0851"[IS] OR "1936-086X"[IS] OR "0141-0768"[IS] OR "1758-1095"[IS] OR "1543-5938"[IS] OR "1545-2050"[IS] OR "1474-760X"[IS] OR "1474-760X"[IS] OR "0169-409X"[IS] OR "1872-8294"[IS] OR "0034-4885"[IS] OR "1361-6633"[IS] OR "0163-8998"[IS] OR "1545-4134"[IS] OR "N/A"[IS] OR "2041-1723"[IS] OR "0734-9750"[IS] OR "1873-1899"[IS] OR "1359-6101"[IS] OR "1879-0305"[IS] OR "0165-6147"[IS] OR "1873-3735"[IS] OR "0022-1007"[IS] OR "1540-9538"[IS] OR "1566-2535"[IS] OR "1872-6305"[IS] OR "N/A"[IS] OR "2198-3844"[IS] OR "0065-2156"[IS] OR "N/A"[IS] OR "1568-9972"[IS] OR "1873-0183"[IS] OR "2055-026X"[IS] OR "2055-0278"[IS] OR "1388-9842"[IS] OR "1879-0844"[IS] OR "N/A"[IS] OR "2001-3078"[IS] OR "0270-9139"[IS] OR "1527-3350"[IS] OR "1355-4786"[IS] OR "1460-2369"[IS] OR "1389-5567"[IS] OR "1873-2739"[IS] OR "0149-5992"[IS] OR "1935-5548"[IS] OR "N/A"[IS] OR "2589-5370"[IS] OR "1754-2189"[IS] OR "1750-2799"[IS] OR "1044-579X"[IS] OR "1096-3650"[IS] OR "2666-3791"[IS] OR "2666-3791"[IS] OR "0166-2236"[IS] OR "1878-108X"[IS] OR "N/A"[IS] OR "2452-199X"[IS] OR "0820-3946"[IS] OR "1488-2329"[IS] OR "2049-2618"[IS] OR "2049-2618"[IS] OR "1433-7851"[IS] OR "1521-3773"[IS] OR "1364-0321"[IS] OR "1879-0690"[IS] OR "1385-8947"[IS] OR "1873-3212"[IS] OR "2168-6254"[IS] OR "2168-6262"[IS] OR "1552-5260"[IS] OR "1552-5279"[IS] OR "1941-1405"[IS] OR "1941-0611"[IS] OR "2077-7000"[IS] OR "2077-7000"[IS] OR "1089-778X"[IS] OR "1941-0026"[IS] OR "0146-2806"[IS] OR "1535-6280"[IS] OR "0362-1642"[IS] OR "1545-4304"[IS] OR "1759-4812"[IS] OR "1759-4820"[IS] OR "0002-7863"[IS] OR "1520-5126"[IS] OR "0003-066X"[IS] OR "1935-990X"[IS] OR "0098-2997"[IS] OR

	"1872-9452"[IS] OR "0084-6597"[IS] OR "1545-4495"[IS] OR "0066-4227"[IS] OR "1545-3251"[IS] OR "1552-4450"[IS] OR "1552-4469"[IS] OR "1088-8683"[IS] OR "1532-7957"[IS] OR "1080-6040"[IS] OR "1080-6059"[IS] OR "2352-3018"[IS] OR "2352-3018"[IS] OR "1936-878X"[IS] OR "1876-7591"[IS] OR "0066-4219"[IS] OR "1545-326X"[IS] OR "1538-7933"[IS] OR "1538-7836"[IS] OR "0924-2244"[IS] OR "1879-3053"[IS] OR "0001-6322"[IS] OR "1432-0533"[IS] OR "1226-4601"[IS] OR "2055-7124"[IS] OR "2515-2459"[IS] OR "2515-2467"[IS] OR "1541-4337"[IS] OR "1541-4337"[IS] OR "0950-6608"[IS] OR "1743-2804"[IS] OR "2051-6347"[IS] OR "2051-6355"[IS] OR "2397-3366"[IS] OR "2397-3366"[IS] OR "0962-4929"[IS] OR "1474-0508"[IS] OR "0147-006X"[IS] OR "1545-4126"[IS] OR "0190-9622"[IS] OR "1097-6787"[IS] OR "2326-5191"[IS] OR "2326-5205"[IS] OR "0924-8579"[IS] OR "1872-7913"[IS] OR "2366-9608"[IS] OR "2366-9608"[IS] OR "0022-2429"[IS] OR "1547-7185"[IS] OR "2398-6352"[IS] OR "2398-6352"[IS] OR "1674-800X"[IS] OR "1674-8018"[IS] OR "0142-9612"[IS] OR "1878-5905"[IS] OR "N/A"[IS] OR "2523-3548"[IS] OR "1471-4914"[IS] OR "1471-499X"[IS] OR "1756-994X"[IS] OR "1756-994X"[IS] OR "0006-8950"[IS] OR "1460-2156"[IS] OR "1053-5888"[IS] OR "1558-0792"[IS] OR "0001-8686"[IS] OR "1873-3727"[IS] OR "0168-6445"[IS] OR "1574-6976"[IS] OR "1613-6810"[IS] OR "1613-6829"[IS] OR "0265-0568"[IS] OR "1460-4752"[IS] OR "1046-6673"[IS] OR "1533-3450"[IS] OR "2375-2548"[IS] OR "2375-2548"[IS] OR "2590-3330"[IS] OR "2590-3322"[IS] OR "0013-0095"[IS] OR "1944-8287"[IS] OR "0018-9219"[IS] OR "1558-2256"[IS] OR "0165-9936"[IS] OR "1879-3142"[IS] OR "0092-0703"[IS] OR "1552-7824"[IS] OR "2211-3835"[IS] OR "2211-3843"[IS] OR "1941-1413"[IS] OR "1941-1421"[IS] OR "0105-4538"[IS] OR "1398-9995"[IS] OR "0963-8687"[IS] OR "1873-1198"[IS] OR "1526-5161"[IS] OR "1536-0075"[IS] OR "0163-7827"[IS] OR "1873-2194"[IS] OR "2369-2960"[IS] OR "2369-2960"[IS] OR "2213-6657"[IS] OR "2213-6665"[IS] OR "1558-3724"[IS] OR "1558-3716"[IS] OR "2050-7488"[IS] OR "2050-7496"[IS] OR "0896-8411"[IS] OR "1095-9157"[IS] OR "1386-6532"[IS] OR "1873-5967"[IS] OR "2160-3308"[IS] OR "2160-3308"[IS] OR "N/A"[IS] OR "2057-3995"[IS] OR "1464-7931"[IS] OR "1469-185X"[IS] OR "1543-592X"[IS] OR "1545-2069"[IS] OR "0360-0300"[IS] OR "1557-7341"[IS] OR "0091-6749"[IS] OR "1097-6825"[IS] OR "1569-1705"[IS] OR "1572-9826"[IS] OR "0161-6420"[IS] OR "1549-4713"[IS] OR "0968-0004"[IS] OR "1362-4326"[IS] OR "2327-056X"[IS] OR "2327-0578"[IS] OR "1757-4676"[IS] OR "1757-4684"[IS] OR "2162-237X"[IS] OR "2162-2388"[IS] OR "0304-3894"[IS] OR "1873-3336"[IS] OR "0001-0782"[IS] OR "1557-7317"[IS] OR "0004-3702"[IS] OR "1872-7921"[IS] OR "1460-2725"[IS] OR "1460-2393"[IS] OR "2095-1779"[IS] OR "2214-0883"[IS] OR "0261-4189"[IS] OR "1460-2075"[IS] OR "1467-5463"[IS] OR "1477-4054"[IS] OR "1949-3045"[IS] OR "1949-3045"[IS] OR "1531-7331"[IS] OR "1545-4118"[IS] OR "0026-0495"[IS] OR "1532-8600"[IS] OR "2473-2397"[IS] OR "2168-6831"[IS] OR "1876-2018"[IS] OR "1876-2026"[IS] OR "0363-7425"[IS] OR "1930-3807"[IS] OR "0034-4257"[IS] OR "1879-0704"[IS] OR "0042-9686"[IS] OR "1564-0604"[IS] OR "1361-8415"[IS] OR "1361-8423"[IS] OR "0066-4197"[IS] OR "1545-2948"[IS] OR "1078-0432"[IS] OR "1557-3265"[IS] OR "0003-4932"[IS] OR "1528-1140"[IS] OR "1540-9295"[IS] OR "1540-9309"[IS] OR "0921-3449"[IS] OR "1879-0658"[IS] OR "0887-6185"[IS] OR "1873-7897"[IS] OR "2155-5435"[IS] OR "2155-5435"[IS] OR "2590-1168"[IS] OR "2590-1168"[IS] OR "0022-3050"[IS] OR "1468-330X"[IS] OR "2214-1588"[IS] OR "2214-1588"[IS] OR "1610-3653"[IS] OR "1610-3661"[IS] OR "1556-6072"[IS] OR "1556-6080"[IS] OR "0305-7372"[IS] OR "1532-1967"[IS] OR "0161-4940"[IS] OR "1520-5703"[IS] OR "2095-4956"[IS] OR "2095-4956"[IS] OR "N/A"[IS] OR "2577-5421"[IS] OR "1542-3565"[IS] OR "1542-7714"[IS] OR "1053-2498"[IS] OR "1557-3117"[IS] OR "0034-6543"[IS] OR "1935-1046"[IS] OR "1362-0347"[IS] OR "1468-960X"[IS] OR "0149-2063"[IS] OR "1557-1211"[IS] OR "N/A"[IS] OR "2575-0356"[IS] OR "1359-4184"[IS] OR "1476-5578"[IS] OR "0167-5729"[IS] OR "1879-274X"[IS] OR "1534-5807"[IS] OR "1878-1551"[IS] OR "1743-9191"[IS] OR "1743-9159"[IS] OR "0163-7258"[IS] OR "1879-016X"[IS] OR "0043-1354"[IS] OR "1879-2448"[IS] OR "1554-8627"[IS] OR "1554-8635"[IS] OR "0920-5691"[IS] OR "1573-1405"[IS] OR "2588-8420"[IS] OR "2588-8420"[IS] OR "2095-4700"[IS] OR "2095-6231"[IS] OR "2574-3805"[IS] OR "2574-3805"[IS] OR "0160-4120"[IS] OR "1873-6750"[IS] OR "2165-8102"[IS] OR "2165-8110"[IS] OR "2472-3428"[IS] OR "2472-3428"[IS] OR "0008-5472"[IS] OR "1538-7445"[IS] OR "1198-743X"[IS] OR "1469-0691"[IS] OR "1554-0669"[IS] OR "1554-0677"[IS] OR "0361-8609"[IS] OR "1096-8652"[IS] OR "1467-7644"[IS] OR "1467-7652"[IS] OR "1354-1013"[IS] OR "1365-2486"[IS] OR "0883-9026"[IS] OR "1873-2003"[IS] OR "0890-8567"[IS] OR "1527-5418"[IS] OR "0008-6363"[IS] OR "1755-3245"[IS] OR "0733-8716"[IS] OR "1558-0008"[IS] OR "2095-2546"[IS] OR "2213-2961"[IS] OR "0954-6820"[IS] OR "1365-2796"[IS] OR "1744-4292"[IS] OR "1744-4292"[IS] OR "1092-2172"[IS] OR "1098-5557"[IS] OR "1522-8517"[IS] OR "1523-5866"[IS] OR "2524-7921"[IS] OR "2524-793X"[IS] OR "0253-9837"[IS] OR "1872-2067"[IS] OR "1525-0016"[IS] OR "1525-0024"[IS] OR "0022-0515"[IS] OR "2328-8175"[IS] OR "0003-2409"[IS] OR "1365-2044"[IS] OR "0890-9369"[IS] OR "1549-5477"[IS] OR "0887-6924"[IS] OR "1476-5551"[IS] OR "0261-5177"[IS] OR "1879-3193"[IS] OR "0148-396X"[IS] OR "1524-4040"[IS] OR "1107-3756"[IS] OR "1791-244X"[IS] OR "1973-9087"[IS] OR "1973-9095"[IS] OR "0017-8748"[IS] OR "1526-4610"[IS] OR "1476-072X"[IS] OR "1476-072X"[IS] OR "1461-4448"[IS] OR "1461-7315"[IS] OR "N/A"[IS] OR "2511-9044"[IS] OR "0735-0015"[IS] OR "1537-2707"[IS] OR "0045-2068"[IS] OR "1090-

	2120"[IS] OR "0002-838X"[IS] OR "1532-0650"[IS] OR "0090-8258"[IS] OR "1095-6859"[IS] OR "0026-265X"[IS] OR "1095-9149"[IS] OR "1086-0266"[IS] OR "1552-7417"[IS] OR "0962-1105"[IS] OR "1365-2869"[IS] OR "1582-1838"[IS] OR "1582-4934"[IS] OR "1939-1390"[IS] OR "1941-1197"[IS] OR "0733-9364"[IS] OR "1943-7862"[IS] OR "2405-6383"[IS] OR "2405-6545"[IS] OR "1279-7707"[IS] OR "1760-4788"[IS] OR "1875-5100"[IS] OR "2212-3865"[IS] OR "0363-6143"[IS] OR "1522-1563"[IS] OR "2162-2337"[IS] OR "2162-2345"[IS] OR "1465-4644"[IS] OR "1468-4357"[IS] OR "2211-9264"[IS] OR "2211-9264"[IS] OR "2303-9027"[IS] OR "2226-7190"[IS] OR "0971-5916"[IS] OR "0971-5916"[IS] OR "1568-4539"[IS] OR "1573-2908"[IS] OR "0028-3908"[IS] OR "1873-7064"[IS] OR "1618-7598"[IS] OR "1618-7601"[IS] OR "1673-7067"[IS] OR "1995-8218"[IS] OR "0007-1013"[IS] OR "1467-8535"[IS] OR "0031-0808"[IS] OR "1827-1898"[IS] OR "1864-3361"[IS] OR "1864-3361"[IS] OR "2212-0963"[IS] OR "2212-0963"[IS] OR "1867-1306"[IS] OR "1867-1314"[IS] OR "1297-319X"[IS] OR "1778-7254"[IS] OR "0278-7407"[IS] OR "1944-9194"[IS] OR "1387-3326"[IS] OR "1572-9419"[IS] OR "1471-2229"[IS] OR "1471-2229"[IS] OR "0948-3349"[IS] OR "1614-7502"[IS] OR "2214-5745"[IS] OR "2214-5753"[IS] OR "2051-3933"[IS] OR "2051-3933"[IS] OR "0022-3956"[IS] OR "1879-1379"[IS] OR "2161-5063"[IS] OR "N/A"[IS] OR "1061-0421"[IS] OR "2054-1643"[IS] OR "1948-0210"[IS] OR "1948-0210"[IS] OR "N/A"[IS] OR "2296-7745"[IS] OR "0887-6045"[IS] OR "0887-6045"[IS] OR "0070-2153"[IS] OR "N/A"[IS] OR "0023-8333"[IS] OR "1467-9922"[IS] OR "1932-104X"[IS] OR "1932-1031"[IS] OR "2049-8470"[IS] OR "2049-8489"[IS] OR "1862-8516"[IS] OR "1862-8508"[IS] OR "0739-1102"[IS] OR "1538-0254"[IS] OR "0035-8711"[IS] OR "1365-2966"[IS] OR "2048-7193"[IS] OR "2048-7207"[IS] OR "0021-9533"[IS] OR "1477-9137"[IS] OR "2212-2672"[IS] OR "2212-2680"[IS] OR "1932-4545"[IS] OR "1940-9990"[IS] OR "1610-0379"[IS] OR "1610-0387"[IS] OR "1070-9932"[IS] OR "1558-223X"[IS] OR "0114-5916"[IS] OR "1179-1942"[IS] OR "1388-1981"[IS] OR "1879-2618"[IS] OR "0003-2654"[IS] OR "1364-5528"[IS] OR "1077-2626"[IS] OR "1941-0506"[IS] OR "1756-4646"[IS] OR "2214-9414"[IS] OR "2079-6382"[IS] OR "2079-6382"[IS] OR "0929-5305"[IS] OR "1573-742X"[IS] OR "1069-6563"[IS] OR "1553-2712"[IS] OR "0261-9768"[IS] OR "1469-5928"[IS] OR "0266-352X"[IS] OR "1873-7633"[IS] OR "0032-3772"[IS] OR "1897-9483"[IS] OR "2169-897X"[IS] OR "2169-8996"[IS] OR "N/A"[IS] OR "1424-8247"[IS] OR "1871-403X"[IS] OR "1878-0318"[IS] OR "2724-6051"[IS] OR "2724-6442"[IS] OR "2211-8837"[IS] OR "N/A"[IS] OR "N/A"[IS] OR "1743-0003"[IS] OR "0026-4598"[IS] OR "1432-1866"[IS] OR "0197-3975"[IS] OR "1873-5428"[IS] OR "0141-3910"[IS] OR "1873-2321"[IS] OR "1674-7348"[IS] OR "1869-1927"[IS] OR "0166-445X"[IS] OR "1879-1514"[IS] OR "0278-5846"[IS] OR "1878-4216"[IS] OR "1759-0914"[IS] OR "1759-0914"[IS] OR "2058-9883"[IS] OR "2058-9883"[IS] OR "1018-8665"[IS] OR "1421-9832"[IS] OR "0014-2999"[IS] OR "1879-0712"[IS] OR "0378-8741"[IS] OR "1872-7573"[IS] OR "0018-9316"[IS] OR "1557-9611"[IS] OR "1000-0747"[IS] OR "N/A"[IS] OR "0944-1344"[IS] OR "1614-7499"[IS] OR "N/A"[IS] OR "2471-2906"[IS] OR "1085-9489"[IS] OR "1529-8027"[IS] OR "0001-4826"[IS] OR "1558-7967"[IS] OR "1083-4389"[IS] OR "1523-5378"[IS] OR "0065-2504"[IS] OR "2163-582X"[IS] OR "0961-5539"[IS] OR "1758-6585"[IS] OR "0309-0566"[IS] OR "1758-7123"[IS] OR "0022-1465"[IS] OR "2150-6000"[IS] OR "2151-464X"[IS] OR "2151-4658"[IS] OR "1532-4435"[IS] OR "N/A"[IS] OR "1752-1378"[IS] OR "1752-1386"[IS] OR "0268-3369"[IS] OR "1476-5365"[IS] OR "0165-0009"[IS] OR "1573-1480"[IS] OR "1538-4101"[IS] OR "1551-4005"[IS] OR "0032-1052"[IS] OR "1529-4242"[IS] OR "N/A"[IS] OR "2079-7737"[IS] OR "0098-3004"[IS] OR "1873-7803"[IS] OR "0009-2797"[IS] OR "1872-7786"[IS] OR "0920-4105"[IS] OR "1873-4715"[IS] OR "2005-6419"[IS] OR "2005-7563"[IS] OR "0143-2885"[IS] OR "1365-2591"[IS] OR "2056-4724"[IS] OR "2056-4724"[IS] OR "1590-8658"[IS] OR "1878-3562"[IS] OR "1617-9625"[IS] OR "N/A"[IS] OR "1544-1873"[IS] OR "1544-2241"[IS] OR "0305-0548"[IS] OR "1873-765X"[IS] OR "2157-1422"[IS] OR "2157-1422"[IS] OR "1753-4658"[IS] OR "1753-4666"[IS] OR "0743-0167"[IS] OR "1873-1392"[IS] OR "2215-0986"[IS] OR "2215-0986"[IS] OR "1047-7039"[IS] OR "N/A"[IS] OR "N/A"[IS] OR "1662-453X"[IS] OR "1365-8816"[IS] OR "1362-3087"[IS] OR "2325-8292"[IS] OR "2325-8306"[IS] OR "1063-0732"[IS] OR "1466-1853"[IS] OR "0268-3962"[IS] OR "1466-4437"[IS] OR "2055-6640"[IS] OR "2055-6659"[IS] OR "0964-4016"[IS] OR "1743-8934"[IS] OR "N/A"[IS] OR "2049-3002"[IS] OR "N/A"[IS] OR "2050-6201"[IS] OR "2095-0462"[IS] OR "2095-0470"[IS] OR "1010-6030"[IS] OR "1873-2666"[IS] OR "0022-1317"[IS] OR "1465-2099"[IS] OR "0899-1987"[IS] OR "1098-2744"[IS] OR "2212-1447"[IS] OR "2212-1455"[IS] OR "N/A"[IS] OR "2056-6646"[IS] OR "0044-8486"[IS] OR "1873-5622"[IS] OR "0028-3835"[IS] OR "1423-0194"[IS] OR "0197-4580"[IS] OR "1558-1497"[IS] OR "1687-966X"[IS] OR "1687-9678"[IS] OR "0263-2241"[IS] OR "1873-412X"[IS] OR "0363-6135"[IS] OR "1522-1539"[IS] OR "1744-666X"[IS] OR "1744-8409"[IS] OR "N/A"[IS] OR "2311-5637"[IS] OR "0143-7208"[IS] OR "1873-3743"[IS] OR "0964-6906"[IS] OR "1460-2083"[IS] OR "2040-2503"[IS] OR "2040-2511"[IS] OR "0009-2673"[IS] OR "1348-0634"[IS] OR "0032-2687"[IS] OR "1573-0891"[IS] OR "1053-1807"[IS] OR "1522-2586"[IS] OR "0963-1798"[IS] OR "2044-8325"[IS] OR "1469-0292"[IS] OR "1878-5476"[IS] OR "1468-2702"[IS] OR "1468-2710"[IS] OR "0928-0987"[IS] OR "1879-0720"[IS] OR "1467-0895"[IS] OR "1873-4723"[IS] OR "0378-5122"[IS] OR "1873-4111"[IS] OR "0929-1199"[IS] OR "1872-6313"[IS] OR "1661-7800"[IS] OR "1661-7819"[IS] OR "0167-4048"[IS] OR "1872-6208"[IS] OR "0960-3085"[IS] OR "1744-3571"[IS] OR "0934-9723"[IS] OR

	"1435-4373"[IS] OR "0003-4975"[IS] OR "1552-6259"[IS] OR "0941-0643"[IS] OR "1433-3058"[IS] OR "1098-3015"[IS] OR "1524-4733"[IS] OR "1532-3382"[IS] OR "1532-3390"[IS] OR "1445-8330"[IS] OR "1447-0349"[IS] OR "1661-8556"[IS] OR "1661-8564"[IS] OR "0999-193X"[IS] OR "1297-9686"[IS] OR "0002-8703"[IS] OR "1097-6744"[IS] OR "N/A"[IS] OR "2352-4588"[IS] OR "2092-7355"[IS] OR "2092-7363"[IS] OR "0925-4692"[IS] OR "1568-5608"[IS] OR "1726-4170"[IS] OR "1726-4189"[IS] OR "N/A"[IS] OR "2212-6864"[IS] OR "2468-8231"[IS] OR "2468-8231"[IS] OR "0022-0167"[IS] OR "1939-2168"[IS] OR "1179-1314"[IS] OR "1179-1314"[IS] OR "0361-0128"[IS] OR "1554-0774"[IS] OR "1538-6473"[IS] OR "1745-9133"[IS] OR "0265-2323"[IS] OR "1758-5937"[IS] OR "0031-9317"[IS] OR "1399-3054"[IS] OR "1527-2729"[IS] OR "1534-6277"[IS] OR "1138-7548"[IS] OR "1877-8755"[IS] OR "0883-0185"[IS] OR "1563-5244"[IS] OR "1537-4416"[IS] OR "1537-4424"[IS] OR "1746-8094"[IS] OR "1746-8108"[IS] OR "N/A"[IS] OR "2072-6651"[IS] OR "0953-8178"[IS] OR "1460-2377"[IS] OR "0937-941X"[IS] OR "1433-2965"[IS] OR "1939-5078"[IS] OR "1939-5086"[IS] OR "0944-5013"[IS] OR "1618-0623"[IS] OR "0270-7306"[IS] OR "1098-5549"[IS] OR "2225-0719"[IS] OR "2310-8819"[IS] OR "2044-5040"[IS] OR "2044-5040"[IS] OR "1773-2247"[IS] OR "2588-8943"[IS] OR "0954-349X"[IS] OR "1873-6017"[IS] OR "1572-3097"[IS] OR "1573-692X"[IS] OR "1741-3842"[IS] OR "1741-3850"[IS] OR "N/A"[IS] OR "2296-858X"[IS] OR "1591-8890"[IS] OR "1591-9528"[IS] OR "0962-8770"[IS] OR "1467-8608"[IS] OR "N/A"[IS] OR "2052-4439"[IS] OR "1369-118X"[IS] OR "1468-4462"[IS] OR "2363-8346"[IS] OR "2363-8354"[IS] OR "0013-7227"[IS] OR "1945-7170"[IS] OR "1876-2883"[IS] OR "1876-2891"[IS] OR "0047-2689"[IS] OR "1529-7845"[IS] OR "0140-3664"[IS] OR "1873-703X"[IS] OR "N/A"[IS] OR "2306-5354"[IS] OR "0921-2973"[IS] OR "1572-9761"[IS] OR "1741-2560"[IS] OR "1741-2552"[IS] OR "1745-5863"[IS] OR "1745-5871"[IS] OR "1976-6696"[IS] OR "1976-670X"[IS] OR "0305-7364"[IS] OR "1095-8290"[IS] OR "0038-9765"[IS] OR "N/A"[IS] OR "1598-2998"[IS] OR "2005-9256"[IS] OR "1869-4101"[IS] OR "1869-4101"[IS] OR "2327-4697"[IS] OR "2327-4697"[IS] OR "1530-5627"[IS] OR "1556-3669"[IS] OR "N/A"[IS] OR "2379-5042"[IS] OR "2410-339X"[IS] OR "2410-3403"[IS] OR "1606-5131"[IS] OR "1605-8127"[IS] OR "0905-7161"[IS] OR "1600-0501"[IS] OR "2274-5807"[IS] OR "2426-0266"[IS] OR "0947-6539"[IS] OR "1521-3765"[IS] OR "0924-669X"[IS] OR "1573-7497"[IS] OR "0024-3590"[IS] OR "1939-5590"[IS] OR "1055-7903"[IS] OR "1095-9513"[IS] OR "2047-2501"[IS] OR "2047-2501"[IS] OR "0195-6663"[IS] OR "1095-8304"[IS] OR "1061-186X"[IS] OR "1029-2330"[IS] OR "0167-4889"[IS] OR "1879-2596"[IS] OR "0960-0760"[IS] OR "1879-1220"[IS] OR "0570-4928"[IS] OR "1520-569X"[IS] OR "0047-2352"[IS] OR "1873-6203"[IS] OR "2379-8858"[IS] OR "2379-8904"[IS] OR "0149-0400"[IS] OR "1521-0588"[IS] OR "1022-1336"[IS] OR "1521-3927"[IS] OR "N/A"[IS] OR "2571-581X"[IS] OR "0099-2240"[IS] OR "1098-5336"[IS] OR "0272-4391"[IS] OR "1098-2299"[IS] OR "N/A"[IS] OR "2366-3987"[IS] OR "1537-5110"[IS] OR "1537-5129"[IS] OR "1542-6416"[IS] OR "1559-0275"[IS] OR "2096-6490"[IS] OR "2662-1746"[IS] OR "1016-9040"[IS] OR "1878-531X"[IS] OR "1359-0286"[IS] OR "1879-0348"[IS] OR "0160-7383"[IS] OR "1873-7722"[IS] OR "2095-8099"[IS] OR "2096-0026"[IS] OR "2090-1232"[IS] OR "2090-1224"[IS] OR "0006-3223"[IS] OR "1873-2402"[IS] OR "0002-0729"[IS] OR "1468-2834"[IS] OR "2096-2797"[IS] OR "2468-0257"[IS] OR "0027-8424"[IS] OR "1091-6490"[IS] OR "1536-1284"[IS] OR "1558-0687"[IS] OR "0025-729X"[IS] OR "1326-5377"[IS] OR "1021-7770"[IS] OR "1423-0127"[IS] OR "1540-1405"[IS] OR "1540-1413"[IS] OR "N/A"[IS] OR "1756-9966"[IS] OR "2327-0608"[IS] OR "2327-0616"[IS] OR "0956-5663"[IS] OR "1873-4235"[IS] OR "2213-1779"[IS] OR "2213-1787"[IS] OR "0001-8392"[IS] OR "1930-3815"[IS] OR "N/A"[IS] OR "2051-1426"[IS] OR "0003-1224"[IS] OR "1939-8271"[IS] OR "0393-2990"[IS] OR "1573-7284"[IS] OR "2235-1795"[IS] OR "1664-5553"[IS] OR "0146-6410"[IS] OR "1873-2224"[IS] OR "0300-8428"[IS] OR "1435-1803"[IS] OR "1936-1327"[IS] OR "1936-1335"[IS] OR "0198-6325"[IS] OR "1098-1128"[IS] OR "0302-282X"[IS] OR "1423-0224"[IS] OR "1530-6984"[IS] OR "1530-6992"[IS] OR "N/A"[IS] OR "2057-3960"[IS] OR "1063-6706"[IS] OR "1941-0034"[IS] OR "0906-6713"[IS] OR "1600-0757"[IS] OR "N/A"[IS] OR "2567-3173"[IS] OR "2059-7037"[IS] OR "2059-7037"[IS] OR "0009-9147"[IS] OR "1530-8561"[IS] OR "1226-3613"[IS] OR "2092-6413"[IS] OR "1323-1316"[IS] OR "1440-1819"[IS] OR "0017-8012"[IS] OR "0017-8012"[IS] OR "1040-4651"[IS] OR "1532-298X"[IS] OR "0001-8791"[IS] OR "1095-9084"[IS] OR "0742-3098"[IS] OR "1600-079X"[IS] OR "1094-2939"[IS] OR "1545-1577"[IS] OR "1201-9712"[IS] OR "1878-3511"[IS] OR "1350-9047"[IS] OR "1476-5403"[IS] OR "2190-5991"[IS] OR "2190-6009"[IS] OR "0002-9270"[IS] OR "1572-0241"[IS] OR "0012-8252"[IS] OR "1872-6828"[IS] OR "2326-6066"[IS] OR "2326-6074"[IS] OR "N/A"[IS] OR "2397-4621"[IS] OR "1469-493X"[IS] OR "1361-6137"[IS] OR "0008-8846"[IS] OR "1873-3948"[IS] OR "2573-5144"[IS] OR "2573-5144"[IS] OR "0112-1642"[IS] OR "1179-2035"[IS] OR "1081-0706"[IS] OR "1530-8995"[IS] OR "0960-8524"[IS] OR "1873-2976"[IS] OR "1548-9213"[IS] OR "1548-9221"[IS] OR "0168-9525"[IS] OR "1362-4555"[IS] OR "2168-6068"[IS] OR "2168-6084"[IS] OR "0027-8874"[IS] OR "1460-2105"[IS] OR "1866-3508"[IS] OR "1866-3516"[IS] OR "2213-9567"[IS] OR "2213-9567"[IS] OR "2522-0128"[IS] OR "2522-0136"[IS] OR "0021-9010"[IS] OR "1939-1854"[IS] OR "0028-3878"[IS] OR "1526-632X"[IS] OR "1568-1637"[IS] OR "1872-9649"[IS] OR "0924-2716"[IS] OR "1872-8235"[IS] OR "1863-2297"[IS] OR "1863-2300"[IS] OR "1064-3389"[IS] OR "1547-6537"[IS] OR "0007-0912"[IS] OR "1471-6771"[IS] OR "2467-964X"[IS] OR "2452-414X"[IS] OR "2055-

	6756"[IS] OR "2055-6764"[IS] OR "0008-1256"[IS] OR "2162-8564"[IS] OR "0095-1137"[IS] OR "1098-660X"[IS] OR "0031-5850"[IS] OR "N/A"[IS] OR "1551-3203"[IS] OR "1941-0050"[IS] OR "2214-8604"[IS] OR "2214-7810"[IS] OR "1745-6916"[IS] OR "1745-6924"[IS] OR "1549-1277"[IS] OR "1549-1676"[IS] OR "1838-7640"[IS] OR "1838-7640"[IS] OR "2161-8313"[IS] OR "2156-5376"[IS] OR "0006-3568"[IS] OR "1525-3244"[IS] OR "0090-0036"[IS] OR "1541-0048"[IS] OR "2328-8930"[IS] OR "N/A"[IS] OR "1557-1874"[IS] OR "1557-1882"[IS] OR "2226-4108"[IS] OR "2227-8508"[IS] OR "0196-8904"[IS] OR "1879-2227"[IS] OR "0268-005X"[IS] OR "1873-7137"[IS] OR "1759-0876"[IS] OR "1759-0884"[IS] OR "0002-8282"[IS] OR "1944-7981"[IS] OR "2168-2216"[IS] OR "2168-2232"[IS] OR "0168-3659"[IS] OR "1873-4995"[IS] OR "2524-7972"[IS] OR "2524-7867"[IS] OR "0306-2619"[IS] OR "1872-9118"[IS] OR "0012-6667"[IS] OR "1179-1950"[IS] OR "0304-419X"[IS] OR "1879-2561"[IS] OR "1087-0792"[IS] OR "1532-2955"[IS] OR "0272-7358"[IS] OR "1873-7811"[IS] OR "0166-4972"[IS] OR "1879-2383"[IS] OR "2332-7812"[IS] OR "2332-7812"[IS] OR "0013-936X"[IS] OR "1520-5851"[IS] OR "1461-6688"[IS] OR "1470-1340"[IS] OR "0008-3976"[IS] OR "1744-7976"[IS] OR "0003-6900"[IS] OR "2379-0407"[IS] OR "N/A"[IS] OR "2688-4062"[IS] OR "1523-9829"[IS] OR "1545-4274"[IS] OR "1359-8368"[IS] OR "1879-1069"[IS] OR "1094-9968"[IS] OR "1520-6653"[IS] OR "0008-6223"[IS] OR "1873-3891"[IS] OR "0033-0620"[IS] OR "1873-1740"[IS] OR "0364-5134"[IS] OR "1531-8249"[IS] OR "1461-023X"[IS] OR "1461-0248"[IS] OR "1359-8546"[IS] OR "1758-6852"[IS] OR "0925-5273"[IS] OR "1873-7579"[IS] OR "0166-3615"[IS] OR "1872-6194"[IS] OR "0165-1781"[IS] OR "1872-7123"[IS] OR "2530-7614"[IS] OR "2444-569X"[IS] OR "1751-7362"[IS] OR "1751-7370"[IS] OR "0011-9164"[IS] OR "1873-4464"[IS] OR "1040-8398"[IS] OR "1549-7852"[IS] OR "2352-3964"[IS] OR "2352-3964"[IS] OR "0022-4359"[IS] OR "1873-3271"[IS] OR "0360-1315"[IS] OR "1873-782X"[IS] OR "1040-8436"[IS] OR "1547-6561"[IS] OR "2055-6837"[IS] OR "2055-6845"[IS] OR "N/A"[IS] OR "2639-4979"[IS] OR "0959-3780"[IS] OR "1872-9495"[IS] OR "1741-7015"[IS] OR "1741-7015"[IS] OR "0007-1323"[IS] OR "1365-2168"[IS] OR "0007-0963"[IS] OR "1365-2133"[IS] OR "0025-6196"[IS] OR "1942-5546"[IS] OR "0047-2506"[IS] OR "1478-6990"[IS] OR "2192-2640"[IS] OR "2192-2659"[IS] OR "2405-4712"[IS] OR "2405-4720"[IS] OR "0161-5505"[IS] OR "1535-5667"[IS] OR "1936-8798"[IS] OR "1876-7605"[IS] OR "0959-6526"[IS] OR "1879-1786"[IS] OR "0272-6386"[IS] OR "1523-6838"[IS] OR "1077-5552"[IS] OR "1077-5552"[IS] OR "1758-5082"[IS] OR "1758-5090"[IS] OR "0390-6078"[IS] OR "N/A"[IS] OR "1052-9276"[IS] OR "1099-1654"[IS] OR "0002-9297"[IS] OR "1537-6605"[IS] OR "1057-7149"[IS] OR "1941-0042"[IS] OR "0278-0062"[IS] OR "1558-254X"[IS] OR "N/A"[IS] OR "2639-5274"[IS] OR "0091-6765"[IS] OR "1552-9924"[IS] OR "1463-9262"[IS] OR "1463-9270"[IS] OR "2213-2198"[IS] OR "2213-2201"[IS] OR "2542-5293"[IS] OR "2542-5293"[IS] OR "1939-1374"[IS] OR "1939-1374"[IS] OR "1474-9718"[IS] OR "1474-9726"[IS] OR "0105-2896"[IS] OR "1600-065X"[IS] OR "0001-4273"[IS] OR "1948-0989"[IS] OR "2198-6436"[IS] OR "2198-6436"[IS] OR "0969-6989"[IS] OR "1873-1384"[IS] OR "0148-2963"[IS] OR "1873-7978"[IS] OR "1745-2759"[IS] OR "1745-2767"[IS] OR "1863-8880"[IS] OR "1863-8899"[IS] OR "1082-989X"[IS] OR "1939-1463"[IS] OR "0960-9822"[IS] OR "1879-0445"[IS] OR "0301-0082"[IS] OR "1873-5118"[IS] OR "0040-1625"[IS] OR "1873-5509"[IS] OR "1467-7881"[IS] OR "1467-789X"[IS] OR "0066-4286"[IS] OR "1545-2107"[IS] OR "N/A"[IS] OR "2196-1115"[IS] OR "1080-0549"[IS] OR "1559-0267"[IS] OR "0964-4733"[IS] OR "1099-0836"[IS] OR "2213-2317"[IS] OR "2213-2317"[IS] OR "0363-9762"[IS] OR "1536-0229"[IS] OR "1884-4049"[IS] OR "1884-4057"[IS] OR "2590-0064"[IS] OR "2590-0064"[IS] OR "N/A"[IS] OR "2056-6387"[IS] OR "0048-9697"[IS] OR "1879-1026"[IS] OR "1449-2288"[IS] OR "1449-2288"[IS] OR "0144-8617"[IS] OR "1879-1344"[IS] OR "1836-9553"[IS] OR "1836-9561"[IS] OR "1367-5788"[IS] OR "1872-9088"[IS] OR "2210-6707"[IS] OR "2210-6715"[IS] OR "0002-9378"[IS] OR "1097-6868"[IS] OR "N/A"[IS] OR "2380-6761"[IS] OR "2214-9937"[IS] OR "2214-9937"[IS] OR "1044-5323"[IS] OR "1096-3618"[IS] OR "0007-1250"[IS] OR "1472-1465"[IS] OR "1053-4822"[IS] OR "1873-7889"[IS] OR "0969-6970"[IS] OR "1573-7209"[IS] OR "2334-2536"[IS] OR "2334-2536"[IS] OR "1742-7061"[IS] OR "1878-7568"[IS] OR "0268-960X"[IS] OR "1532-1681"[IS] OR "1753-5123"[IS] OR "1753-5131"[IS] OR "1555-9041"[IS] OR "1555-905X"[IS] OR "0033-2917"[IS] OR "1469-8978"[IS] OR "1043-2760"[IS] OR "1879-3061"[IS] OR "1478-0887"[IS] OR "1478-0895"[IS] OR "0007-6813"[IS] OR "1873-6068"[IS] OR "0376-7388"[IS] OR "1873-3123"[IS] OR "1471-4922"[IS] OR "1471-5007"[IS] OR "0926-5805"[IS] OR "1872-7891"[IS] OR "N/A"[IS] OR "2397-7132"[IS] OR "0098-7921"[IS] OR "1728-4457"[IS] OR "1079-5642"[IS] OR "1524-4636"[IS] OR "0897-4756"[IS] OR "1520-5002"[IS] OR "2367-2617"[IS] OR "2367-0983"[IS] OR "2157-6904"[IS] OR "2157-6912"[IS] OR "2095-5162"[IS] OR "2049-9957"[IS] OR "0020-7640"[IS] OR "1741-2854"[IS] OR "0012-186X"[IS] OR "1432-0428"[IS] OR "1941-3289"[IS] OR "1941-3297"[IS] OR "0945-053X"[IS] OR "1569-1802"[IS] OR "0278-4319"[IS] OR "1873-4693"[IS] OR "N/A"[IS] OR "1880-8190"[IS] OR "1569-9056"[IS] OR "1878-1500"[IS] OR "0049-3848"[IS] OR "1879-2472"[IS] OR "2330-8249"[IS] OR "2330-8257"[IS] OR "N/A"[IS] OR "1744-8603"[IS] OR "0016-5107"[IS] OR "1097-6779"[IS] OR "1944-8244"[IS] OR "1944-8252"[IS] OR "1674-7305"[IS] OR "1869-1889"[IS] OR "1043-6618"[IS] OR "1096-1186"[IS] OR "0079-6727"[IS] OR "1873-1627"[IS] OR "0890-6955"[IS] OR "1879-2170"[IS] OR "0378-7206"[IS] OR "1872-7530"[IS] OR "0028-646X"[IS] OR "1469-8137"[IS] OR "1005-0302"[IS] OR "1941-1162"[IS] OR "2110-

	5820"[IS] OR "2110-5820"[IS] OR "0890-8044"[IS] OR "1558-156X"[IS] OR "0958-1669"[IS] OR "1879-0429"[IS] OR "1949-3053"[IS] OR "1949-3061"[IS] OR "1684-1182"[IS] OR "1995-9133"[IS] OR "1998-0124"[IS] OR "1998-0000"[IS] OR "2210-6502"[IS] OR "2210-6510"[IS] OR "0012-3692"[IS] OR "1931-3543"[IS] OR "2327-4662"[IS] OR "2327-4662"[IS] OR "1726-2135"[IS] OR "1684-8799"[IS] OR "1747-938X"[IS] OR "1878-0385"[IS] OR "1521-6616"[IS] OR "1521-7035"[IS] OR "0144-1647"[IS] OR "1464-5327"[IS] OR "2162-2531"[IS] OR "2162-2531"[IS] OR "2040-7122"[IS] OR "2040-7130"[IS] OR "0956-7976"[IS] OR "1467-9280"[IS] OR "1931-5244"[IS] OR "1878-1810"[IS] OR "0039-2499"[IS] OR "1524-4628"[IS] OR "1674-7291"[IS] OR "1869-1870"[IS] OR "0065-3195"[IS] OR "1436-5030"[IS] OR "1449-4035"[IS] OR "1839-3373"[IS] OR "0166-3542"[IS] OR "1872-9096"[IS] OR "0736-5845"[IS] OR "1879-2537"[IS] OR "N/A"[IS] OR "2397-768X"[IS] OR "0894-3796"[IS] OR "1099-1379"[IS] OR "1757-7780"[IS] OR "1757-7799"[IS] OR "1755-263X"[IS] OR "1755-263X"[IS] OR "1093-9687"[IS] OR "1467-8667"[IS] OR "0020-9996"[IS] OR "1536-0210"[IS] OR "1619-7070"[IS] OR "1619-7089"[IS] OR "1094-6705"[IS] OR "1552-7379"[IS] OR "2195-1071"[IS] OR "2195-1071"[IS] OR "1366-5545"[IS] OR "1878-5794"[IS] OR "2196-5404"[IS] OR "2196-5404"[IS] OR "2631-8644"[IS] OR "2631-7990"[IS] OR "0360-0572"[IS] OR "1545-2115"[IS] OR "1873-9946"[IS] OR "1876-4479"[IS] OR "2211-467X"[IS] OR "2211-4688"[IS] OR "0959-8049"[IS] OR "1879-0852"[IS] OR "2211-1247"[IS] OR "2211-1247"[IS] OR "1042-2587"[IS] OR "1540-6520"[IS] OR "0269-7491"[IS] OR "1873-6424"[IS] OR "2041-6520"[IS] OR "2041-6539"[IS] OR "2152-5250"[IS] OR "2152-5250"[IS] OR "0021-9797"[IS] OR "1095-7103"[IS] OR "0895-3309"[IS] OR "1944-7965"[IS] OR "0065-3527"[IS] OR "1557-8399"[IS] OR "0958-9465"[IS] OR "1873-393X"[IS] OR "2095-0217"[IS] OR "2095-0225"[IS] OR "1048-9843"[IS] OR "1873-3409"[IS] OR "0960-0779"[IS] OR "1873-2887"[IS] OR "2590-0498"[IS] OR "2590-0498"[IS] OR "N/A"[IS] OR "2589-5559"[IS] OR "0194-911X"[IS] OR "1524-4563"[IS] OR "2059-8688"[IS] OR "2059-8696"[IS] OR "0737-6782"[IS] OR "1540-5885"[IS] OR "2047-9158"[IS] OR "2047-9158"[IS] OR "0022-3417"[IS] OR "1096-9896"[IS] OR "0266-3538"[IS] OR "1879-1050"[IS] OR "1751-2395"[IS] OR "1751-2409"[IS] OR "1889-1861"[IS] OR "1989-4007"[IS] OR "1544-6123"[IS] OR "1544-6131"[IS] OR "1571-0645"[IS] OR "1873-1457"[IS] OR "1936-8623"[IS] OR "1936-8631"[IS] OR "0012-9615"[IS] OR "1557-7015"[IS] OR "2044-5385"[IS] OR "2044-5385"[IS] OR "1556-603X"[IS] OR "1556-6048"[IS] OR "2214-7993"[IS] OR "2214-8000"[IS] OR "0378-7753"[IS] OR "1873-2755"[IS] OR "0013-726X"[IS] OR "1438-8812"[IS] OR "0304-3835"[IS] OR "1872-7980"[IS] OR "0022-2380"[IS] OR "1467-6486"[IS] OR "2198-6061"[IS] OR "2198-6061"[IS] OR "1943-0264"[IS] OR "1943-0264"[IS] OR "0031-4005"[IS] OR "1098-4275"[IS] OR "1742-4933"[IS] OR "1742-4933"[IS] OR "1947-5438"[IS] OR "1947-5446"[IS] OR "0885-3185"[IS] OR "1531-8257"[IS] OR "0300-5771"[IS] OR "1464-3685"[IS] OR "2041-4889"[IS] OR "2041-4889"[IS] OR "0093-7355"[IS] OR "1548-4475"[IS] OR "0893-6080"[IS] OR "1879-2782"[IS] OR "2213-5979"[IS] OR "2213-5979"[IS] OR "1743-7199"[IS] OR "1743-7202"[IS] OR "0022-3808"[IS] OR "1537-534X"[IS] OR "2379-3694"[IS] OR "2379-3694"[IS] OR "1544-9173"[IS] OR "1545-7885"[IS] OR "0269-2821"[IS] OR "1573-7462"[IS] OR "N/A"[IS] OR "1742-2094"[IS] OR "N/A"[IS] OR "2045-3701"[IS] OR "1286-4579"[IS] OR "1769-714X"[IS] OR "0905-9180"[IS] OR "1600-0617"[IS] OR "1524-9050"[IS] OR "1558-0016"[IS] OR "2287-237X"[IS] OR "2287-2388"[IS] OR "2452-302X"[IS] OR "2452-302X"[IS] OR "0269-2813"[IS] OR "1365-2036"[IS] OR "1945-0877"[IS] OR "1937-9145"[IS] OR "0278-6125"[IS] OR "1878-6642"[IS] OR "N/A"[IS] OR "2379-3708"[IS] OR "2051-8153"[IS] OR "2051-8161"[IS] OR "0048-7333"[IS] OR "1873-7625"[IS] OR "0007-1188"[IS] OR "1476-5381"[IS] OR "0966-9582"[IS] OR "1747-7646"[IS] OR "1359-835X"[IS] OR "1878-5840"[IS] OR "1757-5818"[IS] OR "1757-5826"[IS] OR "2397-3722"[IS] OR "2397-3722"[IS] OR "0079-6565"[IS] OR "1873-3301"[IS] OR "1088-9051"[IS] OR "1549-5469"[IS] OR "1949-0976"[IS] OR "1949-0984"[IS] OR "N/A"[IS] OR "1477-3155"[IS] OR "1939-5116"[IS] OR "1939-0041"[IS] OR "0264-1275"[IS] OR "1873-4197"[IS] OR "0264-2069"[IS] OR "1743-9507"[IS] OR "N/A"[IS] OR "2059-0105"[IS] OR "1942-9681"[IS] OR "1942-969X"[IS] OR "1369-5266"[IS] OR "1879-0356"[IS] OR "2210-4224"[IS] OR "2210-4232"[IS] OR "0952-8180"[IS] OR "1873-4529"[IS] OR "1754-9426"[IS] OR "1754-9434"[IS] OR "2666-4984"[IS] OR "2666-4984"[IS] OR "1600-6135"[IS] OR "1600-6143"[IS] OR "N/A"[IS] OR "2589-9147"[IS] OR "0144-3577"[IS] OR "1758-6593"[IS] OR "1757-7004"[IS] OR "1757-7012"[IS] OR "1527-8204"[IS] OR "1545-293X"[IS] OR "0012-1797"[IS] OR "1939-327X"[IS] OR "1350-4177"[IS] OR "1873-2828"[IS] OR "0199-9885"[IS] OR "1545-4312"[IS] OR "0098-5589"[IS] OR "1939-3520"[IS] OR "0959-6119"[IS] OR "1757-1049"[IS] OR "1759-2879"[IS] OR "1759-2887"[IS] OR "1389-9155"[IS] OR "1573-2606"[IS] OR "N/A"[IS] OR "2373-8057"[IS] OR "1930-7381"[IS] OR "1930-739X"[IS] OR "0090-3493"[IS] OR "1530-0293"[IS] OR "1818-0876"[IS] OR "1818-0876"[IS] OR "0065-2113"[IS] OR "2213-6789"[IS] OR "2468-6069"[IS] OR "2468-6069"[IS] OR "0140-9883"[IS] OR "1873-6181"[IS] OR "0167-7659"[IS] OR "1573-7233"[IS] OR "1041-4347"[IS] OR "1558-2191"[IS] OR "0308-8146"[IS] OR "1873-7072"[IS] OR "0926-9959"[IS] OR "1468-3083"[IS] OR "2168-0485"[IS] OR "2168-0485"[IS] OR "N/A"[IS] OR "0925-4005"[IS] OR "2531-0437"[IS] OR "2531-0437"[IS] OR "1359-6454"[IS] OR "1873-2453"[IS] OR "1420-682X"[IS] OR "1420-9071"[IS] OR "0067-0049"[IS] OR "1538-4365"[IS] OR "N/A"[IS] OR "2307-387X"[IS] OR "1708-8569"[IS] OR "1867-0687"[IS] OR "0031-9007"[IS] OR "1079-7114"[IS] OR "1935-861X"[IS] OR "1876-4754"[IS] OR

	"2367-198X"[IS] OR "2367-198X"[IS] OR "1063-5157"[IS] OR "1076-836X"[IS] OR "0736-5853"[IS] OR "N/A"[IS] OR "1864-5631"[IS] OR "1864-564X"[IS] OR "1383-5866"[IS] OR "1873-3794"[IS] OR "2047-2404"[IS] OR "2047-2412"[IS] OR "0003-0007"[IS] OR "1520-0477"[IS] OR "1743-8977"[IS] OR "1743-8977"[IS] OR "1672-9072"[IS] OR "1744-7909"[IS] OR "0040-6376"[IS] OR "1468-3296"[IS] OR "1881-7815"[IS] OR "1881-7823"[IS] OR "0007-0920"[IS] OR "1532-1827"[IS] OR "1469-221X"[IS] OR "1469-3178"[IS] OR "0738-8551"[IS] OR "1549-7801"[IS] OR "0149-7634"[IS] OR "1873-7528"[IS] OR "0278-2715"[IS] OR "0278-2715"[IS] OR "2165-0497"[IS] OR "2165-0497"[IS] OR "2330-2674"[IS] OR "2330-2682"[IS] OR "2451-9448"[IS] OR "2451-9448"[IS] OR "0263-7863"[IS] OR "1873-4634"[IS] OR "0163-6804"[IS] OR "1558-1896"[IS] OR "1936-0533"[IS] OR "1936-0541"[IS] OR "2211-9124"[IS] OR "2211-9124"[IS] OR "0968-090X"[IS] OR "1879-2359"[IS] OR "0020-7543"[IS] OR "1366-588X"[IS] OR "1047-1987"[IS] OR "1476-4989"[IS] OR "0277-7037"[IS] OR "1098-2787"[IS] OR "0960-085X"[IS] OR "1476-9344"[IS] OR "N/A"[IS] OR "2352-7714"[IS] OR "0884-8173"[IS] OR "1098-111X"[IS] OR "0003-3022"[IS] OR "1528-1175"[IS] OR "1367-5931"[IS] OR "1879-0402"[IS] OR "2168-6181"[IS] OR "2168-619X"[IS] OR "1460-8545"[IS] OR "1468-2370"[IS] OR "0747-5632"[IS] OR "1873-7692"[IS] OR "N/A"[IS] OR "1475-2840"[IS] OR "0195-6701"[IS] OR "1532-2939"[IS] OR "0038-6308"[IS] OR "1572-9672"[IS] OR "0045-6535"[IS] OR "1879-1298"[IS] OR "0376-0421"[IS] OR "1873-1724"[IS] OR "0888-3270"[IS] OR "1096-1216"[IS] OR "2096-4579"[IS] OR "2096-4579"[IS] OR "0047-2875"[IS] OR "1552-6763"[IS] OR "0022-0345"[IS] OR "1544-0591"[IS] OR "2352-5509"[IS] OR "2352-5509"[IS] OR "2040-4603"[IS] OR "2040-4603"[IS] OR "N/A"[IS] OR "1479-5868"[IS] OR "0301-4797"[IS] OR "1095-8630"[IS] OR "N/A"[IS] OR "2352-152X"[IS] OR "2365-0869"[IS] OR "2364-8961"[IS] OR "0019-8501"[IS] OR "1873-2062"[IS] OR "1098-3600"[IS] OR "1530-0366"[IS] OR "0360-5442"[IS] OR "1873-6785"[IS] OR "2365-709X"[IS] OR "2365-709X"[IS] OR "N/A"[IS] OR "2328-4277"[IS] OR "2452-2236"[IS] OR "2452-2236"[IS] OR "1319-1578"[IS] OR "2213-1248"[IS] OR "1878-5077"[IS] OR "1878-5085"[IS] OR "2451-9766"[IS] OR "2451-9685"[IS] OR "1096-7176"[IS] OR "1096-7184"[IS] OR "N/A"[IS] OR "1758-9193"[IS] OR "0956-053X"[IS] OR "1879-2456"[IS] OR "N/A"[IS] OR "2042-6410"[IS] OR "2041-8205"[IS] OR "2041-8213"[IS] OR "0737-4038"[IS] OR "1537-1719"[IS] OR "2352-345X"[IS] OR "2352-345X"[IS] OR "1664-3224"[IS] OR "1664-3224"[IS] OR "1546-3222"[IS] OR "2325-6621"[IS] OR "2059-2302"[IS] OR "2059-2310"[IS] OR "1574-0137"[IS] OR "1876-7745"[IS] OR "0950-9232"[IS] OR "1476-5594"[IS] OR "0960-7722"[IS] OR "1365-2184"[IS] OR "1478-3223"[IS] OR "1478-3231"[IS] OR "2214-2894"[IS] OR "2214-2894"[IS] OR "0167-8299"[IS] OR "2191-0235"[IS] OR "2053-9517"[IS] OR "2053-9517"[IS] OR "0304-4602"[IS] OR "N/A"[IS] OR "2050-084X"[IS] OR "2050-084X"[IS] OR "1425-8153"[IS] OR "1689-1392"[IS] OR "0018-9391"[IS] OR "1558-0040"[IS] OR "1933-0219"[IS] OR "1935-3456"[IS] OR "1040-9238"[IS] OR "1549-7798"[IS] OR "1573-5214"[IS] OR "2212-1307"[IS] OR "1367-3270"[IS] OR "1758-7484"[IS] OR "N/A"[IS] OR "2052-1537"[IS] OR "1387-6473"[IS] OR "1872-9630"[IS] OR "2589-9333"[IS] OR "2589-9333"[IS] OR "1755-098X"[IS] OR "1755-0998"[IS] OR "0960-7692"[IS] OR "1469-0705"[IS] OR "0305-0483"[IS] OR "1873-5274"[IS] OR "0957-4174"[IS] OR "1873-6793"[IS] OR "2352-9407"[IS] OR "2352-9407"[IS] OR "2095-8226"[IS] OR "2199-4501"[IS] OR "1090-9516"[IS] OR "1878-5573"[IS] OR "0960-1481"[IS] OR "1879-0682"[IS] OR "N/A"[IS] OR "2050-7771"[IS] OR "2287-6391"[IS] OR "2287-6405"[IS] OR "2590-3462"[IS] OR "2590-3462"[IS] OR "0007-1048"[IS] OR "1365-2141"[IS] OR "0093-5301"[IS] OR "1537-5277"[IS] OR "1553-2739"[IS] OR "1434-4599"[IS] OR "N/A"[IS] OR "2162-3619"[IS] OR "1096-7516"[IS] OR "1873-5525"[IS] OR "2352-8478"[IS] OR "2352-8478"[IS] OR "1941-9651"[IS] OR "1942-0080"[IS] OR "1129-2369"[IS] OR "1129-2377"[IS] OR "1759-8478"[IS] OR "1759-8486"[IS] OR "2212-8778"[IS] OR "2212-8778"[IS] OR "0968-0802"[IS] OR "1099-1719"[IS] OR "N/A"[IS] OR "2666-6065"[IS] OR "2001-1326"[IS] OR "2001-1326"[IS] OR "0038-0717"[IS] OR "1879-3428"[IS] OR "0271-9142"[IS] OR "1573-2592"[IS] OR "2047-4873"[IS] OR "2047-4881"[IS] OR "2374-6149"[IS] OR "2374-6149"[IS] OR "0031-3203"[IS] OR "1873-5142"[IS] OR "2166-3831"[IS] OR "2166-3831"[IS] OR "2214-6296"[IS] OR "2214-6326"[IS] OR "0276-7783"[IS] OR "N/A"[IS] OR "N/A"[IS] OR "2378-2242"[IS] OR "N/A"[IS] OR "2052-4463"[IS] OR "0749-6419"[IS] OR "1879-2154"[IS] OR "0740-624X"[IS] OR "1872-9517"[IS] OR "1434-6621"[IS] OR "1437-4331"[IS] OR "1062-7995"[IS] OR "1099-159X"[IS] OR "1758-2946"[IS] OR "1758-2946"[IS] OR "0002-9165"[IS] OR "1938-3207"[IS] OR "N/A"[IS] OR "1942-2466"[IS] OR "1535-3958"[IS] OR "1535-3966"[IS] OR "N/A"[IS] OR "2055-5008"[IS] OR "0022-3514"[IS] OR "1939-1315"[IS] OR "0928-4931"[IS] OR "1873-0191"[IS] OR "1001-8417"[IS] OR "1878-5964"[IS] OR "N/A"[IS] OR "1479-5876"[IS] OR "1529-6466"[IS] OR "1943-2666"[IS] OR "0013-9351"[IS] OR "1096-0953"[IS] OR "2666-0873"[IS] OR "2666-0873"[IS] OR "0893-9454"[IS] OR "1465-7368"[IS] OR "1465-5411"[IS] OR "1465-542X"[IS] OR "0079-6786"[IS] OR "1873-1643"[IS] OR "0955-0674"[IS] OR "1879-0410"[IS] OR "2214-2096"[IS] OR "2214-2096"[IS] OR "1359-6446"[IS] OR "1878-5832"[IS] OR "0273-2297"[IS] OR "1090-2406"[IS] OR "1932-4529"[IS] OR "1941-0115"[IS] OR "1536-1276"[IS] OR "1558-2248"[IS] OR "2287-2728"[IS] OR "2287-285X"[IS] OR "2041-210X"[IS] OR "2041-2096"[IS] OR "0091-3022"[IS] OR "1095-6808"[IS] OR "0003-1305"[IS] OR "1537-2731"[IS] OR "0188-4409"[IS] OR "1873-5487"[IS] OR "2212-9820"[IS] OR "2212-9839"[IS] OR "1949-3029"[IS] OR "1949-3037"[IS] OR "2040-3364"[IS] OR "2040-3372"[IS] OR "N/A"[IS]

	OR "2572-9241"[IS] OR "0893-133X"[IS] OR "1740-634X"[IS] OR "1941-7705"[IS] OR "1941-7713"[IS] OR "2304-3881"[IS] OR "2304-389X"[IS] OR "0021-9630"[IS] OR "1469-7610"[IS] OR "1568-4946"[IS] OR "1872-9681"[IS] OR "0047-2727"[IS] OR "0047-2727"[IS] OR "1262-3636"[IS] OR "1878-1780"[IS] OR "2168-6165"[IS] OR "2168-6173"[IS] OR "1866-0452"[IS] OR "1866-0452"[IS] OR "0033-295X"[IS] OR "1939-1471"[IS] OR "1094-4281"[IS] OR "1552-7425"[IS] OR "1040-726X"[IS] OR "1573-336X"[IS] OR "0304-405X"[IS] OR "N/A"[IS] OR "1057-5219"[IS] OR "1873-8079"[IS] OR "0020-0255"[IS] OR "1872-6291"[IS] OR "2214-6350"[IS] OR "2214-6369"[IS] OR "0301-4207"[IS] OR "1873-7641"[IS] OR "0046-1520"[IS] OR "1532-6985"[IS] OR "1359-0294"[IS] OR "1879-0399"[IS] OR "0893-3952"[IS] OR "1530-0285"[IS] OR "N/A"[IS] OR "2588-9311"[IS] OR "1674-2788"[IS] OR "1759-4685"[IS] OR "1018-9068"[IS] OR "1698-0808"[IS] OR "1520-9210"[IS] OR "1941-0077"[IS] OR "0168-8227"[IS] OR "1872-8227"[IS] OR "1054-8408"[IS] OR "1540-7306"[IS] OR "1134-3060"[IS] OR "1886-1784"[IS] OR "1749-7922"[IS] OR "1749-7922"[IS] OR "0278-0046"[IS] OR "1557-9948"[IS] OR "0954-4224"[IS] OR "1475-2700"[IS] OR "1741-038X"[IS] OR "1758-7786"[IS] OR "0033-3352"[IS] OR "1540-6210"[IS] OR "0950-7051"[IS] OR "1872-7409"[IS] OR "0378-3820"[IS] OR "1873-7188"[IS] OR "1742-5247"[IS] OR "1744-7593"[IS] OR "1520-7552"[IS] OR "1520-7560"[IS] OR "1091-4269"[IS] OR "1520-6394"[IS] OR "0196-2892"[IS] OR "1558-0644"[IS] OR "0169-2046"[IS] OR "1872-6062"[IS] OR "1359-107X"[IS] OR "2044-8287"[IS] OR "0891-5849"[IS] OR "1873-4596"[IS] OR "2198-6592"[IS] OR "2198-6592"[IS] OR "0177-0667"[IS] OR "1435-5663"[IS] OR "1523-3812"[IS] OR "1535-1645"[IS] OR "N/A"[IS] OR "1757-6512"[IS] OR "0021-9525"[IS] OR "1540-8140"[IS] OR "0894-1491"[IS] OR "1098-1136"[IS] OR "1093-7404"[IS] OR "1521-6950"[IS] OR "0165-2176"[IS] OR "1875-5941"[IS] OR "1558-9080"[IS] OR "N/A"[IS] OR "2050-7526"[IS] OR "2050-7534"[IS] OR "1867-2949"[IS] OR "1867-2957"[IS] OR "2059-7908"[IS] OR "2059-7908"[IS] OR "1050-1738"[IS] OR "1873-2615"[IS] OR "0003-0554"[IS] OR "1537-5943"[IS] OR "0021-9517"[IS] OR "1090-2694"[IS] OR "0969-5931"[IS] OR "1873-6149"[IS] OR "0167-8116"[IS] OR "1873-8001"[IS] OR "1931-2458"[IS] OR "1931-2466"[IS] OR "2210-6340"[IS] OR "2210-6359"[IS] OR "0022-2623"[IS] OR "1520-4804"[IS] OR "0016-2361"[IS] OR "1873-7153"[IS] OR "1523-2409"[IS] OR "1745-493X"[IS] OR "0141-8130"[IS] OR "1879-0003"[IS] OR "2162-4968"[IS] OR "2162-4968"[IS] OR "N/A"[IS] OR "2213-4530"[IS] OR "0360-3016"[IS] OR "1879-355X"[IS] OR "0003-2700"[IS] OR "1520-6882"[IS] OR "2055-7434"[IS] OR "2055-7434"[IS] OR "0032-0889"[IS] OR "1532-2548"[IS] OR "2008-9244"[IS] OR "2193-8865"[IS])
SJR Q1	("1542-4863"[IS] OR "0007-9235"[IS] OR "0033-5533"[IS] OR "1531-4650"[IS] OR "1471-0072"[IS] OR "1471-0080"[IS] OR "0092-8674"[IS] OR "1097-4172"[IS] OR "0028-4793"[IS] OR "1533-4406"[IS] OR "1546-170X"[IS] OR "1078-8956"[IS] OR "1057-5987"[IS] OR "1545-8601"[IS] OR "1546-1696"[IS] OR "1087-0156"[IS] OR "2058-8437"[IS] OR "2058-8437"[IS] OR "0002-8282"[IS] OR "0002-8282"[IS] OR "1474-175X"[IS] OR "1474-1768"[IS] OR "1476-4687"[IS] OR "0028-0836"[IS] OR "0022-3808"[IS] OR "1537-534X"[IS] OR "0034-6861"[IS] OR "1539-0756"[IS] OR "2058-7546"[IS] OR "2058-7546"[IS] OR "1471-0056"[IS] OR "1471-0064"[IS] OR "1474-1784"[IS] OR "1474-1776"[IS] OR "1520-6890"[IS] OR "0009-2665"[IS] OR "1474-1741"[IS] OR "1474-1733"[IS] OR "0149-2195"[IS] OR "1545-861X"[IS] OR "1061-4036"[IS] OR "1546-1718"[IS] OR "0001-8392"[IS] OR "1930-3815"[IS] OR "1551-8922"[IS] OR "1551-8930"[IS] OR "1941-6520"[IS] OR "1941-6067"[IS] OR "0022-1082"[IS] OR "1540-6261"[IS] OR "1545-3278"[IS] OR "0732-0582"[IS] OR "1545-8636"[IS] OR "1546-0738"[IS] OR "0306-0012"[IS] OR "1460-4744"[IS] OR "1759-4782"[IS] OR "1759-4774"[IS] OR "1097-4180"[IS] OR "1074-7613"[IS] OR "0140-6736"[IS] OR "1474-547X"[IS] OR "1545-4282"[IS] OR "0066-4146"[IS] OR "0034-6527"[IS] OR "1467-937X"[IS] OR "1548-7091"[IS] OR "1548-7105"[IS] OR "1723-8617"[IS] OR "1723-8617"[IS] OR "1553-877X"[IS] OR "1553-877X"[IS] OR "1476-4660"[IS] OR "1476-1122"[IS] OR "1935-8245"[IS] OR "1935-8237"[IS] OR "1522-1210"[IS] OR "0031-9333"[IS] OR "2380-8942"[IS] OR "2380-8950"[IS] OR "1095-9203"[IS] OR "0036-8075"[IS] OR "2520-1158"[IS] OR "2520-1158"[IS] OR "1748-3387"[IS] OR "1748-3395"[IS] OR "0012-9682"[IS] OR "1468-0262"[IS] OR "0022-0515"[IS] OR "0022-0515"[IS] OR "1945-7790"[IS] OR "1945-7782"[IS] OR "1535-6108"[IS] OR "1878-3686"[IS] OR "1754-5692"[IS] OR "1754-5706"[IS] OR "2542-4351"[IS] OR "2542-4351"[IS] OR "1470-2045"[IS] OR "1474-5488"[IS] OR "1465-7368"[IS] OR "0893-9454"[IS] OR "1097-6256"[IS] OR "1546-1726"[IS] OR "1460-6976"[IS] OR "0001-8732"[IS] OR "1569-8041"[IS] OR "0923-7534"[IS] OR "1749-4885"[IS] OR "1749-4885"[IS] OR "1433-8351"[IS] OR "2367-3613"[IS] OR "2056-676X"[IS] OR "2056-676X"[IS] OR "2213-2619"[IS] OR "2213-2600"[IS] OR "2470-9468"[IS] OR "2470-9468"[IS] OR "1545-4509"[IS] OR "0066-4154"[IS] OR "2397-3358"[IS] OR "2397-3358"[IS] OR "2520-1131"[IS] OR "2520-1131"[IS] OR "1529-2908"[IS] OR "1529-2916"[IS] OR "0001-4273"[IS] OR "0001-4273"[IS] OR "1945-7707"[IS] OR "1945-7715"[IS] OR "1547-7185"[IS] OR "0022-2429"[IS] OR "0363-7425"[IS] OR "0363-7425"[IS] OR "1947-5454"[IS] OR "1947-5462"[IS] OR "0304-405X"[IS] OR "0304-405X"[IS] OR "2468-2667"[IS] OR "2468-2667"[IS] OR "1934-3396"[IS] OR "1934-340X"[IS] OR "1740-1534"[IS] OR "1740-1526"[IS] OR "1941-1383"[IS] OR "1941-1391"[IS] OR "1931-3128"[IS] OR "1931-3128"[IS] OR "1759-5045"[IS] OR "1759-5053"[IS] OR "1527-7755"[IS] OR "0732-183X"[IS] OR "1550-4131"[IS] OR "1550-4131"[IS] OR "0163-7525"[IS] OR

	"1545-2093"[IS] OR "0935-4956"[IS] OR "1432-0754"[IS] OR "1474-4465"[IS] OR "1474-4422"[IS] OR "2662-1347"[IS] OR "2662-1347"[IS] OR "1476-4679"[IS] OR "1465-7392"[IS] OR "1097-4164"[IS] OR "1097-2765"[IS] OR "0935-9648"[IS] OR "1521-4095"[IS] OR "2662-8449"[IS] OR "2662-8449"[IS] OR "2666-5247"[IS] OR "2666-5247"[IS] OR "2522-5820"[IS] OR "2522-5820"[IS] OR "0065-2458"[IS] OR "0065-2458"[IS] OR "1474-7596"[IS] OR "1474-7596"[IS] OR "0066-4308"[IS] OR "1545-2085"[IS] OR "2058-5276"[IS] OR "2058-5276"[IS] OR "1934-5909"[IS] OR "1934-5909"[IS] OR "8755-1209"[IS] OR "8755-1209"[IS] OR "1614-6832"[IS] OR "1614-6840"[IS] OR "2520-8136"[IS] OR "2520-8136"[IS] OR "1476-4598"[IS] OR "1476-4598"[IS] OR "1553-4006"[IS] OR "1553-4014"[IS] OR "0895-3309"[IS] OR "0895-3309"[IS] OR "1468-3288"[IS] OR "0017-5749"[IS] OR "1873-2208"[IS] OR "0079-6425"[IS] OR "1097-0266"[IS] OR "0143-2095"[IS] OR "0147-006X"[IS] OR "1545-4126"[IS] OR "0034-6535"[IS] OR "1530-9142"[IS] OR "1094-2939"[IS] OR "1545-1577"[IS] OR "1558-3597"[IS] OR "0735-1097"[IS] OR "1943-8206"[IS] OR "1943-8206"[IS] OR "0305-1048"[IS] OR "1362-4962"[IS] OR "2213-8595"[IS] OR "2213-8587"[IS] OR "1945-7731"[IS] OR "1945-774X"[IS] OR "2215-0374"[IS] OR "2215-0366"[IS] OR "1474-4457"[IS] OR "1473-3099"[IS] OR "1755-4330"[IS] OR "1755-4349"[IS] OR "1545-2123"[IS] OR "1543-5008"[IS] OR "1759-5037"[IS] OR "1759-5029"[IS] OR "2374-2445"[IS] OR "2374-2437"[IS] OR "1748-7838"[IS] OR "1001-0602"[IS] OR "1745-2473"[IS] OR "1745-2473"[IS] OR "2380-8195"[IS] OR "2380-8195"[IS] OR "0009-7322"[IS] OR "1524-4539"[IS] OR "1097-4199"[IS] OR "0896-6273"[IS] OR "1537-5943"[IS] OR "0003-0554"[IS] OR "0733-8716"[IS] OR "0733-8716"[IS] OR "1528-0012"[IS] OR "0016-5085"[IS] OR "0033-2909"[IS] OR "1939-1455"[IS] OR "0732-2399"[IS] OR "1526-548X"[IS] OR "0893-8512"[IS] OR "1098-6618"[IS] OR "1545-9993"[IS] OR "1545-9985"[IS] OR "0022-2437"[IS] OR "1547-7193"[IS] OR "0003-1224"[IS] OR "1939-8271"[IS] OR "1600-0641"[IS] OR "0168-8278"[IS] OR "2380-6583"[IS] OR "2380-6591"[IS] OR "2214-109X"[IS] OR "2214-109X"[IS] OR "1545-2948"[IS] OR "0066-4197"[IS] OR "2159-8274"[IS] OR "2159-8290"[IS] OR "1614-4961"[IS] OR "2367-3648"[IS] OR "0149-2063"[IS] OR "1557-1211"[IS] OR "1945-7189"[IS] OR "0163-769X"[IS] OR "2666-7762"[IS] OR "2666-7762"[IS] OR "1526-5498"[IS] OR "1523-4614"[IS] OR "2405-4712"[IS] OR "2405-4712"[IS] OR "2522-5812"[IS] OR "2522-5812"[IS] OR "2327-0608"[IS] OR "2327-0608"[IS] OR "1471-0048"[IS] OR "1471-003X"[IS] OR "0302-2838"[IS] OR "1421-993X"[IS] OR "1759-5010"[IS] OR "1759-5002"[IS] OR "1936-1238"[IS] OR "1936-122X"[IS] OR "1758-6798"[IS] OR "1758-678X"[IS] OR "0927-796X"[IS] OR "0927-796X"[IS] OR "2451-9294"[IS] OR "2451-9294"[IS] OR "0003-486X"[IS] OR "0003-486X"[IS] OR "1548-5951"[IS] OR "1548-5943"[IS] OR "1754-2189"[IS] OR "1754-2189"[IS] OR "2168-6157"[IS] OR "2168-6149"[IS] OR "1538-3598"[IS] OR "0098-9955"[IS] OR "1932-8346"[IS] OR "1932-8354"[IS] OR "2059-3635"[IS] OR "2095-9907"[IS] OR "1530-8995"[IS] OR "1081-0706"[IS] OR "2157-846X"[IS] OR "2157-846X"[IS] OR "1618-1913"[IS] OR "0073-8301"[IS] OR "2168-6238"[IS] OR "2168-622X"[IS] OR "2398-9629"[IS] OR "2398-9629"[IS] OR "1536-1284"[IS] OR "1536-1284"[IS] OR "1526-5455"[IS] OR "1047-7039"[IS] OR "1759-4766"[IS] OR "1759-4758"[IS] OR "1759-507X"[IS] OR "1759-5061"[IS] OR "0003-4967"[IS] OR "1468-2060"[IS] OR "2470-9476"[IS] OR "2470-9476"[IS] OR "2589-7500"[IS] OR "2589-7500"[IS] OR "0066-4278"[IS] OR "1545-1585"[IS] OR "0001-4842"[IS] OR "1520-4898"[IS] OR "1946-6234"[IS] OR "1946-6242"[IS] OR "1474-0508"[IS] OR "0962-4929"[IS] OR "2160-0031"[IS] OR "1529-1006"[IS] OR "2160-3308"[IS] OR "2160-3308"[IS] OR "2589-7780"[IS] OR "2589-7780"[IS] OR "0092-0703"[IS] OR "1552-7824"[IS] OR "1073-449X"[IS] OR "1535-4970"[IS] OR "1545-4479"[IS] OR "0066-4189"[IS] OR "0022-1007"[IS] OR "1540-9538"[IS] OR "1432-1238"[IS] OR "0342-4642"[IS] OR "1744-4292"[IS] OR "1744-4292"[IS] OR "2522-5839"[IS] OR "2522-5839"[IS] OR "1369-7021"[IS] OR "1369-7021"[IS] OR "2662-138X"[IS] OR "2662-138X"[IS] OR "2468-1253"[IS] OR "2468-1253"[IS] OR "2567-3165"[IS] OR "2567-3165"[IS] OR "0735-0015"[IS] OR "1537-2707"[IS] OR "1939-1854"[IS] OR "0021-9010"[IS] OR "1752-0908"[IS] OR "1752-0894"[IS] OR "0165-4101"[IS] OR "0165-4101"[IS] OR "1542-4774"[IS] OR "1542-4766"[IS] OR "1941-4927"[IS] OR "1941-4935"[IS] OR "0084-6597"[IS] OR "0084-6597"[IS] OR "1756-8722"[IS] OR "1756-8722"[IS] OR "0093-5301"[IS] OR "1537-5277"[IS] OR "1935-5548"[IS] OR "0149-5992"[IS] OR "0962-8924"[IS] OR "1879-3088"[IS] OR "0163-8998"[IS] OR "0163-8998"[IS] OR "1520-5126"[IS] OR "0002-7863"[IS] OR "0304-3932"[IS] OR "0304-3932"[IS] OR "1556-1380"[IS] OR "1556-0864"[IS] OR "1088-6834"[IS] OR "0894-0347"[IS] OR "2666-3791"[IS] OR "2666-3791"[IS] OR "0034-6543"[IS] OR "1935-1046"[IS] OR "1560-2745"[IS] OR "1878-9129"[IS] OR "1549-5477"[IS] OR "0890-9369"[IS] OR "1089-778X"[IS] OR "1089-778X"[IS] OR "0304-4076"[IS] OR "0304-4076"[IS] OR "1088-8683"[IS] OR "1532-7957"[IS] OR "0370-1573"[IS] OR "0370-1573"[IS] OR "1573-692X"[IS] OR "1572-3097"[IS] OR "0360-1285"[IS] OR "0360-1285"[IS] OR "0079-6700"[IS] OR "0079-6700"[IS] OR "1536-1276"[IS] OR "1536-1276"[IS] OR "0066-4170"[IS] OR "1545-4487"[IS] OR "2515-2459"[IS] OR "2515-2467"[IS] OR "2397-3374"[IS] OR "2397-3374"[IS] OR "1879-307X"[IS] OR "1364-6613"[IS] OR "1388-9842"[IS] OR "1879-0844"[IS] OR "1433-7851"[IS] OR "1521-3773"[IS] OR "1616-301X"[IS] OR "1616-301X"[IS] OR "1558-2256"[IS] OR "0018-9219"[IS] OR "1745-6916"[IS] OR "1745-6924"[IS] OR "1552-4469"[IS] OR "1552-4450"[IS] OR "0261-4189"[IS] OR "1460-2075"[IS] OR "2666-7924"[IS] OR "2666-7924"[IS] OR "2046-9136"[IS] OR "2046-9128"[IS] OR "0066-4227"[IS] OR

	"1545-3251"[IS] OR "1543-592X"[IS] OR "1543-592X"[IS] OR "1460-2105"[IS] OR "0027-8874"[IS] OR "2047-7538"[IS] OR "2047-7538"[IS] OR "1534-5807"[IS] OR "1878-1551"[IS] OR "1096-0473"[IS] OR "1042-9573"[IS] OR "2397-334X"[IS] OR "2397-334X"[IS] OR "2168-2275"[IS] OR "2168-2267"[IS] OR "2213-1787"[IS] OR "2213-1779"[IS] OR "1521-0081"[IS] OR "0031-6997"[IS] OR "1572-2740"[IS] OR "1572-2740"[IS] OR "0025-1909"[IS] OR "1526-5501"[IS] OR "0008-8846"[IS] OR "0008-8846"[IS] OR "0163-6804"[IS] OR "0163-6804"[IS] OR "2311-6706"[IS] OR "2150-5551"[IS] OR "0090-5364"[IS] OR "0090-5364"[IS] OR "1674-2052"[IS] OR "1752-9867"[IS] OR "2055-026X"[IS] OR "2055-026X"[IS] OR "0166-0616"[IS] OR "0166-0616"[IS] OR "1527-8204"[IS] OR "1545-293X"[IS] OR "2405-8297"[IS] OR "2405-8297"[IS] OR "2213-6657"[IS] OR "2213-6657"[IS] OR "1475-679X"[IS] OR "0021-8456"[IS] OR "1750-1326"[IS] OR "1750-1326"[IS] OR "1945-7669"[IS] OR "1945-7685"[IS] OR "0169-5347"[IS] OR "1872-8383"[IS] OR "1949-3053"[IS] OR "1949-3053"[IS] OR "0021-9738"[IS] OR "1558-8238"[IS] OR "2041-1723"[IS] OR "2041-1723"[IS] OR "2168-6211"[IS] OR "2168-6203"[IS] OR "0047-2727"[IS] OR "0047-2727"[IS] OR "1941-0611"[IS] OR "1941-1405"[IS] OR "0047-2506"[IS] OR "1478-6990"[IS] OR "2666-6758"[IS] OR "2666-6758"[IS] OR "1573-4412"[IS] OR "1573-4412"[IS] OR "2211-2855"[IS] OR "2211-2855"[IS] OR "0883-9026"[IS] OR "0883-9026"[IS] OR "1094-6705"[IS] OR "1552-7379"[IS] OR "1468-960X"[IS] OR "1362-0347"[IS] OR "0092-5853"[IS] OR "1540-5907"[IS] OR "1537-6605"[IS] OR "0002-9297"[IS] OR "1350-9462"[IS] OR "1873-1635"[IS] OR "1528-0020"[IS] OR "0006-4971"[IS] OR "2168-6114"[IS] OR "2168-6106"[IS] OR "1549-5469"[IS] OR "1088-9051"[IS] OR "0268-4012"[IS] OR "0268-4012"[IS] OR "1531-3468"[IS] OR "1537-2618"[IS] OR "0001-6322"[IS] OR "1432-0533"[IS] OR "2590-2393"[IS] OR "2590-2385"[IS] OR "1873-3883"[IS] OR "0926-3373"[IS] OR "2666-979X"[IS] OR "2666-979X"[IS] OR "0146-6410"[IS] OR "0146-6410"[IS] OR "1935-4185"[IS] OR "1935-4185"[IS] OR "1756-994X"[IS] OR "1756-994X"[IS] OR "1558-9080"[IS] OR "1558-9080"[IS] OR "1522-8517"[IS] OR "1523-5866"[IS] OR "2325-6826"[IS] OR "2325-6818"[IS] OR "1047-1987"[IS] OR "1476-4989"[IS] OR "0020-9910"[IS] OR "1432-1297"[IS] OR "1531-5088"[IS] OR "0020-8183"[IS] OR "2352-3026"[IS] OR "2352-3026"[IS] OR "2643-6728"[IS] OR "2643-6728"[IS] OR "2577-5421"[IS] OR "2577-5421"[IS] OR "1545-2050"[IS] OR "1543-5938"[IS] OR "0378-5912"[IS] OR "0166-108X"[IS] OR "0306-3674"[IS] OR "1473-0480"[IS] OR "1872-6305"[IS] OR "1566-2535"[IS] OR "0033-3190"[IS] OR "1423-0348"[IS] OR "1936-086X"[IS] OR "1936-0851"[IS] OR "0734-306X"[IS] OR "1537-5307"[IS] OR "2666-6510"[IS] OR "2666-6510"[IS] OR "0903-1936"[IS] OR "1399-3003"[IS] OR "1471-4906"[IS] OR "1471-4981"[IS] OR "2327-0578"[IS] OR "2327-056X"[IS] OR "1552-7425"[IS] OR "1094-4281"[IS] OR "1078-0432"[IS] OR "1557-3265"[IS] OR "2472-3428"[IS] OR "2472-3428"[IS] OR "2375-2548"[IS] OR "2375-2548"[IS] OR "2665-9913"[IS] OR "2665-9913"[IS] OR "2211-1247"[IS] OR "2211-1247"[IS] OR "2637-9368"[IS] OR "2637-9368"[IS] OR "2589-5370"[IS] OR "2589-5370"[IS] OR "1873-7811"[IS] OR "0272-7358"[IS] OR "1741-3044"[IS] OR "0170-8406"[IS] OR "2045-9920"[IS] OR "2045-9939"[IS] OR "0009-7330"[IS] OR "1524-4571"[IS] OR "0022-2380"[IS] OR "1467-6486"[IS] OR "1556-6072"[IS] OR "1556-6072"[IS] OR "1361-6633"[IS] OR "0034-4885"[IS] OR "0360-0300"[IS] OR "1557-7341"[IS] OR "1533-3450"[IS] OR "1046-6673"[IS] OR "0162-8828"[IS] OR "0162-8828"[IS] OR "0001-4826"[IS] OR "0001-4826"[IS] OR "2662-1991"[IS] OR "2662-1991"[IS] OR "1460-2156"[IS] OR "0006-8950"[IS] OR "1936-878X"[IS] OR "1876-7591"[IS] OR "2589-5974"[IS] OR "2589-5974"[IS] OR "1461-023X"[IS] OR "1461-0248"[IS] OR "2199-2576"[IS] OR "2524-5317"[IS] OR "2691-3399"[IS] OR "2691-3399"[IS] OR "1368-7646"[IS] OR "1532-2084"[IS] OR "1469-185X"[IS] OR "1464-7931"[IS] OR "1867-2949"[IS] OR "1867-2957"[IS] OR "2575-0348"[IS] OR "2575-0356"[IS] OR "2662-8465"[IS] OR "2662-8465"[IS] OR "1468-0297"[IS] OR "0013-0133"[IS] OR "0018-9286"[IS] OR "1558-2523"[IS] OR "1048-9843"[IS] OR "1048-9843"[IS] OR "1042-2587"[IS] OR "1540-6520"[IS] OR "1741-315X"[IS] OR "1476-1270"[IS] OR "0033-8222"[IS] OR "0033-8222"[IS] OR "0890-8044"[IS] OR "1558-156X"[IS] OR "1362-4326"[IS] OR "0376-0004"[IS] OR "2050-084X"[IS] OR "2050-084X"[IS] OR "1082-989X"[IS] OR "1939-1463"[IS] OR "1866-3516"[IS] OR "1866-3508"[IS] OR "1545-326X"[IS] OR "0066-4219"[IS] OR "0066-426X"[IS] OR "1545-1593"[IS] OR "2333-2077"[IS] OR "2333-2050"[IS] OR "0002-953X"[IS] OR "1535-7228"[IS] OR "1549-1277"[IS] OR "1549-1676"[IS] OR "1573-7136"[IS] OR "1380-6653"[IS] OR "0360-0572"[IS] OR "1545-2115"[IS] OR "2155-5435"[IS] OR "2155-5435"[IS] OR "2056-5968"[IS] OR "2056-5968"[IS] OR "2352-4642"[IS] OR "2352-4642"[IS] OR "2374-7951"[IS] OR "2374-7943"[IS] OR "2162-8564"[IS] OR "0008-1256"[IS] OR "0270-9139"[IS] OR "1527-3350"[IS] OR "1560-7917"[IS] OR "1025-496X"[IS] OR "1931-9401"[IS] OR "1931-9401"[IS] OR "0010-8545"[IS] OR "0010-8545"[IS] OR "2574-3805"[IS] OR "2574-3805"[IS] OR "1540-1413"[IS] OR "1540-1405"[IS] OR "2334-2536"[IS] OR "2334-2536"[IS] OR "1094-9968"[IS] OR "1520-6653"[IS] OR "2198-3844"[IS] OR "2198-3844"[IS] OR "0033-8419"[IS] OR "1527-1315"[IS] OR "1365-2486"[IS] OR "1354-1013"[IS] OR "0034-4257"[IS] OR "0034-4257"[IS] OR "2159-6778"[IS] OR "2159-676X"[IS] OR "0010-3640"[IS] OR "1097-0312"[IS] OR "2198-6061"[IS] OR "2198-6061"[IS] OR "1091-6490"[IS] OR "0027-8424"[IS] OR "0007-2303"[IS] OR "1533-4465"[IS] OR "1460-2369"[IS] OR "1355-4786"[IS] OR "0963-8687"[IS] OR "0963-8687"[IS] OR "0001-5962"[IS] OR "0001-5962"[IS] OR "1523-2409"[IS] OR "1745-493X"[IS] OR "0393-2990"[IS] OR "1573-7284"[IS] OR "1469-896X"[IS] OR

	"0961-8368"[IS] OR "1544-9173"[IS] OR "1545-7885"[IS] OR "1551-3203"[IS] OR "1551-3203"[IS] OR "1537-6591"[IS] OR "1058-4838"[IS] OR "1759-2879"[IS] OR "1759-2887"[IS] OR "2352-3018"[IS] OR "2352-3018"[IS] OR "1531-8249"[IS] OR "0364-5134"[IS] OR "1467-9868"[IS] OR "1369-7412"[IS] OR "1053-5888"[IS] OR "1053-5888"[IS] OR "1757-4676"[IS] OR "1757-4684"[IS] OR "2077-7019"[IS] OR "2077-7000"[IS] OR "1549-4713"[IS] OR "0161-6420"[IS] OR "0163-4453"[IS] OR "1532-2742"[IS] OR "1545-4304"[IS] OR "0362-1642"[IS] OR "2326-8298"[IS] OR "2326-831X"[IS] OR "0167-8116"[IS] OR "0167-8116"[IS] OR "0007-1234"[IS] OR "1469-2112"[IS] OR "2573-5144"[IS] OR "2573-5144"[IS] OR "0090-6778"[IS] OR "1558-0857"[IS] OR "1939-4616"[IS] OR "1939-4608"[IS] OR "2051-6347"[IS] OR "2051-6355"[IS] OR "2059-7983"[IS] OR "2059-7983"[IS] OR "0085-2538"[IS] OR "1523-1755"[IS] OR "1748-0132"[IS] OR "1748-0132"[IS] OR "1943-0264"[IS] OR "1943-0264"[IS] OR "1539-3704"[IS] OR "0003-4819"[IS] OR "0012-8252"[IS] OR "0012-8252"[IS] OR "1099-1379"[IS] OR "0894-3796"[IS] OR "0022-3514"[IS] OR "0022-3514"[IS] OR "0276-7783"[IS] OR "2162-9730"[IS] OR "2326-5191"[IS] OR "2326-5205"[IS] OR "0022-4359"[IS] OR "0022-4359"[IS] OR "0006-3223"[IS] OR "1873-2402"[IS] OR "1552-3829"[IS] OR "0010-4140"[IS] OR "1931-2466"[IS] OR "1931-2458"[IS] OR "0031-5826"[IS] OR "1744-6570"[IS] OR "2327-4662"[IS] OR "2327-4662"[IS] OR "1949-3029"[IS] OR "1949-3029"[IS] OR "1097-6825"[IS] OR "0091-6749"[IS] OR "2041-417X"[IS] OR "2041-4161"[IS] OR "0105-2896"[IS] OR "1600-065X"[IS] OR "0885-8950"[IS] OR "0885-8950"[IS] OR "1098-5557"[IS] OR "1092-2172"[IS] OR "2049-2618"[IS] OR "2049-2618"[IS] OR "0955-0674"[IS] OR "1879-0410"[IS] OR "0022-1090"[IS] OR "1756-6916"[IS] OR "2044-5385"[IS] OR "2044-5385"[IS] OR "1557-7368"[IS] OR "0730-0301"[IS] OR "2047-217X"[IS] OR "2047-217X"[IS] OR "2165-8110"[IS] OR "2165-8102"[IS] OR "2168-2216"[IS] OR "2168-2232"[IS] OR "0360-1315"[IS] OR "0360-1315"[IS] OR "0272-6963"[IS] OR "0272-6963"[IS] OR "1532-6985"[IS] OR "0046-1520"[IS] OR "0955-534X"[IS] OR "0955-534X"[IS] OR "1540-8140"[IS] OR "0021-9525"[IS] OR "0890-8567"[IS] OR "1527-5418"[IS] OR "0005-1098"[IS] OR "0005-1098"[IS] OR "1532-298X"[IS] OR "1040-4651"[IS] OR "0048-7333"[IS] OR "0048-7333"[IS] OR "2366-9608"[IS] OR "2366-9608"[IS] OR "2168-6262"[IS] OR "2168-6254"[IS] OR "1751-7362"[IS] OR "1751-7370"[IS] OR "2666-7568"[IS] OR "2666-7568"[IS] OR "1942-2466"[IS] OR "1942-2466"[IS] OR "1477-0288"[IS] OR "0309-1325"[IS] OR "0304-4130"[IS] OR "1475-6765"[IS] OR "0305-7372"[IS] OR "0305-7372"[IS] OR "1198-743X"[IS] OR "1469-0691"[IS] OR "2542-5196"[IS] OR "2542-5196"[IS] OR "0168-9525"[IS] OR "1362-4555"[IS] OR "2405-8033"[IS] OR "2405-8033"[IS] OR "0890-6955"[IS] OR "0890-6955"[IS] OR "0030-364X"[IS] OR "1526-5463"[IS] OR "1466-609X"[IS] OR "1364-8535"[IS] OR "1464-3510"[IS] OR "0006-3444"[IS] OR "1040-726X"[IS] OR "1573-336X"[IS] OR "2667-1093"[IS] OR "2667-1107"[IS] OR "0261-5177"[IS] OR "0261-5177"[IS] OR "1350-9047"[IS] OR "1476-5403"[IS] OR "2567-3173"[IS] OR "2567-3173"[IS] OR "2326-6066"[IS] OR "2326-6074"[IS] OR "2398-6352"[IS] OR "2398-6352"[IS] OR "2369-9698"[IS] OR "2369-9698"[IS] OR "2042-0226"[IS] OR "1672-7681"[IS] OR "1476-5578"[IS] OR "1359-4184"[IS] OR "1530-6992"[IS] OR "1530-6984"[IS] OR "1063-6706"[IS] OR "1063-6706"[IS] OR "2001-3078"[IS] OR "2001-3078"[IS] OR "2662-1355"[IS] OR "2662-1355"[IS] OR "1540-9295"[IS] OR "1540-9309"[IS] OR "0018-7267"[IS] OR "1741-282X"[IS] OR "0278-2715"[IS] OR "1544-5208"[IS] OR "2042-5805"[IS] OR "2042-5805"[IS] OR "1878-4380"[IS] OR "0966-842X"[IS] OR "2666-6359"[IS] OR "2666-6340"[IS] OR "1759-4790"[IS] OR "1759-4804"[IS] OR "0906-6713"[IS] OR "1600-0757"[IS] OR "1554-8627"[IS] OR "1554-8627"[IS] OR "0190-9320"[IS] OR "1573-6687"[IS] OR "1522-9645"[IS] OR "0195-668X"[IS] OR "1741-7015"[IS] OR "1741-7015"[IS] OR "2162-237X"[IS] OR "2162-2388"[IS] OR "1531-7331"[IS] OR "1545-4118"[IS] OR "0031-8108"[IS] OR "0031-8108"[IS] OR "0160-7383"[IS] OR "0160-7383"[IS] OR "1525-0024"[IS] OR "1525-0016"[IS] OR "1537-1719"[IS] OR "0737-4038"[IS] OR "2639-4979"[IS] OR "2639-4979"[IS] OR "2332-7731"[IS] OR "2332-7731"[IS] OR "1567-2190"[IS] OR "1567-2328"[IS] OR "2639-0213"[IS] OR "2639-0213"[IS] OR "0749-5978"[IS] OR "1095-9920"[IS] OR "2051-1426"[IS] OR "2051-1426"[IS] OR "0162-1459"[IS] OR "1537-274X"[IS] OR "1613-6829"[IS] OR "1613-6810"[IS] OR "0022-1996"[IS] OR "0022-1996"[IS] OR "1226-3613"[IS] OR "2092-6413"[IS] OR "0066-4286"[IS] OR "1545-2107"[IS] OR "0887-6924"[IS] OR "1476-5551"[IS] OR "0019-7939"[IS] OR "2162-271X"[IS] OR "0920-5691"[IS] OR "1573-1405"[IS] OR "1674-8018"[IS] OR "1674-800X"[IS] OR "0003-066X"[IS] OR "1935-990X"[IS] OR "0895-4356"[IS] OR "1878-5921"[IS] OR "1435-9855"[IS] OR "1435-9863"[IS] OR "0012-186X"[IS] OR "1432-0428"[IS] OR "1367-5788"[IS] OR "1367-5788"[IS] OR "0885-8993"[IS] OR "0885-8993"[IS] OR "0958-9465"[IS] OR "0958-9465"[IS] OR "0043-1354"[IS] OR "1879-2448"[IS] OR "1477-9803"[IS] OR "1053-1858"[IS] OR "1548-8004"[IS] OR "0022-166X"[IS] OR "1096-7516"[IS] OR "1096-7516"[IS] OR "2292-8782"[IS] OR "2292-8782"[IS] OR "1759-7323"[IS] OR "1759-7331"[IS] OR "0033-3352"[IS] OR "1540-6210"[IS] OR "0924-2716"[IS] OR "0924-2716"[IS] OR "2056-6387"[IS] OR "2056-6387"[IS] OR "0013-189X"[IS] OR "0013-189X"[IS] OR "2056-3744"[IS] OR "2056-3744"[IS] OR "0112-1642"[IS] OR "1179-2035"[IS] OR "1552-5279"[IS] OR "1552-5260"[IS] OR "1551-3068"[IS] OR "1551-305X"[IS] OR "2379-3708"[IS] OR "2379-3708"[IS] OR "0012-9615"[IS] OR "0012-9615"[IS] OR "1469-3178"[IS] OR "1469-221X"[IS] OR "0031-5850"[IS] OR "0031-5850"[IS] OR "1747-938X"[IS] OR "1747-938X"[IS] OR "2397-3366"[IS] OR "2397-3366"[IS] OR "0743-9156"[IS] OR

	"1547-7207"[IS] OR "1477-870X"[IS] OR "0035-9009"[IS] OR "1526-5536"[IS] OR "1047-7047"[IS] OR "2666-1381"[IS] OR "2666-1381"[IS] OR "1552-6763"[IS] OR "0047-2875"[IS] OR "2308-216X"[IS] OR "2308-2151"[IS] OR "0168-6445"[IS] OR "1574-6976"[IS] OR "1090-9516"[IS] OR "1090-9516"[IS] OR "0098-2997"[IS] OR "1872-9452"[IS] OR "1573-7020"[IS] OR "1381-4338"[IS] OR "1364-0321"[IS] OR "1364-0321"[IS] OR "1540-5885"[IS] OR "0737-6782"[IS] OR "2329-9266"[IS] OR "2329-9274"[IS] OR "1547-7398"[IS] OR "0012-7094"[IS] OR "1941-3297"[IS] OR "1941-3289"[IS] OR "1360-1385"[IS] OR "1878-4372"[IS] OR "1879-033X"[IS] OR "0959-440X"[IS] OR "1879-0356"[IS] OR "1369-5266"[IS] OR "0950-6608"[IS] OR "1743-2804"[IS] OR "0191-2615"[IS] OR "0191-2615"[IS] OR "0167-5729"[IS] OR "1879-274X"[IS] OR "1756-2171"[IS] OR "0741-6261"[IS] OR "2397-4621"[IS] OR "2397-4621"[IS] OR "2059-0105"[IS] OR "2059-0105"[IS] OR "1361-8415"[IS] OR "1361-8423"[IS] OR "0278-0046"[IS] OR "0278-0046"[IS] OR "2772-4247"[IS] OR "2772-4247"[IS] OR "0361-476X"[IS] OR "1090-2384"[IS] OR "1076-836X"[IS] OR "1063-5157"[IS] OR "1468-330X"[IS] OR "0022-3050"[IS] OR "1932-4391"[IS] OR "1932-443X"[IS] OR "2667-193X"[IS] OR "2667-193X"[IS] OR "1873-2194"[IS] OR "0163-7827"[IS] OR "2050-7496"[IS] OR "2050-7488"[IS] OR "1863-8880"[IS] OR "1863-8899"[IS] OR "0304-3878"[IS] OR "0304-3878"[IS] OR "2158-1592"[IS] OR "0735-3766"[IS] OR "1468-3296"[IS] OR "0040-6376"[IS] OR "1554-0669"[IS] OR "1554-0677"[IS] OR "0013-936X"[IS] OR "1520-5851"[IS] OR "1059-1478"[IS] OR "1937-5956"[IS] OR "1079-7114"[IS] OR "0031-9007"[IS] OR "1750-6824"[IS] OR "1750-6816"[IS] OR "1096-3650"[IS] OR "1044-579X"[IS] OR "1536-867X"[IS] OR "1536-867X"[IS] OR "1053-4822"[IS] OR "1053-4822"[IS] OR "1743-7202"[IS] OR "1743-7199"[IS] OR "0091-1798"[IS] OR "0091-1798"[IS] OR "2666-5549"[IS] OR "2666-5549"[IS] OR "2162-2345"[IS] OR "2162-2337"[IS] OR "1538-4365"[IS] OR "0067-0049"[IS] OR "1538-7836"[IS] OR "1538-7933"[IS] OR "1755-263X"[IS] OR "1755-263X"[IS] OR "2333-5963"[IS] OR "2333-5955"[IS] OR "1095-9068"[IS] OR "0094-1190"[IS] OR "1873-6750"[IS] OR "0160-4120"[IS] OR "2052-2525"[IS] OR "2052-2525"[IS] OR "2473-9529"[IS] OR "2473-9537"[IS] OR "0002-9378"[IS] OR "1097-6868"[IS] OR "1557-928X"[IS] OR "0742-1222"[IS] OR "1879-016X"[IS] OR "0163-7258"[IS] OR "1058-4609"[IS] OR "1091-7675"[IS] OR "2227-8508"[IS] OR "2226-4108"[IS] OR "2573-9581"[IS] OR "2573-959X"[IS] OR "1532-2955"[IS] OR "1087-0792"[IS] OR "1080-6040"[IS] OR "1080-6059"[IS] OR "1468-2508"[IS] OR "0022-3816"[IS] OR "1469-8137"[IS] OR "0028-646X"[IS] OR "1558-173X"[IS] OR "0018-9200"[IS] OR "0003-9527"[IS] OR "1432-0673"[IS] OR "0140-9883"[IS] OR "1873-6181"[IS] OR "1090-2139"[IS] OR "0889-1591"[IS] OR "2096-5745"[IS] OR "2096-5745"[IS] OR "1467-8721"[IS] OR "0963-7214"[IS] OR "0925-5273"[IS] OR "0925-5273"[IS] OR "0959-4388"[IS] OR "1873-6882"[IS] OR "0021-9630"[IS] OR "1469-7610"[IS] OR "1420-8970"[IS] OR "1016-443X"[IS] OR "0014-4983"[IS] OR "0014-4983"[IS] OR "1532-8007"[IS] OR "1070-5511"[IS] OR "0823-9150"[IS] OR "0823-9150"[IS] OR "1568-1637"[IS] OR "1568-1637"[IS] OR "1366-5545"[IS] OR "1366-5545"[IS] OR "1095-7200"[IS] OR "0036-1445"[IS] OR "0954-6820"[IS] OR "1365-2796"[IS] OR "1432-1475"[IS] OR "0933-1433"[IS] OR "1941-3084"[IS] OR "1941-3149"[IS] OR "2590-3462"[IS] OR "2590-3462"[IS] OR "0091-732X"[IS] OR "1935-1038"[IS] OR "0008-5472"[IS] OR "1538-7445"[IS] OR "0169-409X"[IS] OR "1872-8294"[IS] OR "1532-7663"[IS] OR "1057-7408"[IS] OR "2057-3960"[IS] OR "2057-3960"[IS] OR "0020-7543"[IS] OR "1366-588X"[IS] OR "1471-499X"[IS] OR "1471-4914"[IS] OR "2095-5138"[IS] OR "2053-714X"[IS] OR "1757-7799"[IS] OR "1757-7780"[IS] OR "2041-3866"[IS] OR "2041-3874"[IS] OR "1523-6838"[IS] OR "0272-6386"[IS] OR "2574-8300"[IS] OR "2574-8300"[IS] OR "0966-9582"[IS] OR "1747-7646"[IS] OR "0007-6503"[IS] OR "1552-4205"[IS] OR "1873-3735"[IS] OR "0165-6147"[IS] OR "1467-8667"[IS] OR "1093-9687"[IS] OR "2235-1795"[IS] OR "1664-5553"[IS] OR "0004-5411"[IS] OR "1557-735X"[IS] OR "0022-040X"[IS] OR "1945-743X"[IS] OR "1528-1140"[IS] OR "0003-4932"[IS] OR "2644-125X"[IS] OR "2644-125X"[IS] OR "0959-3780"[IS] OR "1872-9495"[IS] OR "1367-4803"[IS] OR "1460-2059"[IS] OR "0038-0717"[IS] OR "0038-0717"[IS] OR "0012-9593"[IS] OR "0012-9593"[IS] OR "1542-7714"[IS] OR "1542-3565"[IS] OR "0278-4319"[IS] OR "0278-4319"[IS] OR "1523-9829"[IS] OR "1545-4274"[IS] OR "1359-6454"[IS] OR "1359-6454"[IS] OR "1095-9084"[IS] OR "0001-8791"[IS] OR "2096-4579"[IS] OR "2096-4579"[IS] OR "2328-8930"[IS] OR "2328-8930"[IS] OR "1876-7605"[IS] OR "1936-8798"[IS] OR "0306-2619"[IS] OR "0306-2619"[IS] OR "2589-5559"[IS] OR "2589-5559"[IS] OR "1757-7012"[IS] OR "1757-7004"[IS] OR "2095-9273"[IS] OR "2095-9281"[IS] OR "0361-8609"[IS] OR "1096-8652"[IS] OR "2352-3964"[IS] OR "2352-3964"[IS] OR "1547-6537"[IS] OR "1064-3389"[IS] OR "0959-8049"[IS] OR "1879-0852"[IS] OR "0144-1647"[IS] OR "1464-5327"[IS] OR "1359-8546"[IS] OR "1359-8546"[IS] OR "0148-2963"[IS] OR "0148-2963"[IS] OR "1759-0876"[IS] OR "1759-0884"[IS] OR "0026-0495"[IS] OR "1532-8600"[IS] OR "0002-9602"[IS] OR "1537-5390"[IS] OR "0968-090X"[IS] OR "0968-090X"[IS] OR "0376-0421"[IS] OR "0376-0421"[IS] OR "0736-5845"[IS] OR "0736-5845"[IS] OR "2590-0056"[IS] OR "2590-0056"[IS] OR "1757-5818"[IS] OR "1757-5818"[IS] OR "1095-7235"[IS] OR "0022-0531"[IS] OR "0278-0062"[IS] OR "1558-254X"[IS] OR "1099-0836"[IS] OR "0964-4733"[IS] OR "2397-3722"[IS] OR "2397-3722"[IS] OR "1520-5002"[IS] OR "0897-4756"[IS] OR "1672-0229"[IS] OR "1672-0229"[IS] OR "0890-2070"[IS] OR "1099-0984"[IS] OR "0959-8146"[IS] OR "1756-1833"[IS] OR "1532-1827"[IS] OR "0007-0920"[IS] OR "1873-9946"[IS] OR "1873-9946"[IS] OR "0031-4005"[IS] OR

	"1098-4275"[IS] OR "0065-9266"[IS] OR "0065-9266"[IS] OR "1748-5908"[IS] OR "1748-5908"[IS] OR "2169-5172"[IS] OR "2169-5172"[IS] OR "1460-8545"[IS] OR "1468-2370"[IS] OR "0956-7976"[IS] OR "1467-9280"[IS] OR "1040-9238"[IS] OR "1549-7798"[IS] OR "0024-6301"[IS] OR "1873-1872"[IS] OR "0887-6185"[IS] OR "1873-7897"[IS] OR "2378-2242"[IS] OR "2378-2242"[IS] OR "1750-8606"[IS] OR "1750-8592"[IS] OR "0749-6419"[IS] OR "0749-6419"[IS] OR "1389-5567"[IS] OR "1389-5567"[IS] OR "2040-7122"[IS] OR "2040-7122"[IS] OR "1945-0877"[IS] OR "1937-9145"[IS] OR "0887-378X"[IS] OR "1468-0009"[IS] OR "1526-5447"[IS] OR "0041-1655"[IS] OR "1387-6473"[IS] OR "1387-6473"[IS] OR "1567-2409"[IS] OR "1567-2395"[IS] OR "1948-206X"[IS] OR "2157-5045"[IS] OR "2222-1751"[IS] OR "2222-1751"[IS] OR "1464-3685"[IS] OR "0300-5771"[IS] OR "2405-4283"[IS] OR "2405-4283"[IS] OR "2212-8778"[IS] OR "2212-8778"[IS] OR "1879-0445"[IS] OR "0960-9822"[IS] OR "1046-3283"[IS] OR "1479-277X"[IS] OR "2041-210X"[IS] OR "2041-210X"[IS] OR "1532-1681"[IS] OR "0268-960X"[IS] OR "1385-8947"[IS] OR "1385-8947"[IS] OR "0018-9545"[IS] OR "1939-9359"[IS] OR "0021-7824"[IS] OR "0021-7824"[IS] OR "1939-1471"[IS] OR "0033-295X"[IS] OR "1079-5642"[IS] OR "1524-4636"[IS] OR "1935-3456"[IS] OR "1933-0219"[IS] OR "0269-2813"[IS] OR "1365-2036"[IS] OR "0090-3493"[IS] OR "1530-0293"[IS] OR "0093-6502"[IS] OR "1552-3810"[IS] OR "1600-6135"[IS] OR "1600-6143"[IS] OR "1467-7644"[IS] OR "1467-7652"[IS] OR "1572-9672"[IS] OR "0038-6308"[IS] OR "1759-7684"[IS] OR "1759-7692"[IS] OR "2542-5293"[IS] OR "2542-5293"[IS] OR "2168-6068"[IS] OR "2168-6084"[IS] OR "2330-6696"[IS] OR "2330-6696"[IS] OR "2041-6520"[IS] OR "2041-6539"[IS] OR "0194-911X"[IS] OR "1524-4563"[IS] OR "2095-4956"[IS] OR "2095-4956"[IS] OR "1554-3528"[IS] OR "1554-351X"[IS] OR "1350-1917"[IS] OR "1365-2575"[IS] OR "0198-6325"[IS] OR "1098-1128"[IS] OR "1524-4628"[IS] OR "0039-2499"[IS] OR "1098-3600"[IS] OR "1530-0366"[IS] OR "0278-6125"[IS] OR "0278-6125"[IS] OR "1474-9726"[IS] OR "1474-9718"[IS] OR "2452-199X"[IS] OR "2452-199X"[IS] OR "1468-0068"[IS] OR "0029-4624"[IS] OR "2396-9881"[IS] OR "2396-9873"[IS] OR "0008-543X"[IS] OR "1097-0142"[IS] OR "0105-4538"[IS] OR "1398-9995"[IS] OR "1573-0913"[IS] OR "0921-898X"[IS] OR "0033-5835"[IS] OR "1469-8994"[IS] OR "0278-3649"[IS] OR "1741-3176"[IS] OR "1672-9072"[IS] OR "1744-7909"[IS] OR "2374-4650"[IS] OR "2374-4642"[IS] OR "2041-8213"[IS] OR "2041-8205"[IS] OR "2452-414X"[IS] OR "2452-414X"[IS] OR "0083-2057"[IS] OR "2333-0872"[IS] OR "1548-7660"[IS] OR "1548-7660"[IS] OR "0167-7799"[IS] OR "1879-3096"[IS] OR "0142-9612"[IS] OR "1878-5905"[IS] OR "1552-3098"[IS] OR "1552-3098"[IS] OR "2196-1115"[IS] OR "2196-1115"[IS] OR "1557-3117"[IS] OR "1053-2498"[IS] OR "1479-5868"[IS] OR "1479-5868"[IS] OR "2053-1680"[IS] OR "2053-1680"[IS] OR "2521-327X"[IS] OR "2521-327X"[IS] OR "1467-789X"[IS] OR "1467-7881"[IS] OR "0952-7915"[IS] OR "0952-7915"[IS] OR "1600-0617"[IS] OR "0905-9180"[IS] OR "1531-4804"[IS] OR "0162-2889"[IS] OR "1195-1982"[IS] OR "1708-8305"[IS] OR "0378-7206"[IS] OR "0378-7206"[IS] OR "1574-0137"[IS] OR "1574-0137"[IS] OR "1879-0658"[IS] OR "0921-3449"[IS] OR "0305-0483"[IS] OR "0305-0483"[IS] OR "2523-3548"[IS] OR "2523-3548"[IS] OR "1558-0016"[IS] OR "1524-9050"[IS] OR "0043-8871"[IS] OR "1086-3338"[IS] OR "2397-7132"[IS] OR "2397-7132"[IS] OR "1873-5118"[IS] OR "0301-0082"[IS] OR "0019-8501"[IS] OR "0019-8501"[IS] OR "0304-419X"[IS] OR "0304-419X"[IS] OR "2524-7921"[IS] OR "2524-793X"[IS] OR "1556-6013"[IS] OR "1556-6013"[IS] OR "1044-5323"[IS] OR "1096-3618"[IS] OR "0735-2751"[IS] OR "1467-9558"[IS] OR "1758-9193"[IS] OR "1758-9193"[IS] OR "0273-2297"[IS] OR "1090-2406"[IS] OR "2444-569X"[IS] OR "2530-7614"[IS] OR "2096-8663"[IS] OR "2667-0054"[IS] OR "0166-3615"[IS] OR "0166-3615"[IS] OR "2210-6502"[IS] OR "2210-6502"[IS] OR "0090-0036"[IS] OR "1541-0048"[IS] OR "0040-1625"[IS] OR "0040-1625"[IS] OR "0199-9885"[IS] OR "1545-4312"[IS] OR "2168-6785"[IS] OR "2168-6777"[IS] OR "0012-1797"[IS] OR "0012-1797"[IS] OR "2167-082X"[IS] OR "2167-0811"[IS] OR "2214-8604"[IS] OR "2214-8604"[IS] OR "1932-4553"[IS] OR "1932-4553"[IS] OR "1478-0895"[IS] OR "1478-0887"[IS] OR "1873-1899"[IS] OR "0734-9750"[IS] OR "2452-302X"[IS] OR "2452-302X"[IS] OR "1879-0305"[IS] OR "1359-6101"[IS] OR "1758-6593"[IS] OR "0144-3577"[IS] OR "2367-0983"[IS] OR "2367-2617"[IS] OR "1052-9276"[IS] OR "1099-1654"[IS] OR "2169-4796"[IS] OR "2168-3492"[IS] OR "2590-3330"[IS] OR "2590-3322"[IS] OR "2110-5820"[IS] OR "2110-5820"[IS] OR "2377-2999"[IS] OR "2377-2999"[IS] OR "0162-895X"[IS] OR "1467-9221"[IS] OR "1557-7805"[IS] OR "0091-3367"[IS] OR "0021-9916"[IS] OR "1460-2466"[IS] OR "2157-6912"[IS] OR "2157-6904"[IS] OR "2049-3169"[IS] OR "1674-2818"[IS] OR "1941-1359"[IS] OR "1941-1340"[IS] OR "1934-8525"[IS] OR "1934-8533"[IS] OR "0885-3185"[IS] OR "1531-8257"[IS] OR "1949-0984"[IS] OR "1949-0976"[IS] OR "1873-7528"[IS] OR "0149-7634"[IS] OR "2666-3864"[IS] OR "2666-3864"[IS] OR "1530-0285"[IS] OR "0893-3952"[IS] OR "0007-1250"[IS] OR "1472-1465"[IS] OR "2588-9311"[IS] OR "2588-9311"[IS] OR "1743-9191"[IS] OR "1743-9159"[IS] OR "1470-1340"[IS] OR "1461-6688"[IS] OR "1095-9157"[IS] OR "0896-8411"[IS] OR "1755-098X"[IS] OR "1755-0998"[IS] OR "1573-0697"[IS] OR "0167-4544"[IS] OR "2049-8470"[IS] OR "2049-8489"[IS] OR "1096-6099"[IS] OR "1094-2025"[IS] OR "1744-8603"[IS] OR "1744-8603"[IS] OR "1756-8935"[IS] OR "1756-8935"[IS] OR "2572-4568"[IS] OR "2572-4568"[IS] OR "0268-005X"[IS] OR "0268-005X"[IS] OR "2095-4700"[IS] OR "2095-6231"[IS] OR "1091-4269"[IS] OR "1520-6394"[IS] OR "0194-4363"[IS] OR "0194-4363"[IS] OR "0960-7692"[IS] OR "1469-0705"[IS] OR "2047-9158"[IS] OR

	"2047-9158"[IS] OR "0304-3894"[IS] OR "0304-3894"[IS] OR "1541-4337"[IS] OR "1541-4337"[IS] OR "1000-0747"[IS] OR "1000-0747"[IS] OR "0024-3892"[IS] OR "0024-3892"[IS] OR "2590-1168"[IS] OR "2590-1168"[IS] OR "2405-5018"[IS] OR "2405-500X"[IS] OR "1592-8721"[IS] OR "0390-6078"[IS] OR "1937-3333"[IS] OR "1941-1189"[IS] OR "1564-6971"[IS] OR "0257-3032"[IS] OR "2313-1691"[IS] OR "2214-2584"[IS] OR "1568-9972"[IS] OR "1873-0183"[IS] OR "1548-9213"[IS] OR "1548-9221"[IS] OR "1872-2067"[IS] OR "0253-9837"[IS] OR "1573-0476"[IS] OR "0895-5646"[IS] OR "0969-6989"[IS] OR "0969-6989"[IS] OR "2451-9448"[IS] OR "2451-9456"[IS] OR "1536-1233"[IS] OR "1536-1233"[IS] OR "1096-9896"[IS] OR "0022-3417"[IS] OR "2168-6831"[IS] OR "2168-6831"[IS] OR "1941-7705"[IS] OR "1941-7713"[IS] OR "0028-3878"[IS] OR "1526-632X"[IS] OR "0360-5302"[IS] OR "1532-4133"[IS] OR "2522-0128"[IS] OR "2522-0136"[IS] OR "2096-0654"[IS] OR "2096-0654"[IS] OR "2166-3831"[IS] OR "2166-3831"[IS] OR "0969-2126"[IS] OR "1878-4186"[IS] OR "2195-1071"[IS] OR "2195-1071"[IS] OR "1672-7703"[IS] OR "1672-7703"[IS] OR "1096-0449"[IS] OR "0095-0696"[IS] OR "0924-2244"[IS] OR "0924-2244"[IS] OR "1021-7770"[IS] OR "1423-0127"[IS] OR "1936-8631"[IS] OR "1936-8623"[IS] OR "2397-768X"[IS] OR "2397-768X"[IS] OR "0267-8373"[IS] OR "1464-5335"[IS] OR "2213-2317"[IS] OR "2213-2317"[IS] OR "2211-3843"[IS] OR "2211-3835"[IS] OR "1573-353X"[IS] OR "0889-3268"[IS] OR "0178-8051"[IS] OR "1432-2064"[IS] OR "1367-5931"[IS] OR "1879-0402"[IS] OR "0196-8904"[IS] OR "0196-8904"[IS] OR "0079-6727"[IS] OR "0079-6727"[IS] OR "1095-9572"[IS] OR "1053-8119"[IS] OR "1939-2176"[IS] OR "0022-0663"[IS] OR "1535-1645"[IS] OR "1523-3812"[IS] OR "2328-4277"[IS] OR "2328-4277"[IS] OR "1525-2019"[IS] OR "1525-2019"[IS] OR "0066-4812"[IS] OR "1467-8330"[IS] OR "0305-750X"[IS] OR "1873-5991"[IS] OR "1941-0476"[IS] OR "1053-587X"[IS] OR "1884-4057"[IS] OR "1884-4049"[IS] OR "0959-6119"[IS] OR "0959-6119"[IS] OR "2380-8977"[IS] OR "2380-8985"[IS] OR "1745-9125"[IS] OR "0011-1384"[IS] OR "1099-1255"[IS] OR "0883-7252"[IS] OR "0021-4027"[IS] OR "0021-4027"[IS] OR "1558-3724"[IS] OR "1558-3716"[IS] OR "0269-2821"[IS] OR "1573-7462"[IS] OR "1553-7374"[IS] OR "1553-7366"[IS] OR "0007-0912"[IS] OR "1471-6771"[IS] OR "2332-7782"[IS] OR "2332-7782"[IS] OR "1998-0124"[IS] OR "1998-0000"[IS] OR "2055-6764"[IS] OR "2055-6756"[IS] OR "1755-3245"[IS] OR "0008-6363"[IS] OR "0960-085X"[IS] OR "1476-9344"[IS] OR "0029-7844"[IS] OR "1873-233X"[IS] OR "2667-0968"[IS] OR "2667-0968"[IS] OR "1364-0380"[IS] OR "1465-3060"[IS] OR "1939-1307"[IS] OR "1076-8998"[IS] OR "0363-5465"[IS] OR "1552-3365"[IS] OR "0007-6813"[IS] OR "0007-6813"[IS] OR "0888-3270"[IS] OR "1096-1216"[IS] OR "1365-2044"[IS] OR "0003-2409"[IS] OR "1873-1430"[IS] OR "0294-1449"[IS] OR "0960-8524"[IS] OR "1873-2976"[IS] OR "1359-0286"[IS] OR "1359-0286"[IS] OR "1477-9129"[IS] OR "0950-1991"[IS] OR "0097-5397"[IS] OR "1095-7111"[IS] OR "1573-2827"[IS] OR "1096-4037"[IS] OR "1469-8978"[IS] OR "0033-2917"[IS] OR "0747-5632"[IS] OR "0747-5632"[IS] OR "2213-6711"[IS] OR "2213-6711"[IS] OR "1680-7324"[IS] OR "1680-7316"[IS] OR "1540-4781"[IS] OR "0026-7902"[IS] OR "0091-7451"[IS] OR "1943-4456"[IS] OR "1863-2297"[IS] OR "1863-2300"[IS] OR "1600-0854"[IS] OR "1398-9219"[IS] OR "2662-4435"[IS] OR "2662-4435"[IS] OR "0969-5931"[IS] OR "0969-5931"[IS] OR "2352-345X"[IS] OR "2352-345X"[IS] OR "2524-485X"[IS] OR "2524-4868"[IS] OR "2164-5760"[IS] OR "2164-5744"[IS] OR "0140-2382"[IS] OR "1743-9655"[IS] OR "1466-8238"[IS] OR "1466-822X"[IS] OR "1872-6623"[IS] OR "0304-3959"[IS] OR "1600-0447"[IS] OR "0001-690X"[IS] OR "0022-3433"[IS] OR "0022-3433"[IS] OR "0926-5805"[IS] OR "0926-5805"[IS] OR "2213-9567"[IS] OR "2213-9567"[IS] OR "2666-0873"[IS] OR "2666-0873"[IS] OR "1527-974X"[IS] OR "1067-5027"[IS] OR "1742-2094"[IS] OR "1742-2094"[IS] OR "1556-603X"[IS] OR "1556-603X"[IS] OR "2050-5086"[IS] OR "2050-5086"[IS] OR "1540-1979"[IS] OR "1540-1960"[IS] OR "0026-4423"[IS] OR "1460-2113"[IS] OR "2638-6100"[IS] OR "2638-6100"[IS] OR "1555-7561"[IS] OR "1555-7561"[IS] OR "1572-0241"[IS] OR "0002-9270"[IS] OR "0079-6565"[IS] OR "1873-3301"[IS] OR "1524-8380"[IS] OR "1552-8324"[IS] OR "0022-2836"[IS] OR "1089-8638"[IS] OR "1525-3244"[IS] OR "0006-3568"[IS] OR "1520-0477"[IS] OR "0003-0007"[IS] OR "2210-4224"[IS] OR "2210-4224"[IS] OR "0012-821X"[IS] OR "0012-821X"[IS] OR "0020-9996"[IS] OR "1536-0210"[IS] OR "1756-9966"[IS] OR "0392-9078"[IS] OR "1469-3062"[IS] OR "1752-7457"[IS] OR "1940-1612"[IS] OR "1940-1620"[IS] OR "0361-3682"[IS] OR "0361-3682"[IS] OR "1569-1705"[IS] OR "1572-9826"[IS] OR "2052-4463"[IS] OR "2052-4463"[IS] OR "1864-3361"[IS] OR "1864-3361"[IS] OR "0166-4972"[IS] OR "0166-4972"[IS] OR "0022-1465"[IS] OR "2150-6000"[IS] OR "1465-542X"[IS] OR "1465-5411"[IS] OR "1600-051X"[IS] OR "0303-6979"[IS] OR "1948-5514"[IS] OR "1948-5506"[IS] OR "1558-0644"[IS] OR "0196-2892"[IS] OR "0959-4752"[IS] OR "0959-4752"[IS] OR "0091-7613"[IS] OR "1943-2682"[IS] OR "2051-5960"[IS] OR "2051-5960"[IS] OR "1080-0549"[IS] OR "1559-0267"[IS] OR "0305-9006"[IS] OR "0305-9006"[IS] OR "2053-9517"[IS] OR "2053-9517"[IS] OR "1742-4933"[IS] OR "1742-4933"[IS] OR "0586-7614"[IS] OR "1745-1701"[IS] OR "0022-1899"[IS] OR "1537-6613"[IS] OR "1741-3737"[IS] OR "0022-2445"[IS] OR "0893-133X"[IS] OR "1470-634X"[IS] OR "1564-0604"[IS] OR "0042-9686"[IS] OR "1095-8592"[IS] OR "1084-8045"[IS] OR "1432-0835"[IS] OR "0944-2669"[IS] OR "1838-7640"[IS] OR "1838-7640"[IS] OR "2050-7771"[IS] OR "2050-7771"[IS] OR "2515-5091"[IS] OR "2515-5091"[IS] OR "2059-7908"[IS] OR "2059-7908"[IS] OR "0894-1491"[IS] OR "1098-1136"[IS] OR "2325-5870"[IS] OR "2325-5870"[IS] OR "2043-8206"[IS] OR

	"2043-8214"[IS] OR "1520-9156"[IS] OR "1557-8593"[IS] OR "0003-1305"[IS] OR "1537-2731"[IS] OR "1600-0587"[IS] OR "0906-7590"[IS] OR "1359-107X"[IS] OR "2044-8287"[IS] OR "1420-682X"[IS] OR "1420-9071"[IS] OR "0377-2217"[IS] OR "0377-2217"[IS] OR "1449-4035"[IS] OR "1449-4035"[IS] OR "1554-0634"[IS] OR "1554-0626"[IS] OR "1759-8753"[IS] OR "1759-8753"[IS] OR "1553-7390"[IS] OR "1553-7404"[IS] OR "1475-2840"[IS] OR "1475-2840"[IS] OR "0010-437X"[IS] OR "1570-5846"[IS] OR "1098-660X"[IS] OR "0095-1137"[IS] OR "2662-8457"[IS] OR "2662-8457"[IS] OR "1057-7149"[IS] OR "1057-7149"[IS] OR "1041-4347"[IS] OR "1041-4347"[IS] OR "0270-6474"[IS] OR "1529-2401"[IS] OR "2168-6173"[IS] OR "2168-6165"[IS] OR "1475-1461"[IS] OR "1475-1461"[IS] OR "0013-7952"[IS] OR "0013-7952"[IS] OR "2058-9565"[IS] OR "2058-9565"[IS] OR "1934-3671"[IS] OR "1934-368X"[IS] OR "0090-4848"[IS] OR "1099-050X"[IS] OR "2588-8420"[IS] OR "2588-8420"[IS] OR "0022-4871"[IS] OR "1552-7816"[IS] OR "2057-1577"[IS] OR "2057-1577"[IS] OR "0038-0407"[IS] OR "1939-8573"[IS] OR "0263-7863"[IS] OR "0263-7863"[IS] OR "2156-5376"[IS] OR "2161-8313"[IS] OR "1471-6372"[IS] OR "0022-0507"[IS] OR "0969-2290"[IS] OR "1466-4526"[IS] OR "0964-4563"[IS] OR "1468-3318"[IS] OR "1942-4795"[IS] OR "1942-4787"[IS] OR "1479-8387"[IS] OR "1479-8387"[IS] OR "1741-7007"[IS] OR "1741-7007"[IS] OR "2045-7979"[IS] OR "2045-7960"[IS] OR "1697-2600"[IS] OR "1697-2600"[IS] OR "0012-3692"[IS] OR "1931-3543"[IS] OR "1045-5736"[IS] OR "1086-3214"[IS] OR "0740-624X"[IS] OR "0740-624X"[IS] OR "0954-5395"[IS] OR "0954-5395"[IS] OR "1558-2221"[IS] OR "0018-926X"[IS] OR "1060-3743"[IS] OR "1060-3743"[IS] OR "1079-5014"[IS] OR "1758-5368"[IS] OR "1476-5594"[IS] OR "0950-9232"[IS] OR "0075-4102"[IS] OR "1435-5345"[IS] OR "1355-8382"[IS] OR "1469-9001"[IS] OR "2471-2825"[IS] OR "2471-2825"[IS] OR "2666-9161"[IS] OR "2666-9161"[IS] OR "2210-6707"[IS] OR "2210-6707"[IS] OR "2631-7990"[IS] OR "2631-8644"[IS] OR "2631-9268"[IS] OR "2631-9268"[IS] OR "1043-2760"[IS] OR "1879-3061"[IS] OR "0734-371X"[IS] OR "0734-371X"[IS] OR "2325-6621"[IS] OR "2325-6621"[IS] OR "1359-8368"[IS] OR "1359-8368"[IS] OR "2190-4979"[IS] OR "2190-4987"[IS] OR "1941-7632"[IS] OR "1941-7640"[IS] OR "1610-3653"[IS] OR "1610-3653"[IS] OR "1947-5438"[IS] OR "1947-5446"[IS] OR "0003-6900"[IS] OR "1088-8535"[IS] OR "1555-905X"[IS] OR "1555-9041"[IS] OR "1573-6938"[IS] OR "1386-4157"[IS] OR "2639-5274"[IS] OR "2096-5168"[IS] OR "0301-4215"[IS] OR "0301-4215"[IS] OR "2332-7812"[IS] OR "2332-7812"[IS] OR "0020-0255"[IS] OR "0020-0255"[IS] OR "0265-0568"[IS] OR "1460-4752"[IS] OR "0002-9599"[IS] OR "0002-9599"[IS] OR "1342-937X"[IS] OR "1342-937X"[IS] OR "2161-2129"[IS] OR "2150-7511"[IS] OR "1052-6234"[IS] OR "1095-7189"[IS] OR "1533-7928"[IS] OR "1532-4435"[IS] OR "1436-4646"[IS] OR "0025-5610"[IS] OR "0269-994X"[IS] OR "1464-0597"[IS] OR "0306-6150"[IS] OR "1743-9361"[IS] OR "0929-1199"[IS] OR "0929-1199"[IS] OR "1050-8406"[IS] OR "1532-7809"[IS] OR "0959-437X"[IS] OR "1879-0380"[IS] OR "1936-1335"[IS] OR "1936-1327"[IS] OR "0020-7136"[IS] OR "1097-0215"[IS] OR "0266-2426"[IS] OR "0266-2426"[IS] OR "1547-5271"[IS] OR "1556-3871"[IS] OR "2047-2412"[IS] OR "2047-2404"[IS] OR "0007-0963"[IS] OR "1365-2133"[IS] OR "0049-1241"[IS] OR "1552-8294"[IS] OR "2399-3642"[IS] OR "2399-3642"[IS] OR "1552-9924"[IS] OR "0091-6765"[IS] OR "1465-4644"[IS] OR "1468-4357"[IS] OR "2666-4984"[IS] OR "2666-4984"[IS] OR "1751-2409"[IS] OR "1751-2395"[IS] OR "2049-1948"[IS] OR "2049-1948"[IS] OR "1520-0442"[IS] OR "0894-8755"[IS] OR "1460-2415"[IS] OR "0022-3530"[IS] OR "1873-1570"[IS] OR "0300-9572"[IS] OR "0001-8686"[IS] OR "0001-8686"[IS] OR "1460-2121"[IS] OR "0266-903X"[IS] OR "1869-1870"[IS] OR "1674-7291"[IS] OR "2367-198X"[IS] OR "2367-198X"[IS] OR "0146-1672"[IS] OR "1552-7433"[IS] OR "1179-1349"[IS] OR "1179-1349"[IS] OR "1468-0432"[IS] OR "0968-6673"[IS] OR "0002-8312"[IS] OR "1935-1011"[IS] OR "1532-429X"[IS] OR "1097-6647"[IS] OR "1544-6131"[IS] OR "1544-6123"[IS] OR "1573-7209"[IS] OR "0969-6970"[IS] OR "1991-959X"[IS] OR "1991-9603"[IS] OR "2191-950X"[IS] OR "2191-9496"[IS] OR "1994-0416"[IS] OR "1994-0424"[IS] OR "0893-6080"[IS] OR "1879-2782"[IS] OR "2397-4648"[IS] OR "2397-4648"[IS] OR "1367-3270"[IS] OR "1367-3270"[IS] OR "0045-7825"[IS] OR "0045-7825"[IS] OR "2398-4910"[IS] OR "2398-4929"[IS] OR "0165-9936"[IS] OR "0165-9936"[IS] OR "2374-4677"[IS] OR "2374-4677"[IS] OR "2473-4284"[IS] OR "2473-4284"[IS] OR "1470-0328"[IS] OR "1471-0528"[IS] OR "1467-6419"[IS] OR "0950-0804"[IS] OR "1096-3634"[IS] OR "1084-9521"[IS] OR "2059-7029"[IS] OR "2059-7029"[IS] OR "1879-0364"[IS] OR "1369-5274"[IS] OR "1467-9922"[IS] OR "0023-8333"[IS] OR "1873-4235"[IS] OR "0956-5663"[IS] OR "0022-3506"[IS] OR "1467-6494"[IS] OR "0886-6236"[IS] OR "1944-9224"[IS] OR "2041-4889"[IS] OR "2041-4889"[IS] OR "0022-006X"[IS] OR "0022-006X"[IS] OR "1935-861X"[IS] OR "1876-4754"[IS] OR "0749-3797"[IS] OR "1873-2607"[IS] OR "1944-8252"[IS] OR "1944-8244"[IS] OR "1360-0443"[IS] OR "0965-2140"[IS] OR "1054-139X"[IS] OR "1879-1972"[IS] OR "2211-9124"[IS] OR "2211-9124"[IS] OR "2214-6296"[IS] OR "2214-6296"[IS] OR "0016-7606"[IS] OR "1943-2674"[IS] OR "2666-6065"[IS] OR "2666-6065"[IS] OR "2667-3789"[IS] OR "2667-3789"[IS] OR "0032-0889"[IS] OR "1532-2548"[IS] OR "0016-7037"[IS] OR "0046-564X"[IS] OR "0306-0225"[IS] OR "0306-0225"[IS] OR "0022-0027"[IS] OR "0022-0027"[IS] OR "1477-4054"[IS] OR "1467-5463"[IS] OR "1572-8145"[IS] OR "0956-5515"[IS] OR "0022-4405"[IS] OR "1873-3506"[IS] OR "2190-5991"[IS] OR "2190-6009"[IS] OR "0300-0729"[IS] OR "0300-0729"[IS] OR "1556-1607"[IS] OR "1556-1615"[IS] OR "1600-079X"[IS] OR "0742-3098"[IS] OR "1471-9037"[IS] OR

	"1471-9037"[IS] OR "0167-806X"[IS] OR "1573-0859"[IS] OR "1097-6744"[IS] OR "0002-8703"[IS] OR
	"1045-3172"[IS] OR "1467-8551"[IS] OR "2410-339X"[IS] OR "2410-3403"[IS] OR "0271-9142"[IS] OR
	"1573-2592"[IS] OR "0004-6280"[IS] OR "1538-3873"[IS] OR "2049-4408"[IS] OR "2049-4394"[IS] OR
	"0309-1317"[IS] OR "1468-2427"[IS] OR "1537-5927"[IS] OR "1537-5927"[IS] OR "2158-3188"[IS] OR
	"2158-3188"[IS] OR "0006-3207"[IS] OR "0006-3207"[IS] OR "0361-0128"[IS] OR "0361-0128"[IS] OR
	"2667-2375"[IS] OR "2667-2375"[IS] OR "2198-6436"[IS] OR "2198-6436"[IS] OR "2052-2630"[IS] OR
	"2052-2649"[IS] OR "2046-2441"[IS] OR "2046-2441"[IS] OR "2040-4603"[IS] OR "2040-4603"[IS] OR
	"1535-9476"[IS] OR "1535-9484"[IS] OR "0165-1781"[IS] OR "1872-7123"[IS] OR "2373-8057"[IS] OR
	"2373-8057"[IS] OR "1467-8624"[IS] OR "0009-3920"[IS] OR "1535-3958"[IS] OR "1535-3966"[IS] OR
	"2213-5979"[IS] OR "2213-5979"[IS] OR "0091-3022"[IS] OR "1095-6808"[IS] OR "1062-7995"[IS] OR
	"1099-159X"[IS] OR "1534-7605"[IS] OR "0037-7732"[IS] OR "0070-3370"[IS] OR "1533-7790"[IS] OR
	"2352-250X"[IS] OR "2352-250X"[IS] OR "1005-0302"[IS] OR "1005-0302"[IS] OR "1178-122X"[IS] OR
	"1178-122X"[IS] OR "0033-362X"[IS] OR "1537-5331"[IS] OR "2578-1863"[IS] OR "2578-1863"[IS] OR
	"1054-8408"[IS] OR "1540-7306"[IS] OR "0886-7798"[IS] OR "0886-7798"[IS] OR "2327-9125"[IS] OR
	"2327-9125"[IS] OR "0011-7315"[IS] OR "1540-5915"[IS] OR "1748-9326"[IS] OR "1748-9326"[IS] OR
	"1436-3291"[IS] OR "1436-3305"[IS] OR "1077-291X"[IS] OR "1077-291X"[IS] OR "1365-313X"[IS] OR
	"0960-7412"[IS] OR "0144-6665"[IS] OR "2044-8309"[IS] OR "1753-5131"[IS] OR "1753-5123"[IS] OR
	"0007-1013"[IS] OR "1467-8535"[IS] OR "2644-1330"[IS] OR "2644-1330"[IS] OR "2167-7034"[IS] OR
	"2167-7026"[IS] OR "1468-2354"[IS] OR "0020-6598"[IS] OR "0036-8326"[IS] OR "1098-237X"[IS] OR
	"2192-2640"[IS] OR "2192-2659"[IS] OR "2330-4022"[IS] OR "2330-4022"[IS] OR "1754-0747"[IS] OR
	"1754-0739"[IS] OR "1939-1374"[IS] OR "1939-1374"[IS] OR "0276-8739"[IS] OR "1520-6688"[IS] OR
	"0269-7491"[IS] OR "1873-6424"[IS] OR "2157-1422"[IS] OR "2157-1422"[IS] OR "1569-1802"[IS] OR
	"0945-053X"[IS] OR "1073-8584"[IS] OR "1089-4098"[IS] OR "1942-969X"[IS] OR "1942-9681"[IS] OR
	"0007-1323"[IS] OR "1365-2168"[IS] OR "0167-7659"[IS] OR "1573-7233"[IS] OR "2628-3735"[IS] OR
	"2628-3735"[IS] OR "2348-5779"[IS] OR "2347-6311"[IS] OR "0025-5831"[IS] OR "1432-1807"[IS] OR
	"1873-5371"[IS] OR "0306-4573"[IS] OR "1080-6377"[IS] OR "0002-9327"[IS] OR "1470-2738"[IS] OR
	"0143-005X"[IS] OR "1096-0821"[IS] OR "0749-596X"[IS] OR "0037-976X"[IS] OR "1540-5834"[IS] OR
	"0143-5671"[IS] OR "1475-5890"[IS] OR "2377-8253"[IS] OR "2377-8261"[IS] OR "0008-6223"[IS] OR
	"0008-6223"[IS] OR "1877-3435"[IS] OR "1877-3435"[IS] OR "1619-7089"[IS] OR "1619-7070"[IS] OR
	"0042-0980"[IS] OR "1360-063X"[IS] OR "0012-6667"[IS] OR "1179-1950"[IS] OR "2056-7944"[IS] OR
	"2056-7944"[IS] OR "1478-4092"[IS] OR "1478-4092"[IS] OR "1879-6265"[IS] OR "1879-6257"[IS] OR
	"2058-1742"[IS] OR "2058-5225"[IS] OR "0360-3016"[IS] OR "1879-355X"[IS] OR "0304-3835"[IS] OR
	"1872-7980"[IS] OR "1547-7215"[IS] OR "1069-031X"[IS] OR "0031-3203"[IS] OR "0031-3203"[IS] OR
	"1461-4448"[IS] OR "1461-7315"[IS] OR "0962-8436"[IS] OR "1471-2970"[IS] OR "1879-1646"[IS] OR
	"0167-6296"[IS] OR "1741-038X"[IS] OR "1741-038X"[IS] OR "0742-6046"[IS] OR "1520-6793"[IS] OR
	"0020-7225"[IS] OR "0020-7225"[IS] OR "1759-3948"[IS] OR "0265-0487"[IS] OR "0260-2938"[IS] OR
	"1469-297X"[IS] OR "2287-6391"[IS] OR "2287-6405"[IS] OR "2047-9980"[IS] OR "2047-9980"[IS] OR
	"1056-3911"[IS] OR "1534-7486"[IS] OR "0272-2631"[IS] OR "0272-2631"[IS] OR "0167-9236"[IS] OR
	"0167-9236"[IS] OR "1099-3460"[IS] OR "1468-2869"[IS] OR "1467-8276"[IS] OR "0002-9092"[IS] OR
	"0269-8463"[IS] OR "1365-2435"[IS] OR "1175-0561"[IS] OR "1175-0561"[IS] OR "1523-1739"[IS] OR
	"0888-8892"[IS] OR "0022-0477"[IS] OR "1365-2745"[IS] OR "1083-4435"[IS] OR "1083-4435"[IS] OR
	"2211-467X"[IS] OR "2211-467X"[IS] OR "0278-7407"[IS] OR "1944-9194"[IS] OR "1861-1125"[IS] OR
	"1861-1133"[IS] OR "1549-6325"[IS] OR "1549-6333"[IS] OR "0950-7051"[IS] OR "0950-7051"[IS] OR
	"0169-2046"[IS] OR "0169-2046"[IS] OR "1532-0979"[IS] OR "0147-5185"[IS] OR "1864-564X"[IS] OR
	"1864-5631"[IS] OR "2330-8249"[IS] OR "2330-8257"[IS] OR "1539-7718"[IS] OR "0033-5770"[IS] OR
	"1747-7603"[IS] OR "1368-3500"[IS] OR "2044-8325"[IS] OR "0963-1798"[IS] OR "1941-1413"[IS] OR
	"1941-1421"[IS] OR "1461-6696"[IS] OR "1469-8307"[IS] OR "1015-6305"[IS] OR "1750-3639"[IS] OR
	"1741-6248"[IS] OR "0894-4865"[IS] OR "0034-0553"[IS] OR "0034-0553"[IS] OR "1942-5546"[IS] OR
	"0025-6196"[IS] OR "2589-9147"[IS] OR "2589-9147"[IS] OR "1879-0429"[IS] OR "0958-1669"[IS] OR
	"0002-8614"[IS] OR "0002-8614"[IS] OR "1941-1367"[IS] OR "1941-1375"[IS] OR "1758-0854"[IS] OR
	"1758-0846"[IS] OR "2040-5804"[IS] OR "2040-5790"[IS] OR "2365-9440"[IS] OR "2365-9440"[IS] OR
	"1463-1326"[IS] OR "1462-8902"[IS] OR "1097-6779"[IS] OR "0016-5107"[IS] OR "0944-1174"[IS] OR
	"0944-1174"[IS] OR "2468-6069"[IS] OR "2468-6069"[IS] OR "0272-4944"[IS] OR "1522-9610"[IS] OR
	"1817-6127"[IS] OR "1817-6119"[IS] OR "0167-739X"[IS] OR "0167-739X"[IS] OR "1552-6542"[IS] OR
	"1056-4926"[IS] OR "1751-7656"[IS] OR "0016-8505"[IS] OR "2054-9369"[IS] OR "2095-7467"[IS] OR
	"2213-2961"[IS] OR "2095-2546"[IS] OR "1209-1367"[IS] OR "0840-6529"[IS] OR "2590-0498"[IS] OR
	"2590-0498"[IS] OR "1932-4529"[IS] OR "1932-4529"[IS] OR "0026-4598"[IS] OR "1432-1866"[IS] OR
	"0885-8969"[IS] OR "1558-0059"[IS] OR "0161-5505"[IS] OR "2159-662X"[IS] OR "1099-1719"[IS] OR
	"0968-0802"[IS] OR "1939-843X"[IS] OR "0021-843X"[IS] OR "1573-6601"[IS] OR "0047-2891"[IS] OR
	"0140-7791"[IS] OR "1365-3040"[IS] OR "2352-5509"[IS] OR "2352-5509"[IS] OR "0965-8564"[IS] OR

	"0965-8564"[IS] OR "0883-5403"[IS] OR "1532-8406"[IS] OR "1097-6809"[IS] OR "0741-5214"[IS] OR "0004-6256"[IS] OR "1538-3881"[IS] OR "1814-9332"[IS] OR "1814-9324"[IS] OR "1063-6692"[IS] OR "1063-6692"[IS] OR "2056-5933"[IS] OR "2056-5933"[IS] OR "1361-9209"[IS] OR "1361-9209"[IS] OR "0096-3445"[IS] OR "1939-2222"[IS] OR "0300-8428"[IS] OR "1435-1803"[IS] OR "1664-3224"[IS] OR "1664-3224"[IS] OR "1545-7214"[IS] OR "1064-7481"[IS] OR "1536-1225"[IS] OR "1536-1225"[IS] OR "1476-5381"[IS] OR "0007-1188"[IS] OR "1556-4967"[IS] OR "1556-4959"[IS] OR "1573-6660"[IS] OR "1040-7308"[IS] OR "1559-744X"[IS] OR "1559-7431"[IS] OR "1520-0469"[IS] OR "0022-4928"[IS] OR "0263-7758"[IS] OR "1472-3433"[IS] OR "2287-2728"[IS] OR "2287-285X"[IS] OR "1868-7083"[IS] OR "1868-7075"[IS] OR "2192-8614"[IS] OR "2192-8606"[IS] OR "0835-1813"[IS] OR "1532-7973"[IS] OR "2150-8097"[IS] OR "2150-8097"[IS] OR "1759-8486"[IS] OR "1759-8478"[IS] OR "0749-8063"[IS] OR "1526-3231"[IS] OR "1201-9712"[IS] OR "1878-3511"[IS] OR "1386-6532"[IS] OR "1873-5967"[IS] OR "2162-402X"[IS] OR "2162-4011"[IS] OR "1937-6448"[IS] OR "1937-6448"[IS] OR "0004-6361"[IS] OR "1432-0746"[IS] OR "2451-9022"[IS] OR "2451-9022"[IS] OR "1471-7727"[IS] OR "1471-7727"[IS] OR "1535-1386"[IS] OR "0021-9355"[IS] OR "1743-9507"[IS] OR "0264-2069"[IS] OR "1467-9531"[IS] OR "0081-1750"[IS] OR "1090-2082"[IS] OR "0001-8708"[IS] OR "0036-1410"[IS] OR "1095-7154"[IS] OR "0924-9338"[IS] OR "1778-3585"[IS] OR "1877-8585"[IS] OR "1877-8585"[IS] OR "1439-4456"[IS] OR "1438-8871"[IS] OR "2095-2686"[IS] OR "2095-2686"[IS] OR "1931-6690"[IS] OR "1936-0975"[IS] OR "1873-6785"[IS] OR "0360-5442"[IS] OR "2169-9100"[IS] OR "2169-9097"[IS] OR "0165-0327"[IS] OR "1573-2517"[IS] OR "0218-2025"[IS] OR "0218-2025"[IS] OR "1469-8722"[IS] OR "0950-0170"[IS] OR "1063-6536"[IS] OR "1558-0865"[IS] OR "2307-387X"[IS] OR "2307-387X"[IS] OR "0020-7640"[IS] OR "1741-2854"[IS] OR "0022-0396"[IS] OR "1090-2732"[IS] OR "1525-1314"[IS] OR "0263-4929"[IS] OR "1542-0124"[IS] OR "1542-0124"[IS] OR "0959-6526"[IS] OR "1879-1786"[IS] OR "0340-6717"[IS] OR "1432-1203"[IS] OR "1552-390X"[IS] OR "0013-9165"[IS] OR "1475-6773"[IS] OR "0017-9124"[IS] OR "2055-5008"[IS] OR "2055-5008"[IS] OR "1873-5347"[IS] OR "0277-9536"[IS] OR "0883-4237"[IS] OR "0883-4237"[IS] OR "1934-8266"[IS] OR "1934-8258"[IS] OR "0169-2070"[IS] OR "0169-2070"[IS] OR "0346-251X"[IS] OR "0346-251X"[IS] OR "1050-5164"[IS] OR "1050-5164"[IS] OR "0190-292X"[IS] OR "1541-0072"[IS] OR "1545-5955"[IS] OR "1545-5955"[IS] OR "2214-7535"[IS] OR "2214-7535"[IS] OR "2057-3995"[IS] OR "2057-3995"[IS] OR "2524-7867"[IS] OR "2524-7867"[IS] OR "1528-1167"[IS] OR "0013-9580"[IS] OR "1359-432X"[IS] OR "1359-432X"[IS] OR "1365-1609"[IS] OR "1365-1609"[IS] OR "0021-8901"[IS] OR "1365-2664"[IS] OR "0014-2921"[IS] OR "0014-2921"[IS] OR "1467-7687"[IS] OR "1363-755X"[IS] OR "2042-6410"[IS] OR "2042-6410"[IS] OR "1938-3207"[IS] OR "0002-9165"[IS] OR "1531-5487"[IS] OR "1044-3983"[IS] OR "1759-4812"[IS] OR "1759-4820"[IS] OR "1350-5084"[IS] OR "1461-7323"[IS] OR "1463-9262"[IS] OR "1463-9270"[IS] OR "1096-0783"[IS] OR "0022-1236"[IS] OR "1097-6795"[IS] OR "0894-7317"[IS] OR "0012-9658"[IS] OR "0012-9658"[IS] OR "0071-1365"[IS] OR "1744-1358"[IS] OR "2050-3911"[IS] OR "2050-3911"[IS] OR "1096-9071"[IS] OR "0146-6615"[IS] OR "1879-2057"[IS] OR "0001-4575"[IS] OR "2212-0416"[IS] OR "2212-0416"[IS] OR "1758-5341"[IS] OR "0016-9013"[IS] OR "1083-6101"[IS] OR "1083-6101"[IS] OR "2589-7918"[IS] OR "2589-7918"[IS] OR "1468-2702"[IS] OR "1468-2710"[IS] OR "0018-1560"[IS] OR "1573-174X"[IS] OR "1747-4930"[IS] OR "1747-4949"[IS] OR "2470-2986"[IS] OR "2470-2986"[IS] OR "2056-3051"[IS] OR "2056-3051"[IS] OR "2378-0967"[IS] OR "2378-0967"[IS] OR "0013-1644"[IS] OR "1552-3888"[IS] OR "2666-5425"[IS] OR "2666-5425"[IS] OR "1052-150X"[IS] OR "1052-150X"[IS] OR "2162-2531"[IS] OR "2162-2531"[IS] OR "0048-9697"[IS] OR "1879-1026"[IS] OR "0022-5347"[IS] OR "1527-3792"[IS] OR "2212-0947"[IS] OR "2212-0947"[IS] OR "1537-260X"[IS] OR "1537-260X"[IS] OR "1880-9693"[IS] OR "1880-8190"[IS] OR "1365-294X"[IS] OR "0962-1083"[IS] OR "2212-571X"[IS] OR "2212-571X"[IS] OR "1539-7262"[IS] OR "0022-2275"[IS] OR "1872-6259"[IS] OR "0016-7061"[IS] OR "1570-8659"[IS] OR "1570-8659"[IS] OR "1533-8533"[IS] OR "0037-7791"[IS] OR "1873-0442"[IS] OR "1477-8939"[IS] OR "1674-9871"[IS] OR "1674-9871"[IS] OR "1354-0688"[IS] OR "1460-3683"[IS] OR "1478-3223"[IS] OR "1478-3231"[IS] OR "1612-5118"[IS] OR "1612-510X"[IS] OR "2667-2952"[IS] OR "2667-2952"[IS] OR "0004-637X"[IS] OR "1538-4357"[IS] OR "0271-678X"[IS] OR "1559-7016"[IS] OR "2365-709X"[IS] OR "2365-709X"[IS] OR "1055-9965"[IS] OR "1538-7755"[IS] OR "1466-4429"[IS] OR "1350-1763"[IS] OR "2666-2477"[IS] OR "2666-2477"[IS] OR "1937-4208"[IS] OR "0885-8977"[IS] OR "2211-9736"[IS] OR "2211-9736"[IS] OR "2062-5871"[IS] OR "2063-5303"[IS] OR "2096-6482"[IS] OR "2589-9651"[IS] OR "0021-8790"[IS] OR "1365-2656"[IS] OR "2196-5420"[IS] OR "2196-5625"[IS] OR "0024-6115"[IS] OR "1460-244X"[IS] OR "2662-4443"[IS] OR "2662-4443"[IS] OR "1741-3052"[IS] OR "1473-0952"[IS] OR "1989-4007"[IS] OR "1889-1861"[IS] OR "0376-7388"[IS] OR "0376-7388"[IS] OR "2468-0257"[IS] OR "2096-2797"[IS] OR "2575-8314"[IS] OR "2575-8314"[IS] OR "2050-0068"[IS] OR "2050-0068"[IS] OR "2162-3619"[IS] OR "2162-3619"[IS] OR "1573-7756"[IS] OR "1059-0161"[IS] OR "1949-3045"[IS] OR "1949-3045"[IS] OR "1759-4685"[IS] OR "1674-2788"[IS] OR "2772-4204"[IS] OR "2772-4204"[IS] OR "0921-8009"[IS] OR "0921-8009"[IS] OR "1099-5129"[IS] OR "1532-2092"[IS] OR "0036-1429"[IS] OR "1095-7170"[IS] OR "2096-7438"[IS] OR "2666-6839"[IS] OR "0962-8452"[IS] OR "0962-8452"[IS] OR "2542-4653"[IS] OR

	"2542-4653"[IS] OR "0306-9192"[IS] OR "0306-9192"[IS] OR "2374-6149"[IS] OR "2374-6149"[IS] OR "0002-9394"[IS] OR "1879-1891"[IS] OR "2199-3971"[IS] OR "2199-398X"[IS] OR "1095-953X"[IS] OR "0969-9961"[IS] OR "1469-5928"[IS] OR "0261-9768"[IS] OR "1557-9670"[IS] OR "0018-9480"[IS] OR "2199-160X"[IS] OR "2199-160X"[IS] OR "2666-5468"[IS] OR "2666-5468"[IS] OR "2196-5404"[IS] OR "2196-5404"[IS] OR "1045-9219"[IS] OR "1045-9219"[IS] OR "1535-7163"[IS] OR "1538-8514"[IS] OR "1520-0493"[IS] OR "0027-0644"[IS] OR "0950-0618"[IS] OR "0950-0618"[IS] OR "2059-7037"[IS] OR "2059-7037"[IS] OR "0266-4658"[IS] OR "1468-0327"[IS] OR "0013-161X"[IS] OR "1552-3519"[IS] OR "1552-8545"[IS] OR "1075-5470"[IS] OR "2328-9503"[IS] OR "2328-9503"[IS] OR "2575-1077"[IS] OR "2575-1077"[IS] OR "1568-4946"[IS] OR "1568-4946"[IS] OR "1098-2736"[IS] OR "0022-4308"[IS] OR "1057-5219"[IS] OR "1057-5219"[IS] OR "0360-3989"[IS] OR "1468-2958"[IS] OR "1743-9892"[IS] OR "1743-9884"[IS] OR "1475-0198"[IS] OR "0158-7919"[IS] OR "0736-5853"[IS] OR "0736-5853"[IS] OR "1759-8451"[IS] OR "1660-5373"[IS] OR "1468-201X"[IS] OR "1355-6037"[IS] OR "1552-7417"[IS] OR "1086-0266"[IS] OR "1475-9217"[IS] OR "1475-9217"[IS] OR "0163-8343"[IS] OR "1873-7714"[IS] OR "0022-5223"[IS] OR "1097-685X"[IS] OR "2379-3694"[IS] OR "2379-3694"[IS] OR "0957-4174"[IS] OR "0957-4174"[IS] OR "1367-0050"[IS] OR "1367-0050"[IS] OR "1541-1672"[IS] OR "1541-1672"[IS] OR "0022-0345"[IS] OR "0022-0345"[IS] OR "1553-7358"[IS] OR "1553-734X"[IS] OR "1745-2767"[IS] OR "1745-2759"[IS] OR "0018-9448"[IS] OR "1557-9654"[IS] OR "0010-440X"[IS] OR "1532-8384"[IS] OR "1096-0902"[IS] OR "0095-8956"[IS] OR "0301-4207"[IS] OR "0301-4207"[IS] OR "1478-811X"[IS] OR "1478-811X"[IS] OR "1467-2960"[IS] OR "1467-2979"[IS] OR "2154-1272"[IS] OR "2154-1264"[IS] OR "0891-2432"[IS] OR "1552-3977"[IS] OR "1046-8188"[IS] OR "1558-2868"[IS] OR "0266-1144"[IS] OR "0266-1144"[IS] OR "1520-7552"[IS] OR "1520-7560"[IS] OR "1063-4584"[IS] OR "1522-9653"[IS] OR "1549-7852"[IS] OR "1040-8398"[IS] OR "1359-2998"[IS] OR "1468-2052"[IS] OR "1943-5606"[IS] OR "1090-0241"[IS] OR "1651-2316"[IS] OR "1650-6073"[IS] OR "1549-490X"[IS] OR "1083-7159"[IS] OR "1931-5244"[IS] OR "1931-5244"[IS] OR "1548-7733"[IS] OR "1548-7733"[IS] OR "2451-9103"[IS] OR "2451-9111"[IS] OR "0378-7753"[IS] OR "0378-7753"[IS] OR "2472-1751"[IS] OR "2472-1751"[IS] OR "0966-6923"[IS] OR "0966-6923"[IS] OR "1879-310X"[IS] OR "0967-070X"[IS] OR "1040-8363"[IS] OR "1549-781X"[IS] OR "0964-4016"[IS] OR "0964-4016"[IS] OR "1948-7185"[IS] OR "1948-7185"[IS] OR "1262-3636"[IS] OR "1262-3636"[IS] OR "0723-2632"[IS] OR "0723-2632"[IS] OR "1943-2631"[IS] OR "0016-6731"[IS] OR "1873-1457"[IS] OR "1571-0645"[IS] OR "2352-8729"[IS] OR "2352-8729"[IS] OR "1432-0894"[IS] OR "0930-7575"[IS] OR "2214-2096"[IS] OR "2214-2096"[IS] OR "2399-3650"[IS] OR "2399-3650"[IS] OR "1873-4995"[IS] OR "0168-3659"[IS] OR "1088-1980"[IS] OR "1530-9290"[IS] OR "1477-9137"[IS] OR "0021-9533"[IS] OR "1573-5214"[IS] OR "1573-5214"[IS] OR "0010-938X"[IS] OR "0010-938X"[IS] OR "0094-8276"[IS] OR "0094-8276"[IS] OR "0960-1627"[IS] OR "1467-9965"[IS] OR "1096-0260"[IS] OR "0091-7435"[IS] OR "0010-7999"[IS] OR "1432-0967"[IS] OR "2168-619X"[IS] OR "2168-6181"[IS] OR "1573-2606"[IS] OR "1389-9155"[IS] OR "1525-2027"[IS] OR "1525-2027"[IS] OR "1078-0998"[IS] OR "1536-4844"[IS] OR "1879-0887"[IS] OR "0167-8140"[IS] OR "1873-2585"[IS] OR "1047-2797"[IS] OR "0013-0095"[IS] OR "0013-0095"[IS] OR "1758-2652"[IS] OR "1758-2652"[IS] OR "1460-2083"[IS] OR "0964-6906"[IS] OR "0162-3737"[IS] OR "1935-1062"[IS] OR "1089-2680"[IS] OR "1939-1552"[IS] OR "2324-6200"[IS] OR "2324-6200"[IS] OR "1545-5971"[IS] OR "1545-5971"[IS] OR "1930-7381"[IS] OR "1930-7381"[IS] OR "2329-0501"[IS] OR "2329-0501"[IS] OR "1066-8888"[IS] OR "0949-877X"[IS] OR "2643-1564"[IS] OR "2643-1564"[IS] OR "1460-2431"[IS] OR "0022-0957"[IS] OR "1438-8812"[IS] OR "0013-726X"[IS] OR "0004-8674"[IS] OR "1440-1614"[IS] OR "1473-6578"[IS] OR "0951-7367"[IS] OR "0004-3702"[IS] OR "0004-3702"[IS] OR "1532-480X"[IS] OR "1088-8691"[IS] OR "0246-0203"[IS] OR "0246-0203"[IS] OR "2213-2198"[IS] OR "2213-2201"[IS] OR "0022-2593"[IS] OR "1468-6244"[IS] OR "0960-1481"[IS] OR "0960-1481"[IS] OR "1525-1497"[IS] OR "0884-8734"[IS] OR "2666-6367"[IS] OR "2666-6375"[IS] OR "1050-1738"[IS] OR "1873-2615"[IS] OR "1441-3582"[IS] OR "1839-3349"[IS] OR "1537-4424"[IS] OR "1537-4416"[IS] OR "2637-4943"[IS] OR "2637-4943"[IS] OR "0002-9262"[IS] OR "1476-6256"[IS] OR "1096-7176"[IS] OR "1096-7184"[IS] OR "1369-118X"[IS] OR "1369-118X"[IS] OR "2515-7655"[IS] OR "2515-7655"[IS] OR "1044-1549"[IS] OR "1535-4989"[IS] OR "2662-6810"[IS] OR "2052-7276"[IS] OR "0021-9258"[IS] OR "1083-351X"[IS] OR "0022-2623"[IS] OR "1520-4804"[IS] OR "1743-8977"[IS] OR "1743-8977"[IS] OR "1874-9399"[IS] OR "1876-4320"[IS] OR "2352-8737"[IS] OR "2352-8737"[IS] OR "2352-8478"[IS] OR "2352-8486"[IS] OR "2199-4501"[IS] OR "2095-8226"[IS] OR "2352-1546"[IS] OR "2352-1546"[IS] OR "1018-8827"[IS] OR "1435-165X"[IS] OR "2169-897X"[IS] OR "2169-8996"[IS] OR "2055-7124"[IS] OR "2055-7124"[IS] OR "2352-8648"[IS] OR "2468-5925"[IS] OR "0167-1987"[IS] OR "0167-1987"[IS] OR "2044-5423"[IS] OR "2044-5415"[IS] OR "1090-5138"[IS] OR "1090-5138"[IS] OR "1089-7798"[IS] OR "1089-7798"[IS] OR "0960-0035"[IS] OR "0960-0035"[IS] OR "1098-5514"[IS] OR "0022-538X"[IS] OR "1525-8610"[IS] OR "1538-9375"[IS] OR "1878-9293"[IS] OR "1878-9307"[IS] OR "1938-274X"[IS] OR "1065-9129"[IS] OR "2196-5412"[IS] OR "2196-5412"[IS] OR "0289-2316"[IS] OR "0289-2316"[IS] OR "1520-5703"[IS] OR "0161-4940"[IS] OR "0942-2056"[IS] OR "1433-7347"[IS] OR "0005-7967"[IS] OR "1873-622X"[IS] OR "0266-352X"[IS] OR

	"1873-7633"[IS] OR "1758-0463"[IS] OR "1758-0463"[IS] OR "1351-0347"[IS] OR "1743-890X"[IS] OR "1432-0479"[IS] OR "0938-2259"[IS] OR "0305-1846"[IS] OR "1365-2990"[IS] OR "2352-8273"[IS] OR "2352-8273"[IS] OR "1059-6011"[IS] OR "1059-6011"[IS] OR "1043-6618"[IS] OR "1096-1186"[IS] OR "1022-1824"[IS] OR "1022-1824"[IS] OR "2352-6734"[IS] OR "2352-6734"[IS] OR "1069-9384"[IS] OR "1531-5320"[IS] OR "2666-1233"[IS] OR "2666-1233"[IS] OR "2107-6952"[IS] OR "0301-0422"[IS] OR "2214-1588"[IS] OR "2214-1588"[IS] OR "1471-2288"[IS] OR "1471-2288"[IS] OR "1574-7891"[IS] OR "1878-0261"[IS] OR "1027-5606"[IS] OR "1607-7938"[IS] OR "0093-9994"[IS] OR "1939-9367"[IS] OR "1755-7739"[IS] OR "1755-7747"[IS] OR "1090-2694"[IS] OR "0021-9517"[IS] OR "0021-972X"[IS] OR "1945-7197"[IS] OR "1557-9611"[IS] OR "0018-9316"[IS] OR "1464-5114"[IS] OR "0898-5626"[IS] OR "1873-491X"[IS] OR "0020-7489"[IS] OR "1839-5260"[IS] OR "1447-6770"[IS] OR "2475-1421"[IS] OR "2475-1421"[IS] OR "1654-7209"[IS] OR "0044-7447"[IS] OR "1075-4253"[IS] OR "1075-4253"[IS] OR "2000-8066"[IS] OR "2000-8066"[IS] OR "1468-2478"[IS] OR "0020-8833"[IS] OR "2522-0691"[IS] OR "2522-0705"[IS] OR "1461-7005"[IS] OR "1362-3613"[IS] OR "1674-733X"[IS] OR "1869-1919"[IS] OR "0266-3538"[IS] OR "0266-3538"[IS] OR "0014-2980"[IS] OR "1521-4141"[IS] OR "1568-7864"[IS] OR "1568-7864"[IS] OR "1018-4813"[IS] OR "1476-5438"[IS] OR "1876-4517"[IS] OR "1876-4525"[IS] OR "2471-285X"[IS] OR "2471-285X"[IS] OR "1674-7755"[IS] OR "1674-7755"[IS] OR "1573-7152"[IS] OR "1569-5239"[IS] OR "0360-8352"[IS] OR "0360-8352"[IS] OR "1879-0836"[IS] OR "0951-8320"[IS] OR "1497-0015"[IS] OR "0706-7437"[IS] OR "0266-7215"[IS] OR "1468-2672"[IS] OR "1939-134X"[IS] OR "1040-3590"[IS] OR "1360-0591"[IS] OR "0034-3404"[IS] OR "1359-6462"[IS] OR "1359-6462"[IS] OR "2193-7680"[IS] OR "2193-7680"[IS] OR "1545-2263"[IS] OR "1545-2255"[IS] OR "1744-3210"[IS] OR "0958-8221"[IS] OR "1470-6423"[IS] OR "1470-6431"[IS] OR "1090-2716"[IS] OR "0021-9991"[IS] OR "0890-765X"[IS] OR "1748-0361"[IS] OR "1465-993X"[IS] OR "1465-9921"[IS] OR "2045-3701"[IS] OR "2045-3701"[IS] OR "2095-8099"[IS] OR "2095-8099"[IS] OR "1449-2288"[IS] OR "1449-2288"[IS] OR "1433-9285"[IS] OR "0933-7954"[IS] OR "1942-0870"[IS] OR "1942-0862"[IS] OR "1569-1993"[IS] OR "1873-5010"[IS] OR "1552-3357"[IS] OR "0275-0740"[IS] OR "0956-053X"[IS] OR "1879-2456"[IS] OR "2168-0485"[IS] OR "2168-0485"[IS] OR "0305-9049"[IS] OR "1468-0084"[IS] OR "2169-9356"[IS] OR "2169-9313"[IS] OR "1536-9323"[IS] OR "1536-9323"[IS] OR "0264-1275"[IS] OR "1873-4197"[IS] OR "2666-9552"[IS] OR "2666-9552"[IS] OR "1552-6712"[IS] OR "1552-6712"[IS] OR "0002-0729"[IS] OR "1468-2834"[IS] OR "1047-3211"[IS] OR "1460-2199"[IS] OR "1934-3639"[IS] OR "1934-3647"[IS] OR "1745-9133"[IS] OR "1538-6473"[IS] OR "1558-6898"[IS] OR "1558-6901"[IS] OR "1098-8971"[IS] OR "0272-8087"[IS] OR "0046-3892"[IS] OR "0046-3892"[IS] OR "2059-8696"[IS] OR "2059-8688"[IS] OR "1530-0315"[IS] OR "0195-9131"[IS] OR "1365-2966"[IS] OR "0035-8711"[IS] OR "1520-6882"[IS] OR "0003-2700"[IS] OR "1468-2079"[IS] OR "0007-1161"[IS] OR "0938-7994"[IS] OR "1432-1084"[IS] OR "1532-690X"[IS] OR "0737-0008"[IS] OR "1939-0599"[IS] OR "0012-1649"[IS] OR "1366-9516"[IS] OR "1472-4642"[IS] OR "0952-1976"[IS] OR "0952-1976"[IS] OR "0010-7824"[IS] OR "0010-7824"[IS] OR "1756-2864"[IS] OR "1756-2856"[IS] OR "0951-3574"[IS] OR "0951-3574"[IS] OR "0045-6535"[IS] OR "0045-6535"[IS] OR "1879-1344"[IS] OR "0144-8617"[IS] OR "1548-3568"[IS] OR "1548-3576"[IS] OR "1939-5582"[IS] OR "1051-0761"[IS] OR "2471-254X"[IS] OR "2471-254X"[IS] OR "0145-2134"[IS] OR "0145-2134"[IS] OR "1545-0821"[IS] OR "0895-9420"[IS] OR "2572-4517"[IS] OR "2572-4525"[IS] OR "1462-0324"[IS] OR "1462-0332"[IS] OR "2569-2925"[IS] OR "2569-2925"[IS] OR "1061-8600"[IS] OR "1537-2715"[IS] OR "0885-4122"[IS] OR "1552-6593"[IS] OR "0953-7287"[IS] OR "1366-5871"[IS] OR "2151-464X"[IS] OR "2151-4658"[IS] OR "0264-2751"[IS] OR "0264-2751"[IS] OR "0820-3946"[IS] OR "0008-2329"[IS] OR "0305-0548"[IS] OR "1873-765X"[IS] OR "0378-4266"[IS] OR "0378-4266"[IS] OR "0307-5079"[IS] OR "1470-174X"[IS] OR "1944-7973"[IS] OR "0043-1397"[IS] OR "1958-5969"[IS] OR "1294-8322"[IS] OR "1096-9845"[IS] OR "0098-8847"[IS] OR "2096-1529"[IS] OR "2096-1529"[IS] OR "0098-5589"[IS] OR "0098-5589"[IS] OR "1471-5007"[IS] OR "1471-4922"[IS] OR "1749-7922"[IS] OR "1749-7922"[IS] OR "1742-464X"[IS] OR "1432-1033"[IS] OR "1460-2350"[IS] OR "0268-1161"[IS] OR "1750-984X"[IS] OR "1750-9858"[IS] OR "0142-6001"[IS] OR "1477-450X"[IS] OR "1939-005X"[IS] OR "1939-5094"[IS] OR "1557-4644"[IS] OR "0362-5915"[IS] OR "1474-0346"[IS] OR "1474-0346"[IS] OR "1527-4764"[IS] OR "1527-4764"[IS] OR "0529-567X"[IS] OR "0529-567X"[IS] OR "1528-1175"[IS] OR "0003-3022"[IS] OR "1523-0864"[IS] OR "1557-7716"[IS] OR "2001-1326"[IS] OR "2001-1326"[IS] OR "1523-4681"[IS] OR "0884-0431"[IS] OR "1674-7313"[IS] OR "1869-1897"[IS] OR "1384-6647"[IS] OR "1384-6647"[IS] OR "0892-9912"[IS] OR "0892-9912"[IS] OR "0370-2693"[IS] OR "0370-2693"[IS] OR "1079-5006"[IS] OR "1079-5006"[IS] OR "0166-3542"[IS] OR "1872-9096"[IS] OR "1473-6551"[IS] OR "1350-7540"[IS] OR "1743-9760"[IS] OR "1743-9779"[IS] OR "0277-3791"[IS] OR "0277-3791"[IS] OR "1399-5618"[IS] OR "1398-5647"[IS] OR "2666-920X"[IS] OR "2666-920X"[IS] OR "1551-3076"[IS] OR "1551-3084"[IS] OR "2054-4650"[IS] OR "2054-4650"[IS] OR "1538-6341"[IS] OR "1538-6341"[IS] OR "0301-9268"[IS] OR "0301-9268"[IS] OR "2589-9333"[IS] OR "2589-9333"[IS] OR "2073-4468"[IS] OR "2073-4468"[IS] OR "0251-5350"[IS] OR "1423-0208"[IS] OR "2095-6339"[IS] OR "2095-6339"[IS] OR "1745-3925"[IS] OR "1745-3933"[IS] OR "2377-3766"[IS] OR "2377-3766"[IS] OR "1932-4537"[IS] OR

	"1932-4537"[IS] OR "1470-5931"[IS] OR "1741-301X"[IS] OR "2049-9957"[IS] OR "2049-9957"[IS] OR "1600-0501"[IS] OR "0905-7161"[IS] OR "1873-7838"[IS] OR "0010-0277"[IS] OR "0022-5096"[IS] OR "0022-5096"[IS] OR "2379-5077"[IS] OR "2379-5077"[IS] OR "0308-5147"[IS] OR "1469-5766"[IS] OR "1065-9471"[IS] OR "1097-0193"[IS] OR "2270-518X"[IS] OR "2429-7100"[IS] OR "0272-3638"[IS] OR "1938-2847"[IS] OR "1869-8182"[IS] OR "1869-814X"[IS] OR "2475-1456"[IS] OR "2475-1456"[IS] OR "1469-7874"[IS] OR "1741-2625"[IS] OR "2152-5250"[IS] OR "2152-5250"[IS] OR "0960-7722"[IS] OR "1365-2184"[IS] OR "2253-1556"[IS] OR "2253-1556"[IS] OR "1726-2135"[IS] OR "1684-8799"[IS] OR "1573-0956"[IS] OR "0169-3298"[IS] OR "1526-3800"[IS] OR "1526-3800"[IS] OR "2473-2400"[IS] OR "2473-2400"[IS] OR "1573-0565"[IS] OR "0885-6125"[IS] OR "1873-6416"[IS] OR "1462-9011"[IS] OR "1095-8630"[IS] OR "0301-4797"[IS] OR "2574-1292"[IS] OR "2574-1292"[IS] OR "1671-4083"[IS] OR "1745-7254"[IS] OR "0079-6816"[IS] OR "0079-6816"[IS] OR "2169-0375"[IS] OR "1536-1365"[IS] OR "1365-7852"[IS] OR "1476-5608"[IS] OR "0090-8258"[IS] OR "1095-6859"[IS] OR "2644-1314"[IS] OR "2644-1314"[IS] OR "1541-4140"[IS] OR "0735-6331"[IS] OR "1573-7853"[IS] OR "0304-2421"[IS] OR "1071-1007"[IS] OR "1071-1007"[IS] OR "2168-2208"[IS] OR "2168-2194"[IS] OR "0305-1838"[IS] OR "1365-2907"[IS] OR "1573-059X"[IS] OR "0923-0645"[IS] OR "1931-1516"[IS] OR "1528-3542"[IS] OR "0022-1694"[IS] OR "0022-1694"[IS] OR "0268-1072"[IS] OR "1468-005X"[IS] OR "2196-7822"[IS] OR "2196-7822"[IS] OR "1541-7786"[IS] OR "1557-3125"[IS] OR "0921-5093"[IS] OR "0921-5093"[IS] OR "1532-6500"[IS] OR "1058-2746"[IS] OR "0276-5624"[IS] OR "0276-5624"[IS] OR "1549-9626"[IS] OR "1549-9618"[IS] OR "1609-4069"[IS] OR "1609-4069"[IS] OR "0267-257X"[IS] OR "1472-1376"[IS] OR "2050-6406"[IS] OR "2050-6414"[IS] OR "1432-0851"[IS] OR "0340-7004"[IS] OR "1432-1394"[IS] OR "0949-7714"[IS] OR "2166-532X"[IS] OR "2166-532X"[IS] OR "1552-8286"[IS] OR "0894-4393"[IS] OR "0007-8506"[IS] OR "1726-0604"[IS] OR "1359-835X"[IS] OR "1359-835X"[IS] OR "1077-5552"[IS] OR "1077-5552"[IS] OR "1532-1983"[IS] OR "0261-5614"[IS] OR "2045-8118"[IS] OR "2045-8118"[IS] OR "0264-8377"[IS] OR "0264-8377"[IS] OR "2161-1653"[IS] OR "2161-1653"[IS] OR "1558-7991"[IS] OR "0278-0380"[IS] OR "0039-8322"[IS] OR "0039-8322"[IS] OR "2076-393X"[IS] OR "2076-393X"[IS] OR "2469-4460"[IS] OR "2469-4452"[IS] OR "0024-4937"[IS] OR "0024-4937"[IS] OR "1615-3383"[IS] OR "1615-3375"[IS] OR "1069-4404"[IS] OR "1069-4404"[IS] OR "1749-8198"[IS] OR "1749-8198"[IS] OR "1552-6127"[IS] OR "1090-1981"[IS] OR "1066-2243"[IS] OR "1066-2243"[IS] OR "2516-4236"[IS] OR "2516-4236"[IS] OR "0378-3839"[IS] OR "0378-3839"[IS] OR "1007-0214"[IS] OR "1007-0214"[IS] OR "2327-4697"[IS] OR "2327-4697"[IS] OR "2040-2392"[IS] OR "2040-2392"[IS] OR "1173-8804"[IS] OR "1179-190X"[IS] OR "1751-9004"[IS] OR "1751-9004"[IS] OR "1464-3618"[IS] OR "0165-1587"[IS] OR "1479-8409"[IS] OR "1479-8409"[IS] OR "1943-5479"[IS] OR "0742-597X"[IS] OR "0166-5162"[IS] OR "0166-5162"[IS] OR "1552-4558"[IS] OR "0095-7984"[IS] OR "1229-2370"[IS] OR "1229-2370"[IS] OR "2325-8306"[IS] OR "2325-8292"[IS] OR "1689-1392"[IS] OR "1425-8153"[IS] OR "1532-6535"[IS] OR "0009-9236"[IS] OR "2666-2027"[IS] OR "2666-2027"[IS] OR "2055-6837"[IS] OR "2055-6845"[IS] OR "0163-4437"[IS] OR "1460-3675"[IS] OR "2531-0429"[IS] OR "2531-0437"[IS] OR "0167-8809"[IS] OR "0167-8809"[IS] OR "1354-0661"[IS] OR "1354-0661"[IS] OR "2470-0010"[IS] OR "2470-0029"[IS] OR "1742-7061"[IS] OR "1742-7061"[IS] OR "1557-7449"[IS] OR "1087-2914"[IS] OR "0266-4666"[IS] OR "1469-4360"[IS] OR "0016-8033"[IS] OR "1942-2156"[IS] OR "1520-9210"[IS] OR "1520-9210"[IS] OR "1549-7801"[IS] OR "0738-8551"[IS] OR "1465-7309"[IS] OR "1067-3229"[IS] OR "0167-6245"[IS] OR "0167-6245"[IS] OR "2352-2895"[IS] OR "2352-2895"[IS] OR "1573-0816"[IS] OR "0013-1954"[IS] OR "1550-6606"[IS] OR "0022-1767"[IS] OR "1384-5810"[IS] OR "1573-756X"[IS] OR "1936-6574"[IS] OR "1876-7583"[IS] OR "1096-0953"[IS] OR "0013-9351"[IS] OR "2194-041X"[IS] OR "2194-0401"[IS] OR "0168-8227"[IS] OR "1872-8227"[IS] OR "1540-6229"[IS] OR "1080-8620"[IS] OR "2044-8295"[IS] OR "0007-1269"[IS] OR "0079-6786"[IS] OR "0079-6786"[IS] OR "2053-1583"[IS] OR "2053-1583"[IS] OR "1461-7188"[IS] OR "1368-4302"[IS] OR "0885-2006"[IS] OR "0885-2006"[IS] OR "0309-0566"[IS] OR "0309-0566"[IS] OR "1758-2946"[IS] OR "1758-2946"[IS] OR "1365-2729"[IS] OR "0266-4909"[IS] OR "1572-3089"[IS] OR "1572-3089"[IS] OR "1365-2923"[IS] OR "0308-0110"[IS] OR "2590-1982"[IS] OR "2590-1982"[IS] OR "1365-2141"[IS] OR "0007-1048"[IS] OR "1872-826X"[IS] OR "1569-8432"[IS] OR "2638-6135"[IS] OR "2638-6135"[IS] OR "2352-9407"[IS] OR "2352-9407"[IS] OR "1547-6561"[IS] OR "1040-8436"[IS] OR "1099-1530"[IS] OR "1045-6740"[IS] OR "1749-6632"[IS] OR "0077-8923"[IS] OR "0197-9183"[IS] OR "0197-9183"[IS] OR "1389-1286"[IS] OR "1389-1286"[IS] OR "0263-2373"[IS] OR "0263-2373"[IS] OR "0891-5849"[IS] OR "1873-4596"[IS] OR "1774-024X"[IS] OR "1969-6213"[IS] OR "1873-7072"[IS] OR "0308-8146"[IS] OR "2589-0042"[IS] OR "2589-0042"[IS] OR "0149-1423"[IS] OR "0149-1423"[IS] OR "0168-1923"[IS] OR "0168-1923"[IS] OR "1474-7480"[IS] OR "1475-3030"[IS] OR "2056-4724"[IS] OR "2056-4724"[IS] OR "0741-5400"[IS] OR "1938-3673"[IS] OR "2468-6530"[IS] OR "2468-6530"[IS] OR "1744-9324"[IS] OR "0008-4239"[IS] OR "1097-6787"[IS] OR "0190-9622"[IS] OR "2399-3669"[IS] OR "2399-3669"[IS] OR "1673-8527"[IS] OR "1673-8527"[IS] OR "2040-3372"[IS] OR "2040-3364"[IS] OR "2198-6592"[IS] OR "2198-6592"[IS] OR "1532-799X"[IS] OR "1088-8438"[IS] OR "2056-9548"[IS] OR "2056-9556"[IS] OR "1751-570X"[IS] OR "1751-570X"[IS] OR "1932-4545"[IS] OR

"1932-4545"[IS] OR "2210-6359"[IS] OR "2210-6340"[IS] OR "2426-0266"[IS] OR "2426-0266"[IS] OR
"0742-051X"[IS] OR "0742-051X"[IS] OR "0022-362X"[IS] OR "1939-8549"[IS] OR "1538-3008"[IS] OR
"0091-6331"[IS] OR "2666-4453"[IS] OR "2666-4453"[IS] OR "1745-3682"[IS] OR "1745-3674"[IS] OR
"1537-5285"[IS] OR "0022-2186"[IS] OR "2641-3337"[IS] OR "2641-3337"[IS] OR "0895-0695"[IS] OR
"0895-0695"[IS] OR "2590-1834"[IS] OR "2590-1834"[IS] OR "2572-2611"[IS] OR "2572-2611"[IS] OR
"2157-9024"[IS] OR "2157-9024"[IS] OR "2334-265X"[IS] OR "2334-265X"[IS] OR "0265-5322"[IS] OR
"1477-0946"[IS] OR "0169-5002"[IS] OR "1872-8332"[IS] OR "1557-9077"[IS] OR "1050-7256"[IS] OR
"0378-7788"[IS] OR "0378-7788"[IS] OR "1756-2589"[IS] OR "1756-2570"[IS] OR "2046-3758"[IS] OR
"2046-3758"[IS] OR "1208-6010"[IS] OR "0008-3674"[IS] OR "1873-7323"[IS] OR "0141-0296"[IS] OR
"2051-8161"[IS] OR "2051-8153"[IS] OR "0261-3794"[IS] OR "0261-3794"[IS] OR "0027-3171"[IS] OR
"1532-7906"[IS] OR "1095-7197"[IS] OR "1064-8275"[IS] OR "0167-4048"[IS] OR "0167-4048"[IS] OR
"2214-9937"[IS] OR "2214-9937"[IS] OR "1095-7103"[IS] OR "0021-9797"[IS] OR "1043-951X"[IS] OR
"1043-951X"[IS] OR "2090-1232"[IS] OR "2090-1232"[IS] OR "2473-4276"[IS] OR "2473-4276"[IS] OR
"2332-8363"[IS] OR "2164-8344"[IS] OR "1749-5024"[IS] OR "1749-5016"[IS] OR "0019-2805"[IS] OR
"1365-2567"[IS] OR "0167-4870"[IS] OR "0167-4870"[IS] OR "0002-9440"[IS] OR "1525-2191"[IS] OR
"2207-9963"[IS] OR "2208-598X"[IS] OR "1468-2850"[IS] OR "0969-5893"[IS] OR "1726-4189"[IS] OR
"1726-4170"[IS] OR "1931-7883"[IS] OR "1931-7891"[IS] OR "1365-2559"[IS] OR "0309-0167"[IS] OR
"0022-1031"[IS] OR "1096-0465"[IS] OR "1469-9923"[IS] OR "1356-3467"[IS] OR "0039-6257"[IS] OR
"1879-3304"[IS] OR "2052-4439"[IS] OR "2052-4439"[IS] OR "0049-0172"[IS] OR "1532-866X"[IS] OR
"0007-1420"[IS] OR "1471-8391"[IS] OR "1129-2377"[IS] OR "1129-2369"[IS] OR "1478-6354"[IS] OR
"1478-6354"[IS] OR "0924-6460"[IS] OR "1573-1502"[IS] OR "1461-670X"[IS] OR "1469-9699"[IS] OR
"1432-0789"[IS] OR "0178-2762"[IS] OR "1861-6909"[IS] OR "1813-7253"[IS] OR "1662-5102"[IS] OR
"1662-5102"[IS] OR "0002-9947"[IS] OR "1088-6850"[IS] OR "1066-2316"[IS] OR "1936-1351"[IS] OR
"2214-5745"[IS] OR "2214-5753"[IS] OR "2059-4798"[IS] OR "2059-4798"[IS] OR "2574-0962"[IS] OR
"2574-0962"[IS] OR "1472-3409"[IS] OR "0308-518X"[IS] OR "0019-0578"[IS] OR "0019-0578"[IS] OR
"1939-5116"[IS] OR "1939-0041"[IS] OR "0168-1699"[IS] OR "0168-1699"[IS] OR "2366-7451"[IS] OR
"2366-7443"[IS] OR "2096-1081"[IS] OR "2509-3312"[IS] OR "1520-0485"[IS] OR "0022-3670"[IS] OR
"1664-073X"[IS] OR "1663-487X"[IS] OR "0360-1323"[IS] OR "0360-1323"[IS] OR "1559-8136"[IS] OR
"1559-8136"[IS] OR "2214-367X"[IS] OR "2214-367X"[IS] OR "2379-8858"[IS] OR "2379-8858"[IS] OR
"1552-731X"[IS] OR "0022-4278"[IS] OR "2047-4881"[IS] OR "2047-4873"[IS] OR "1469-1930"[IS] OR
"1469-1930"[IS] OR "1047-8477"[IS] OR "1095-8657"[IS] OR "1886-1784"[IS] OR "1134-3060"[IS] OR
"1472-8222"[IS] OR "1744-7631"[IS] OR "1059-1524"[IS] OR "1939-4586"[IS] OR "0161-8105"[IS] OR
"1550-9109"[IS] OR "1040-2446"[IS] OR "1938-808X"[IS] OR "2001-0370"[IS] OR "2001-0370"[IS] OR
"2620-0104"[IS] OR "2560-6018"[IS] OR "1556-5653"[IS] OR "0015-0282"[IS] OR "2510-2044"[IS] OR
"2510-2044"[IS] OR "2511-9044"[IS] OR "2511-9044"[IS] OR "1936-0533"[IS] OR "1936-0541"[IS] OR
"0887-6045"[IS] OR "0887-6045"[IS] OR "1753-8416"[IS] OR "1753-8424"[IS] OR "2052-1537"[IS] OR
"2052-1537"[IS] OR "0308-521X"[IS] OR "1873-2267"[IS] OR "0301-3073"[IS] OR "0301-3073"[IS] OR
"1461-7307"[IS] OR "1350-5076"[IS] OR "2378-0231"[IS] OR "2378-0231"[IS] OR "0047-2352"[IS] OR
"0047-2352"[IS] OR "0193-1849"[IS] OR "1522-1555"[IS] OR "1365-3113"[IS] OR "0307-6970"[IS] OR
"0958-5192"[IS] OR "1466-4399"[IS] OR "0270-7306"[IS] OR "1098-5549"[IS] OR "1073-1911"[IS] OR
"1552-3489"[IS] OR "2632-1297"[IS] OR "2632-1297"[IS] OR "1931-7913"[IS] OR "1931-7913"[IS] OR
"0956-2478"[IS] OR "1746-0301"[IS] OR "2509-2715"[IS] OR "2509-2723"[IS] OR "1836-9553"[IS] OR
"1836-9561"[IS] OR "0042-0859"[IS] OR "1552-8340"[IS] OR "1940-8412"[IS] OR "0305-7267"[IS] OR
"0198-9715"[IS] OR "0198-9715"[IS] OR "0032-3217"[IS] OR "1467-9248"[IS] OR "0001-9909"[IS] OR
"1468-2621"[IS] OR "0340-6245"[IS] OR "2567-689X"[IS] OR "1573-6598"[IS] OR "0894-9867"[IS] OR
"1745-9109"[IS] OR "0741-8825"[IS] OR "1523-7052"[IS] OR "1523-7060"[IS] OR "1521-7035"[IS] OR
"1521-6616"[IS] OR "0376-8716"[IS] OR "0376-8716"[IS] OR "2156-3357"[IS] OR "2156-3357"[IS] OR
"1529-6466"[IS] OR "1943-2666"[IS] OR "1529-9430"[IS] OR "1878-1632"[IS] OR "1468-2982"[IS] OR
"0333-1024"[IS] OR "1467-9299"[IS] OR "0033-3298"[IS] OR "0093-3104"[IS] OR "2163-1654"[IS] OR
"1095-5623"[IS] OR "0010-0285"[IS] OR "1570-2820"[IS] OR "1569-3953"[IS] OR "0960-3166"[IS] OR
"1573-5184"[IS] OR "1359-4311"[IS] OR "1359-4311"[IS] OR "1090-2473"[IS] OR "0899-8256"[IS] OR
"1540-7977"[IS] OR "1540-7977"[IS] OR "2644-1284"[IS] OR "2644-1284"[IS] OR "0022-3751"[IS] OR
"1469-7793"[IS] OR "2666-1659"[IS] OR "2666-1659"[IS] OR "0340-5354"[IS] OR "1432-1459"[IS] OR
"0047-2778"[IS] OR "1540-627X"[IS] OR "1468-1331"[IS] OR "1351-5101"[IS] OR "1879-1379"[IS] OR
"0022-3956"[IS] OR "1811-5209"[IS] OR "1811-5209"[IS] OR "8755-2930"[IS] OR "8755-2930"[IS] OR
"1538-4640"[IS] OR "0022-1546"[IS] OR "2405-4569"[IS] OR "2405-4569"[IS] OR "1475-9276"[IS] OR
"1475-9276"[IS] OR "1527-6465"[IS] OR "1527-6473"[IS] OR "2369-2960"[IS] OR "2369-2960"[IS] OR
"2048-7207"[IS] OR "2048-7193"[IS] OR "1879-2596"[IS] OR "0167-4889"[IS] OR "2058-7716"[IS] OR
"2058-7716"[IS] OR "1866-3389"[IS] OR "1866-3370"[IS] OR "1422-8890"[IS] OR "1019-6781"[IS] OR
"1532-785X"[IS] OR "1521-3269"[IS] OR "0924-8463"[IS] OR "1572-8382"[IS] OR "1479-5876"[IS] OR

	"1479-5876"[IS] OR "0899-7667"[IS] OR "1530-888X"[IS] OR "0020-2754"[IS] OR "1475-5661"[IS] OR "1878-4216"[IS] OR "0278-5846"[IS] OR "1549-8328"[IS] OR "1549-8328"[IS] OR "2332-3493"[IS] OR "2332-3507"[IS] OR "1052-6773"[IS] OR "1745-6614"[IS] OR "1861-9533"[IS] OR "0256-1530"[IS] OR "2530-0644"[IS] OR "2530-0644"[IS] OR "2524-6372"[IS] OR "2524-6372"[IS] OR "1098-1004"[IS] OR "1059-7794"[IS] OR "2198-9761"[IS] OR "2198-9761"[IS] OR "2073-4409"[IS] OR "2073-4409"[IS] OR "1530-8561"[IS] OR "0009-9147"[IS] OR "1323-3580"[IS] OR "1448-6083"[IS] OR "1867-0202"[IS] OR "2363-7005"[IS] OR "1862-4065"[IS] OR "1862-4057"[IS] OR "1758-5082"[IS] OR "1758-5090"[IS] OR "1466-4372"[IS] OR "1354-5701"[IS] OR "2050-7534"[IS] OR "2050-7526"[IS] OR "1879-1190"[IS] OR "1072-7515"[IS] OR "1352-4585"[IS] OR "1477-0970"[IS] OR "0031-8205"[IS] OR "1933-1592"[IS] OR "1349-7006"[IS] OR "1347-9032"[IS] OR "0142-0615"[IS] OR "0142-0615"[IS] OR "0020-7403"[IS] OR "0020-7403"[IS] OR "2056-3973"[IS] OR "2056-3973"[IS] OR "2044-5040"[IS] OR "2044-5040"[IS] OR "1868-4483"[IS] OR "0868-601X"[IS] OR "0253-9985"[IS] OR "0253-9985"[IS] OR "2542-5048"[IS] OR "2542-5048"[IS] OR "1389-4978"[IS] OR "1389-4978"[IS] OR "2048-8734"[IS] OR "2048-8726"[IS] OR "0253-2697"[IS] OR "0253-2697"[IS] OR "1434-6052"[IS] OR "1434-6044"[IS] OR "2398-8703"[IS] OR "2398-8703"[IS] OR "0378-3774"[IS] OR "0378-3774"[IS] OR "1554-7191"[IS] OR "1554-7191"[IS] OR "2210-6014"[IS] OR "2210-6006"[IS] OR "1937-0652"[IS] OR "1937-0652"[IS] OR "1939-5086"[IS] OR "1939-5078"[IS] OR "2352-3042"[IS] OR "2352-3042"[IS] OR "0197-4580"[IS] OR "1558-1497"[IS] OR "0029-6643"[IS] OR "1753-4887"[IS] OR "0167-6911"[IS] OR "0167-6911"[IS] OR "1386-4181"[IS] OR "1386-4181"[IS] OR "1050-8392"[IS] OR "1532-7795"[IS] OR "1270-9638"[IS] OR "1270-9638"[IS] OR "0374-1052"[IS] OR "0094-114X"[IS] OR "2509-9426"[IS] OR "2509-9434"[IS] OR "1042-1629"[IS] OR "1556-6501"[IS] OR "2169-9011"[IS] OR "2169-9003"[IS] OR "1949-1042"[IS] OR "1949-1034"[IS] OR "1077-2626"[IS] OR "1077-2626"[IS] OR "2055-7434"[IS] OR "2055-7434"[IS] OR "2698-4016"[IS] OR "2698-4016"[IS] OR "1557-7317"[IS] OR "0001-0782"[IS] OR "1558-2213"[IS] OR "0733-8724"[IS] OR "1758-8340"[IS] OR "1758-8359"[IS] OR "1750-8495"[IS] OR "1750-8487"[IS] OR "2291-5222"[IS] OR "2291-5222"[IS] OR "0264-8172"[IS] OR "1873-4073"[IS] OR "1441-3523"[IS] OR "1441-3523"[IS] OR "1469-994X"[IS] OR "1462-2203"[IS] OR "2333-9683"[IS] OR "2333-9691"[IS] OR "1464-8849"[IS] OR "1741-3001"[IS] OR "1468-7941"[IS] OR "1468-7941"[IS] OR "2198-3402"[IS] OR "2198-2627"[IS] OR "1040-8428"[IS] OR "1040-8428"[IS] OR "2165-0373"[IS] OR "2165-0381"[IS] OR "0926-9959"[IS] OR "1468-3083"[IS] OR "1773-0155"[IS] OR "1774-0746"[IS] OR "2590-1362"[IS] OR "2590-1362"[IS] OR "2468-5844"[IS] OR "2468-5844"[IS] OR "0925-4439"[IS] OR "0925-4439"[IS] OR "1071-9164"[IS] OR "1532-8414"[IS] OR "1528-4042"[IS] OR "1534-6293"[IS] OR "1368-4221"[IS] OR "1368-4221"[IS] OR "1365-2591"[IS] OR "0143-2885"[IS] OR "1873-1740"[IS] OR "0033-8643"[IS] OR "1468-2885"[IS] OR "1050-3293"[IS] OR "1873-4758"[IS] OR "0955-3959"[IS] OR "0142-1123"[IS] OR "0142-1123"[IS] OR "2590-1516"[IS] OR "2590-1516"[IS] OR "1558-5646"[IS] OR "0014-3820"[IS] OR "0927-0248"[IS] OR "0927-0248"[IS] OR "2566-6223"[IS] OR "2566-6223"[IS] OR "1479-683X"[IS] OR "0804-4643"[IS] OR "0734-743X"[IS] OR "0734-743X"[IS] OR "1873-6483"[IS] OR "0740-5472"[IS] OR "1527-1323"[IS] OR "0271-5333"[IS] OR "2192-1962"[IS] OR "2192-1962"[IS] OR "1538-4616"[IS] OR "0022-2879"[IS] OR "2233-6087"[IS] OR "2233-6079"[IS] OR "1097-0134"[IS] OR "0887-3585"[IS] OR "2517-5599"[IS] OR "2517-5599"[IS] OR "1527-1927"[IS] OR "0361-6878"[IS] OR "2328-8957"[IS] OR "2328-8957"[IS] OR "1757-6512"[IS] OR "1757-6512"[IS] OR "1748-1716"[IS] OR "1748-1708"[IS] OR "1467-9310"[IS] OR "0033-6807"[IS] OR "0013-0117"[IS] OR "1468-0289"[IS] OR "0261-4448"[IS] OR "1475-3049"[IS] OR "1878-0334"[IS] OR "1871-4021"[IS] OR "1662-5099"[IS] OR "1662-5099"[IS] OR "2517-729X"[IS] OR "2517-729X"[IS] OR "2044-1398"[IS] OR "2044-1401"[IS] OR "0094-5145"[IS] OR "1573-3610"[IS] OR "2666-9366"[IS] OR "2666-9366"[IS] OR "0264-410X"[IS] OR "1873-2518"[IS] OR "1864-8266"[IS] OR "1864-8258"[IS] OR "2331-7019"[IS] OR "2331-7019"[IS] OR "2363-8354"[IS] OR "2363-8346"[IS] OR "1051-8215"[IS] OR "1051-8215"[IS] OR "0129-0657"[IS] OR "0129-0657"[IS] OR "1932-0620"[IS] OR "1935-3227"[IS] OR "0951-7715"[IS] OR "1361-6544"[IS] OR "0160-791X"[IS] OR "0160-791X"[IS] OR "2041-8396"[IS] OR "2041-840X"[IS] OR "0003-0147"[IS] OR "1537-5323"[IS] OR "0007-1102"[IS] OR "2044-8317"[IS] OR "0025-326X"[IS] OR "1879-3363"[IS] OR "2159-3345"[IS] OR "2159-3337"[IS] OR "1531-6963"[IS] OR "1040-8711"[IS] OR "1754-8403"[IS] OR "1754-8411"[IS] OR "2469-2964"[IS] OR "2469-2964"[IS] OR "2096-0662"[IS] OR "2096-0433"[IS] OR "0300-8126"[IS] OR "1439-0973"[IS] OR "0049-3848"[IS] OR "1879-2472"[IS] OR "1464-4096"[IS] OR "1464-410X"[IS] OR "2311-553X"[IS] OR "2311-553X"[IS] OR "1179-1934"[IS] OR "1172-7047"[IS] OR "0924-0136"[IS] OR "1873-4774"[IS] OR "2380-6761"[IS] OR "2380-6761"[IS] OR "0945-3245"[IS] OR "0029-599X"[IS] OR "1045-2354"[IS] OR "1095-9955"[IS] OR "0938-8249"[IS] OR "1861-8901"[IS] OR "1916-7075"[IS] OR "0828-282X"[IS] OR "0925-4005"[IS] OR "1944-8201"[IS] OR "0038-0385"[IS] OR "1469-8684"[IS] OR "1099-0992"[IS] OR "0046-2772"[IS] OR "0925-2312"[IS] OR "0925-2312"[IS] OR "0927-5371"[IS] OR "0927-5371"[IS] OR "2452-2236"[IS] OR "2452-2236"[IS] OR "2405-6650"[IS] OR "2405-6642"[IS] OR "2042-6976"[IS] OR "2042-6984"[IS] OR "1464-3537"[IS] OR "0007-0882"[IS] OR "1385-4046"[IS] OR "1744-4144"[IS] OR "0022-4537"[IS] OR "1540-4560"[IS] OR "1465-1858"[IS] OR "1465-1858"[IS] OR "1538-4780"[IS] OR

	"0022-2194"[IS] OR "2301-3850"[IS] OR "2301-3869"[IS] OR "0860-021X"[IS] OR "0860-021X"[IS] OR "1441-2772"[IS] OR "1441-2772"[IS] OR "1471-4159"[IS] OR "0022-3042"[IS] OR "0049-4488"[IS] OR "1572-9435"[IS] OR "1520-6505"[IS] OR "1060-1538"[IS] OR "2542-6605"[IS] OR "2542-6605"[IS] OR "2332-6506"[IS] OR "2332-6492"[IS] OR "2575-9108"[IS] OR "2575-9108"[IS] OR "2197-6775"[IS] OR "2197-6775"[IS] OR "0160-6689"[IS] OR "1555-2101"[IS] OR "0341-8162"[IS] OR "0341-8162"[IS] OR "1758-6550"[IS] OR "0957-4093"[IS] OR "0011-9164"[IS] OR "0011-9164"[IS] OR "1572-9761"[IS] OR "0921-2973"[IS] OR "1600-0706"[IS] OR "0030-1299"[IS] OR "0277-6715"[IS] OR "1097-0258"[IS] OR "1470-8752"[IS] OR "0300-5127"[IS] OR "1438-4639"[IS] OR "1438-4639"[IS] OR "0167-4730"[IS] OR "0167-4730"[IS] OR "1938-9507"[IS] OR "8756-9728"[IS] OR "1522-1563"[IS] OR "0363-6143"[IS] OR "1098-108X"[IS] OR "0276-3478"[IS] OR "1086-4415"[IS] OR "1557-9301"[IS] OR "2056-7936"[IS] OR "2056-7936"[IS] OR "2469-9950"[IS] OR "2469-9969"[IS] OR "2730-7166"[IS] OR "2730-7174"[IS] OR "1534-6242"[IS] OR "1523-3804"[IS] OR "2451-9766"[IS] OR "2451-9685"[IS] OR "2687-8941"[IS] OR "2687-8941"[IS] OR "1522-1504"[IS] OR "1040-0605"[IS] OR "2095-3941"[IS] OR "2095-3941"[IS] OR "2152-2715"[IS] OR "2152-2723"[IS] OR "0024-3590"[IS] OR "1939-5590"[IS] OR "2212-1447"[IS] OR "2212-1447"[IS] OR "1000-0976"[IS] OR "1000-0976"[IS] OR "0708-5591"[IS] OR "0708-5591"[IS] OR "2186-9057"[IS] OR "0026-1165"[IS] OR "1545-1151"[IS] OR "1545-1151"[IS] OR "0093-7754"[IS] OR "1532-8708"[IS] OR "0098-3063"[IS] OR "0098-3063"[IS] OR "2589-2991"[IS] OR "2589-2991"[IS] OR "0191-8869"[IS] OR "0191-8869"[IS] OR "1553-2712"[IS] OR "1069-6563"[IS] OR "1469-8366"[IS] OR "0729-4360"[IS] OR "1662-8128"[IS] OR "1662-811X"[IS] OR "2096-7209"[IS] OR "2666-9536"[IS] OR "1861-0692"[IS] OR "1861-0684"[IS] OR "0024-9297"[IS] OR "1520-5835"[IS] OR "1933-7213"[IS] OR "1933-7213"[IS] OR "0009-2541"[IS] OR "0009-2541"[IS] OR "2095-0225"[IS] OR "2095-0217"[IS] OR "1468-2346"[IS] OR "0020-5850"[IS] OR "1757-9880"[IS] OR "1757-9899"[IS] OR "1460-2385"[IS] OR "0931-0509"[IS] OR "1776-260X"[IS] OR "1776-2596"[IS] OR "1740-1445"[IS] OR "1873-6807"[IS] OR "1741-2757"[IS] OR "1465-1165"[IS] OR "0921-8181"[IS] OR "0921-8181"[IS] OR "0141-8955"[IS] OR "1573-2665"[IS] OR "2364-1541"[IS] OR "2364-1185"[IS] OR "0915-5635"[IS] OR "1443-1661"[IS] OR "2504-110X"[IS] OR "2504-110X"[IS] OR "1063-0732"[IS] OR "1466-1853"[IS] OR "1935-9772"[IS] OR "1935-9780"[IS] OR "2352-152X"[IS] OR "2352-152X"[IS] OR "0263-8223"[IS] OR "0263-8223"[IS] OR "1557-9824"[IS] OR "0891-5520"[IS] OR "2196-8837"[IS] OR "2196-8837"[IS] OR "2052-0492"[IS] OR "2052-0492"[IS] OR "2056-6085"[IS] OR "2056-6085"[IS] OR "1365-2982"[IS] OR "1350-1925"[IS] OR "1072-4109"[IS] OR "1533-4031"[IS] OR "1350-7265"[IS] OR "1350-7265"[IS] OR "1090-0268"[IS] OR "1943-5614"[IS] OR "1995-9133"[IS] OR "1684-1182"[IS] OR "1598-9100"[IS] OR "2288-1956"[IS] OR "1748-5983"[IS] OR "1748-5991"[IS] OR "1674-7305"[IS] OR "1869-1889"[IS] OR "1755-4365"[IS] OR "1878-0067"[IS] OR "1353-8292"[IS] OR "1353-8292"[IS] OR "1937-9234"[IS] OR "1932-8184"[IS] OR "1360-8185"[IS] OR "1573-675X"[IS] OR "1741-4326"[IS] OR "0029-5515"[IS] OR "2666-6235"[IS] OR "2666-6235"[IS] OR "2590-0064"[IS] OR "2590-0064"[IS] OR "0933-3657"[IS] OR "1873-2860"[IS] OR "0167-6806"[IS] OR "1573-7217"[IS] OR "0209-9683"[IS] OR "1439-6912"[IS] OR "0924-977X"[IS] OR "1873-7862"[IS] OR "2197-9995"[IS] OR "2197-9987"[IS] OR "1472-4677"[IS] OR "1472-4669"[IS] OR "1091-9856"[IS] OR "1526-5528"[IS] OR "1942-0080"[IS] OR "1941-9651"[IS] OR "1568-9883"[IS] OR "1568-9883"[IS] OR "1362-1688"[IS] OR "1477-0954"[IS] OR "1434-1816"[IS] OR "1435-1102"[IS] OR "2168-7161"[IS] OR "2168-7161"[IS] OR "1098-111X"[IS] OR "0884-8173"[IS] OR "0307-0565"[IS] OR "1476-5497"[IS] OR "1477-9552"[IS] OR "0021-857X"[IS] OR "1132-0559"[IS] OR "2173-4712"[IS] OR "1570-7156"[IS] OR "1573-711X"[IS] OR "1464-3650"[IS] OR "0960-6491"[IS] OR "1997-3500"[IS] OR "1994-4136"[IS] OR "1469-9451"[IS] OR "1369-183X"[IS] OR "0022-202X"[IS] OR "0022-202X"[IS] OR "1526-5471"[IS] OR "0364-765X"[IS] OR "1876-3804"[IS] OR "1876-3804"[IS] OR "2523-0204"[IS] OR "2523-0204"[IS] OR "2687-7910"[IS] OR "2687-7910"[IS] OR "0263-8231"[IS] OR "0263-8231"[IS] OR "1618-8667"[IS] OR "1618-8667"[IS] OR "1467-8543"[IS] OR "0007-1080"[IS] OR "2212-9820"[IS] OR "2212-9820"[IS] OR "1521-690X"[IS] OR "1532-1908"[IS] OR "0090-3752"[IS] OR "1095-9904"[IS] OR "0363-8111"[IS] OR "0363-8111"[IS] OR "2214-7993"[IS] OR "2214-7993"[IS] OR "2212-6864"[IS] OR "2212-6864"[IS] OR "2052-4897"[IS] OR "2052-4897"[IS] OR "1066-5684"[IS] OR "1066-5684"[IS] OR "0362-9805"[IS] OR "1939-9162"[IS] OR "0355-3140"[IS] OR "1795-990X"[IS] OR "0363-9061"[IS] OR "1096-9853"[IS] OR "1073-7928"[IS] OR "1687-0247"[IS] OR "0925-7535"[IS] OR "0925-7535"[IS] OR "0920-9964"[IS] OR "1573-2509"[IS] OR "2051-3933"[IS] OR "2051-3933"[IS] OR "1432-9840"[IS] OR "1435-0629"[IS] OR "1062-6050"[IS] OR "1938-162X"[IS] OR "1061-0421"[IS] OR "1061-0421"[IS] OR "0925-854X"[IS] OR "0925-854X"[IS] OR "0391-173X"[IS] OR "0391-173X"[IS] OR "0169-8095"[IS] OR "0169-8095"[IS] OR "1477-030X"[IS] OR "0269-2163"[IS] OR "1532-4796"[IS] OR "0883-6612"[IS] OR "1572-9419"[IS] OR "1387-3326"[IS] OR "1674-9782"[IS] OR "2049-1891"[IS] OR "2515-7639"[IS] OR "2515-7639"[IS] OR "1477-3155"[IS] OR "1477-3155"[IS] OR "0037-0746"[IS] OR "1365-3091"[IS] OR "2169-9291"[IS] OR "2169-9275"[IS] OR "1529-7535"[IS] OR "1947-3893"[IS] OR "1389-4986"[IS] OR "1573-6695"[IS] OR "2633-1462"[IS] OR "2633-1462"[IS] OR "2296-634X"[IS] OR "2296-634X"[IS] OR "1567-9764"[IS] OR "1573-1553"[IS] OR "2472-5730"[IS] OR "2472-5730"[IS] OR "1471-6402"[IS] OR

"0361-6843"[IS] OR "1098-6596"[IS] OR "0066-4804"[IS] OR "1559-064X"[IS] OR "1559-0631"[IS] OR "1553-040X"[IS] OR "1553-040X"[IS] OR "1477-7517"[IS] OR "1477-7517"[IS] OR "2194-7511"[IS] OR "2194-7511"[IS] OR "1095-9513"[IS] OR "1055-7903"[IS] OR "1937-3368"[IS] OR "1937-3376"[IS] OR "1098-3015"[IS] OR "1524-4733"[IS] OR "1931-857X"[IS] OR "1931-857X"[IS] OR "1432-0916"[IS] OR "0010-3616"[IS] OR "1134-3478"[IS] OR "1134-3478"[IS] OR "1440-1711"[IS] OR "0818-9641"[IS] OR "0217-4561"[IS] OR "0217-4561"[IS] OR "0017-257X"[IS] OR "1477-7053"[IS] OR "0268-0939"[IS] OR "1464-5106"[IS] OR "2368-7959"[IS] OR "2368-7959"[IS] OR "1469-7645"[IS] OR "0022-1120"[IS] OR "2211-3436"[IS] OR "2211-3428"[IS] OR "1559-2308"[IS] OR "1559-2294"[IS] OR "2213-4530"[IS] OR "2213-4530"[IS] OR "0963-6625"[IS] OR "1361-6609"[IS] OR "2161-5063"[IS] OR "2161-5063"[IS] OR "0002-5100"[IS] OR "0002-5100"[IS] OR "1461-1457"[IS] OR "1469-5111"[IS] OR "1532-2939"[IS] OR "0195-6701"[IS] OR "1557-2862"[IS] OR "1557-2862"[IS] OR "1557-9700"[IS] OR "1075-2730"[IS] OR "0213-2230"[IS] OR "0213-2230"[IS] OR "1564-698X"[IS] OR "0258-6770"[IS] OR "0197-3975"[IS] OR "0197-3975"[IS] OR "1049-8923"[IS] OR "1099-1239"[IS] OR "1432-2242"[IS] OR "0040-5752"[IS] OR "1863-9704"[IS] OR "1863-9690"[IS] OR "1440-1592"[IS] OR "1323-8930"[IS] OR "1879-0771"[IS] OR "0895-6111"[IS] OR "2050-5094"[IS] OR "2050-5094"[IS] OR "1930-7810"[IS] OR "0278-6133"[IS] OR "1464-5300"[IS] OR "1364-5579"[IS] OR "0737-4607"[IS] OR "0737-4607"[IS] OR "0969-6997"[IS] OR "0969-6997"[IS] OR "1545-5300"[IS] OR "0014-7370"[IS] OR "2514-4820"[IS] OR "2514-4820"[IS] OR "1530-9134"[IS] OR "1058-6407"[IS] OR "0007-0998"[IS] OR "2044-8279"[IS] OR "1753-2000"[IS] OR "1753-2000"[IS] OR "0099-2399"[IS] OR "1878-3554"[IS] OR "0022-1295"[IS] OR "1540-7748"[IS] OR "1364-3703"[IS] OR "1464-6722"[IS] OR "1878-5832"[IS] OR "1359-6446"[IS] OR "0018-9456"[IS] OR "1557-9662"[IS] OR "1524-9557"[IS] OR "1537-4513"[IS] OR "1097-184X"[IS] OR "1097-184X"[IS] OR "1931-390X"[IS] OR "1931-3896"[IS] OR "1470-160X"[IS] OR "1470-160X"[IS] OR "1872-6852"[IS] OR "0378-4290"[IS] OR "1938-1344"[IS] OR "0190-6011"[IS] OR "2165-1442"[IS] OR "2165-1434"[IS] OR "0140-3664"[IS] OR "0140-3664"[IS] OR "1098-2787"[IS] OR "0277-7037"[IS] OR "2213-1582"[IS] OR "2213-1582"[IS] OR "0146-6380"[IS] OR "0146-6380"[IS] OR "2051-6673"[IS] OR "2051-6673"[IS] OR "1557-9859"[IS] OR "0025-7125"[IS] OR "2156-8731"[IS] OR "2156-8693"[IS] OR "2366-7486"[IS] OR "2366-7486"[IS] OR "0960-0779"[IS] OR "0960-0779"[IS] OR "0378-3820"[IS] OR "0378-3820"[IS] OR "1090-7165"[IS] OR "1573-3254"[IS] OR "1867-2469"[IS] OR "1867-2450"[IS] OR "1872-4973"[IS] OR "1872-4973"[IS] OR "1001-0742"[IS] OR "1878-7320"[IS] OR "1867-1381"[IS] OR "1867-8548"[IS] OR "2452-3100"[IS] OR "2452-3100"[IS] OR "1462-2912"[IS] OR "1462-2920"[IS] OR "2297-055X"[IS] OR "2297-055X"[IS] OR "0306-4530"[IS] OR "1873-3360"[IS] OR "1793-6683"[IS] OR "0219-1997"[IS] OR "2157-6564"[IS] OR "2157-6580"[IS] OR "1001-8417"[IS] OR "1001-8417"[IS] OR "1170-7690"[IS] OR "1179-2027"[IS] OR "1440-1819"[IS] OR "1323-1316"[IS] OR "1053-4296"[IS] OR "1532-9461"[IS] OR "1549-7828"[IS] OR "1040-841X"[IS] OR "0167-7187"[IS] OR "0167-7187"[IS] OR "2056-7189"[IS] OR "2056-7189"[IS] OR "1470-109X"[IS] OR "1361-3324"[IS] OR "0730-8884"[IS] OR "1552-8464"[IS] OR "2452-2139"[IS] OR "2452-2139"[IS] OR "1534-4827"[IS] OR "1539-0829"[IS] OR "2666-3740"[IS] OR "2666-3740"[IS] OR "0967-0661"[IS] OR "0967-0661"[IS] OR "2576-988X"[IS] OR "2576-9898"[IS] OR "2491-6765"[IS] OR "2491-6765"[IS] OR "1530-6860"[IS] OR "0892-6638"[IS] OR "1934-9297"[IS] OR "1934-9300"[IS] OR "2472-7245"[IS] OR "2472-7245"[IS] OR "1476-5500"[IS] OR "0929-1903"[IS] OR "2096-2320"[IS] OR "2589-5532"[IS] OR "1674-9278"[IS] OR "1674-9278"[IS] OR "0193-1857"[IS] OR "1522-1547"[IS] OR "1863-2513"[IS] OR "1863-2505"[IS] OR "1464-3642"[IS] OR "0272-4979"[IS] OR "2468-2152"[IS] OR "2468-0427"[IS] OR "1460-2377"[IS] OR "0953-8178"[IS] OR "1093-7404"[IS] OR "1521-6950"[IS] OR "1523-0899"[IS] OR "1523-0899"[IS] OR "0305-7453"[IS] OR "1460-2091"[IS] OR "0006-8314"[IS] OR "1573-1472"[IS] OR "1755-5949"[IS] OR "1755-5930"[IS] OR "2691-4581"[IS] OR "2691-4581"[IS] OR "0014-4886"[IS] OR "1090-2430"[IS] OR "0016-2361"[IS] OR "1873-7153"[IS] OR "1866-1947"[IS] OR "1866-1955"[IS] OR "1872-6216"[IS] OR "0047-6374"[IS] OR "1527-8999"[IS] OR "1528-0691"[IS] OR "1363-2434"[IS] OR "1364-2626"[IS] OR "1522-2586"[IS] OR "1053-1807"[IS] OR "0065-2911"[IS] OR "0065-2911"[IS] OR "2589-8116"[IS] OR "2589-8116"[IS] OR "2223-7704"[IS] OR "2223-7690"[IS] OR "2589-3823"[IS] OR "2589-3831"[IS] OR "1558-1691"[IS] OR "0364-9059"[IS] OR "1095-8568"[IS] OR "0022-460X"[IS] OR "1574-0862"[IS] OR "0169-5150"[IS] OR "0021-9150"[IS] OR "1879-1484"[IS] OR "0895-9048"[IS] OR "1552-3896"[IS] OR "1466-4437"[IS] OR "0268-3962"[IS] OR "2213-1388"[IS] OR "2213-1388"[IS] OR "1752-4571"[IS] OR "1752-4571"[IS] OR "1759-6653"[IS] OR "1759-6653"[IS] OR "1525-755X"[IS] OR "1525-7541"[IS] OR "0022-0167"[IS] OR "0022-0167"[IS] OR "0309-1740"[IS] OR "1873-4138"[IS] OR "0038-092X"[IS] OR "0038-092X"[IS] OR "1557-7813"[IS] OR "0885-3134"[IS] OR "1534-6080"[IS] OR "0041-1337"[IS] OR "1533-0028"[IS] OR "1533-0028"[IS] OR "1365-2699"[IS] OR "0305-0270"[IS] OR "0025-729X"[IS] OR "1326-5377"[IS] OR "0032-0781"[IS] OR "1471-9053"[IS] OR "1554-8937"[IS] OR "1554-8929"[IS] OR "1598-2629"[IS] OR "2092-6685"[IS] OR "0017-9310"[IS] OR "0017-9310"[IS] OR "1929-7750"[IS] OR "1929-7750"[IS] OR "1350-6129"[IS] OR "1744-2818"[IS] OR "2397-0642"[IS] OR "2397-0642"[IS] OR "1750-2640"[IS] OR "1750-2659"[IS] OR "1932-2267"[IS] OR "1932-2259"[IS] OR "2047-2994"[IS] OR "2047-2994"[IS] OR "1873-3425"[IS] OR

	"1041-6080"[IS] OR "0753-3322"[IS] OR "0753-3322"[IS] OR "1367-6539"[IS] OR "1468-9618"[IS] OR "0969-7128"[IS] OR "1476-5462"[IS] OR "2056-3485"[IS] OR "2056-3485"[IS] OR "1526-7598"[IS] OR "0003-2999"[IS] OR "2590-1745"[IS] OR "2590-1745"[IS] OR "1526-5900"[IS] OR "1528-8447"[IS] OR "0195-9255"[IS] OR "0195-9255"[IS] OR "1098-2264"[IS] OR "1045-2257"[IS] OR "2352-3808"[IS] OR "2352-3808"[IS] OR "1359-1789"[IS] OR "1359-1789"[IS] OR "0963-9969"[IS] OR "0963-9969"[IS] OR "0733-9445"[IS] OR "0733-9445"[IS] OR "1939-5108"[IS] OR "1939-0068"[IS] OR "2155-384X"[IS] OR "2155-384X"[IS] OR "0010-2571"[IS] OR "1420-8946"[IS] OR "2196-632X"[IS] OR "2196-6311"[IS] OR "1743-923X"[IS] OR "1743-9248"[IS] OR "1000-6818"[IS] OR "1000-6818"[IS] OR "1049-8931"[IS] OR "1557-0657"[IS] OR "1549-960X"[IS] OR "1549-9596"[IS] OR "1096-3480"[IS] OR "1557-7554"[IS] OR "1361-6471"[IS] OR "0954-3899"[IS] OR "1046-2023"[IS] OR "1095-9130"[IS] OR "1040-2608"[IS] OR "1040-2608"[IS] OR "1433-8491"[IS] OR "0940-1334"[IS] OR "0969-7764"[IS] OR "1461-7145"[IS] OR "0092-6566"[IS] OR "1095-7251"[IS] OR "1520-0434"[IS] OR "0882-8156"[IS] OR "0265-9247"[IS] OR "1521-1878"[IS] OR "1466-4461"[IS] OR "1366-9877"[IS] OR "0962-6298"[IS] OR "0962-6298"[IS] OR "1470-8736"[IS] OR "0143-5221"[IS] OR "1478-4505"[IS] OR "1478-4505"[IS] OR "2168-6750"[IS] OR "2168-6750"[IS] OR "1529-4242"[IS] OR "0032-1052"[IS] OR "0005-7894"[IS] OR "1878-1888"[IS] OR "1945-0818"[IS] OR "1539-1523"[IS] OR "1369-8478"[IS] OR "1369-8478"[IS] OR "0016-7185"[IS] OR "0016-7185"[IS] OR "2329-924X"[IS] OR "2329-924X"[IS] OR "2632-2153"[IS] OR "2632-2153"[IS] OR "1469-0292"[IS] OR "1469-0292"[IS] OR "1399-3089"[IS] OR "0908-665X"[IS] OR "1364-8152"[IS] OR "1364-8152"[IS] OR "2666-9110"[IS] OR "2666-9110"[IS] OR "0938-8974"[IS] OR "1432-1467"[IS] OR "1869-9510"[IS] OR "1869-9529"[IS] OR "1350-4177"[IS] OR "1873-2828"[IS] OR "1472-0701"[IS] OR "1472-0701"[IS] OR "1432-1122"[IS] OR "0949-2984"[IS] OR "2688-1527"[IS] OR "2688-1535"[IS] OR "0887-3801"[IS] OR "0887-3801"[IS] OR "1526-6125"[IS] OR "1526-6125"[IS] OR "0740-3194"[IS] OR "1522-2594"[IS] OR "0006-341X"[IS] OR "1541-0420"[IS] OR "0147-6513"[IS] OR "1090-2414"[IS] OR "2329-9304"[IS] OR "2329-9290"[IS] OR "2161-430X"[IS] OR "1077-6990"[IS] OR "1229-6929"[IS] OR "2005-8330"[IS] OR "1097-6817"[IS] OR "0194-5998"[IS] OR "0882-7974"[IS] OR "1939-1498"[IS] OR "0038-9765"[IS] OR "0038-9765"[IS] OR "0301-679X"[IS] OR "0301-679X"[IS] OR "2398-502X"[IS] OR "2398-502X"[IS] OR "1352-2310"[IS] OR "1873-2844"[IS] OR "1096-0031"[IS] OR "0748-3007"[IS] OR "0956-540X"[IS] OR "1365-246X"[IS] OR "0362-546X"[IS] OR "0362-546X"[IS] OR "2652-3310"[IS] OR "2709-2119"[IS] OR "1476-0584"[IS] OR "1744-8395"[IS] OR "2377-3782"[IS] OR "2377-3782"[IS] OR "0013-7227"[IS] OR "1945-7170"[IS] OR "1465-7341"[IS] OR "8756-6222"[IS] OR "0025-1747"[IS] OR "0025-1747"[IS] OR "1540-5958"[IS] OR "0048-8986"[IS] OR "0034-6446"[IS] OR "1936-4814"[IS] OR "2515-4478"[IS] OR "2515-446X"[IS] OR "1364-548X"[IS] OR "1359-7345"[IS] OR "2095-2201"[IS] OR "2095-221X"[IS] OR "1758-1095"[IS] OR "0141-0768"[IS] OR "1749-8104"[IS] OR "1749-8104"[IS] OR "1024-5294"[IS] OR "1477-2221"[IS] OR "1399-5448"[IS] OR "1399-543X"[IS] OR "0010-7514"[IS] OR "1366-5812"[IS] OR "1098-3058"[IS] OR "1943-4294"[IS] OR "1598-2998"[IS] OR "2005-9256"[IS] OR "1532-2165"[IS] OR "1078-5884"[IS] OR "0966-0429"[IS] OR "1365-2834"[IS] OR "2643-6515"[IS] OR "2643-6515"[IS] OR "2049-937X"[IS] OR "2049-9361"[IS] OR "2214-594X"[IS] OR "2214-594X"[IS] OR "1740-6749"[IS] OR "1740-6749"[IS] OR "1548-1603"[IS] OR "1548-1603"[IS] OR "1535-3893"[IS] OR "1535-3907"[IS] OR "1526-0550"[IS] OR "1526-0542"[IS] OR "2666-3899"[IS] OR "2666-3899"[IS] OR "1420-908X"[IS] OR "1023-3830"[IS] OR "1042-1726"[IS] OR "1867-1233"[IS] OR "2372-7705"[IS] OR "2372-7705"[IS] OR "1383-5866"[IS] OR "1873-3794"[IS] OR "1066-5099"[IS] OR "1549-4918"[IS] OR "2214-2894"[IS] OR "2214-2894"[IS] OR "0018-9251"[IS] OR "0018-9251"[IS] OR "0047-2484"[IS] OR "1095-8606"[IS] OR "1744-3598"[IS] OR "0957-5820"[IS] OR "0021-8251"[IS] OR "0021-8251"[IS] OR "1061-5806"[IS] OR "1477-2205"[IS] OR "1072-5245"[IS] OR "1573-3424"[IS] OR "1573-1480"[IS] OR "0165-0009"[IS] OR "1939-8425"[IS] OR "1939-8433"[IS] OR "1552-3373"[IS] OR "1532-673X"[IS] OR "0066-2313"[IS] OR "0066-2313"[IS] OR "0954-6111"[IS] OR "1532-3064"[IS] OR "0008-3976"[IS] OR "1744-7976"[IS] OR "1555-0265"[IS] OR "1555-0273"[IS] OR "2157-1716"[IS] OR "2157-1724"[IS] OR "1520-5436"[IS] OR "1520-5436"[IS] OR "1695-6885"[IS] OR "1695-6885"[IS] OR "1940-4026"[IS] OR "0896-4289"[IS] OR "1361-4916"[IS] OR "1474-0044"[IS] OR "2052-1545"[IS] OR "2052-1553"[IS] OR "1600-0838"[IS] OR "0905-7188"[IS] OR "0303-1179"[IS] OR "0303-1179"[IS] OR "0950-091X"[IS] OR "1365-2117"[IS] OR "1873-2968"[IS] OR "0006-2952"[IS] OR "2772-3909"[IS] OR "2772-3909"[IS] OR "0306-4603"[IS] OR "0306-4603"[IS] OR "1673-5447"[IS] OR "1673-5447"[IS] OR "1743-8764"[IS] OR "1352-3260"[IS] OR "1941-0921"[IS] OR "1941-7381"[IS] OR "2332-8584"[IS] OR "2332-8584"[IS] OR "0268-3369"[IS] OR "1476-5365"[IS] OR "1618-1298"[IS] OR "0171-9335"[IS] OR "2198-1639"[IS] OR "2198-1620"[IS] OR "1876-2018"[IS] OR "1876-2026"[IS] OR "0021-8898"[IS] OR "1600-5767"[IS] OR "0029-8018"[IS] OR "0029-8018"[IS] OR "1001-0521"[IS] OR "1867-7185"[IS] OR "2058-5241"[IS] OR "2396-7544"[IS] OR "1877-718X"[IS] OR "1877-7171"[IS] OR "0893-7648"[IS] OR "1559-1182"[IS] OR "0171-8177"[IS] OR "0171-8177"[IS] OR "1878-1861"[IS] OR "1440-2440"[IS] OR "1942-0994"[IS] OR "1942-0900"[IS] OR "1544-1717"[IS] OR "1544-1709"[IS] OR "1939-3792"[IS] OR "1939-3806"[IS] OR "1366-2716"[IS] OR "1469-8390"[IS] OR "1475-1585"[IS] OR "1475-1585"[IS] OR "1940-5480"[IS] OR

"1067-151X"[IS] OR "0340-1022"[IS] OR "0340-1022"[IS] OR "1432-0924"[IS] OR "0178-7675"[IS] OR
"0954-349X"[IS] OR "0954-349X"[IS] OR "0091-0562"[IS] OR "1573-2770"[IS] OR "2475-0379"[IS] OR
"2475-0379"[IS] OR "1534-6307"[IS] OR "1523-3774"[IS] OR "1072-6349"[IS] OR "1751-7613"[IS] OR
"0143-974X"[IS] OR "0143-974X"[IS] OR "1756-5529"[IS] OR "1756-5537"[IS] OR "0360-3199"[IS] OR
"0360-3199"[IS] OR "1475-5785"[IS] OR "1353-8047"[IS] OR "0741-3106"[IS] OR "0741-3106"[IS] OR
"0743-0167"[IS] OR "0743-0167"[IS] OR "2196-7350"[IS] OR "2196-7350"[IS] OR "0954-5794"[IS] OR
"1469-2198"[IS] OR "1365-8816"[IS] OR "1365-8824"[IS] OR "1360-0540"[IS] OR "0260-7476"[IS] OR
"1534-6269"[IS] OR "1523-3790"[IS] OR "1359-0294"[IS] OR "1359-0294"[IS] OR "2590-0552"[IS] OR
"2590-0552"[IS] OR "1473-0197"[IS] OR "1473-0189"[IS] OR "0360-0025"[IS] OR "1573-2762"[IS] OR
"1476-069X"[IS] OR "1476-069X"[IS] OR "2072-6694"[IS] OR "2072-6694"[IS] OR "0010-4655"[IS] OR
"0010-4655"[IS] OR "1360-0516"[IS] OR "0954-0253"[IS] OR "0022-2828"[IS] OR "1095-8584"[IS] OR
"1863-6683"[IS] OR "1863-6691"[IS] OR "2297-3486"[IS] OR "2297-3508"[IS] OR "0898-929X"[IS] OR
"1530-8898"[IS] OR "0926-7220"[IS] OR "0926-7220"[IS] OR "1557-9042"[IS] OR "0897-7151"[IS] OR
"1612-4758"[IS] OR "1612-4766"[IS] OR "0790-8318"[IS] OR "0790-8318"[IS] OR "0195-6574"[IS] OR
"0195-6574"[IS] OR "2373-2822"[IS] OR "2373-2822"[IS] OR "0265-1335"[IS] OR "0265-1335"[IS] OR
"1461-7269"[IS] OR "0958-9287"[IS] OR "0040-1951"[IS] OR "0040-1951"[IS] OR "1365-3121"[IS] OR
"0954-4879"[IS] OR "2235-2988"[IS] OR "2235-2988"[IS] OR "1662-5110"[IS] OR "1662-5110"[IS] OR
"1473-9542"[IS] OR "1479-7364"[IS] OR "0261-5606"[IS] OR "0261-5606"[IS] OR "1086-296X"[IS] OR
"1554-8430"[IS] OR "1557-1890"[IS] OR "1557-1904"[IS] OR "0033-3123"[IS] OR "1860-0980"[IS] OR
"0271-3586"[IS] OR "1097-0274"[IS] OR "1471-2458"[IS] OR "1471-2458"[IS] OR "1567-4223"[IS] OR
"1567-4223"[IS] OR "0028-3908"[IS] OR "1873-7064"[IS] OR "0014-4029"[IS] OR "2163-5560"[IS] OR
"0268-3946"[IS] OR "0268-3946"[IS] OR "1873-6513"[IS] OR "0885-3924"[IS] OR "1545-9683"[IS] OR
"1545-9683"[IS] OR "0033-1538"[IS] OR "0033-1538"[IS] OR "1897-4317"[IS] OR "1895-5770"[IS] OR
"1754-4769"[IS] OR "1754-4750"[IS] OR "0952-1895"[IS] OR "1468-0491"[IS] OR "2469-9985"[IS] OR
"2469-9993"[IS] OR "2312-0541"[IS] OR "2312-0541"[IS] OR "1973-8102"[IS] OR "0010-9452"[IS] OR
"0264-9993"[IS] OR "0264-9993"[IS] OR "2050-1579"[IS] OR "2050-1587"[IS] OR "1080-5370"[IS] OR
"1080-5370"[IS] OR "1365-2869"[IS] OR "0962-1105"[IS] OR "0025-5718"[IS] OR "1088-6842"[IS] OR
"0167-8655"[IS] OR "0167-8655"[IS] OR "1098-7339"[IS] OR "1532-8651"[IS] OR "1570-8705"[IS] OR
"1570-8705"[IS] OR "0361-803X"[IS] OR "1546-3141"[IS] OR "0014-2972"[IS] OR "1365-2362"[IS] OR
"1064-8011"[IS] OR "1533-4287"[IS] OR "1477-0334"[IS] OR "0962-2802"[IS] OR "1541-2040"[IS] OR
"1541-2040"[IS] OR "1549-7836"[IS] OR "0735-2689"[IS] OR "2050-7887"[IS] OR "2050-7895"[IS] OR
"2210-5433"[IS] OR "2210-5441"[IS] OR "1099-2340"[IS] OR "1522-1970"[IS] OR "1935-2727"[IS] OR
"1935-2735"[IS] OR "1367-8868"[IS] OR "1469-8374"[IS] OR "0924-8579"[IS] OR "1872-7913"[IS] OR
"2211-5684"[IS] OR "2211-5714"[IS] OR "1558-1721"[IS] OR "0018-9529"[IS] OR "0094-730X"[IS] OR
"1873-801X"[IS] OR "2211-5374"[IS] OR "2211-5366"[IS] OR "0305-5736"[IS] OR "1470-8442"[IS] OR
"0717-6287"[IS] OR "0716-9760"[IS] OR "2399-3421"[IS] OR "2399-3421"[IS] OR "1097-4652"[IS] OR
"0021-9541"[IS] OR "2008-9244"[IS] OR "2193-8865"[IS] OR "1440-1843"[IS] OR "1323-7799"[IS] OR
"1920-4531"[IS] OR "1920-454X"[IS] OR "0168-2563"[IS] OR "1573-515X"[IS] OR "1744-9561"[IS] OR
"1744-957X"[IS] OR "0959-9851"[IS] OR "1619-1560"[IS] OR "1099-9809"[IS] OR "1939-0106"[IS] OR
"1532-1096"[IS] OR "1044-8004"[IS] OR "1531-636X"[IS] OR "1531-636X"[IS] OR "1095-9254"[IS] OR
"0140-1971"[IS] OR "1537-1948"[IS] OR "0025-7079"[IS] OR "1354-3776"[IS] OR "1744-7674"[IS] OR
"1615-4169"[IS] OR "1615-4150"[IS] OR "1471-244X"[IS] OR "1471-244X"[IS] OR "1744-1706"[IS] OR
"1744-1692"[IS] OR "2373-776X"[IS] OR "2373-776X"[IS] OR "2072-6643"[IS] OR "2072-6643"[IS] OR
"2196-2952"[IS] OR "2196-2952"[IS] OR "1354-3784"[IS] OR "1744-7658"[IS] OR "1083-4389"[IS] OR
"1523-5378"[IS] OR "1477-0326"[IS] OR "0267-6583"[IS] OR "1999-4915"[IS] OR "1999-4915"[IS] OR
"1661-8564"[IS] OR "1661-8556"[IS] OR "2056-4538"[IS] OR "2056-4538"[IS] OR "2379-5042"[IS] OR
"2379-5042"[IS] OR "1382-3256"[IS] OR "1573-7616"[IS] OR "1355-2554"[IS] OR "1355-2554"[IS] OR
"1943-0620"[IS] OR "1943-0620"[IS] OR "0042-3114"[IS] OR "1744-5159"[IS] OR "0037-1106"[IS] OR
"1943-3573"[IS] OR "2009-8774"[IS] OR "2305-6983"[IS] OR "2047-2986"[IS] OR "2047-2978"[IS] OR
"2426-8399"[IS] OR "2426-8399"[IS] OR "2169-8953"[IS] OR "2169-8961"[IS] OR "0924-090X"[IS] OR
"1573-269X"[IS] OR "1545-4290"[IS] OR "0084-6570"[IS] OR "1545-598X"[IS] OR "1545-598X"[IS] OR
"0363-6135"[IS] OR "1522-1539"[IS] OR "1818-0876"[IS] OR "2221-285X"[IS] OR "1470-8728"[IS] OR
"0264-6021"[IS] OR "0109-5641"[IS] OR "1879-0097"[IS] OR "0017-8748"[IS] OR "1526-4610"[IS] OR
"2044-8341"[IS] OR "1476-0835"[IS] OR "0925-5214"[IS] OR "0925-5214"[IS] OR "1095-7138"[IS] OR
"0363-0129"[IS] OR "1025-3866"[IS] OR "1477-223X"[IS] OR "0013-7944"[IS] OR "0013-7944"[IS] OR
"0927-5398"[IS] OR "0927-5398"[IS] OR "2327-0039"[IS] OR "2327-0012"[IS] OR "1365-2958"[IS] OR
"0950-382X"[IS] OR "1042-9832"[IS] OR "1098-2418"[IS] OR "1522-8037"[IS] OR "1534-312X"[IS] OR
"2639-5908"[IS] OR "2639-5916"[IS] OR "1059-0145"[IS] OR "1573-1839"[IS] OR "2589-4196"[IS] OR
"2589-4196"[IS] OR "0078-6179"[IS] OR "0078-6179"[IS] OR "2055-5563"[IS] OR "2055-5571"[IS] OR
"2214-2126"[IS] OR "2214-2134"[IS] OR "2052-4110"[IS] OR "2052-4129"[IS] OR "0002-7316"[IS] OR

	"2325-5064"[IS] OR "1664-2392"[IS] OR "1664-2392"[IS] OR "1525-4135"[IS] OR "1944-7884"[IS] OR "1479-6805"[IS] OR "0022-0795"[IS] OR "1812-0792"[IS] OR "1812-0784"[IS] OR "2162-0989"[IS] OR "2162-0989"[IS] OR "2212-4934"[IS] OR "2212-4926"[IS] OR "1552-6259"[IS] OR "0003-4975"[IS] OR "0014-5793"[IS] OR "1873-3468"[IS] OR "0946-2716"[IS] OR "1432-1440"[IS] OR "1383-7621"[IS] OR "1383-7621"[IS] OR "1095-8347"[IS] OR "0890-8389"[IS] OR "1538-4667"[IS] OR "0196-0202"[IS] OR "0959-6658"[IS] OR "1460-2423"[IS] OR "2468-2047"[IS] OR "2468-080X"[IS] OR "0275-5319"[IS] OR "0275-5319"[IS] OR "1556-4681"[IS] OR "1556-4681"[IS] OR "1460-2210"[IS] OR "0141-5387"[IS] OR "1552-6801"[IS] OR "1077-5587"[IS] OR "1084-0702"[IS] OR "1943-5592"[IS] OR "1361-2026"[IS] OR "1361-2026"[IS] OR "1532-7752"[IS] OR "0022-3891"[IS] OR "1879-0593"[IS] OR "1368-8375"[IS] OR "1478-7210"[IS] OR "1744-8336"[IS] OR "2215-1486"[IS] OR "2215-1478"[IS] OR "0022-3492"[IS] OR "1943-3670"[IS] OR "2473-2877"[IS] OR "2473-2877"[IS] OR "1532-3080"[IS] OR "0960-9776"[IS] OR "1466-8564"[IS] OR "1466-8564"[IS] OR "1751-1577"[IS] OR "1751-1577"[IS] OR "1760-4788"[IS] OR "1279-7707"[IS] OR "2046-4053"[IS] OR "2046-4053"[IS] OR "0003-9985"[IS] OR "0003-9985"[IS] OR "1460-2237"[IS] OR "0268-1080"[IS] OR "0165-0254"[IS] OR "1464-0651"[IS] OR "1095-9300"[IS] OR "1071-5819"[IS] OR "1557-1882"[IS] OR "1557-1874"[IS] OR "1434-6621"[IS] OR "1437-4331"[IS] OR "2096-4862"[IS] OR "2523-3246"[IS] OR "0272-7757"[IS] OR "0272-7757"[IS] OR "1558-3791"[IS] OR "1549-7747"[IS] OR "1878-0199"[IS] OR "1755-2966"[IS] OR "1467-7660"[IS] OR "0012-155X"[IS] OR "2666-3546"[IS] OR "2666-3546"[IS] OR "1361-6382"[IS] OR "0264-9381"[IS] OR "0159-8090"[IS] OR "0159-8090"[IS] OR "0013-4686"[IS] OR "0013-4686"[IS] OR "1878-5174"[IS] OR "0256-2928"[IS] OR "2151-1535"[IS] OR "1939-1404"[IS] OR "0309-1708"[IS] OR "0309-1708"[IS] OR "1878-4119"[IS] OR "1010-5182"[IS] OR "2372-7330"[IS] OR "2372-7322"[IS] OR "0003-004X"[IS] OR "1945-3027"[IS] OR "1614-7065"[IS] OR "1001-604X"[IS] OR "1552-681X"[IS] OR "0272-989X"[IS] OR "0969-7330"[IS] OR "1477-0989"[IS] OR "1747-5139"[IS] OR "1475-939X"[IS] OR "1473-5571"[IS] OR "0269-9370"[IS] OR "1461-6734"[IS] OR "1469-2988"[IS] OR "1534-4320"[IS] OR "1558-0210"[IS] OR "0278-4254"[IS] OR "0278-4254"[IS] OR "1549-5787"[IS] OR "1549-5787"[IS] OR "0045-7949"[IS] OR "0045-7949"[IS] OR "1878-531X"[IS] OR "1016-9040"[IS] OR "0015-198X"[IS] OR "0015-198X"[IS] OR "0146-0404"[IS] OR "1552-5783"[IS] OR "1467-6451"[IS] OR "0022-1821"[IS] OR "1476-9255"[IS] OR "1476-9255"[IS] OR "1868-8535"[IS] OR "1868-8527"[IS] OR "2212-2761"[IS] OR "2212-277X"[IS] OR "2471-2531"[IS] OR "2471-2531"[IS] OR "0005-2728"[IS] OR "1879-2650"[IS] OR "1873-619X"[IS] OR "0955-2219"[IS] OR "2057-5858"[IS] OR "2057-5858"[IS] OR "2398-4902"[IS] OR "2398-4902"[IS] OR "2666-0164"[IS] OR "2666-0164"[IS] OR "1933-2874"[IS] OR "1933-2874"[IS] OR "1468-1218"[IS] OR "1468-1218"[IS] OR "0032-2687"[IS] OR "1573-0891"[IS] OR "1351-0088"[IS] OR "1351-0088"[IS] OR "2637-6407"[IS] OR "2637-6407"[IS] OR "1360-0486"[IS] OR "0305-0068"[IS] OR "1552-4922"[IS] OR "1552-4930"[IS] OR "2691-3321"[IS] OR "2691-3321"[IS] OR "0040-5841"[IS] OR "1543-0421"[IS] OR "2399-6552"[IS] OR "2399-6544"[IS] OR "1540-9996"[IS] OR "1931-843X"[IS] OR "0267-7261"[IS] OR "0267-7261"[IS] OR "1741-203X"[IS] OR "1041-6102"[IS] OR "0022-4499"[IS] OR "1559-8519"[IS] OR "0033-3549"[IS] OR "1468-2877"[IS] OR "2055-7647"[IS] OR "2055-7647"[IS] OR "1360-2357"[IS] OR "1360-2357"[IS] OR "1573-7322"[IS] OR "1382-4147"[IS] OR "1386-6346"[IS] OR "1386-6346"[IS] OR "1651-2278"[IS] OR "1403-6096"[IS] OR "0168-9452"[IS] OR "1873-2259"[IS] OR "1369-5258"[IS] OR "1469-3569"[IS] OR "1573-5001"[IS] OR "0925-2738"[IS] OR "1879-2308"[IS] OR "0166-0462"[IS] OR "1403-4948"[IS] OR "1651-1905"[IS] OR "1464-5491"[IS] OR "0742-3071"[IS] OR "1098-1063"[IS] OR "1050-9631"[IS] OR "1573-188X"[IS] OR "0361-0365"[IS] OR "1096-9861"[IS] OR "0021-9967"[IS] OR "1525-1578"[IS] OR "1943-7811"[IS] OR "1076-1551"[IS] OR "1528-3658"[IS] OR "1388-2139"[IS] OR "1383-5742"[IS] OR "0921-8890"[IS] OR "0921-8890"[IS] OR "1526-4602"[IS] OR "1525-7797"[IS] OR "2691-1299"[IS] OR "2691-1299"[IS] OR "2424-8630"[IS] OR "2424-8622"[IS] OR "2590-0986"[IS] OR "2590-0986"[IS] OR "1041-0236"[IS] OR "1532-7027"[IS] OR "1880-9952"[IS] OR "1346-4280"[IS] OR "1535-0665"[IS] OR "0097-8507"[IS] OR "2214-6350"[IS] OR "2214-6369"[IS] OR "1741-0398"[IS] OR "1741-0398"[IS] OR "2095-1779"[IS] OR "2095-1779"[IS] OR "0031-8116"[IS] OR "0031-8116"[IS] OR "2198-6576"[IS] OR "2198-6584"[IS] OR "1756-8765"[IS] OR "1756-8757"[IS] OR "1521-6942"[IS] OR "1532-1770"[IS] OR "0009-9163"[IS] OR "1399-0004"[IS] OR "1090-2643"[IS] OR "0019-1035"[IS] OR "1467-9817"[IS] OR "0141-0423"[IS] OR "0963-8695"[IS] OR "0963-8695"[IS] OR "1944-2866"[IS] OR "1944-2866"[IS] OR "0021-7670"[IS] OR "1565-8538"[IS] OR "1533-3884"[IS] OR "0731-5090"[IS] OR "0095-3997"[IS] OR "1552-3039"[IS] OR "1179-1926"[IS] OR "0312-5963"[IS] OR "0891-2017"[IS] OR "1530-9312"[IS] OR "1526-100X"[IS] OR "1061-2971"[IS] OR "0515-0361"[IS] OR "1783-1350"[IS] OR "1475-2875"[IS] OR "1475-2875"[IS] OR "1878-5506"[IS] OR "1389-9457"[IS] OR "1556-3669"[IS] OR "1530-5627"[IS] OR "1746-630X"[IS] OR "1746-630X"[IS] OR "2095-7564"[IS] OR "2095-7564"[IS] OR "1083-6489"[IS] OR "1083-6489"[IS] OR "2352-4316"[IS] OR "2352-4316"[IS] OR "1464-5343"[IS] OR "1369-1066"[IS] OR "1570-761X"[IS] OR "1570-761X"[IS] OR "2296-889X"[IS] OR "2296-889X"[IS] OR "2325-5889"[IS] OR "2325-5897"[IS] OR "2473-3938"[IS] OR "2473-4446"[IS] OR "1934-8584"[IS] OR "1934-8576"[IS] OR "1099-1166"[IS] OR "0885-6230"[IS] OR "2352-7102"[IS] OR "2352-7102"[IS] OR "1499-2752"[IS] OR

	"0315-162X"[IS] OR "0924-6495"[IS] OR "1572-8641"[IS] OR "0007-1315"[IS] OR "1468-4446"[IS] OR "1934-662X"[IS] OR "1934-6638"[IS] OR "1740-5025"[IS] OR "1470-7330"[IS] OR "1674-1137"[IS] OR "1674-1137"[IS] OR "0142-694X"[IS] OR "0142-694X"[IS] OR "1664-462X"[IS] OR "1664-462X"[IS] OR "0033-3506"[IS] OR "1476-5616"[IS] OR "0165-1684"[IS] OR "0165-1684"[IS] OR "1573-1391"[IS] OR "0924-1868"[IS] OR "0013-0427"[IS] OR "1468-0335"[IS] OR "1445-8330"[IS] OR "1440-0979"[IS] OR "1521-3765"[IS] OR "0947-6539"[IS] OR "0883-8151"[IS] OR "1550-6878"[IS] OR "2468-5194"[IS] OR "2468-5194"[IS] OR "2214-3912"[IS] OR "2214-3912"[IS] OR "1550-4859"[IS] OR "1550-4859"[IS] OR "0951-8339"[IS] OR "0951-8339"[IS] OR "1600-0412"[IS] OR "0001-6349"[IS] OR "0373-0956"[IS] OR "0373-0956"[IS] OR "1469-5820"[IS] OR "0144-3410"[IS] OR "2190-4707"[IS] OR "2190-4715"[IS] OR "2471-1403"[IS] OR "2471-1403"[IS] OR "2589-9155"[IS] OR "2589-9155"[IS] OR "0038-0806"[IS] OR "0038-0806"[IS] OR "1756-283X"[IS] OR "1756-2848"[IS] OR "1471-6828"[IS] OR "0004-8402"[IS] OR "1000-9361"[IS] OR "1000-9361"[IS] OR "1059-941X"[IS] OR "1532-849X"[IS] OR "0191-8141"[IS] OR "0191-8141"[IS] OR "1573-7802"[IS] OR "0892-7537"[IS] OR "1050-3307"[IS] OR "1468-4381"[IS] OR "2352-4677"[IS] OR "2352-4677"[IS] OR "0893-9659"[IS] OR "0893-9659"[IS] OR "1472-6890"[IS] OR "1472-6890"[IS] OR "1933-9747"[IS] OR "1933-9747"[IS] OR "1367-2630"[IS] OR "1367-2630"[IS] OR "2326-0254"[IS] OR "2326-0254"[IS] OR "0022-2518"[IS] OR "0022-2518"[IS] OR "1357-633X"[IS] OR "1758-1109"[IS] OR "0032-7786"[IS] OR "0032-7786"[IS] OR "1532-1991"[IS] OR "0143-4160"[IS] OR "0010-4825"[IS] OR "1879-0534"[IS] OR "1664-0640"[IS] OR "1664-0640"[IS] OR "1531-1309"[IS] OR "1531-1309"[IS] OR "1938-8926"[IS] OR "1938-8926"[IS] OR "1552-7557"[IS] OR "1049-7323"[IS] OR "1522-6417"[IS] OR "1534-3111"[IS] OR "2197-8670"[IS] OR "2197-8670"[IS] OR "1077-260X"[IS] OR "1558-4542"[IS] OR "1479-6813"[IS] OR "0952-5041"[IS] OR "1524-4040"[IS] OR "0148-396X"[IS] OR "1423-0003"[IS] OR "0304-324X"[IS] OR "1488-2434"[IS] OR "1180-4882"[IS] OR "1533-628X"[IS] OR "1533-628X"[IS] OR "1526-5161"[IS] OR "1536-0075"[IS] OR "0964-8410"[IS] OR "1467-8683"[IS] OR "1557-3079"[IS] OR "1557-3060"[IS] OR "1746-045X"[IS] OR "1746-0441"[IS] OR "0263-5577"[IS] OR "0263-5577"[IS] OR "1543-270X"[IS] OR "0888-4773"[IS] OR "1550-7289"[IS] OR "1550-7289"[IS] OR "0305-4403"[IS] OR "1095-9238"[IS] OR "1530-0307"[IS] OR "0023-6837"[IS] OR "0954-4224"[IS] OR "1475-2700"[IS] OR "1867-1748"[IS] OR "1867-1756"[IS] OR "1468-0629"[IS] OR "2164-7402"[IS] OR "0142-159X"[IS] OR "1466-187X"[IS] OR "1542-0086"[IS] OR "0006-3495"[IS] OR "1752-1386"[IS] OR "1752-1378"[IS] OR "1367-9430"[IS] OR "1469-1795"[IS] OR "0305-7364"[IS] OR "1095-8290"[IS] OR "0267-1905"[IS] OR "0267-1905"[IS] OR "1077-3142"[IS] OR "1090-235X"[IS] OR "2192-4384"[IS] OR "2192-4376"[IS] OR "1092-8987"[IS] OR "1092-8987"[IS] OR "0748-4518"[IS] OR "1573-7799"[IS] OR "1619-0033"[IS] OR "1314-2488"[IS] OR "1388-2457"[IS] OR "1872-8952"[IS] OR "0165-0114"[IS] OR "0165-0114"[IS] OR "1874-8597"[IS] OR "1874-8600"[IS] OR "0905-6947"[IS] OR "1600-0668"[IS] OR "2050-750X"[IS] OR "2050-7518"[IS] OR "1557-6051"[IS] OR "1533-5399"[IS] OR "1063-3995"[IS] OR "1099-0879"[IS] OR "2095-5421"[IS] OR "2214-5141"[IS] OR "1095-9971"[IS] OR "0195-6698"[IS] OR "1521-3978"[IS] OR "0015-8208"[IS] OR "0703-6337"[IS] OR "1477-2280"[IS] OR "1573-1618"[IS] OR "1385-2256"[IS] OR "2662-4753"[IS] OR "2662-4745"[IS] OR "2576-7666"[IS] OR "2576-7658"[IS] OR "1463-4996"[IS] OR "1463-4996"[IS] OR "1469-1841"[IS] OR "1366-7289"[IS] OR "1573-2894"[IS] OR "0926-6003"[IS] OR "0169-555X"[IS] OR "1872-695X"[IS] OR "1749-9518"[IS] OR "1749-9526"[IS] OR "2046-4924"[IS] OR "1366-5278"[IS] OR "0167-6105"[IS] OR "0167-6105"[IS] OR "1879-0631"[IS] OR "0024-3205"[IS] OR "1942-2962"[IS] OR "1942-2970"[IS] OR "0024-6107"[IS] OR "1469-7750"[IS] OR "1539-2864"[IS] OR "0275-004X"[IS] OR "0193-3973"[IS] OR "0193-3973"[IS] OR "1520-4812"[IS] OR "1043-1802"[IS] OR "2167-4809"[IS] OR "2167-4795"[IS] OR "2405-9595"[IS] OR "2405-9595"[IS] OR "1888-9891"[IS] OR "1989-4600"[IS] OR "1478-4491"[IS] OR "1478-4491"[IS] OR "1559-3169"[IS] OR "1096-7494"[IS] OR "0378-8733"[IS] OR "0378-8733"[IS] OR "2589-7217"[IS] OR "2589-7217"[IS] OR "2047-6302"[IS] OR "2047-6310"[IS] OR "2474-9567"[IS] OR "2474-9567"[IS] OR "1752-1505"[IS] OR "1752-1505"[IS] OR "1949-2723"[IS] OR "1949-2715"[IS] OR "1573-1677"[IS] OR "1382-4996"[IS] OR "1090-820X"[IS] OR "1527-330X"[IS] OR "2378-1823"[IS] OR "2378-1815"[IS] OR "2198-0926"[IS] OR "2198-0926"[IS] OR "0920-5489"[IS] OR "0920-5489"[IS] OR "1552-3861"[IS] OR "0011-0000"[IS] OR "0973-0826"[IS] OR "0973-0826"[IS] OR "2095-9885"[IS] OR "2468-0141"[IS] OR "1095-7227"[IS] OR "0147-5967"[IS] OR "1467-9787"[IS] OR "0022-4146"[IS] OR "1557-7392"[IS] OR "1049-331X"[IS] OR "0141-1187"[IS] OR "0141-1187"[IS] OR "2095-0462"[IS] OR "2095-0470"[IS] OR "2213-3437"[IS] OR "2213-3437"[IS] OR "0079-6611"[IS] OR "0079-6611"[IS] OR "1573-2649"[IS] OR "0962-9343"[IS] OR "1872-8243"[IS] OR "1386-5056"[IS] OR "0362-2436"[IS] OR "1528-1159"[IS] OR "1554-0170"[IS] OR "0268-1102"[IS] OR "1573-3432"[IS] OR "0162-3257"[IS] OR "1089-7690"[IS] OR "0021-9606"[IS] OR "1742-5689"[IS] OR "1742-5662"[IS] OR "1532-3005"[IS] OR "1532-2998"[IS] OR "1431-6730"[IS] OR "1437-4315"[IS] OR "1758-5996"[IS] OR "1758-5996"[IS] OR "1474-2837"[IS] OR "1474-2837"[IS] OR "1473-5598"[IS] OR "0263-6352"[IS] OR "2590-0617"[IS] OR "2590-0617"[IS] OR "2574-0970"[IS] OR "2574-0970"[IS] OR "2049-8772"[IS] OR "2049-8772"[IS] OR "0263-2764"[IS] OR "1460-3616"[IS] OR "0178-2770"[IS] OR "1432-0452"[IS] OR "0097-3165"[IS] OR "1096-0899"[IS] OR "1547-5646"[IS] OR

"1547-5654"[IS] OR "0308-5961"[IS] OR "0308-5961"[IS] OR "0040-747X"[IS] OR "1467-9663"[IS] OR
"1683-3511"[IS] OR "1683-3511"[IS] OR "2399-5300"[IS] OR "2399-5300"[IS] OR "0162-5748"[IS] OR
"1090-7009"[IS] OR "0953-2048"[IS] OR "1361-6668"[IS] OR "1521-6934"[IS] OR "1521-6934"[IS] OR
"0147-5916"[IS] OR "1573-2819"[IS] OR "0889-8529"[IS] OR "0889-8529"[IS] OR "0267-3231"[IS] OR
"1460-3705"[IS] OR "1664-302X"[IS] OR "1664-302X"[IS] OR "1424-3199"[IS] OR "1424-3202"[IS] OR
"1424-9294"[IS] OR "1424-9286"[IS] OR "0033-2720"[IS] OR "1573-6709"[IS] OR "0922-4777"[IS] OR
"1573-0905"[IS] OR "1098-9064"[IS] OR "0094-6176"[IS] OR "1934-5925"[IS] OR "1934-5925"[IS] OR
"0300-5712"[IS] OR "0300-5712"[IS] OR "2096-2754"[IS] OR "2467-9674"[IS] OR "0003-9888"[IS] OR
"1468-2044"[IS] OR "2045-7634"[IS] OR "2045-7634"[IS] OR "1867-3880"[IS] OR "1867-3899"[IS] OR
"1708-3087"[IS] OR "1708-3087"[IS] OR "1092-0684"[IS] OR "1092-0684"[IS] OR "1469-8188"[IS] OR
"0952-6757"[IS] OR "0195-6663"[IS] OR "1095-8304"[IS] OR "0169-4332"[IS] OR "0169-4332"[IS] OR
"0141-8130"[IS] OR "1879-0003"[IS] OR "1533-287X"[IS] OR "1533-2861"[IS] OR "1741-3850"[IS] OR
"1741-3842"[IS] OR "1573-1855"[IS] OR "1387-1579"[IS] OR "1557-7295"[IS] OR "0098-3500"[IS] OR
"1528-1132"[IS] OR "0009-921X"[IS] OR "1477-2566"[IS] OR "1465-3249"[IS] OR "1755-5345"[IS] OR
"1755-5345"[IS] OR "1502-2269"[IS] OR "1502-2250"[IS] OR "1463-5771"[IS] OR "1463-5771"[IS] OR
"0889-4906"[IS] OR "0889-4906"[IS] OR "1076-9986"[IS] OR "1935-1054"[IS] OR "1472-3263"[IS] OR
"1368-4973"[IS] OR "2212-0955"[IS] OR "2212-0955"[IS] OR "0378-1127"[IS] OR "0378-1127"[IS] OR
"1741-5659"[IS] OR "1758-8510"[IS] OR "0167-2681"[IS] OR "0167-2681"[IS] OR "2352-1791"[IS] OR
"2352-1791"[IS] OR "1568-7767"[IS] OR "1572-980X"[IS] OR "0304-4149"[IS] OR "0304-4149"[IS] OR
"1873-734X"[IS] OR "1010-7940"[IS] OR "1876-0341"[IS] OR "1876-035X"[IS] OR "2234-0726"[IS] OR
"2234-2451"[IS] OR "2332-8940"[IS] OR "2332-8959"[IS] OR "2590-2075"[IS] OR "2590-2075"[IS] OR
"1935-9748"[IS] OR "1935-9748"[IS] OR "0301-472X"[IS] OR "1873-2399"[IS] OR "2332-7790"[IS] OR
"2332-7790"[IS] OR "0950-5849"[IS] OR "0950-5849"[IS] OR "0219-1032"[IS] OR "1016-8478"[IS] OR
"1871-1014"[IS] OR "1871-1014"[IS] OR "2397-8570"[IS] OR "2397-8570"[IS] OR "2578-5745"[IS] OR
"2578-5745"[IS] OR "2631-7117"[IS] OR "0069-4827"[IS] OR "0098-3004"[IS] OR "0098-3004"[IS] OR
"0956-5221"[IS] OR "1873-3387"[IS] OR "1471-4973"[IS] OR "1471-4892"[IS] OR "1879-0135"[IS] OR
"0020-7519"[IS] OR "1525-3198"[IS] OR "0022-0302"[IS] OR "0022-0388"[IS] OR "1743-9140"[IS] OR
"0146-9592"[IS] OR "1539-4794"[IS] OR "1946-5238"[IS] OR "1946-5238"[IS] OR "1532-9496"[IS] OR
"0887-7963"[IS] OR "1938-9663"[IS] OR "1938-9655"[IS] OR "1532-7051"[IS] OR "0737-0024"[IS] OR
"1097-0088"[IS] OR "0899-8418"[IS] OR "1573-7373"[IS] OR "0167-594X"[IS] OR "0721-7714"[IS] OR
"1432-203X"[IS] OR "1559-1131"[IS] OR "1559-1131"[IS] OR "0197-9337"[IS] OR "0197-9337"[IS] OR
"0013-0079"[IS] OR "1539-2988"[IS] OR "1875-4805"[IS] OR "1875-4791"[IS] OR "1177-5491"[IS] OR
"1177-5475"[IS] OR "0950-222X"[IS] OR "1476-5454"[IS] OR "2527-8045"[IS] OR "2528-1410"[IS] OR
"2198-7823"[IS] OR "2095-8293"[IS] OR "2197-7364"[IS] OR "2197-7364"[IS] OR "1477-4526"[IS] OR
"0951-0893"[IS] OR "1468-0017"[IS] OR "0268-1064"[IS] OR "2379-8920"[IS] OR "2379-8939"[IS] OR
"0027-9684"[IS] OR "1943-4693"[IS] OR "2468-0249"[IS] OR "2468-0249"[IS] OR "1432-1750"[IS] OR
"0341-2040"[IS] OR "1096-1127"[IS] OR "0023-6438"[IS] OR "0304-422X"[IS] OR "0304-422X"[IS] OR
"0958-3440"[IS] OR "0958-3440"[IS] OR "2194-8011"[IS] OR "2194-802X"[IS] OR "2041-9139"[IS] OR
"2041-9139"[IS] OR "1520-4383"[IS] OR "1520-4391"[IS] OR "1070-9932"[IS] OR "1070-9932"[IS] OR
"2561-7605"[IS] OR "2561-7605"[IS] OR "0169-1368"[IS] OR "0169-1368"[IS] OR "2047-4849"[IS] OR
"2047-4830"[IS] OR "1996-8744"[IS] OR "1996-3599"[IS] OR "0934-9723"[IS] OR "1435-4373"[IS] OR
"2199-4730"[IS] OR "2199-4730"[IS] OR "2666-5212"[IS] OR "2666-5212"[IS] OR "0022-4294"[IS] OR
"1945-0095"[IS] OR "0885-7474"[IS] OR "1573-7691"[IS] OR "1939-1676"[IS] OR "0891-6640"[IS] OR
"0033-8362"[IS] OR "1826-6983"[IS] OR "1049-4820"[IS] OR "1049-4820"[IS] OR "0306-686X"[IS] OR
"1468-5957"[IS] OR "1552-7395"[IS] OR "0899-7640"[IS] OR "0162-2439"[IS] OR "1552-8251"[IS] OR
"2474-7394"[IS] OR "2474-7394"[IS] OR "1529-7322"[IS] OR "1534-6315"[IS] OR "0941-0643"[IS] OR
"1433-3058"[IS] OR "1728-4457"[IS] OR "0098-7921"[IS] OR "1468-6996"[IS] OR "1468-6996"[IS] OR
"1017-0405"[IS] OR "1017-0405"[IS] OR "1863-2653"[IS] OR "1863-2661"[IS] OR "1875-6190"[IS] OR
"1570-159X"[IS] OR "1740-357X"[IS] OR "1740-357X"[IS] OR "0944-5013"[IS] OR "1618-0623"[IS] OR
"0195-6108"[IS] OR "0195-6108"[IS] OR "0003-3219"[IS] OR "0003-3219"[IS] OR "0927-6505"[IS] OR
"0927-6505"[IS] OR "0018-9294"[IS] OR "1558-2531"[IS] OR "0047-259X"[IS] OR "1095-7243"[IS] OR
"1044-5005"[IS] OR "1096-1224"[IS] OR "1354-0602"[IS] OR "1470-1278"[IS] OR "1940-0632"[IS] OR
"1940-0640"[IS] OR "2193-8229"[IS] OR "2193-6382"[IS] OR "1939-1285"[IS] OR "0278-7393"[IS] OR
"0031-3955"[IS] OR "1557-8240"[IS] OR "1573-7527"[IS] OR "0929-5593"[IS] OR "0022-1856"[IS] OR
"1472-9296"[IS] OR "0929-1393"[IS] OR "0929-1393"[IS] OR "2468-2330"[IS] OR "2468-2330"[IS] OR
"2160-6064"[IS] OR "2160-6056"[IS] OR "1976-3786"[IS] OR "0253-6269"[IS] OR "1532-3641"[IS] OR
"1943-5622"[IS] OR "2509-498X"[IS] OR "2509-498X"[IS] OR "1002-0160"[IS] OR "1002-0160"[IS] OR
"1432-0738"[IS] OR "0340-5761"[IS] OR "0144-6657"[IS] OR "2044-8260"[IS] OR "2212-2672"[IS] OR
"2212-2672"[IS] OR "0950-3293"[IS] OR "0950-3293"[IS] OR "0260-8774"[IS] OR "0260-8774"[IS] OR
"1364-6915"[IS] OR "1360-7863"[IS] OR "1058-0530"[IS] OR "1058-0530"[IS] OR "0012-0456"[IS] OR

	"0012-0456"[IS] OR "0378-5122"[IS] OR "0378-5122"[IS] OR "1573-1898"[IS] OR "0157-244X"[IS] OR "2352-7218"[IS] OR "2352-7218"[IS] OR "1532-7361"[IS] OR "0039-6060"[IS] OR "1613-9860"[IS] OR "1869-6716"[IS] OR "1941-7330"[IS] OR "1932-6157"[IS] OR "1471-2229"[IS] OR "1471-2229"[IS] OR "1467-0895"[IS] OR "1467-0895"[IS] OR "0016-0032"[IS] OR "0016-0032"[IS] OR "1424-3911"[IS] OR "1424-3903"[IS] OR "2212-0122"[IS] OR "2212-0122"[IS] OR "2196-4092"[IS] OR "2196-4092"[IS] OR "1040-7294"[IS] OR "1040-7294"[IS] OR "0743-7315"[IS] OR "1096-0848"[IS] OR "2662-1746"[IS] OR "2096-6490"[IS] OR "1873-1732"[IS] OR "0079-6107"[IS] OR "2662-2289"[IS] OR "2662-2297"[IS] OR "0955-470X"[IS] OR "0955-470X"[IS] OR "1007-9327"[IS] OR "1007-9327"[IS] OR "2352-1864"[IS] OR "2352-1864"[IS] OR "1349-9092"[IS] OR "0917-5040"[IS] OR "0084-0173"[IS] OR "0084-0173"[IS] OR "1464-3545"[IS] OR "0309-166X"[IS] OR "1552-6879"[IS] OR "0021-8863"[IS] OR "1096-0317"[IS] OR "0049-089X"[IS] OR "0003-0090"[IS] OR "0003-0090"[IS] OR "1997-003X"[IS] OR "1994-2060"[IS] OR "2322-5939"[IS] OR "2322-5939"[IS] OR "0140-7007"[IS] OR "0140-7007"[IS] OR "1422-0067"[IS] OR "1661-6596"[IS] OR "0022-3913"[IS] OR "1097-6841"[IS] OR "1478-9299"[IS] OR "1478-9302"[IS] OR "0939-3889"[IS] OR "0939-3889"[IS] OR "2373-8227"[IS] OR "2373-8227"[IS] OR "0015-718X"[IS] OR "0015-718X"[IS] OR "1932-751X"[IS] OR "1932-7501"[IS] OR "1469-7742"[IS] OR "0022-2267"[IS] OR "1573-5036"[IS] OR "0032-079X"[IS] OR "0166-445X"[IS] OR "1879-1514"[IS] OR "1573-2908"[IS] OR "1568-4539"[IS] OR "0018-9340"[IS] OR "0018-9340"[IS] OR "0733-9364"[IS] OR "0733-9364"[IS] OR "1871-1871"[IS] OR "1871-1871"[IS] OR "2573-1599"[IS] OR "2573-1602"[IS] OR "2332-2950"[IS] OR "2332-2969"[IS] OR "1208-6053"[IS] OR "1181-8700"[IS] OR "1740-8989"[IS] OR "1742-5786"[IS] OR "2378-9689"[IS] OR "2378-9697"[IS] OR "1936-5233"[IS] OR "1936-5233"[IS] OR "2474-9842"[IS] OR "2474-9842"[IS] OR "1573-6792"[IS] OR "0896-0267"[IS] OR "2367-1726"[IS] OR "2367-1734"[IS] OR "1069-4730"[IS] OR "2168-9830"[IS] OR "1470-7926"[IS] OR "1351-0711"[IS] OR "2470-5470"[IS] OR "2470-5470"[IS] OR "2190-4936"[IS] OR "1864-6956"[IS] OR "0888-4064"[IS] OR "1944-4931"[IS] OR "0010-4159"[IS] OR "2151-6227"[IS] OR "1469-8749"[IS] OR "0012-1622"[IS] OR "1478-6729"[IS] OR "0193-936X"[IS] OR "1461-7129"[IS] OR "0959-6801"[IS] OR "1530-9304"[IS] OR "1063-6560"[IS] OR "2374-4235"[IS] OR "2374-4243"[IS] OR "1556-8334"[IS] OR "1556-8318"[IS] OR "1387-2877"[IS] OR "1875-8908"[IS] OR "1472-698X"[IS] OR "1472-698X"[IS] OR "1478-5242"[IS] OR "0960-1643"[IS] OR "1475-2867"[IS] OR "1475-2867"[IS] OR "1351-8216"[IS] OR "1365-2516"[IS] OR "2572-9241"[IS] OR "2572-9241"[IS] OR "0165-2370"[IS] OR "0165-2370"[IS] OR "1099-145X"[IS] OR "1085-3278"[IS] OR "0269-5022"[IS] OR "1365-3016"[IS] OR "1040-1334"[IS] OR "1532-8015"[IS] OR "2519-173X"[IS] OR "2519-173X"[IS] OR "2214-7144"[IS] OR "2214-7144"[IS] OR "1086-055X"[IS] OR "1086-055X"[IS] OR "0223-5234"[IS] OR "1768-3254"[IS] OR "1662-5129"[IS] OR "1662-5129"[IS] OR "2197-4284"[IS] OR "2197-4284"[IS] OR "1480-9222"[IS] OR "1480-9222"[IS] OR "1460-1060"[IS] OR "1460-1060"[IS] OR "1476-072X"[IS] OR "1476-072X"[IS] OR "1916-0216"[IS] OR "1916-0208"[IS] OR "1550-2724"[IS] OR "1550-2716"[IS] OR "0167-8442"[IS] OR "0167-8442"[IS] OR "1534-7656"[IS] OR "1085-9373"[IS] OR "1613-7027"[IS] OR "1613-7027"[IS] OR "2042-8898"[IS] OR "2042-8901"[IS] OR "1573-3521"[IS] OR "0160-7715"[IS] OR "1573-3750"[IS] OR "1573-3750"[IS] OR "1466-447X"[IS] OR "0264-0414"[IS] OR "1028-3153"[IS] OR "1028-3153"[IS] OR "0031-9317"[IS] OR "1399-3054"[IS] OR "0981-9428"[IS] OR "1873-2690"[IS] OR "0165-2176"[IS] OR "1875-5941"[IS] OR "0892-3647"[IS] OR "1538-9286"[IS] OR "1573-3327"[IS] OR "0009-398X"[IS] OR "1939-1382"[IS] OR "1939-1382"[IS] OR "1178-1653"[IS] OR "1178-1661"[IS] OR "0039-3606"[IS] OR "1936-6167"[IS] OR "1530-261X"[IS] OR "1530-261X"[IS] OR "0951-6433"[IS] OR "1872-8081"[IS] OR "0143-6228"[IS] OR "0143-6228"[IS] OR "2199-4536"[IS] OR "2198-6053"[IS] OR "1752-4458"[IS] OR "1752-4458"[IS] OR "1063-8539"[IS] OR "1063-8539"[IS] OR "1933-0693"[IS] OR "0022-3085"[IS] OR "1746-0220"[IS] OR "1743-9671"[IS] OR "1432-8232"[IS] OR "0025-5874"[IS] OR "1094-4087"[IS] OR "1094-4087"[IS] OR "0924-1884"[IS] OR "0924-1884"[IS] OR "1475-3081"[IS] OR "1471-0684"[IS] OR "1471-2407"[IS] OR "1471-2407"[IS] OR "1938-0690"[IS] OR "1525-7304"[IS] OR "1835-8535"[IS] OR "1324-9347"[IS] OR "1479-9723"[IS] OR "1479-9731"[IS] OR "0272-5231"[IS] OR "1557-8216"[IS] OR "0955-2863"[IS] OR "1873-4847"[IS] OR "1475-2891"[IS] OR "1475-2891"[IS] OR "2072-4292"[IS] OR "2072-4292"[IS] OR "1876-2859"[IS] OR "1876-2867"[IS] OR "1872-7441"[IS] OR "0165-232X"[IS] OR "1161-0301"[IS] OR "1161-0301"[IS] OR "0948-3349"[IS] OR "1614-7502"[IS] OR "1741-2560"[IS] OR "1741-2560"[IS] OR "1356-7667"[IS] OR "1479-1870"[IS] OR "1520-4995"[IS] OR "0006-2960"[IS] OR "2090-1321"[IS] OR "2090-133X"[IS] OR "1554-0642"[IS] OR "1554-0650"[IS] OR "1525-1438"[IS] OR "1048-891X"[IS] OR "0165-1889"[IS] OR "0165-1889"[IS] OR "1743-0003"[IS] OR "1743-0003"[IS] OR "1421-9670"[IS] OR "0250-8095"[IS] OR "1068-9265"[IS] OR "1534-4681"[IS] OR "1612-9067"[IS] OR "1612-9059"[IS] OR "1752-9727"[IS] OR "1752-9719"[IS] OR "0002-9149"[IS] OR "1879-1913"[IS] OR "1327-2020"[IS] OR "0312-8962"[IS] OR "1040-8738"[IS] OR "1531-7021"[IS] OR "2212-4209"[IS] OR "2212-4209"[IS] OR "2641-4368"[IS] OR "2641-4368"[IS] OR "1573-5028"[IS] OR "0167-4412"[IS] OR "2010-1406"[IS] OR "2010-1392"[IS] OR "2633-0679"[IS] OR "2633-0679"[IS] OR "1432-5233"[IS] OR "0940-5429"[IS] OR "1879-1360"[IS] OR "0022-3999"[IS] OR "0038-0121"[IS] OR "0038-0121"[IS] OR "1368-0005"[IS] OR "1467-9612"[IS] OR "1553-5231"[IS] OR

	"1078-0947"[IS] OR "2689-5269"[IS] OR "2689-5277"[IS] OR "2468-2284"[IS] OR "2468-2179"[IS] OR "2380-193X"[IS] OR "2380-193X"[IS] OR "2577-6207"[IS] OR "2577-6207"[IS] OR "0098-8472"[IS] OR "0098-8472"[IS] OR "0894-6507"[IS] OR "1558-2345"[IS] OR "2472-5862"[IS] OR "2472-5854"[IS] OR "1470-1243"[IS] OR "1357-3322"[IS] OR "1755-375X"[IS] OR "1755-3768"[IS] OR "1177-9322"[IS] OR "1177-9322"[IS] OR "0883-7694"[IS] OR "1938-1425"[IS] OR "1545-6943"[IS] OR "0097-9740"[IS] OR "1552-8278"[IS] OR "1046-4964"[IS] OR "1464-0708"[IS] OR "1354-6783"[IS] OR "2055-0936"[IS] OR "2055-0936"[IS] OR "1078-0874"[IS] OR "1078-0874"[IS] OR "1471-2318"[IS] OR "1471-2318"[IS] OR "8756-3282"[IS] OR "8756-3282"[IS] OR "1639-4488"[IS] OR "1639-4488"[IS] OR "1178-6469"[IS] OR "1178-6469"[IS] OR "0165-0157"[IS] OR "0165-0157"[IS] OR "1662-4025"[IS] OR "1662-4033"[IS] OR "0960-8923"[IS] OR "1708-0428"[IS] OR "0031-6768"[IS] OR "1432-2013"[IS] OR "1388-2481"[IS] OR "1388-2481"[IS] OR "1749-4613"[IS] OR "1749-4613"[IS] OR "2667-3053"[IS] OR "2667-3053"[IS] OR "2523-8906"[IS] OR "2523-8906"[IS] OR "0164-1212"[IS] OR "0164-1212"[IS] OR "0964-5691"[IS] OR "0964-5691"[IS] OR "0944-7113"[IS] OR "1618-095X"[IS] OR "1940-6207"[IS] OR "1940-6215"[IS] OR "0262-0898"[IS] OR "1573-7276"[IS] OR "1538-7216"[IS] OR "1538-7216"[IS] OR "2296-2565"[IS] OR "2296-2565"[IS] OR "1541-6100"[IS] OR "0022-3166"[IS] OR "1094-3501"[IS] OR "1094-3501"[IS] OR "1389-9341"[IS] OR "1389-9341"[IS] OR "2211-3681"[IS] OR "2211-3681"[IS] OR "1745-0101"[IS] OR "1745-011X"[IS] OR "1432-2218"[IS] OR "0930-2794"[IS] OR "1070-9908"[IS] OR "1558-2361"[IS] OR "1938-3703"[IS] OR "0021-8855"[IS] OR "0146-8693"[IS] OR "1465-735X"[IS] OR "2194-7961"[IS] OR "2194-7953"[IS] OR "0967-0106"[IS] OR "0967-0106"[IS] OR "2352-8532"[IS] OR "2352-8532"[IS] OR "1469-5871"[IS] OR "1350-4622"[IS] OR "2296-7745"[IS] OR "2296-7745"[IS] OR "1874-1754"[IS] OR "0167-5273"[IS] OR "2041-479X"[IS] OR "0016-7649"[IS] OR "1049-3867"[IS] OR "1878-4321"[IS] OR "2053-1400"[IS] OR "2053-1419"[IS] OR "1757-1693"[IS] OR "1757-1707"[IS] OR "1746-4811"[IS] OR "1746-4811"[IS] OR "2405-6383"[IS] OR "2405-6545"[IS] OR "1471-2598"[IS] OR "1744-7682"[IS] OR "2473-1242"[IS] OR "2473-1242"[IS] OR "1367-5567"[IS] OR "1469-848X"[IS] OR "1461-7285"[IS] OR "0269-8811"[IS] OR "1750-1172"[IS] OR "1750-1172"[IS] OR "2324-9943"[IS] OR "2324-9935"[IS] OR "2163-8306"[IS] OR "2163-8306"[IS] OR "2328-8620"[IS] OR "2328-8620"[IS] OR "1552-6887"[IS] OR "0898-2643"[IS] OR "1386-4416"[IS] OR "1386-4416"[IS] OR "2053-051X"[IS] OR "0004-6264"[IS] OR "1527-3296"[IS] OR "0196-6553"[IS] OR "0169-2607"[IS] OR "1872-7565"[IS] OR "2096-0042"[IS] OR "2096-0042"[IS] OR "1534-6277"[IS] OR "1527-2729"[IS] OR "2637-6431"[IS] OR "2637-6431"[IS] OR "0923-1811"[IS] OR "0923-1811"[IS] OR "1874-9968"[IS] OR "0041-624X"[IS] OR "1550-3585"[IS] OR "1550-3585"[IS] OR "1745-6150"[IS] OR "1745-6150"[IS] OR "2162-0555"[IS] OR "2162-0563"[IS] OR "2211-4645"[IS] OR "2211-4645"[IS] OR "2167-9223"[IS] OR "2167-8421"[IS] OR "1875-5100"[IS] OR "1875-5100"[IS] OR "1615-1488"[IS] OR "1615-147X"[IS] OR "2468-6557"[IS] OR "2468-2322"[IS] OR "0266-5611"[IS] OR "0266-5611"[IS] OR "1937-1888"[IS] OR "1938-4114"[IS] OR "2044-4052"[IS] OR "2044-4052"[IS] OR "1799-7267"[IS] OR "1457-4969"[IS] OR "2516-5542"[IS] OR "2516-5542"[IS] OR "1022-6877"[IS] OR "1421-9891"[IS] OR "1545-1550"[IS] OR "1526-6028"[IS] OR "1465-3931"[IS] OR "0031-3025"[IS] OR "0945-6317"[IS] OR "1432-2307"[IS] OR "1434-3916"[IS] OR "0936-8051"[IS] OR "2325-9671"[IS] OR "2325-9671"[IS] OR "1615-9861"[IS] OR "1615-9853"[IS] OR "1949-0461"[IS] OR "1084-1806"[IS] OR "0738-8942"[IS] OR "1549-9219"[IS] OR "2473-4039"[IS] OR "2473-4039"[IS] OR "0927-538X"[IS] OR "0927-538X"[IS] OR "1674-7348"[IS] OR "1674-7348"[IS] OR "1467-954X"[IS] OR "0038-0261"[IS] OR "1178-2013"[IS] OR "1176-9114"[IS] OR "1473-4257"[IS] OR "0306-6800"[IS] OR "0920-4105"[IS] OR "0920-4105"[IS] OR "1057-9249"[IS] OR "1099-1611"[IS] OR "1866-7910"[IS] OR "1866-7929"[IS] OR "1560-4292"[IS] OR "1560-4306"[IS] OR "2163-0755"[IS] OR "2163-0763"[IS] OR "1556-0961"[IS] OR "1541-6933"[IS] OR "2469-9934"[IS] OR "2469-9926"[IS] OR "2661-8036"[IS] OR "2661-8028"[IS] OR "1552-4841"[IS] OR "1552-485X"[IS] OR "1365-7305"[IS] OR "1365-7313"[IS] OR "1569-1721"[IS] OR "1573-8701"[IS] OR "0022-4375"[IS] OR "0022-4375"[IS] OR "1044-5803"[IS] OR "1044-5803"[IS] OR "1530-9576"[IS] OR "1530-9576"[IS] OR "2542-5285"[IS] OR "2542-5277"[IS] OR "2211-3398"[IS] OR "2211-3398"[IS] OR "0951-7375"[IS] OR "1473-6527"[IS] OR "1095-9947"[IS] OR "1050-4648"[IS] OR "0096-1523"[IS] OR "1939-1277"[IS] OR "1558-8017"[IS] OR "0198-9073"[IS] OR "1751-7915"[IS] OR "1751-7907"[IS] OR "2211-1662"[IS] OR "2211-1670"[IS] OR "1939-0025"[IS] OR "0002-9432"[IS] OR "1471-2164"[IS] OR "1471-2164"[IS] OR "0377-0273"[IS] OR "0377-0273"[IS] OR "1073-2780"[IS] OR "1073-2780"[IS] OR "2522-0098"[IS] OR "2522-0098"[IS] OR "1750-0680"[IS] OR "1750-0680"[IS] OR "0965-1748"[IS] OR "1879-0240"[IS] OR "1752-4598"[IS] OR "1752-458X"[IS] OR "0263-2241"[IS] OR "0263-2241"[IS] OR "2376-7839"[IS] OR "2376-7839"[IS] OR "1747-9991"[IS] OR "1747-9991"[IS] OR "0114-5916"[IS] OR "1179-1942"[IS] OR "2333-9403"[IS] OR "2333-9403"[IS] OR "0020-7721"[IS] OR "1464-5319"[IS] OR "2590-2601"[IS] OR "2590-2601"[IS] OR "2475-9953"[IS] OR "2475-9953"[IS] OR "0033-3174"[IS] OR "1534-7796"[IS] OR "1449-5554"[IS] OR "1449-5554"[IS] OR "2405-6308"[IS] OR "2405-6308"[IS] OR "1873-6173"[IS] OR "1566-0141"[IS] OR "1463-5003"[IS] OR "1463-5011"[IS] OR "2150-5594"[IS] OR "2150-5608"[IS] OR "1098-2337"[IS] OR "0096-140X"[IS] OR "1600-9657"[IS] OR "1600-4469"[IS] OR "1356-336X"[IS] OR "1741-2749"[IS] OR "2042-6496"[IS] OR

	"2042-650X"[IS] OR "1570-7873"[IS] OR "1570-7873"[IS] OR "1531-4995"[IS] OR "0023-852X"[IS] OR "0267-1522"[IS] OR "1470-1146"[IS] OR "2214-5095"[IS] OR "2214-5095"[IS] OR "0965-8416"[IS] OR "0965-8416"[IS] OR "1573-7810"[IS] OR "0199-0039"[IS] OR "1759-720X"[IS] OR "1759-7218"[IS] OR "0749-0720"[IS] OR "1558-4240"[IS] OR "2637-6113"[IS] OR "2637-6113"[IS] OR "1178-7023"[IS] OR "1178-7023"[IS] OR "2196-0763"[IS] OR "2196-0763"[IS] OR "0021-9193"[IS] OR "1098-5530"[IS] OR "1547-2450"[IS] OR "1547-2442"[IS] OR "2311-2638"[IS] OR "2311-2638"[IS] OR "0928-7655"[IS] OR "0928-7655"[IS] OR "1471-2105"[IS] OR "1471-2105"[IS] OR "1878-1705"[IS] OR "1567-5769"[IS] OR "1944-7477"[IS] OR "0028-0283"[IS] OR "1525-3171"[IS] OR "0032-5791"[IS] OR "1465-3397"[IS] OR "0013-1911"[IS] OR "0378-7796"[IS] OR "0378-7796"[IS] OR "1520-5118"[IS] OR "0021-8561"[IS] OR "1080-2371"[IS] OR "1538-6899"[IS] OR "0313-5926"[IS] OR "0313-5926"[IS] OR "1740-4762"[IS] OR "1740-4754"[IS] OR "1661-7800"[IS] OR "1661-7800"[IS] OR "2372-952X"[IS] OR "2372-952X"[IS] OR "1532-2157"[IS] OR "0748-7983"[IS] OR "1066-033X"[IS] OR "1066-033X"[IS] OR "0031-0182"[IS] OR "0031-0182"[IS] OR "0214-9915"[IS] OR "1886-144X"[IS] OR "0034-5318"[IS] OR "0034-5318"[IS] OR "2666-6790"[IS] OR "2666-6790"[IS] OR "1939-4551"[IS] OR "1939-4551"[IS] OR "2666-9579"[IS] OR "2666-9579"[IS] OR "2291-0026"[IS] OR "2291-0026"[IS] OR "1435-5663"[IS] OR "0177-0667"[IS] OR "2804-7214"[IS] OR "2822-7840"[IS] OR "1538-4632"[IS] OR "0016-7363"[IS] OR "1939-7089"[IS] OR "1548-0518"[IS] OR "2444-8834"[IS] OR "2444-8834"[IS] OR "2296-701X"[IS] OR "2296-701X"[IS] OR "1468-5965"[IS] OR "0021-9886"[IS] OR "2165-0497"[IS] OR "2165-0497"[IS] OR "1538-4756"[IS] OR "0741-9325"[IS] OR "1075-2935"[IS] OR "1075-2935"[IS] OR "1618-0089"[IS] OR "1439-1791"[IS] OR "1600-0609"[IS] OR "0902-4441"[IS] OR "1436-6215"[IS] OR "1436-6207"[IS] OR "1684-9981"[IS] OR "1561-8633"[IS] OR "2197-5620"[IS] OR "2095-6355"[IS] OR "0363-5023"[IS] OR "1531-6564"[IS] OR "1740-8695"[IS] OR "1740-8709"[IS] OR "1742-4755"[IS] OR "1742-4755"[IS] OR "1746-1391"[IS] OR "1746-1391"[IS] OR "0888-7985"[IS] OR "1558-7959"[IS] OR "1537-2723"[IS] OR "0040-1706"[IS] OR "1753-4658"[IS] OR "1753-4666"[IS] OR "2044-0375"[IS] OR "1354-8166"[IS] OR "1743-422X"[IS] OR "1743-422X"[IS] OR "0018-506X"[IS] OR "1095-6867"[IS] OR "0265-4075"[IS] OR "0265-4075"[IS] OR "1475-2859"[IS] OR "1475-2859"[IS] OR "1757-7241"[IS] OR "1757-7241"[IS] OR "1946-6315"[IS] OR "1946-6315"[IS] OR "1479-1803"[IS] OR "1350-231X"[IS] OR "1009-637X"[IS] OR "1009-637X"[IS] OR "2041-2649"[IS] OR "2041-2657"[IS] OR "1724-4935"[IS] OR "1724-4935"[IS] OR "2514-8486"[IS] OR "2514-8494"[IS] OR "2327-3801"[IS] OR "2327-3798"[IS] OR "1552-3381"[IS] OR "0002-7642"[IS] OR "1476-0711"[IS] OR "1476-0711"[IS] OR "2045-0915"[IS] OR "2045-0915"[IS] OR "2689-1808"[IS] OR "2689-1808"[IS] OR "0739-456X"[IS] OR "1552-6577"[IS] OR "1532-7965"[IS] OR "1047-840X"[IS] OR "1469-4409"[IS] OR "0950-2688"[IS] OR "0963-8237"[IS] OR "1360-0567"[IS] OR "0022-1430"[IS] OR "0022-1430"[IS] OR "0928-4931"[IS] OR "1873-0191"[IS] OR "2055-1045"[IS] OR "2055-1045"[IS] OR "0009-8981"[IS] OR "1873-3492"[IS] OR "1530-7026"[IS] OR "1531-135X"[IS] OR "1934-2616"[IS] OR "1934-2500"[IS] OR "1542-7951"[IS] OR "1944-9488"[IS] OR "1557-8615"[IS] OR "0883-9441"[IS] OR "2214-5818"[IS] OR "2214-5818"[IS] OR "0378-2166"[IS] OR "0378-2166"[IS] OR "1021-9722"[IS] OR "1420-9004"[IS] OR "0036-021X"[IS] OR "1468-4837"[IS] OR "2076-3921"[IS] OR "2076-3921"[IS] OR "2590-1621"[IS] OR "2590-1621"[IS] OR "1752-8062"[IS] OR "1752-8054"[IS] OR "1072-5369"[IS] OR "1072-5369"[IS] OR "2516-1091"[IS] OR "2516-1091"[IS] OR "1532-0480"[IS] OR "1532-0464"[IS] OR "2399-9802"[IS] OR "2399-9802"[IS] OR "1529-9740"[IS] OR "1529-9732"[IS] OR "2590-0285"[IS] OR "2590-0285"[IS] OR "1070-6631"[IS] OR "1089-7666"[IS] OR "0044-8486"[IS] OR "0044-8486"[IS] OR "0997-7538"[IS] OR "0997-7538"[IS] OR "1815-4654"[IS] OR "1815-7556"[IS] OR "0022-0965"[IS] OR "1096-0457"[IS] OR "1179-3201"[IS] OR "1179-3201"[IS] OR "0031-9201"[IS] OR "0031-9201"[IS] OR "1531-2267"[IS] OR "1094-8341"[IS] OR "1544-8444"[IS] OR "1544-8444"[IS] OR "1423-0356"[IS] OR "0025-7931"[IS] OR "0722-4028"[IS] OR "1432-0975"[IS] OR "1477-2574"[IS] OR "1365-182X"[IS] OR "0732-3123"[IS] OR "0732-3123"[IS] OR "1877-0568"[IS] OR "1877-0568"[IS] OR "1359-4338"[IS] OR "1359-4338"[IS] OR "0307-904X"[IS] OR "0307-904X"[IS] OR "2352-801X"[IS] OR "2352-801X"[IS] OR "0959-3845"[IS] OR "0959-3845"[IS] OR "1574-1192"[IS] OR "1574-1192"[IS] OR "2048-8505"[IS] OR "2048-8513"[IS] OR "1741-1440"[IS] OR "1741-1432"[IS] OR "1869-4101"[IS] OR "1869-4101"[IS] OR "0925-8388"[IS] OR "0925-8388"[IS] OR "2589-2347"[IS] OR "2589-2347"[IS] OR "0028-3835"[IS] OR "1423-0194"[IS] OR "1029-4937"[IS] OR "1055-6788"[IS] OR "2162-1934"[IS] OR "2162-1918"[IS] OR "1976-670X"[IS] OR "1976-6696"[IS] OR "0048-3486"[IS] OR "0048-3486"[IS] OR "1471-0366"[IS] OR "1471-0358"[IS] OR "2214-031X"[IS] OR "2214-031X"[IS] OR "0044-0094"[IS] OR "0044-0094"[IS] OR "0741-238X"[IS] OR "1865-8652"[IS] OR "0145-6008"[IS] OR "1530-0277"[IS] OR "0141-9870"[IS] OR "1466-4356"[IS] OR "1521-9488"[IS] OR "1468-2486"[IS] OR "1465-7333"[IS] OR "0022-1503"[IS] OR "1522-6379"[IS] OR "1522-6379"[IS] OR "0304-4203"[IS] OR "0304-4203"[IS] OR "1433-8319"[IS] OR "1433-8319"[IS] OR "1423-0054"[IS] OR "0018-716X"[IS] OR "1399-3038"[IS] OR "0905-6157"[IS] OR "2218-273X"[IS] OR "2218-273X"[IS] OR "0208-5216"[IS] OR "0208-5216"[IS] OR "0954-3007"[IS] OR "1476-5640"[IS] OR "1099-1573"[IS] OR "0951-418X"[IS] OR "0963-8180"[IS] OR "1468-4497"[IS] OR "0972-2696"[IS] OR "0972-2696"[IS] OR "2152-2812"[IS] OR "2194-5888"[IS] OR "1469-9508"[IS] OR

	"1360-080X"[IS] OR "0094-2405"[IS] OR "0094-2405"[IS] OR "0095-3628"[IS] OR "1432-184X"[IS] OR "1746-8108"[IS] OR "1746-8094"[IS] OR "0143-3334"[IS] OR "1460-2180"[IS] OR "1052-5173"[IS] OR "1052-5173"[IS] OR "2211-8845"[IS] OR "2211-8837"[IS] OR "2212-8689"[IS] OR "2212-8689"[IS] OR "2508-6235"[IS] OR "2508-7576"[IS] OR "1673-7067"[IS] OR "1673-7067"[IS] OR "2192-1709"[IS] OR "2192-1709"[IS] OR "1090-3801"[IS] OR "1532-2149"[IS] OR "1936-6426"[IS] OR "1936-6434"[IS] OR "1756-5391"[IS] OR "1756-5383"[IS] OR "1087-2981"[IS] OR "1087-2981"[IS] OR "1556-1631"[IS] OR "1556-1623"[IS] OR "1469-9044"[IS] OR "0260-2105"[IS] OR "0037-1963"[IS] OR "1532-8686"[IS] OR "2083-5205"[IS] OR "2084-1965"[IS] OR "2694-2461"[IS] OR "2694-2461"[IS] OR "0893-3200"[IS] OR "1939-1293"[IS] OR "0167-5133"[IS] OR "1477-4593"[IS] OR "1535-1084"[IS] OR "1559-1174"[IS] OR "1099-1492"[IS] OR "0952-3480"[IS] OR "1876-1631"[IS] OR "1876-1623"[IS] OR "1097-9891"[IS] OR "0095-2990"[IS] OR "2047-2927"[IS] OR "2047-2919"[IS] OR "1389-0417"[IS] OR "1389-0417"[IS] OR "1544-2241"[IS] OR "1544-1873"[IS] OR "2150-8925"[IS] OR "2150-8925"[IS] OR "1935-7524"[IS] OR "1935-7524"[IS] OR "1613-9372"[IS] OR "1613-9380"[IS] OR "0020-6814"[IS] OR "1938-2839"[IS] OR "1932-2968"[IS] OR "1932-2968"[IS] OR "1440-1746"[IS] OR "0815-9319"[IS] OR "1930-5311"[IS] OR "1930-532X"[IS] OR "1522-1598"[IS] OR "0022-3077"[IS] OR "0276-3869"[IS] OR "1540-9597"[IS] OR "1099-1557"[IS] OR "1053-8569"[IS] OR "2405-6316"[IS] OR "2405-6316"[IS] OR "0188-4409"[IS] OR "0188-4409"[IS] OR "2320-2890"[IS] OR "2319-4170"[IS] OR "2193-1127"[IS] OR "2193-1127"[IS] OR "2055-6225"[IS] OR "2055-6225"[IS] OR "0907-676X"[IS] OR "1747-6623"[IS] OR "0250-8281"[IS] OR "2320-0308"[IS] OR "1551-6865"[IS] OR "1551-6857"[IS] OR "1755-5817"[IS] OR "1878-0016"[IS] OR "1555-4139"[IS] OR "1555-4120"[IS] OR "1532-8392"[IS] OR "0046-8177"[IS] OR "1076-6332"[IS] OR "1878-4046"[IS] OR "1877-0657"[IS] OR "1877-0665"[IS] OR "1663-9812"[IS] OR "1663-9812"[IS] OR "1464-3332"[IS] OR "1464-3332"[IS] OR "1522-7200"[IS] OR "1523-908X"[IS] OR "1081-0730"[IS] OR "1087-0415"[IS] OR "2399-7532"[IS] OR "2399-7532"[IS] OR "0078-3218"[IS] OR "0078-3218"[IS] OR "2333-8113"[IS] OR "2333-8121"[IS] OR "2516-0230"[IS] OR "2516-0230"[IS] OR "0094-8373"[IS] OR "1938-5331"[IS] OR "1423-0291"[IS] OR "1015-2008"[IS] OR "1547-0164"[IS] OR "0889-7077"[IS] OR "1097-6752"[IS] OR "0889-5406"[IS] OR "1532-821X"[IS] OR "0003-9993"[IS] OR "1872-6054"[IS] OR "0168-8510"[IS] OR "0937-941X"[IS] OR "1433-2965"[IS] OR "1070-4698"[IS] OR "1070-4698"[IS] OR "1537-5129"[IS] OR "1537-5110"[IS] OR "1945-0826"[IS] OR "1049-510X"[IS] OR "1552-4523"[IS] OR "0733-4648"[IS] OR "2414-469X"[IS] OR "2414-4630"[IS] OR "1740-8776"[IS] OR "1740-8784"[IS] OR "2633-5409"[IS] OR "2633-5409"[IS] OR "1521-0111"[IS] OR "0026-895X"[IS] OR "2329-0382"[IS] OR "2329-0390"[IS] OR "2195-7177"[IS] OR "2195-7185"[IS] OR "0311-6999"[IS] OR "0311-6999"[IS] OR "1431-0635"[IS] OR "1431-0635"[IS] OR "2214-4048"[IS] OR "2214-4048"[IS] OR "1525-383X"[IS] OR "1525-383X"[IS] OR "2044-6055"[IS] OR "2044-6055"[IS] OR "2364-4974"[IS] OR "2364-4966"[IS] OR "0160-2896"[IS] OR "0160-2896"[IS] OR "0969-6016"[IS] OR "1475-3995"[IS] OR "2294-3307"[IS] OR "2030-1006"[IS] OR "1042-6914"[IS] OR "1532-2475"[IS] OR "0933-7407"[IS] OR "1439-0507"[IS] OR "1552-7565"[IS] OR "1077-8004"[IS] OR "1477-7827"[IS] OR "1477-7827"[IS] OR "2470-6566"[IS] OR "2470-6566"[IS] OR "1466-4208"[IS] OR "1466-4208"[IS] OR "2397-7264"[IS] OR "2397-7264"[IS] OR "1471-6909"[IS] OR "0954-2892"[IS] OR "1430-0532"[IS] OR "1613-415X"[IS] OR "1475-4983"[IS] OR "0031-0239"[IS] OR "2042-0196"[IS] OR "2042-0188"[IS] OR "0364-0213"[IS] OR "1551-6709"[IS] OR "1815-2406"[IS] OR "1991-7120"[IS] OR "0965-4313"[IS] OR "1469-5944"[IS] OR "0956-7135"[IS] OR "0956-7135"[IS] OR "1976-2283"[IS] OR "2005-1212"[IS] OR "0364-9024"[IS] OR "1097-0118"[IS] OR "1473-7124"[IS] OR "0308-2105"[IS] OR "0258-8900"[IS] OR "1432-0819"[IS] OR "1748-7382"[IS] OR "1354-8565"[IS] OR "0951-192X"[IS] OR "1362-3052"[IS] OR "1472-8117"[IS] OR "1472-8117"[IS] OR "2515-7647"[IS] OR "2515-7647"[IS] OR "1571-0068"[IS] OR "1571-0068"[IS] OR "1087-0547"[IS] OR "1557-1246"[IS] OR "1751-9020"[IS] OR "1751-9020"[IS] OR "1096-3642"[IS] OR "0024-4082"[IS] OR "0317-0861"[IS] OR "0317-0861"[IS] OR "0163-2116"[IS] OR "1573-2568"[IS] OR "1618-727X"[IS] OR "0897-1889"[IS] OR "0920-5861"[IS] OR "0920-5861"[IS] OR "1477-268X"[IS] OR "1029-8436"[IS] OR "1532-5024"[IS] OR "1532-5032"[IS] OR "1469-8005"[IS] OR "0269-8889"[IS] OR "1755-148X"[IS] OR "1755-1471"[IS] OR "0893-164X"[IS] OR "0893-164X"[IS] OR "1744-8980"[IS] OR "1573-2479"[IS] OR "0785-3890"[IS] OR "1651-2219"[IS] OR "0747-4938"[IS] OR "1532-4168"[IS] OR "1753-8955"[IS] OR "1753-8947"[IS] OR "1095-8622"[IS] OR "0889-9746"[IS] OR "2632-2498"[IS] OR "2632-2498"[IS] OR "0273-0979"[IS] OR "1088-9485"[IS] OR "2588-9133"[IS] OR "2588-9133"[IS] OR "2166-4250"[IS] OR "1793-6292"[IS] OR "1531-7048"[IS] OR "1065-6251"[IS] OR "2470-9239"[IS] OR "2470-9239"[IS] OR "1099-1069"[IS] OR "0278-0232"[IS] OR "2168-2291"[IS] OR "2168-2291"[IS] OR "1573-2657"[IS] OR "0142-4319"[IS] OR "2468-6964"[IS] OR "2468-6964"[IS] OR "0091-0260"[IS] OR "0091-0260"[IS] OR "2524-4914"[IS] OR "2524-4906"[IS] OR "1557-8070"[IS] OR "1531-1074"[IS] OR "0889-8553"[IS] OR "1120-3757"[IS] OR "2156-3381"[IS] OR "2156-3381"[IS] OR "1366-5839"[IS] OR "0022-0272"[IS] OR "2238-7854"[IS] OR "2238-7854"[IS] OR "1554-6578"[IS] OR "0022-3069"[IS] OR "2214-1405"[IS] OR "2214-1405"[IS] OR "1873-5126"[IS] OR "1353-8020"[IS] OR "0033-3158"[IS] OR "1432-2072"[IS] OR "1355-6215"[IS] OR "1369-1600"[IS] OR "1176-3647"[IS] OR "1436-4522"[IS] OR "2048-3694"[IS] OR

	"2048-3694"[IS] OR "1753-318X"[IS] OR "1753-318X"[IS] OR "0167-8299"[IS] OR "2191-0235"[IS] OR "0008-414X"[IS] OR "1496-4279"[IS] OR "1432-1890"[IS] OR "0940-6360"[IS] OR "2449-6499"[IS] OR "2083-2567"[IS] OR "1940-0683"[IS] OR "0022-0973"[IS] OR "0143-8166"[IS] OR "0143-8166"[IS] OR "0254-5330"[IS] OR "1572-9338"[IS] OR "1934-8282"[IS] OR "1934-8290"[IS] OR "0020-7683"[IS] OR "0020-7683"[IS] OR "1087-1357"[IS] OR "1528-8935"[IS] OR "1930-2975"[IS] OR "1930-2975"[IS] OR "1948-7193"[IS] OR "1948-7193"[IS] OR "2049-7121"[IS] OR "2049-7113"[IS] OR "1572-9710"[IS] OR "0960-3115"[IS] OR "0961-9534"[IS] OR "0961-9534"[IS] OR "1531-7072"[IS] OR "1070-5295"[IS] OR "1465-3419"[IS] OR "1355-7858"[IS] OR "1554-7914"[IS] OR "1554-7914"[IS] OR "2351-9894"[IS] OR "2351-9894"[IS] OR "1432-1831"[IS] OR "0300-8584"[IS] OR "1347-4820"[IS] OR "1346-9843"[IS] OR "2198-9753"[IS] OR "2198-9753"[IS] OR "1290-0729"[IS] OR "1290-0729"[IS] OR "0167-2789"[IS] OR "0167-2789"[IS] OR "2471-2906"[IS] OR "2471-2906"[IS] OR "0003-3472"[IS] OR "1095-8282"[IS] OR "2475-0328"[IS] OR "2475-0328"[IS] OR "1077-3118"[IS] OR "0003-6951"[IS] OR "1556-6935"[IS] OR "1040-9289"[IS] OR "2192-5682"[IS] OR "2192-5690"[IS] OR "1466-1810"[IS] OR "0267-3037"[IS] OR "0029-5981"[IS] OR "1097-0207"[IS] OR "0886-2605"[IS] OR "0886-2605"[IS] OR "0890-5339"[IS] OR "1531-2291"[IS] OR "0022-3476"[IS] OR "1097-6833"[IS] OR "0043-1648"[IS] OR "0043-1648"[IS] OR "1476-1645"[IS] OR "0002-9637"[IS] OR "0003-2670"[IS] OR "1873-4324"[IS] OR "1759-0914"[IS] OR "1759-0914"[IS] OR "1618-7598"[IS] OR "1439-6637"[IS] OR "1476-511X"[IS] OR "1476-511X"[IS] OR "1865-9284"[IS] OR "1865-9292"[IS] OR "2472-5552"[IS] OR "2472-5560"[IS] OR "1573-1928"[IS] OR "1381-2890"[IS] OR "1475-357X"[IS] OR "1475-3588"[IS] OR "1873-6130"[IS] OR "1570-677X"[IS] OR "2164-5515"[IS] OR "2164-554X"[IS] OR "1521-6543"[IS] OR "1521-6551"[IS] OR "0021-8499"[IS] OR "1740-1909"[IS] OR "1550-2783"[IS] OR "1550-2783"[IS] OR "0926-2601"[IS] OR "1572-929X"[IS] OR "1461-4456"[IS] OR "1461-7080"[IS] OR "1367-5494"[IS] OR "1367-5494"[IS] OR "2095-2767"[IS] OR "2095-2759"[IS] OR "0031-3998"[IS] OR "1530-0447"[IS] OR "2366-3987"[IS] OR "2366-3987"[IS] OR "1869-6961"[IS] OR "1869-6953"[IS] OR "1751-7583"[IS] OR "1751-7575"[IS] OR "1525-1489"[IS] OR "0885-0666"[IS] OR "1460-2407"[IS] OR "1360-9947"[IS] OR "1540-7969"[IS] OR "1540-7969"[IS] OR "1573-2800"[IS] OR "0004-0002"[IS] OR "1099-0968"[IS] OR "1072-4133"[IS] OR "1941-1197"[IS] OR "1939-1390"[IS] OR "2211-3207"[IS] OR "2211-3207"[IS] OR "1613-4133"[IS] OR "1613-4125"[IS] OR "1542-7617"[IS] OR "1542-7609"[IS] OR "1542-2119"[IS] OR "1542-2119"[IS] OR "2212-0963"[IS] OR "2212-0963"[IS] OR "1866-9964"[IS] OR "1866-9956"[IS] OR "2325-1026"[IS] OR "2325-1026"[IS] OR "1422-6928"[IS] OR "1422-6952"[IS] OR "1748-717X"[IS] OR "1748-717X"[IS] OR "0141-9889"[IS] OR "1467-9566"[IS] OR "2329-7778"[IS] OR "2329-7778"[IS] OR "2096-7071"[IS] OR "2096-7071"[IS] OR "2215-0986"[IS] OR "2215-0986"[IS] OR "0340-6199"[IS] OR "1432-1076"[IS] OR "0740-0020"[IS] OR "1095-9998"[IS] OR "1940-1906"[IS] OR "0161-5440"[IS] OR "1022-1336"[IS] OR "1521-3927"[IS] OR "1572-8366"[IS] OR "0889-048X"[IS] OR "1351-0754"[IS] OR "1365-2389"[IS] OR "1012-6902"[IS] OR "1012-6902"[IS] OR "2053-8790"[IS] OR "2053-8790"[IS] OR "1471-2393"[IS] OR "1471-2393"[IS] OR "0260-3594"[IS] OR "1548-9574"[IS] OR "2193-8210"[IS] OR "2190-9172"[IS] OR "1071-5797"[IS] OR "1090-2465"[IS] OR "1179-8602"[IS] OR "1179-8602"[IS] OR "1878-5786"[IS] OR "1367-9120"[IS] OR "2254-7339"[IS] OR "2254-7339"[IS] OR "0308-597X"[IS] OR "0308-597X"[IS] OR "0891-3668"[IS] OR "1532-0987"[IS] OR "2475-4455"[IS] OR "2475-4455"[IS] OR "0198-7429"[IS] OR "0198-7429"[IS] OR "1552-3594"[IS] OR "0093-8548"[IS] OR "0925-1022"[IS] OR "1573-7586"[IS] OR "0883-5993"[IS] OR "1536-0237"[IS] OR "2050-0521"[IS] OR "2050-0521"[IS] OR "1871-5192"[IS] OR "1871-5192"[IS] OR "0099-2240"[IS] OR "1098-5336"[IS] OR "1473-6535"[IS] OR "0957-9672"[IS] OR "1057-9230"[IS] OR "1099-1050"[IS] OR "2644-1276"[IS] OR "2644-1276"[IS] OR "1869-5868"[IS] OR "0021-2148"[IS] OR "2589-0360"[IS] OR "2226-5856"[IS] OR "0894-0282"[IS] OR "0894-0282"[IS] OR "1558-7673"[IS] OR "1558-7673"[IS] OR "1532-7647"[IS] OR "1524-8372"[IS] OR "1042-4431"[IS] OR "1042-4431"[IS] OR "1573-6644"[IS] OR "0146-7239"[IS] OR "1177-1062"[IS] OR "1177-1062"[IS] OR "0739-8859"[IS] OR "0739-8859"[IS] OR "1077-5595"[IS] OR "1077-5595"[IS] OR "0176-2680"[IS] OR "0176-2680"[IS] OR "1866-8887"[IS] OR "1867-0717"[IS] OR "2090-8024"[IS] OR "2090-0252"[IS] OR "1791-244X"[IS] OR "1107-3756"[IS] OR "0391-2078"[IS] OR "1972-4977"[IS] OR "1759-6831"[IS] OR "1674-4918"[IS] OR "1464-360X"[IS] OR "1101-1262"[IS] OR "1664-042X"[IS] OR "1664-042X"[IS] OR "1932-7447"[IS] OR "1932-7455"[IS] OR "1469-4417"[IS] OR "0143-3857"[IS] OR "1540-6288"[IS] OR "0732-8516"[IS] OR "0999-193X"[IS] OR "1297-9686"[IS] OR "1056-7895"[IS] OR "1530-7921"[IS] OR "1745-7289"[IS] OR "1745-7289"[IS] OR "2287-2892"[IS] OR "2288-2561"[IS] OR "1303-2968"[IS] OR "1303-2968"[IS] OR "1683-1470"[IS] OR "1683-1470"[IS] OR "2399-8083"[IS] OR "2399-8091"[IS] OR "1943-5533"[IS] OR "0899-1561"[IS] OR "1543-4303"[IS] OR "1543-4311"[IS] OR "1552-8618"[IS] OR "0730-7268"[IS] OR "0168-6496"[IS] OR "1574-6941"[IS] OR "1477-0911"[IS] OR "0959-6836"[IS] OR "1882-6695"[IS] OR "1882-6695"[IS] OR "1432-1378"[IS] OR "0933-2790"[IS] OR "0090-502X"[IS] OR "1532-5946"[IS] OR "1314-4057"[IS] OR "1314-4049"[IS] OR "0887-0446"[IS] OR "1476-8321"[IS] OR "1749-4001"[IS] OR "0034-4893"[IS] OR "2196-0216"[IS] OR "2196-0216"[IS] OR "1359-5997"[IS] OR "1871-6873"[IS] OR "2332-8878"[IS] OR "2096-4129"[IS] OR "1089-7801"[IS] OR "1089-7801"[IS] OR "1420-9039"[IS] OR

	"0044-2275"[IS] OR "1573-3289"[IS] OR "0894-587X"[IS] OR "1540-2010"[IS] OR "1540-2002"[IS] OR "1754-6834"[IS] OR "1754-6834"[IS] OR "2624-893X"[IS] OR "2624-893X"[IS] OR "1526-4998"[IS] OR "1526-498X"[IS] OR "1536-0040"[IS] OR "1536-0040"[IS] OR "2379-1691"[IS] OR "2379-1683"[IS] OR "0964-8305"[IS] OR "0964-8305"[IS] OR "0022-2216"[IS] OR "0022-2216"[IS] OR "1435-9537"[IS] OR "1435-9529"[IS] OR "2196-3061"[IS] OR "2196-3061"[IS] OR "1179-2744"[IS] OR "1179-2744"[IS] OR "2193-6528"[IS] OR "2193-8245"[IS] OR "1573-6822"[IS] OR "0742-2091"[IS] OR "1433-2981"[IS] OR "0936-6555"[IS] OR "1973-9095"[IS] OR "1973-9087"[IS] OR "0721-832X"[IS] OR "1435-702X"[IS] OR "2573-9603"[IS] OR "2573-9603"[IS] OR "1873-4529"[IS] OR "0952-8180"[IS] OR "1464-3758"[IS] OR "1369-3034"[IS] OR "1588-2861"[IS] OR "0138-9130"[IS] OR "0829-318X"[IS] OR "1758-4469"[IS] OR "2045-7022"[IS] OR "2045-7022"[IS] OR "0733-8635"[IS] OR "0733-8635"[IS] OR "1742-5247"[IS] OR "1742-5247"[IS] OR "1477-9145"[IS] OR "0022-0949"[IS] OR "0892-6875"[IS] OR "0892-6875"[IS] OR "1175-5652"[IS] OR "1179-1896"[IS] OR "1744-9081"[IS] OR "1744-9081"[IS] OR "0883-0355"[IS] OR "0883-0355"[IS] OR "1880-3873"[IS] OR "1340-3478"[IS] OR "0171-5216"[IS] OR "1432-1335"[IS] OR "1552-5465"[IS] OR "1070-4965"[IS] OR "2055-6659"[IS] OR "2055-6640"[IS] OR "0924-3453"[IS] OR "1744-5124"[IS] OR "1743-8594"[IS] OR "1743-8586"[IS] OR "1532-7078"[IS] OR "1525-0008"[IS] OR "0021-8693"[IS] OR "1090-266X"[IS] OR "1558-349X"[IS] OR "1546-1440"[IS] OR "0031-2746"[IS] OR "1477-464X"[IS] OR "2164-3970"[IS] OR "2164-3962"[IS] OR "1467-9590"[IS] OR "0022-2526"[IS] OR "0094-5765"[IS] OR "0094-5765"[IS] OR "2666-3074"[IS] OR "2666-3074"[IS] OR "2192-6395"[IS] OR "2095-0055"[IS] OR "1879-6400"[IS] OR "1879-6397"[IS] OR "1530-7972"[IS] OR "1099-6362"[IS] OR "2330-1643"[IS] OR "2330-1635"[IS] OR "2352-7714"[IS] OR "2352-7714"[IS] OR "2469-9896"[IS] OR "2469-9896"[IS] OR "1349-2896"[IS] OR "0386-2208"[IS] OR "0033-0655"[IS] OR "0300-9440"[IS] OR "1097-0045"[IS] OR "0270-4137"[IS] OR "2152-0828"[IS] OR "2152-081X"[IS] OR "2624-6511"[IS] OR "2624-6511"[IS] OR "1617-6278"[IS] OR "0939-6314"[IS] OR "1521-7388"[IS] OR "0278-6826"[IS] OR "2092-7193"[IS] OR "2092-7193"[IS] OR "1464-2859"[IS] OR "1464-2859"[IS] OR "2095-4719"[IS] OR "2052-3289"[IS] OR "0021-9762"[IS] OR "1097-4679"[IS] OR "1759-9954"[IS] OR "1759-9962"[IS] OR "0724-4983"[IS] OR "1433-8726"[IS] OR "0300-9483"[IS] OR "1502-3885"[IS] OR "1442-9071"[IS] OR "1442-6404"[IS] OR "0172-8083"[IS] OR "1432-0983"[IS] OR "1573-1413"[IS] OR "1386-145X"[IS] OR "0304-386X"[IS] OR "0304-386X"[IS] OR "1466-4445"[IS] OR "1352-7266"[IS] OR "1531-586X"[IS] OR "1083-4362"[IS] OR "0969-0239"[IS] OR "0969-0239"[IS] OR "2772-4875"[IS] OR "2772-4875"[IS] OR "1466-7657"[IS] OR "0020-8132"[IS] OR "2206-5865"[IS] OR "2206-5865"[IS] OR "2452-3062"[IS] OR "2452-3062"[IS] OR "2303-9027"[IS] OR "2226-7190"[IS] OR "0143-7739"[IS] OR "0143-7739"[IS] OR "0889-938X"[IS] OR "1573-7160"[IS] OR "1878-0946"[IS] OR "1744-165X"[IS] OR "0167-4943"[IS] OR "0167-4943"[IS] OR "2211-3576"[IS] OR "2211-3568"[IS] OR "0021-9746"[IS] OR "1472-4146"[IS] OR "1100-9233"[IS] OR "1100-9233"[IS] OR "2204-2091"[IS] OR "2204-2091"[IS] OR "1432-0541"[IS] OR "0178-4617"[IS] OR "1424-0637"[IS] OR "1424-0661"[IS] OR "1547-6898"[IS] OR "1040-8444"[IS] OR "0947-3580"[IS] OR "0947-3580"[IS] OR "2213-1272"[IS] OR "2213-1272"[IS] OR "2329-9460"[IS] OR "2329-9037"[IS] OR "0029-8519"[IS] OR "0029-1939"[IS] OR "1533-385X"[IS] OR "0001-1452"[IS] OR "2666-9528"[IS] OR "2096-9147"[IS] OR "0025-3227"[IS] OR "0025-3227"[IS] OR "1866-2625"[IS] OR "1866-2633"[IS] OR "0196-0644"[IS] OR "1097-6760"[IS] OR "1535-6841"[IS] OR "1535-6841"[IS] OR "0012-3706"[IS] OR "1530-0358"[IS] OR "1381-4788"[IS] OR "1751-1402"[IS] OR "2296-665X"[IS] OR "2296-665X"[IS] OR "1226-086X"[IS] OR "1226-086X"[IS] OR "0743-684X"[IS] OR "1098-8947"[IS] OR "2352-9520"[IS] OR "2352-9520"[IS] OR "1672-5107"[IS] OR "1995-8226"[IS] OR "0031-9023"[IS] OR "1538-6724"[IS] OR "1873-328X"[IS] OR "0032-5910"[IS] OR "0094-0496"[IS] OR "1548-1425"[IS] OR "2193-6544"[IS] OR "2193-8261"[IS] OR "2365-7464"[IS] OR "2365-7464"[IS] OR "2245-408X"[IS] OR "2245-408X"[IS] OR "2197-4314"[IS] OR "2197-4314"[IS] OR "1097-9883"[IS] OR "0360-2532"[IS] OR "1530-891X"[IS] OR "1934-2403"[IS] OR "0950-0693"[IS] OR "1464-5289"[IS] OR "2469-9888"[IS] OR "2469-9888"[IS] OR "0958-2029"[IS] OR "1471-5449"[IS] OR "1931-7565"[IS] OR "1931-7557"[IS] OR "0010-0870"[IS] OR "2150-6701"[IS] OR "0018-9391"[IS] OR "1558-0040"[IS] OR "1868-808X"[IS] OR "1868-8071"[IS] OR "2214-3599"[IS] OR "2214-3602"[IS] OR "0022-3115"[IS] OR "0022-3115"[IS] OR "1463-6409"[IS] OR "0300-3256"[IS] OR "0022-3778"[IS] OR "1469-7807"[IS] OR "1538-8239"[IS] OR "1538-8220"[IS] OR "0254-4962"[IS] OR "1423-033X"[IS] OR "0014-2999"[IS] OR "1879-0712"[IS] OR "1746-9902"[IS] OR "1747-6348"[IS] OR "1460-9584"[IS] OR "1268-7731"[IS] OR "1601-183X"[IS] OR "1601-1848"[IS] OR "1938-2480"[IS] OR "1538-8506"[IS] OR "2199-4668"[IS] OR "2199-4676"[IS] OR "1871-6784"[IS] OR "1876-4347"[IS] OR "1873-5134"[IS] OR "0738-3991"[IS] OR "2637-6105"[IS] OR "2637-6105"[IS] OR "2056-5909"[IS] OR "2056-5909"[IS] OR "1723-7785"[IS] OR "1723-7785"[IS] OR "1467-9507"[IS] OR "0961-205X"[IS] OR "2047-1033"[IS] OR "2047-1025"[IS] OR "1756-1663"[IS] OR "1340-2838"[IS] OR "1348-4214"[IS] OR "0916-9636"[IS] OR "0888-7993"[IS] OR "1558-7975"[IS] OR "1474-774X"[IS] OR "1474-7731"[IS] OR "0020-1669"[IS] OR "1520-510X"[IS] OR "1541-4469"[IS] OR "0020-7314"[IS] OR "1540-3602"[IS] OR "0091-8369"[IS] OR "2724-6442"[IS] OR "2724-6051"[IS] OR "1590-3729"[IS] OR "0939-4753"[IS] OR "2235-6282"[IS] OR "2235-6282"[IS] OR "1538-4047"[IS] OR

	"1555-8576"[IS] OR "2367-0932"[IS] OR "2367-0932"[IS] OR "1342-078X"[IS] OR "1347-4715"[IS] OR "1708-8240"[IS] OR "1496-4155"[IS] OR "2212-4632"[IS] OR "1883-1958"[IS] OR "2196-0739"[IS] OR "2196-0739"[IS] OR "1556-276X"[IS] OR "1931-7573"[IS] OR "1741-3095"[IS] OR "1462-4745"[IS] OR "1479-3555"[IS] OR "1479-3555"[IS] OR "1058-2916"[IS] OR "1538-943X"[IS] OR "1469-2120"[IS] OR "0024-6093"[IS] OR "1473-7175"[IS] OR "1744-8360"[IS] OR "1873-3514"[IS] OR "0028-3932"[IS] OR "2590-051X"[IS] OR "2590-051X"[IS] OR "1355-5855"[IS] OR "1758-4248"[IS] OR "1528-3968"[IS] OR "0029-6554"[IS] OR "2215-017X"[IS] OR "2215-017X"[IS] OR "2158-9100"[IS] OR "0225-5189"[IS] OR "1477-7525"[IS] OR "1477-7525"[IS] OR "0020-8523"[IS] OR "1461-7226"[IS] OR "1520-7439"[IS] OR "1520-7439"[IS] OR "1976-7846"[IS] OR "1976-1902"[IS] OR "0016-8025"[IS] OR "1365-2478"[IS] OR "2040-1116"[IS] OR "2040-1124"[IS] OR "1933-1681"[IS] OR "1933-169X"[IS] OR "2397-2106"[IS] OR "2397-2106"[IS] OR "0171-6468"[IS] OR "1436-6304"[IS] OR "0002-9343"[IS] OR "1555-7162"[IS] OR "0006-3363"[IS] OR "1529-7268"[IS] OR "2538-2128"[IS] OR "2538-2136"[IS] OR "1040-8703"[IS] OR "1531-698X"[IS] OR "2041-7314"[IS] OR "2041-7314"[IS] OR "2211-3355"[IS] OR "2211-3355"[IS] OR "2352-5568"[IS] OR "2352-5568"[IS] OR "2243-7908"[IS] OR "0119-5646"[IS] OR "2379-4607"[IS] OR "2379-4615"[IS] OR "2590-0536"[IS] OR "2590-0536"[IS] OR "1746-4358"[IS] OR "1746-4358"[IS] OR "1865-1674"[IS] OR "1865-1682"[IS] OR "1018-8665"[IS] OR "1421-9832"[IS] OR "2055-2076"[IS] OR "2055-2076"[IS] OR "1468-1293"[IS] OR "1464-2662"[IS] OR "1750-5836"[IS] OR "1750-5836"[IS] OR "2162-6057"[IS] OR "0022-0175"[IS] OR "1598-6357"[IS] OR "1011-8934"[IS] OR "0889-1583"[IS] OR "1095-8681"[IS] OR "1751-2786"[IS] OR "1751-2794"[IS] OR "1741-7899"[IS] OR "1470-1626"[IS] OR "1055-0496"[IS] OR "1521-0391"[IS] OR "0272-4340"[IS] OR "1573-6830"[IS] OR "1354-7798"[IS] OR "1468-036X"[IS] OR "1432-5195"[IS] OR "0341-2695"[IS] OR "1361-6587"[IS] OR "0741-3335"[IS] OR "0926-860X"[IS] OR "1873-3875"[IS] OR "1432-0878"[IS] OR "0302-766X"[IS] OR "1369-6513"[IS] OR "1369-7625"[IS] OR "1530-437X"[IS] OR "1530-437X"[IS] OR "1460-7425"[IS] OR "1460-7425"[IS] OR "0267-8179"[IS] OR "1099-1417"[IS] OR "0190-2725"[IS] OR "1939-8999"[IS] OR "2198-4018"[IS] OR "2198-4026"[IS] OR "0963-1690"[IS] OR "1467-8691"[IS] OR "0959-0552"[IS] OR "0959-0552"[IS] OR "2522-896X"[IS] OR "2522-8978"[IS] OR "0032-3292"[IS] OR "1552-7514"[IS] OR "1390-3306"[IS] OR "1138-2783"[IS] OR "1099-1727"[IS] OR "0883-7066"[IS] OR "0039-9140"[IS] OR "1873-3573"[IS] OR "0169-1317"[IS] OR "0169-1317"[IS] OR "1687-1499"[IS] OR "1687-1472"[IS] OR "2380-6567"[IS] OR "2380-6567"[IS] OR "0301-9322"[IS] OR "0301-9322"[IS] OR "1941-5257"[IS] OR "1941-5265"[IS] OR "2211-9493"[IS] OR "2211-9493"[IS] OR "0570-1783"[IS] OR "0570-1783"[IS] OR "1538-4101"[IS] OR "1551-4005"[IS] OR "1591-8890"[IS] OR "1591-9528"[IS] OR "1935-5149"[IS] OR "1935-5130"[IS] OR "2156-342X"[IS] OR "2156-342X"[IS] OR "0735-1933"[IS] OR "0735-1933"[IS] OR "1353-3312"[IS] OR "1743-906X"[IS] OR "0303-8300"[IS] OR "1573-0921"[IS] OR "2379-6146"[IS] OR "2379-6146"[IS] OR "2469-990X"[IS] OR "2469-990X"[IS] OR "1547-5069"[IS] OR "1527-6546"[IS] OR "1600-5775"[IS] OR "0909-0495"[IS] OR "2090-1844"[IS] OR "2090-1836"[IS] OR "1096-0929"[IS] OR "1096-6080"[IS] OR "0146-6216"[IS] OR "1552-3497"[IS] OR "1094-1665"[IS] OR "1741-6507"[IS] OR "2096-4471"[IS] OR "2574-5417"[IS] OR "2731-4553"[IS] OR "2731-4553"[IS] OR "1740-7753"[IS] OR "1740-7745"[IS] OR "2192-0567"[IS] OR "2192-0567"[IS] OR "1751-8687"[IS] OR "1751-8695"[IS] OR "1463-9971"[IS] OR "1463-9963"[IS] OR "2214-0301"[IS] OR "2214-0301"[IS] OR "1353-3452"[IS] OR "1471-5546"[IS] OR "2095-8137"[IS] OR "2095-8137"[IS] OR "0803-5253"[IS] OR "1651-2227"[IS] OR "2578-4854"[IS] OR "2578-4854"[IS] OR "1878-5077"[IS] OR "1878-5085"[IS] OR "1476-5543"[IS] OR "0743-8346"[IS] OR "0022-3611"[IS] OR "1573-0433"[IS] OR "0735-2166"[IS] OR "1467-9906"[IS] OR "2321-0281"[IS] OR "2277-9760"[IS] OR "1542-7595"[IS] OR "1542-7587"[IS] OR "0732-118X"[IS] OR "0732-118X"[IS] OR "1470-1197"[IS] OR "1464-9365"[IS] OR "0888-613X"[IS] OR "1873-4731"[IS] OR "1479-1838"[IS] OR "1472-0817"[IS] OR "1661-6960"[IS] OR "1661-6952"[IS] OR "1432-2048"[IS] OR "0032-0935"[IS] OR "1079-0632"[IS] OR "1573-286X"[IS] OR "1931-4523"[IS] OR "1931-4531"[IS] OR "1095-9939"[IS] OR "0048-3575"[IS] OR "0346-1238"[IS] OR "1651-2030"[IS] OR "2398-5348"[IS] OR "2398-5348"[IS] OR "0306-4379"[IS] OR "0306-4379"[IS] OR "2192-113X"[IS] OR "2192-113X"[IS] OR "1050-6926"[IS] OR "1559-002X"[IS] OR "0275-5408"[IS] OR "1475-1313"[IS] OR "2210-5395"[IS] OR "2210-5395"[IS] OR "1753-3740"[IS] OR "1532-169X"[IS] OR "1809-9246"[IS] OR "1413-3555"[IS] OR "1000-1026"[IS] OR "1000-1026"[IS] OR "1662-5153"[IS] OR "1662-5153"[IS] OR "1662-5196"[IS] OR "1662-5196"[IS] OR "2054-3085"[IS] OR "2054-3085"[IS] OR "1069-0727"[IS] OR "1552-4590"[IS] OR "0957-8234"[IS] OR "0957-8234"[IS] OR "2666-0334"[IS] OR "2666-0334"[IS] OR "0090-5550"[IS] OR "1939-1544"[IS] OR "2040-6231"[IS] OR "2040-6223"[IS] OR "1869-0327"[IS] OR "1869-0327"[IS] OR "2190-5495"[IS] OR "2190-5487"[IS] OR "1855-3974"[IS] OR "1855-3966"[IS] OR "0190-7409"[IS] OR "0190-7409"[IS] OR "1477-0296"[IS] OR "0309-1333"[IS] OR "0323-3847"[IS] OR "1521-4036"[IS] OR "1091-7683"[IS] OR "0748-1187"[IS] OR "2197-425X"[IS] OR "2197-425X"[IS] OR "0022-1554"[IS] OR "1551-5044"[IS] OR "2772-2716"[IS] OR "2772-2716"[IS] OR "2045-2322"[IS] OR "2045-2322"[IS] OR "1748-4995"[IS] OR "1748-5002"[IS] OR "0961-3218"[IS] OR "1466-4321"[IS] OR "1476-5411"[IS] OR "1367-0484"[IS] OR "2365-421X"[IS] OR "2365-421X"[IS] OR "1758-2229"[IS] OR "1758-2229"[IS] OR "0305-0009"[IS] OR

	"1469-7602"[IS] OR "2000-2297"[IS] OR "2000-2297"[IS] OR "1521-0103"[IS] OR "0022-3565"[IS] OR "2452-2627"[IS] OR "2452-2627"[IS] OR "1009-5020"[IS] OR "1009-5020"[IS] OR "2195-9706"[IS] OR "2195-9706"[IS] OR "0278-0070"[IS] OR "0278-0070"[IS] OR "0018-9375"[IS] OR "0018-9375"[IS] OR "1499-2027"[IS] OR "1708-8186"[IS] OR "1050-0472"[IS] OR "1050-0472"[IS] OR "1674-4926"[IS] OR "1674-4926"[IS] OR "0964-1998"[IS] OR "1467-985X"[IS] OR "1548-2456"[IS] OR "1531-426X"[IS] OR "2666-7894"[IS] OR "2666-7894"[IS] OR "1879-3460"[IS] OR "0168-1605"[IS] OR "1091-255X"[IS] OR "1873-4626"[IS] OR "0023-9216"[IS] OR "1540-5893"[IS] OR "2255-4165"[IS] OR "2255-4165"[IS] OR "1038-4111"[IS] OR "1744-7941"[IS] OR "1540-8167"[IS] OR "1045-3873"[IS] OR "0959-1524"[IS] OR "0959-1524"[IS] OR "2329-423X"[IS] OR "2329-4248"[IS] OR "1098-9048"[IS] OR "1069-3424"[IS] OR "2364-3587"[IS] OR "2364-3579"[IS] OR "1432-0614"[IS] OR "0175-7598"[IS] OR "1472-6785"[IS] OR "1472-6785"[IS] OR "2522-087X"[IS] OR "2522-087X"[IS] OR "0045-7930"[IS] OR "0045-7930"[IS] OR "1536-4798"[IS] OR "0277-3740"[IS] OR "2161-3311"[IS] OR "2161-3311"[IS] OR "1341-9625"[IS] OR "1437-7772"[IS] OR "1614-2241"[IS] OR "1614-1881"[IS] OR "1875-9114"[IS] OR "0277-0008"[IS] OR "1939-151X"[IS] OR "1524-9220"[IS] OR "1569-190X"[IS] OR "1569-190X"[IS] OR "1745-5065"[IS] OR "1745-5057"[IS] OR "1548-5595"[IS] OR "1548-5609"[IS] OR "1863-7221"[IS] OR "1864-8312"[IS] OR "1007-5704"[IS] OR "1007-5704"[IS] OR "2047-2382"[IS] OR "2047-2382"[IS] OR "0143-9782"[IS] OR "1467-9892"[IS] OR "0091-3057"[IS] OR "1873-5177"[IS] OR "1537-4521"[IS] OR "0148-5717"[IS] OR "2196-7091"[IS] OR "2196-7091"[IS] OR "1079-9389"[IS] OR "1079-9389"[IS] OR "1473-4230"[IS] OR "1473-4222"[IS] OR "1461-7161"[IS] OR "0959-3535"[IS] OR "1815-7920"[IS] OR "1027-3719"[IS] OR "1879-1964"[IS] OR "0021-8502"[IS] OR "1873-1244"[IS] OR "0899-9007"[IS] OR "1432-0827"[IS] OR "0171-967X"[IS] OR "0267-3843"[IS] OR "0267-3843"[IS] OR "0172-4622"[IS] OR "1439-3964"[IS] OR "1757-2215"[IS] OR "1757-2215"[IS] OR "1601-6335"[IS] OR "1601-6343"[IS] OR "0164-0275"[IS] OR "1552-7573"[IS] OR "1461-7439"[IS] OR "1362-4806"[IS] OR "1472-6963"[IS] OR "1472-6963"[IS] OR "1522-726X"[IS] OR "1522-1946"[IS] OR "8750-7587"[IS] OR "1522-1601"[IS] OR "2396-8923"[IS] OR "2396-8923"[IS] OR "1450-2194"[IS] OR "1758-888X"[IS] OR "2352-0094"[IS] OR "2352-0094"[IS] OR "1051-1482"[IS] OR "2152-050X"[IS] OR "2573-7732"[IS] OR "2573-7732"[IS] OR "2214-3173"[IS] OR "2214-3173"[IS] OR "1743-7075"[IS] OR "1743-7075"[IS] OR "1873-5649"[IS] OR "0096-3003"[IS] OR "2196-9744"[IS] OR "2196-9744"[IS] OR "1468-5973"[IS] OR "0966-0879"[IS] OR "2058-9689"[IS] OR "2058-9689"[IS] OR "1545-5017"[IS] OR "1545-5009"[IS] OR "1541-1559"[IS] OR "1541-1559"[IS] OR "0008-6568"[IS] OR "1421-976X"[IS] OR "2365-4252"[IS] OR "2365-4244"[IS] OR "1745-3992"[IS] OR "0731-1745"[IS] OR "2397-1835"[IS] OR "2397-1835"[IS] OR "0022-4065"[IS] OR "0022-4065"[IS] OR "1520-6750"[IS] OR "0894-069X"[IS] OR "1756-3305"[IS] OR "1756-3305"[IS] OR "1262-3377"[IS] OR "1292-8119"[IS] OR "0144-235X"[IS] OR "1366-591X"[IS] OR "1098-6111"[IS] OR "1552-745X"[IS] OR "2164-2591"[IS] OR "2164-2591"[IS] OR "2578-0611"[IS] OR "2578-062X"[IS] OR "1878-5891"[IS] OR "0378-5955"[IS] OR "2411-2119"[IS] OR "0960-233X"[IS] OR "1309-1042"[IS] OR "1309-1042"[IS] OR "2333-5084"[IS] OR "2333-5084"[IS] OR "1139-613X"[IS] OR "1139-613X"[IS] OR "2215-1532"[IS] OR "2215-1532"[IS] OR "2192-5372"[IS] OR "2192-5372"[IS] OR "2586-6583"[IS] OR "2586-6591"[IS] OR "2516-1571"[IS] OR "2096-5303"[IS] OR "1936-900X"[IS] OR "1936-9018"[IS] OR "2690-0637"[IS] OR "2690-0637"[IS] OR "1651-226X"[IS] OR "0284-186X"[IS] OR "1572-9885"[IS] OR "0168-6577"[IS] OR "1365-2427"[IS] OR "0046-5070"[IS] OR "1941-2444"[IS] OR "0148-6071"[IS] OR "2154-3321"[IS] OR "2156-2261"[IS] OR "2211-050X"[IS] OR "1033-2170"[IS] OR "0037-0738"[IS] OR "0037-0738"[IS] OR "1098-2140"[IS] OR "1098-2140"[IS] OR "1861-4728"[IS] OR "1861-471X"[IS] OR "2057-4991"[IS] OR "2057-4991"[IS] OR "0966-9795"[IS] OR "0966-9795"[IS] OR "1943-7722"[IS] OR "0002-9173"[IS] OR "2156-7085"[IS] OR "2156-7085"[IS] OR "0305-764X"[IS] OR "1469-3577"[IS] OR "1940-1892"[IS] OR "0095-8964"[IS] OR "1478-7954"[IS] OR "1478-7954"[IS] OR "1570-8268"[IS] OR "1570-8268"[IS] OR "2701-0198"[IS] OR "2701-0198"[IS] OR "1435-5558"[IS] OR "1435-5566"[IS] OR "0886-7356"[IS] OR "1548-1360"[IS] OR "1552-4957"[IS] OR "1552-4949"[IS] OR "2296-2646"[IS] OR "2296-2646"[IS] OR "2194-7899"[IS] OR "2194-7899"[IS] OR "1386-9620"[IS] OR "1572-9389"[IS] OR "0191-5886"[IS] OR "1573-3653"[IS] OR "0013-4651"[IS] OR "1945-7111"[IS] OR "1617-9625"[IS] OR "2070-7266"[IS] OR "1745-1744"[IS] OR "0003-598X"[IS] OR "0168-9274"[IS] OR "0168-9274"[IS] OR "0022-4545"[IS] OR "1940-1183"[IS] OR "0364-3190"[IS] OR "1573-6903"[IS] OR "2468-3124"[IS] OR "2468-3124"[IS] OR "0258-8013"[IS] OR "0258-8013"[IS] OR "1465-7279"[IS] OR "1045-2249"[IS] OR "2160-1836"[IS] OR "2160-1836"[IS] OR "2045-2543"[IS] OR "2045-2543"[IS] OR "0021-2172"[IS] OR "1565-8511"[IS] OR "1467-9760"[IS] OR "0963-8016"[IS] OR "1568-4555"[IS] OR "1568-4555"[IS] OR "1543-8635"[IS] OR "1543-8627"[IS] OR "1672-6308"[IS] OR "1672-6308"[IS] OR "0731-1214"[IS] OR "1533-8673"[IS] OR "1879-3649"[IS] OR "1537-1891"[IS] OR "0964-3397"[IS] OR "0964-3397"[IS] OR "0192-5121"[IS] OR "1460-373X"[IS] OR "1750-399X"[IS] OR "1757-0417"[IS] OR "1050-5350"[IS] OR "1573-3602"[IS] OR "0167-7055"[IS] OR "1467-8659"[IS] OR "0045-7906"[IS] OR "0045-7906"[IS] OR "2665-9727"[IS] OR "2665-9727"[IS] OR "0957-4824"[IS] OR "1460-2245"[IS] OR "1750-1237"[IS] OR "1750-1229"[IS] OR "0022-0221"[IS] OR "1552-5422"[IS] OR "2206-7418"[IS] OR "2206-7418"[IS] OR "2452-073X"[IS] OR

	"2452-073X"[IS] OR "1746-031X"[IS] OR "1618-4742"[IS] OR "1608-4799"[IS] OR "0923-4748"[IS] OR "2666-2485"[IS] OR "2666-2485"[IS] OR "2378-9638"[IS] OR "2378-962X"[IS] OR "2373-9878"[IS] OR "2373-9878"[IS] OR "1552-387X"[IS] OR "0011-1287"[IS] OR "0143-4004"[IS] OR "1532-3102"[IS] OR "1472-6483"[IS] OR "1472-6491"[IS] OR "1095-7162"[IS] OR "0895-4798"[IS] OR "1573-7861"[IS] OR "0884-8971"[IS] OR "1399-6576"[IS] OR "0001-5172"[IS] OR "1756-137X"[IS] OR "1756-1388"[IS] OR "1743-6095"[IS] OR "1743-6109"[IS] OR "1472-2747"[IS] OR "1472-2739"[IS] OR "1096-603X"[IS] OR "1063-5203"[IS] OR "0925-8574"[IS] OR "0925-8574"[IS] OR "2469-7311"[IS] OR "2469-7311"[IS] OR "0279-1072"[IS] OR "0279-1072"[IS] OR "1532-2793"[IS] OR "0260-6917"[IS] OR "0257-8972"[IS] OR "0257-8972"[IS] OR "1435-9871"[IS] OR "1435-9871"[IS] OR "2050-6201"[IS] OR "2050-6201"[IS] OR "2590-1575"[IS] OR "2590-1575"[IS] OR "2110-7017"[IS] OR "2110-7017"[IS] OR "1365-263X"[IS] OR "0960-7439"[IS] OR "1552-4574"[IS] OR "1050-6519"[IS] OR "1389-2843"[IS] OR "1389-2843"[IS] OR "1793-8759"[IS] OR "1793-9453"[IS] OR "1946-3014"[IS] OR "1946-3022"[IS] OR "1559-0097"[IS] OR "1046-3976"[IS] OR "0944-1344"[IS] OR "1614-7499"[IS] OR "1744-7917"[IS] OR "1672-9609"[IS] OR "1871-403X"[IS] OR "1871-403X"[IS] OR "2468-2659"[IS] OR "2096-2703"[IS] OR "2331-2165"[IS] OR "2331-2165"[IS] OR "2234-3806"[IS] OR "2234-3814"[IS] OR "1552-3349"[IS] OR "0002-7162"[IS] OR "1464-2867"[IS] OR "1747-0059"[IS] OR "2667-3258"[IS] OR "2667-3258"[IS] OR "2096-3947"[IS] OR "2414-6447"[IS] OR "0167-6997"[IS] OR "1573-0646"[IS] OR "1570-7385"[IS] OR "1570-7385"[IS] OR "2040-8021"[IS] OR "2040-803X"[IS] OR "1439-4227"[IS] OR "1439-7633"[IS] OR "1741-2684"[IS] OR "1471-3012"[IS] OR "0940-6719"[IS] OR "1432-0932"[IS] OR "0790-9667"[IS] OR "0790-9667"[IS] OR "2451-9049"[IS] OR "2451-9049"[IS] OR "2053-2733"[IS] OR "2053-2733"[IS] OR "1362-3001"[IS] OR "0144-929X"[IS] OR "1365-2125"[IS] OR "0306-5251"[IS] OR "0269-9931"[IS] OR "1464-0600"[IS] OR "1744-5175"[IS] OR "0899-3408"[IS] OR "2095-3119"[IS] OR "2095-3119"[IS] OR "1590-9921"[IS] OR "1590-9999"[IS] OR "2590-0595"[IS] OR "2590-0595"[IS] OR "2057-2107"[IS] OR "2057-2107"[IS] OR "0141-3910"[IS] OR "0141-3910"[IS] OR "1618-0984"[IS] OR "0723-2020"[IS] OR "1470-1294"[IS] OR "1356-2517"[IS] OR "1472-6947"[IS] OR "1472-6947"[IS] OR "1757-4749"[IS] OR "1757-4749"[IS] OR "1095-9289"[IS] OR "1054-3139"[IS] OR "1557-9999"[IS] OR "1063-8210"[IS] OR "0147-7307"[IS] OR "1573-661X"[IS] OR "0305-6244"[IS] OR "1740-1720"[IS] OR "2166-2525"[IS] OR "2166-2525"[IS] OR "0960-3174"[IS] OR "1573-1375"[IS] OR "0363-7751"[IS] OR "1479-5787"[IS] OR "1863-5407"[IS] OR "1613-4796"[IS] OR "1872-7727"[IS] OR "0720-048X"[IS] OR "2513-843X"[IS] OR "2513-843X"[IS] OR "1099-1085"[IS] OR "0885-6087"[IS] OR "1541-4167"[IS] OR "1521-0251"[IS] OR "2053-9711"[IS] OR "2053-9711"[IS] OR "0145-9740"[IS] OR "1545-5882"[IS] OR "1354-8506"[IS] OR "1465-3966"[IS] OR "2095-4697"[IS] OR "2095-4689"[IS] OR "1460-3659"[IS] OR "0306-3127"[IS] OR "2666-3376"[IS] OR "2666-3376"[IS] OR "0832-610X"[IS] OR "1496-8975"[IS] OR "2190-5479"[IS] OR "2190-5452"[IS] OR "1365-2702"[IS] OR "0962-1067"[IS] OR "1382-6689"[IS] OR "1382-6689"[IS] OR "2631-6420"[IS] OR "2631-6439"[IS] OR "1552-6526"[IS] OR "0261-927X"[IS] OR "1042-444X"[IS] OR "1042-444X"[IS] OR "0893-3189"[IS] OR "0893-3189"[IS] OR "2641-0397"[IS] OR "2641-0397"[IS] OR "0144-5596"[IS] OR "1467-9515"[IS] OR "2153-2028"[IS] OR "1948-4682"[IS] OR "1866-9565"[IS] OR "1866-9557"[IS] OR "0967-0645"[IS] OR "0967-0645"[IS] OR "1756-932X"[IS] OR "1756-9338"[IS] OR "1045-9243"[IS] OR "1045-9243"[IS] OR "2469-7281"[IS] OR "2469-7281"[IS] OR "1553-5606"[IS] OR "1553-5592"[IS] OR "2150-1327"[IS] OR "2150-1319"[IS] OR "1558-8599"[IS] OR "1558-8602"[IS] OR "2077-0383"[IS] OR "2077-0383"[IS] OR "2093-4947"[IS] OR "1226-8453"[IS] OR "0964-2633"[IS] OR "1365-2788"[IS] OR "1931-7204"[IS] OR "1931-7204"[IS] OR "2632-3338"[IS] OR "2632-3338"[IS] OR "2213-3054"[IS] OR "2213-3054"[IS] OR "1976-8710"[IS] OR "2005-0720"[IS] OR "1600-0625"[IS] OR "0906-6705"[IS] OR "1654-9716"[IS] OR "1654-9880"[IS] OR "2193-3685"[IS] OR "2193-3677"[IS] OR "1527-5256"[IS] OR "1540-2347"[IS] OR "0031-8094"[IS] OR "1467-9213"[IS] OR "1533-4295"[IS] OR "1524-1602"[IS] OR "1110-0168"[IS] OR "1110-0168"[IS] OR "2522-8552"[IS] OR "2096-5524"[IS] OR "1053-8100"[IS] OR "1090-2376"[IS] OR "0014-4835"[IS] OR "1096-0007"[IS] OR "1579-1505"[IS] OR "1578-7303"[IS] OR "2210-8327"[IS] OR "2634-1964"[IS] OR "0925-4692"[IS] OR "1568-5608"[IS] OR "1553-118X"[IS] OR "1553-1198"[IS] OR "1867-111X"[IS] OR "1674-487X"[IS] OR "1758-3764"[IS] OR "1758-3756"[IS] OR "1612-3174"[IS] OR "1612-3174"[IS] OR "2409-515X"[IS] OR "2409-515X"[IS] OR "0095-4470"[IS] OR "1095-8576"[IS] OR "0273-5024"[IS] OR "1543-2769"[IS] OR "1539-6924"[IS] OR "0272-4332"[IS] OR "1556-1852"[IS] OR "0963-6412"[IS] OR "0248-4900"[IS] OR "1768-322X"[IS] OR "2296-4185"[IS] OR "2296-4185"[IS] OR "1874-5482"[IS] OR "1874-5482"[IS] OR "2040-4166"[IS] OR "2040-4174"[IS] OR "2213-333X"[IS] OR "2213-3348"[IS] OR "2058-9883"[IS] OR "2058-9883"[IS] OR "1477-7487"[IS] OR "1477-7487"[IS] OR "2050-3377"[IS] OR "2050-3369"[IS] OR "0749-0704"[IS] OR "1557-8232"[IS] OR "1120-8694"[IS] OR "1442-2050"[IS] OR "1754-9426"[IS] OR "1754-9434"[IS] OR "1059-0560"[IS] OR "1059-0560"[IS] OR "1532-7833"[IS] OR "1098-6065"[IS] OR "1543-8384"[IS] OR "1543-8392"[IS] OR "1520-586X"[IS] OR "1083-6160"[IS] OR "2321-3868"[IS] OR "2321-3876"[IS] OR "2397-0022"[IS] OR "2397-0030"[IS] OR "0020-4277"[IS] OR "1573-1952"[IS] OR "2057-3189"[IS] OR "2057-3170"[IS] OR "1365-232X"[IS] OR "0969-9988"[IS] OR "0016-9862"[IS] OR "1934-9041"[IS] OR "1097-0347"[IS] OR

	"1043-3074"[IS] OR "1828-0447"[IS] OR "1828-0447"[IS] OR "0020-7292"[IS] OR "1879-3479"[IS] OR "2524-7859"[IS] OR "2096-6873"[IS] OR "1674-2370"[IS] OR "1674-2370"[IS] OR "0305-1862"[IS] OR "1365-2214"[IS] OR "2057-0481"[IS] OR "2057-0481"[IS] OR "2296-858X"[IS] OR "2296-858X"[IS] OR "2169-3536"[IS] OR "2169-3536"[IS] OR "0197-2243"[IS] OR "1087-6537"[IS] OR "1469-8013"[IS] OR "0047-4045"[IS] OR "1072-0502"[IS] OR "1549-5485"[IS] OR "2363-9520"[IS] OR "2363-9512"[IS] OR "1573-2983"[IS] OR "0269-4042"[IS] OR "1265-4906"[IS] OR "1248-9204"[IS] OR "1080-7039"[IS] OR "1549-7860"[IS] OR "2397-7000"[IS] OR "1939-7267"[IS] OR "1878-187X"[IS] OR "1077-7229"[IS] OR "1178-2005"[IS] OR "1176-9106"[IS] OR "2213-1248"[IS] OR "1319-1578"[IS] OR "1942-7905"[IS] OR "1942-7891"[IS] OR "0007-0955"[IS] OR "1464-3529"[IS] OR "1178-1998"[IS] OR "1176-9092"[IS] OR "1552-3918"[IS] OR "0163-2787"[IS] OR "0966-0410"[IS] OR "1365-2524"[IS] OR "1098-5522"[IS] OR "0019-9567"[IS] OR "2517-4843"[IS] OR "2517-4843"[IS] OR "1749-5687"[IS] OR "1749-5679"[IS] OR "2635-0114"[IS] OR "2635-0106"[IS] OR "1872-9126"[IS] OR "0003-6870"[IS] OR "1472-0213"[IS] OR "1472-0205"[IS] OR "1056-6163"[IS] OR "1538-2982"[IS] OR "1464-066X"[IS] OR "0020-7594"[IS] OR "1745-2627"[IS] OR "1350-4509"[IS] OR "1439-037X"[IS] OR "0931-2250"[IS] OR "2005-0399"[IS] OR "2005-0380"[IS] OR "1538-4764"[IS] OR "0022-4669"[IS] OR "2325-3444"[IS] OR "2326-7186"[IS] OR "1387-1811"[IS] OR "1387-1811"[IS] OR "1464-9357"[IS] OR "1470-000X"[IS] OR "1558-075X"[IS] OR "0146-0005"[IS] OR "1536-0083"[IS] OR "1535-3516"[IS] OR "2214-6628"[IS] OR "2214-6628"[IS] OR "1539-297X"[IS] OR "0014-1704"[IS] OR "2214-790X"[IS] OR "2214-790X"[IS] OR "2515-2947"[IS] OR "2515-2947"[IS] OR "0167-6687"[IS] OR "0167-6687"[IS] OR "1538-7151"[IS] OR "0277-1691"[IS] OR "1538-1927"[IS] OR "1538-1927"[IS] OR "0266-6138"[IS] OR "1532-3099"[IS] OR "2329-2237"[IS] OR "2329-2229"[IS] OR "0141-6359"[IS] OR "0141-6359"[IS] OR "0960-8974"[IS] OR "0960-8974"[IS] OR "1558-4488"[IS] OR "0270-9295"[IS] OR "0262-6667"[IS] OR "0262-6667"[IS] OR "0733-9399"[IS] OR "0733-9399"[IS] OR "2211-0356"[IS] OR "2211-0348"[IS] OR "1179-1578"[IS] OR "1179-1578"[IS] OR "2287-4690"[IS] OR "2287-4208"[IS] OR "1873-5339"[IS] OR "0965-9978"[IS] OR "1435-9448"[IS] OR "1435-9456"[IS] OR "2379-2957"[IS] OR "2379-2957"[IS] OR "1470-2118"[IS] OR "1473-4893"[IS] OR "2169-575X"[IS] OR "2169-575X"[IS] OR "2474-8307"[IS] OR "2474-8307"[IS] OR "1469-0756"[IS] OR "0032-5473"[IS] OR "0168-0072"[IS] OR "0168-0072"[IS] OR "1472-7234"[IS] OR "0007-4918"[IS] OR "1463-7154"[IS] OR "1463-7154"[IS] OR "0272-8842"[IS] OR "0272-8842"[IS] OR "2666-8211"[IS] OR "2666-8211"[IS] OR "2665-9271"[IS] OR "2665-9271"[IS] OR "2045-7758"[IS] OR "2045-7758"[IS] OR "1557-900X"[IS] OR "0892-7790"[IS] OR "1098-3007"[IS] OR "1538-4772"[IS] OR "1475-2743"[IS] OR "0266-0032"[IS] OR "2662-1738"[IS] OR "2096-6326"[IS] OR "0003-4916"[IS] OR "1096-035X"[IS] OR "1570-1611"[IS] OR "1875-6212"[IS] OR "1110-8665"[IS] OR "1110-8665"[IS] OR "2162-2256"[IS] OR "2162-2248"[IS] OR "1557-7023"[IS] OR "1540-7063"[IS] OR "1975-5937"[IS] OR "1975-5937"[IS] OR "1558-8033"[IS] OR "1049-2127"[IS] OR "1088-4963"[IS] OR "0048-3915"[IS] OR "2214-157X"[IS] OR "2214-157X"[IS] OR "1612-9202"[IS] OR "1612-9202"[IS] OR "1469-5081"[IS] OR "0016-7568"[IS] OR "1473-5903"[IS] OR "1747-762X"[IS] OR "0035-9254"[IS] OR "1467-9876"[IS] OR "0033-2828"[IS] OR "2167-4086"[IS] OR "0952-0767"[IS] OR "1749-4192"[IS] OR "0741-0883"[IS] OR "0741-0883"[IS] OR "1574-9541"[IS] OR "1574-9541"[IS] OR "0160-0176"[IS] OR "1552-6925"[IS] OR "2772-3747"[IS] OR "2772-3747"[IS] OR "0169-7722"[IS] OR "1873-6009"[IS] OR "1436-5073"[IS] OR "0026-3672"[IS] OR "0882-7508"[IS] OR "1547-7401"[IS] OR "2666-5239"[IS] OR "2666-5239"[IS] OR "1472-6920"[IS] OR "1472-6920"[IS] OR "0016-6987"[IS] OR "2035-5556"[IS] OR "1562-3599"[IS] OR "1562-3599"[IS] OR "0264-3707"[IS] OR "0264-3707"[IS] OR "0148-5598"[IS] OR "1573-689X"[IS] OR "1873-3166"[IS] OR "0167-7322"[IS] OR "2056-306X"[IS] OR "2056-306X"[IS] OR "1756-0381"[IS] OR "1756-0381"[IS] OR "2397-3269"[IS] OR "2397-3269"[IS] OR "1573-7241"[IS] OR "0920-3206"[IS] OR "1465-3362"[IS] OR "0959-5236"[IS] OR "1751-7192"[IS] OR "1751-8253"[IS] OR "2329-9185"[IS] OR "2329-9185"[IS] OR "0309-2402"[IS] OR "1365-2648"[IS] OR "1048-6682"[IS] OR "1542-7854"[IS] OR "1070-5325"[IS] OR "1099-1506"[IS] OR "0964-1726"[IS] OR "1361-665X"[IS] OR "0191-491X"[IS] OR "0191-491X"[IS] OR "0375-6505"[IS] OR "0375-6505"[IS] OR "1751-8644"[IS] OR "1751-8652"[IS] OR "1479-2931"[IS] OR "1479-2931"[IS] OR "2589-1529"[IS] OR "2589-1529"[IS] OR "1478-3363"[IS] OR "1478-3371"[IS] OR "1936-7406"[IS] OR "1936-7414"[IS] OR "1758-5872"[IS] OR "1758-5864"[IS] OR "1860-7314"[IS] OR "1860-6768"[IS] OR "0007-1145"[IS] OR "1475-2662"[IS] OR "0091-5521"[IS] OR "0091-5521"[IS] OR "1871-5656"[IS] OR "1871-5621"[IS] OR "2196-5641"[IS] OR "2196-5641"[IS] OR "1432-6981"[IS] OR "1436-3771"[IS] OR "1744-8352"[IS] OR "1473-7159"[IS] OR "1367-8779"[IS] OR "1367-8779"[IS] OR "0958-0611"[IS] OR "2213-4368"[IS] OR "1471-1842"[IS] OR "1471-1834"[IS] OR "1053-0479"[IS] OR "1053-0479"[IS] OR "0078-0421"[IS] OR "0078-0421"[IS] OR "8755-6863"[IS] OR "1099-0496"[IS] OR "1939-1536"[IS] OR "0033-3204"[IS] OR "1054-8807"[IS] OR "1879-1336"[IS] OR "1573-3343"[IS] OR "0091-1674"[IS] OR "2641-435X"[IS] OR "2096-7004"[IS] OR "2154-1671"[IS] OR "2154-1663"[IS] OR "1097-0037"[IS] OR "0028-3045"[IS] OR "2632-3524"[IS] OR "2632-3524"[IS] OR "0340-5443"[IS] OR "1432-0762"[IS] OR "1573-6768"[IS] OR "1389-5729"[IS] OR "1472-684X"[IS] OR "1472-684X"[IS] OR "1754-2413"[IS] OR "1754-2413"[IS] OR "1010-061X"[IS] OR "1420-9101"[IS] OR "2096-7187"[IS] OR

	"2096-7187"[IS] OR "0197-5897"[IS] OR "1745-655X"[IS] OR "1353-5773"[IS] OR "1365-2095"[IS] OR "1523-536X"[IS] OR "0730-7659"[IS] OR "2050-7283"[IS] OR "2050-7283"[IS] OR "0146-2806"[IS] OR "1535-6280"[IS] OR "2474-7882"[IS] OR "2474-7882"[IS] OR "1873-3476"[IS] OR "0378-5173"[IS] OR "1080-6954"[IS] OR "1555-7898"[IS] OR "1477-7835"[IS] OR "1477-7835"[IS] OR "1529-8868"[IS] OR "1529-8868"[IS] OR "0957-1787"[IS] OR "0957-1787"[IS] OR "2213-7459"[IS] OR "2213-7459"[IS] OR "1946-6226"[IS] OR "1946-6226"[IS] OR "0144-686X"[IS] OR "1469-1779"[IS] OR "0887-0624"[IS] OR "1520-5029"[IS] OR "2444-8451"[IS] OR "2444-8494"[IS] OR "1311-0454"[IS] OR "1314-2224"[IS] OR "1833-2595"[IS] OR "1833-2595"[IS] OR "2198-0810"[IS] OR "2288-6206"[IS] OR "1868-5145"[IS] OR "1868-5137"[IS] OR "1369-1635"[IS] OR "0953-7104"[IS] OR "1558-4623"[IS] OR "0001-2998"[IS] OR "2005-4408"[IS] OR "2005-291X"[IS] OR "2003-0177"[IS] OR "2002-0244"[IS] OR "1469-9567"[IS] OR "1356-1820"[IS] OR "2376-6964"[IS] OR "2376-6972"[IS] OR "1350-6285"[IS] OR "1464-0716"[IS] OR "0883-2919"[IS] OR "0883-2919"[IS] OR "1802-7962"[IS] OR "1802-7962"[IS] OR "0143-831X"[IS] OR "1461-7099"[IS] OR "1868-6982"[IS] OR "1868-6974"[IS] OR "0721-7595"[IS] OR "1435-8107"[IS] OR "1751-732X"[IS] OR "1751-7311"[IS] OR "2051-1434"[IS] OR "2051-1434"[IS] OR "1936-2447"[IS] OR "1936-2455"[IS] OR "1447-2600"[IS] OR "1445-5226"[IS] OR "0278-4165"[IS] OR "1090-2686"[IS] OR "0163-3864"[IS] OR "1520-6025"[IS] OR "2288-5919"[IS] OR "2288-5943"[IS] OR "0364-2313"[IS] OR "1432-2323"[IS] OR "0963-5483"[IS] OR "1469-2163"[IS] OR "1473-6543"[IS] OR "1062-4821"[IS] OR "1934-5739"[IS] OR "1934-5747"[IS] OR "1043-9463"[IS] OR "1043-9463"[IS] OR "1862-8508"[IS] OR "1862-8516"[IS] OR "1095-7146"[IS] OR "0895-4801"[IS] OR "1552-8499"[IS] OR "0044-118X"[IS] OR "1948-5875"[IS] OR "1948-5875"[IS] OR "0145-4455"[IS] OR "1552-4167"[IS] OR "1464-0627"[IS] OR "0264-3294"[IS] OR "2666-7657"[IS] OR "2666-7657"[IS] OR "2213-3291"[IS] OR "2213-3291"[IS] OR "0018-7208"[IS] OR "1547-8181"[IS] OR "1526-484X"[IS] OR "1543-2742"[IS] OR "1945-2020"[IS] OR "1945-2020"[IS] OR "1614-7480"[IS] OR "1439-0108"[IS] OR "0957-1736"[IS] OR "0957-1736"[IS] OR "0894-4105"[IS] OR "1931-1559"[IS] OR "1469-3518"[IS] OR "0141-1926"[IS] OR "2055-5822"[IS] OR "2055-5822"[IS] OR "2399-4908"[IS] OR "2399-4908"[IS] OR "0099-1333"[IS] OR "0099-1333"[IS] OR "1360-0559"[IS] OR "0964-0568"[IS] OR "2314-7156"[IS] OR "2314-8861"[IS] OR "1549-9634"[IS] OR "1549-9642"[IS] OR "0036-1399"[IS] OR "1095-712X"[IS] OR "1864-5755"[IS] OR "1864-5755"[IS] OR "2156-8952"[IS] OR "2156-8944"[IS] OR "0965-254X"[IS] OR "1466-4488"[IS] OR "1878-3449"[IS] OR "0749-2081"[IS] OR "0957-8765"[IS] OR "1573-7888"[IS] OR "1651-2057"[IS] OR "0001-5555"[IS] OR "1058-0360"[IS] OR "1558-9110"[IS] OR "0095-4616"[IS] OR "1432-0606"[IS] OR "2227-9059"[IS] OR "2227-9059"[IS] OR "0098-1354"[IS] OR "0098-1354"[IS] OR "0142-5455"[IS] OR "0142-5455"[IS] OR "2057-5637"[IS] OR "2057-5645"[IS] OR "2164-1846"[IS] OR "2164-1862"[IS] OR "0926-6690"[IS] OR "0926-6690"[IS] OR "1387-6988"[IS] OR "1572-5448"[IS] OR "1057-0829"[IS] OR "1536-481X"[IS] OR "2155-7470"[IS] OR "2155-7470"[IS] OR "2043-0809"[IS] OR "2043-0795"[IS] OR "1617-416X"[IS] OR "1617-416X"[IS] OR "0048-7554"[IS] OR "2191-0308"[IS] OR "2471-2566"[IS] OR "2471-2574"[IS] OR "1432-0584"[IS] OR "0939-5555"[IS] OR "1879-114X"[IS] OR "0149-2918"[IS] OR "0040-0912"[IS] OR "0040-0912"[IS] OR "0253-9993"[IS] OR "0253-9993"[IS] OR "0300-9084"[IS] OR "6183-1638"[IS] OR "1090-2112"[IS] OR "1049-9644"[IS] OR "2590-2903"[IS] OR "2590-2903"[IS] OR "1742-5255"[IS] OR "1742-5255"[IS] OR "2578-4226"[IS] OR "2578-4218"[IS] OR "1877-9603"[IS] OR "1877-959X"[IS] OR "2212-3717"[IS] OR "2212-3717"[IS] OR "2340-9444"[IS] OR "2340-9436"[IS] OR "2515-7620"[IS] OR "2515-7620"[IS] OR "2330-2674"[IS] OR "2330-2682"[IS] OR "0021-5155"[IS] OR "1613-2246"[IS] OR "1756-4646"[IS] OR "1756-4646"[IS] OR "1557-2625"[IS] OR "1558-7118"[IS] OR "2396-8370"[IS] OR "2396-8370"[IS] OR "1355-2198"[IS] OR "1879-2502"[IS] OR "1661-8734"[IS] OR "1661-8726"[IS] OR "0920-4741"[IS] OR "1573-1650"[IS] OR "2162-2523"[IS] OR "2162-2515"[IS] OR "1356-3890"[IS] OR "1461-7153"[IS] OR "2197-1714"[IS] OR "2197-1714"[IS] OR "2095-6037"[IS] OR "2198-0934"[IS] OR "0957-4158"[IS] OR "0957-4158"[IS] OR "1006-4982"[IS] OR "1995-8196"[IS] OR "1754-5048"[IS] OR "1754-5048"[IS] OR "1520-6904"[IS] OR "0022-3263"[IS] OR "1876-1070"[IS] OR "1876-1070"[IS] OR "0340-6253"[IS] OR "0340-6253"[IS] OR "2168-0582"[IS] OR "2168-0566"[IS] OR "1060-0280"[IS] OR "1542-6270"[IS] OR "0009-2509"[IS] OR "0009-2509"[IS] OR "1472-8214"[IS] OR "1744-7623"[IS] OR "1755-4543"[IS] OR "1755-4535"[IS] OR "0160-5682"[IS] OR "1476-9360"[IS] OR "1572-9249"[IS] OR "1380-7870"[IS] OR "2211-9264"[IS] OR "2211-9264"[IS] OR "2168-6602"[IS] OR "0890-1171"[IS] OR "0016-3287"[IS] OR "0016-3287"[IS] OR "1437-3254"[IS] OR "1437-3262"[IS] OR "2666-3287"[IS] OR "2666-3287"[IS] OR "1553-7250"[IS] OR "1553-7250"[IS] OR "1573-3688"[IS] OR "1053-0487"[IS] OR "0827-6331"[IS] OR "2169-2610"[IS] OR "1029-4945"[IS] OR "0233-1934"[IS] OR "2167-8375"[IS] OR "2167-8383"[IS] OR "2090-4479"[IS] OR "2090-4479"[IS] OR "2475-2991"[IS] OR "2475-2991"[IS] OR "1872-7026"[IS] OR "0304-3800"[IS] OR "2589-5486"[IS] OR "2589-5486"[IS] OR "1793-6861"[IS] OR "0219-5305"[IS] OR "0340-3696"[IS] OR "1432-069X"[IS] OR "1874-8988"[IS] OR "1874-897X"[IS] OR "1177-8881"[IS] OR "1177-8881"[IS] OR "1164-5563"[IS] OR "1164-5563"[IS] OR "0022-4049"[IS] OR "0022-4049"[IS] OR "1651-2081"[IS] OR "1650-1977"[IS] OR "1469-7823"[IS] OR "0047-2794"[IS] OR "1537-4505"[IS] OR "1531-7129"[IS] OR "2352-0124"[IS] OR "2352-0124"[IS] OR "1432-0703"[IS] OR

	"0090-4341"[IS] OR "1469-5405"[IS] OR "1741-2900"[IS] OR "1745-526X"[IS] OR "0033-6882"[IS] OR "1532-2130"[IS] OR "1090-3798"[IS] OR "2335-0164"[IS] OR "0354-2025"[IS] OR "1932-6203"[IS] OR "1932-6203"[IS] OR "1573-7829"[IS] OR "0167-5923"[IS] OR "1439-099X"[IS] OR "0179-7158"[IS] OR "1029-3132"[IS] OR "1029-3132"[IS] OR "2667-0747"[IS] OR "2667-0747"[IS] OR "1723-2007"[IS] OR "1723-2007"[IS] OR "1465-3346"[IS] OR "0142-5692"[IS] OR "1573-2789"[IS] OR "0010-3853"[IS] OR "0014-3057"[IS] OR "0014-3057"[IS] OR "0901-5027"[IS] OR "0901-5027"[IS] OR "1557-0584"[IS] OR "1557-0576"[IS] OR "2770-3371"[IS] OR "2770-338X"[IS] OR "0167-6636"[IS] OR "0167-6636"[IS] OR "0167-8507"[IS] OR "1613-3684"[IS] OR "1527-8018"[IS] OR "0899-2363"[IS] OR "0951-5070"[IS] OR "1469-3674"[IS] OR "2471-1411"[IS] OR "2096-1790"[IS] OR "2632-8925"[IS] OR "2632-8925"[IS] OR "1976-9148"[IS] OR "2005-4483"[IS] OR "1340-6868"[IS] OR "1340-6868"[IS] OR "1868-6958"[IS] OR "1868-6966"[IS] OR "1364-5498"[IS] OR "1364-5498"[IS] OR "0570-4928"[IS] OR "1520-569X"[IS] OR "1872-8111"[IS] OR "0168-0102"[IS] OR "1532-8171"[IS] OR "0735-6757"[IS] OR "2041-2851"[IS] OR "2041-2851"[IS] OR "1755-3091"[IS] OR "1755-3091"[IS] OR "8756-758X"[IS] OR "1460-2695"[IS] OR "2296-6463"[IS] OR "2296-6463"[IS] OR "0047-2336"[IS] OR "1752-7554"[IS] OR "2041-1006"[IS] OR "2041-1014"[IS] OR "1849-5435"[IS] OR "1849-5435"[IS] OR "1466-853X"[IS] OR "1873-1600"[IS] OR "2352-9385"[IS] OR "2352-9385"[IS] OR "2075-4663"[IS] OR "2075-4663"[IS] OR "0037-9409"[IS] OR "0037-9409"[IS] OR "1309-4297"[IS] OR "2147-4281"[IS] OR "2571-581X"[IS] OR "2571-581X"[IS] OR "1532-7590"[IS] OR "1044-7318"[IS] OR "0176-1617"[IS] OR "1618-1328"[IS] OR "2191-9097"[IS] OR "2191-9089"[IS] OR "1092-9126"[IS] OR "1876-4665"[IS] OR "1943-7684"[IS] OR "0031-949X"[IS] OR "1520-5010"[IS] OR "0893-228X"[IS] OR "0010-2180"[IS] OR "0010-2180"[IS] OR "0376-8929"[IS] OR "1469-4387"[IS] OR "1448-5516"[IS] OR "1049-8001"[IS] OR "1931-3160"[IS] OR "1931-3152"[IS] OR "0219-0613"[IS] OR "0219-0613"[IS] OR "1365-2842"[IS] OR "0305-182X"[IS] OR "1085-9489"[IS] OR "1529-8027"[IS] OR "1472-0825"[IS] OR "1468-1811"[IS] OR "0167-9473"[IS] OR "0167-9473"[IS] OR "0379-7112"[IS] OR "0379-7112"[IS] OR "1120-1797"[IS] OR "1724-191X"[IS] OR "1747-0218"[IS] OR "1747-0226"[IS] OR "2093-7997"[IS] OR "2093-7911"[IS] OR "1388-1957"[IS] OR "1388-1957"[IS] OR "1439-6319"[IS] OR "1439-6327"[IS] OR "1743-7288"[IS] OR "1743-727X"[IS] OR "0950-0782"[IS] OR "0950-0782"[IS] OR "1573-1596"[IS] OR "1381-2386"[IS] OR "1741-3036"[IS] OR "0027-9501"[IS] OR "1476-8305"[IS] OR "1028-415X"[IS] OR "2353-1827"[IS] OR "2083-1277"[IS] OR "1873-5150"[IS] OR "0887-8994"[IS] OR "1539-1663"[IS] OR "1539-1663"[IS] OR "1532-4818"[IS] OR "0895-7347"[IS] OR "2515-2009"[IS] OR "2515-1991"[IS] OR "1920-7476"[IS] OR "0008-4263"[IS] OR "1095-8355"[IS] OR "1065-6995"[IS] OR "1943-5819"[IS] OR "0146-4833"[IS] OR "1573-076X"[IS] OR "0165-005X"[IS] OR "2055-0464"[IS] OR "2055-0464"[IS] OR "2296-861X"[IS] OR "2296-861X"[IS] OR "2517-5025"[IS] OR "2517-5025"[IS] OR "1879-1573"[IS] OR "0924-7963"[IS] OR "1003-6326"[IS] OR "2210-3384"[IS] OR "1606-6359"[IS] OR "1476-7392"[IS] OR "0214-4840"[IS] OR "0214-4840"[IS] OR "2513-0390"[IS] OR "2513-0390"[IS] OR "1477-0873"[IS] OR "0269-2155"[IS] OR "1710-3568"[IS] OR "1710-3568"[IS] OR "1744-8301"[IS] OR "1479-6694"[IS] OR "1360-0648"[IS] OR "0790-0627"[IS] OR "0021-8294"[IS] OR "0021-8294"[IS] OR "2043-6289"[IS] OR "1753-1934"[IS] OR "1937-2817"[IS] OR "0022-541X"[IS] OR "0030-3992"[IS] OR "0030-3992"[IS] OR "0029-6651"[IS] OR "1475-2719"[IS] OR "1475-2727"[IS] OR "1368-9800"[IS] OR "1078-1439"[IS] OR "1873-2496"[IS] OR "1556-8253"[IS] OR "1556-8342"[IS] OR "0009-2673"[IS] OR "1348-0634"[IS] OR "1360-4813"[IS] OR "1470-3629"[IS] OR "2234-1315"[IS] OR "1976-0485"[IS] OR "1090-4018"[IS] OR "1932-586X"[IS] OR "0885-2545"[IS] OR "0885-2545"[IS] OR "0022-3468"[IS] OR "1531-5037"[IS] OR "1469-672X"[IS] OR "0264-6838"[IS] OR "1749-818X"[IS] OR "1749-818X"[IS] OR "2214-7160"[IS] OR "2214-7160"[IS] OR "2518-0231"[IS] OR "2518-0231"[IS] OR "1440-169X"[IS] OR "0012-1592"[IS] OR "2212-4306"[IS] OR "2212-4292"[IS] OR "1873-2682"[IS] OR "1011-1344"[IS] OR "2213-8463"[IS] OR "2213-8463"[IS] OR "1042-8275"[IS] OR "1042-8275"[IS] OR "1699-2407"[IS] OR "1386-6710"[IS] OR "1814-1412"[IS] OR "1562-2975"[IS] OR "2661-8877"[IS] OR "2661-8869"[IS] OR "1751-2433"[IS] OR "1751-2441"[IS] OR "2046-1402"[IS] OR "2046-1402"[IS] OR "1949-1247"[IS] OR "1949-1255"[IS] OR "2153-2974"[IS] OR "2332-7383"[IS] OR "2309-608X"[IS] OR "2309-608X"[IS] OR "1880-5981"[IS] OR "1343-8832"[IS] OR "2159-9165"[IS] OR "2159-9173"[IS] OR "2211-8179"[IS] OR "2211-8160"[IS] OR "1431-2174"[IS] OR "1435-0157"[IS] OR "0022-4391"[IS] OR "1746-1561"[IS] OR "1742-7150"[IS] OR "1742-7169"[IS] OR "1558-1349"[IS] OR "1042-3680"[IS] OR "0214-1493"[IS] OR "0214-1493"[IS] OR "2072-6651"[IS] OR "2072-6651"[IS] OR "1532-3382"[IS] OR "1532-3390"[IS] OR "2224-2708"[IS] OR "2224-2708"[IS] OR "1072-3714"[IS] OR "1530-0374"[IS] OR "0889-8545"[IS] OR "0889-8545"[IS] OR "2056-2802"[IS] OR "2056-2802"[IS] OR "2210-5379"[IS] OR "2210-5379"[IS] OR "0928-4249"[IS] OR "1297-9716"[IS] OR "0275-2565"[IS] OR "1098-2345"[IS] OR "0927-7765"[IS] OR "1873-4367"[IS] OR "0250-832X"[IS] OR "1476-542X"[IS] OR "1573-7594"[IS] OR "0924-6703"[IS] OR "1479-0718"[IS] OR "1479-0718"[IS] OR "0277-2116"[IS] OR "1536-4801"[IS] OR "2587-0130"[IS] OR "2587-0130"[IS] OR "0167-482X"[IS] OR "1743-8942"[IS] OR "1664-039X"[IS] OR "1664-0403"[IS] OR "1520-6041"[IS] OR "0276-7333"[IS] OR "1354-0793"[IS] OR "1354-0793"[IS] OR "2210-7797"[IS] OR "2210-7789"[IS] OR "0003-9861"[IS] OR "1096-0384"[IS] OR "2363-8427"[IS] OR

	"2363-8419"[IS] OR "1092-4388"[IS] OR "1558-9102"[IS] OR "1878-7789"[IS] OR "1878-7789"[IS] OR "1539-767X"[IS] OR "0031-8248"[IS] OR "1947-5691"[IS] OR "1947-5683"[IS] OR "0009-2797"[IS] OR "0009-2797"[IS] OR "1749-4877"[IS] OR "1749-4877"[IS] OR "2632-8682"[IS] OR "2632-8682"[IS] OR "1864-9335"[IS] OR "2151-2590"[IS] OR "0734-242X"[IS] OR "1096-3669"[IS] OR "1550-7416"[IS] OR "1550-7416"[IS] OR "1573-1464"[IS] OR "1387-3547"[IS] OR "2199-692X"[IS] OR "2199-692X"[IS] OR "1170-229X"[IS] OR "1179-1969"[IS] OR "0890-3344"[IS] OR "1552-5732"[IS] OR "1938-7849"[IS] OR "1938-7849"[IS] OR "1879-0291"[IS] OR "0141-1136"[IS] OR "2183-2439"[IS] OR "2183-2439"[IS] OR "1086-9379"[IS] OR "1086-9379"[IS] OR "0031-9155"[IS] OR "1361-6560"[IS] OR "0164-2472"[IS] OR "1527-1951"[IS] OR "2468-2039"[IS] OR "2468-2039"[IS] OR "2328-7047"[IS] OR "2328-7055"[IS] OR "2156-4574"[IS] OR "2153-3660"[IS] OR "0003-682X"[IS] OR "1872-910X"[IS] OR "2405-8866"[IS] OR "2405-8866"[IS] OR "2576-3202"[IS] OR "2576-3202"[IS] OR "1748-2801"[IS] OR "1748-2798"[IS] OR "0031-8019"[IS] OR "0031-8019"[IS] OR "1941-1022"[IS] OR "1941-1022"[IS] OR "2666-691X"[IS] OR "2666-691X"[IS] OR "0375-5444"[IS] OR "0375-5444"[IS] OR "1537-2197"[IS] OR "0002-9122"[IS] OR "2398-3795"[IS] OR "2398-3795"[IS] OR "1045-5752"[IS] OR "1548-3290"[IS] OR "2325-0992"[IS] OR "2325-0984"[IS] OR "1435-5957"[IS] OR "1056-8190"[IS] OR "2633-2892"[IS] OR "2633-2892"[IS] OR "2617-4782"[IS] OR "2617-4782"[IS] OR "1618-954X"[IS] OR "1618-954X"[IS] OR "0889-311X"[IS] OR "0889-311X"[IS] OR "0364-152X"[IS] OR "1432-1009"[IS] OR "1026-9185"[IS] OR "1026-9185"[IS] OR "1556-9519"[IS] OR "1556-3650"[IS] OR "0894-1777"[IS] OR "0894-1777"[IS] OR "1878-4658"[IS] OR "0020-7187"[IS] OR "1573-0409"[IS] OR "0921-0296"[IS] OR "1062-726X"[IS] OR "1532-754X"[IS] OR "1744-6848"[IS] OR "1744-683X"[IS] OR "2162-268X"[IS] OR "2162-2671"[IS] OR "1545-102X"[IS] OR "1741-6787"[IS] OR "1559-2731"[IS] OR "1559-2723"[IS] OR "1525-142X"[IS] OR "1520-541X"[IS] OR "0017-467X"[IS] OR "1745-6584"[IS] OR "0033-5894"[IS] OR "0033-5894"[IS] OR "1877-0541"[IS] OR "1877-0533"[IS] OR "1438-1656"[IS] OR "1527-2648"[IS] OR "0306-4549"[IS] OR "1873-2100"[IS] OR "2056-807X"[IS] OR "2056-807X"[IS] OR "1466-4348"[IS] OR "0950-2386"[IS] OR "1934-2845"[IS] OR "0071-3260"[IS] OR "1573-0638"[IS] OR "0020-8566"[IS] OR "0171-8630"[IS] OR "1616-1599"[IS] OR "2373-8065"[IS] OR "2373-8065"[IS] OR "2694-2518"[IS] OR "2694-2518"[IS] OR "2308-5835"[IS] OR "2308-5827"[IS] OR "1750-9653"[IS] OR "1750-9661"[IS] OR "2093-2685"[IS] OR "2325-4483"[IS] OR "1539-2791"[IS] OR "1559-0089"[IS] OR "0032-4728"[IS] OR "1477-4747"[IS] OR "0162-0436"[IS] OR "1573-7837"[IS] OR "0898-1221"[IS] OR "0898-1221"[IS] OR "0937-4477"[IS] OR "1434-4726"[IS] OR "2405-6766"[IS] OR "2405-6766"[IS] OR "1046-560X"[IS] OR "1046-560X"[IS] OR "2571-8789"[IS] OR "2571-8789"[IS] OR "1542-7625"[IS] OR "1057-2252"[IS] OR "2694-6424"[IS] OR "2694-6416"[IS] OR "0890-6327"[IS] OR "1099-1115"[IS] OR "2509-4696"[IS] OR "2096-241X"[IS] OR "2005-7563"[IS] OR "2005-6419"[IS] OR "1743-5390"[IS] OR "1743-5404"[IS] OR "2055-1010"[IS] OR "2055-1010"[IS] OR "1438-8677"[IS] OR "1435-8603"[IS] OR "1040-6182"[IS] OR "1040-6182"[IS] OR "2468-0230"[IS] OR "2468-0230"[IS] OR "2576-6422"[IS] OR "2576-6422"[IS] OR "1872-8006"[IS] OR "0304-4165"[IS] OR "1871-1502"[IS] OR "1871-1502"[IS] OR "1070-9878"[IS] OR "1070-9878"[IS] OR "2043-6882"[IS] OR "1465-7503"[IS] OR "1615-5270"[IS] OR "1615-5262"[IS] OR "1360-6441"[IS] OR "1467-9841"[IS] OR "1616-5195"[IS] OR "1616-5187"[IS] OR "1540-3459"[IS] OR "1540-3467"[IS] OR "1758-2245"[IS] OR "1520-8583"[IS] OR "0386-1112"[IS] OR "0386-1112"[IS] OR "1869-103X"[IS] OR "1674-4799"[IS] OR "1526-2375"[IS] OR "1526-4637"[IS] OR "1430-2772"[IS] OR "0340-0727"[IS] OR "2056-7529"[IS] OR "2056-7529"[IS] OR "1862-5347"[IS] OR "1862-5355"[IS] OR "1549-6511"[IS] OR "0195-6744"[IS] OR "2191-219X"[IS] OR "2191-219X"[IS] OR "1742-9994"[IS] OR "1742-9994"[IS] OR "1937-5395"[IS] OR "1937-5387"[IS] OR "1724-6059"[IS] OR "1121-8428"[IS] OR "0029-5493"[IS] OR "0029-5493"[IS] OR "1875-8355"[IS] OR "1572-3887"[IS] OR "1831-4732"[IS] OR "1831-4732"[IS] OR "0954-6634"[IS] OR "0954-6634"[IS] OR "1551-2916"[IS] OR "0002-7820"[IS] OR "0309-8168"[IS] OR "0309-8168"[IS] OR "1933-6969"[IS] OR "1933-6950"[IS] OR "1536-3724"[IS] OR "1050-642X"[IS] OR "0090-9556"[IS] OR "1521-009X"[IS] OR "1474-5151"[IS] OR "1474-5151"[IS] OR "2516-8398"[IS] OR "2516-8398"[IS] OR "2329-4884"[IS] OR "2329-4892"[IS] OR "1754-1611"[IS] OR "1754-1611"[IS] OR "0885-8624"[IS] OR "0885-8624"[IS] OR "1021-9498"[IS] OR "2224-6614"[IS] OR "1534-7362"[IS] OR "1534-7362"[IS] OR "0024-3795"[IS] OR "0024-3795"[IS] OR "2666-3511"[IS] OR "2666-3511"[IS] OR "1930-0212"[IS] OR "1094-6470"[IS] OR "0002-9610"[IS] OR "1879-1883"[IS] OR "2053-0196"[IS] OR "2053-020X"[IS] OR "1878-5352"[IS] OR "1878-5352"[IS] OR "1523-5882"[IS] OR "1523-5890"[IS] OR "1110-9823"[IS] OR "1110-9823"[IS] OR "0885-8985"[IS] OR "0885-8985"[IS] OR "2380-0844"[IS] OR "2380-0852"[IS] OR "2288-5048"[IS] OR "2288-4300"[IS] OR "1533-6077"[IS] OR "1758-2237"[IS] OR "0361-7734"[IS] OR "1559-2863"[IS] OR "1543-2750"[IS] OR "0033-6297"[IS] OR "1570-9639"[IS] OR "1878-1454"[IS] OR "1070-986X"[IS] OR "1070-986X"[IS] OR "0891-1762"[IS] OR "1528-6975"[IS] OR "1601-0825"[IS] OR "1354-523X"[IS] OR "0022-3239"[IS] OR "1573-2878"[IS] OR "1573-5079"[IS] OR "0166-8595"[IS] OR "1551-7411"[IS] OR "1551-7411"[IS] OR "2211-3711"[IS] OR "2211-372X"[IS] OR "1532-950X"[IS] OR "0161-3499"[IS] OR "2589-014X"[IS] OR "2589-014X"[IS] OR "1559-2030"[IS] OR "1551-7144"[IS] OR "0925-5001"[IS] OR "1573-2916"[IS] OR "1556-1828"[IS] OR "1556-4894"[IS] OR "1879-8500"[IS] OR

	"1879-8519"[IS] OR "1471-5945"[IS] OR "1471-5945"[IS] OR "2676-6957"[IS] OR "2476-3055"[IS] OR "1071-7544"[IS] OR "1521-0464"[IS] OR "0265-0215"[IS] OR "1365-2346"[IS] OR "1548-1387"[IS] OR "0745-5194"[IS] OR "0031-9406"[IS] OR "1873-1465"[IS] OR "1936-4954"[IS] OR "1936-4954"[IS] OR "0926-2040"[IS] OR "1527-3326"[IS] OR "0961-463X"[IS] OR "1461-7463"[IS] OR "2399-9772"[IS] OR "2399-9772"[IS] OR "2504-446X"[IS] OR "2504-446X"[IS] OR "1573-1707"[IS] OR "1082-3301"[IS] OR "0736-3761"[IS] OR "0736-3761"[IS] OR "1359-0987"[IS] OR "1359-0987"[IS] OR "0266-8920"[IS] OR "1878-4275"[IS] OR "1088-6826"[IS] OR "0002-9939"[IS] OR "1557-0851"[IS] OR "1557-086X"[IS] OR "0305-8719"[IS] OR "0305-8719"[IS] OR "0897-5264"[IS] OR "1543-3382"[IS] OR "1532-7701"[IS] OR "1534-8458"[IS] OR "2363-7625"[IS] OR "1001-6112"[IS] OR "1461-7374"[IS] OR "0143-0343"[IS] OR "0300-483X"[IS] OR "1879-3185"[IS] OR "1036-7314"[IS] OR "1036-7314"[IS] OR "0935-1175"[IS] OR "1432-0959"[IS] OR "1053-0819"[IS] OR "1573-3513"[IS] OR "0025-5408"[IS] OR "0025-5408"[IS] OR "2169-3277"[IS] OR "2169-3277"[IS] OR "1942-3454"[IS] OR "1942-3462"[IS] OR "2352-2267"[IS] OR "2352-2267"[IS] OR "1399-0012"[IS] OR "0902-0063"[IS] OR "1464-5351"[IS] OR "1369-1058"[IS] OR "2211-6958"[IS] OR "2211-6958"[IS] OR "0142-727X"[IS] OR "0142-727X"[IS] OR "1747-7611"[IS] OR "1038-2046"[IS] OR "1882-7616"[IS] OR "2213-6851"[IS] OR "1945-2365"[IS] OR "0026-2285"[IS] OR "1098-2426"[IS] OR "0749-159X"[IS] OR "1320-7881"[IS] OR "1440-1800"[IS] OR "2159-4260"[IS] OR "0001-4788"[IS] OR "1525-8955"[IS] OR "0885-3010"[IS] OR "1573-1804"[IS] OR "0957-7572"[IS] OR "2093-5382"[IS] OR "2093-5374"[IS] OR "1384-5640"[IS] OR "1573-272X"[IS] OR "2096-5362"[IS] OR "2524-4167"[IS] OR "2054-5703"[IS] OR "2054-5703"[IS] OR "0192-415X"[IS] OR "0192-415X"[IS] OR "2326-5108"[IS] OR "2326-5094"[IS] OR "1365-4802"[IS] OR "1475-7583"[IS] OR "2516-6034"[IS] OR "2516-6026"[IS] OR "1552-5449"[IS] OR "0272-4316"[IS] OR "0022-0655"[IS] OR "0022-0655"[IS] OR "1873-2364"[IS] OR "0960-8966"[IS] OR "2196-4386"[IS] OR "2196-4378"[IS] OR "1744-2648"[IS] OR "1744-2648"[IS] OR "1465-0045"[IS] OR "1465-0045"[IS] OR "1328-3154"[IS] OR "1328-3154"[IS] OR "0738-0593"[IS] OR "0738-0593"[IS] OR "1539-8412"[IS] OR "2152-0895"[IS] OR "0948-4280"[IS] OR "1437-8213"[IS] OR "1876-3413"[IS] OR "1876-3405"[IS] OR "1096-3367"[IS] OR "1945-1814"[IS] OR "1944-4079"[IS] OR "1944-4079"[IS] OR "0304-4238"[IS] OR "0304-4238"[IS] OR "1464-4177"[IS] OR "1751-7648"[IS] OR "1708-8569"[IS] OR "1867-0687"[IS] OR "1943-393X"[IS] OR "1943-3921"[IS] OR "2369-3762"[IS] OR "2369-3762"[IS] OR "2035-5114"[IS] OR "2035-5106"[IS] OR "1568-7759"[IS] OR "1568-7759"[IS] OR "1545-6846"[IS] OR "0037-8046"[IS] OR "2474-9508"[IS] OR "2474-9508"[IS] OR "1663-2826"[IS] OR "1663-2818"[IS] OR "1468-3148"[IS] OR "1360-2322"[IS] OR "0022-0418"[IS] OR "0022-0418"[IS] OR "1598-0316"[IS] OR "2158-1606"[IS] OR "1432-1432"[IS] OR "0022-2844"[IS] OR "1096-9098"[IS] OR "0022-4790"[IS] OR "1520-5827"[IS] OR "0743-7463"[IS] OR "1360-0508"[IS] OR "0968-7599"[IS] OR "1387-585X"[IS] OR "1573-2975"[IS] OR "2046-0430"[IS] OR "2046-0449"[IS] OR "1743-873X"[IS] OR "1747-6631"[IS] OR "2534-9708"[IS] OR "2534-9708"[IS] OR "0027-5514"[IS] OR "1557-2536"[IS] OR "2043-6866"[IS] OR "0030-7270"[IS] OR "1745-5863"[IS] OR "1745-5871"[IS] OR "0016-7428"[IS] OR "1931-0846"[IS] OR "2397-7604"[IS] OR "2397-7604"[IS] OR "1465-3915"[IS] OR "0305-4985"[IS] OR "1362-1718"[IS] OR "1743-2936"[IS] OR "1540-496X"[IS] OR "1540-496X"[IS] OR "0963-8024"[IS] OR "1464-3723"[IS] OR "0378-8741"[IS] OR "1872-7573"[IS] OR "2168-1023"[IS] OR "2168-1015"[IS] OR "1096-0813"[IS] OR "0022-247X"[IS] OR "1002-0721"[IS] OR "1002-0721"[IS] OR "1658-077X"[IS] OR "1658-077X"[IS] OR "1478-3967"[IS] OR "1478-3975"[IS] OR "2666-1888"[IS] OR "2666-1888"[IS] OR "1868-4297"[IS] OR "1868-4300"[IS] OR "0010-5422"[IS] OR "0010-5422"[IS] OR "1752-4040"[IS] OR "1752-4032"[IS] OR "1940-6940"[IS] OR "1940-6959"[IS] OR "0093-4690"[IS] OR "2042-4582"[IS] OR "0143-4632"[IS] OR "0143-4632"[IS] OR "2195-3325"[IS] OR "2195-3325"[IS] OR "0090-5917"[IS] OR "1552-7476"[IS] OR "1073-2322"[IS] OR "1540-0514"[IS] OR "1573-1960"[IS] OR "0042-0972"[IS] OR "1467-873X"[IS] OR "0362-6784"[IS] OR "0361-3666"[IS] OR "1467-7717"[IS] OR "2468-1709"[IS] OR "2468-1709"[IS] OR "1861-6410"[IS] OR "1861-6429"[IS] OR "2051-3305"[IS] OR "2051-3305"[IS] OR "1478-0941"[IS] OR "1477-2019"[IS] OR "1380-3743"[IS] OR "1572-9788"[IS] OR "1364-985X"[IS] OR "1467-8489"[IS] OR "0253-5068"[IS] OR "1421-9735"[IS] OR "2713-4148"[IS] OR "2713-4148"[IS] OR "0363-4523"[IS] OR "1479-5795"[IS] OR "2397-3129"[IS] OR "2397-3129"[IS] OR "1873-3441"[IS] OR "0939-6411"[IS] OR "1474-8185"[IS] OR "1474-8185"[IS] OR "1744-1374"[IS] OR "1744-1382"[IS] OR "0393-697X"[IS] OR "0393-697X"[IS] OR "0305-0041"[IS] OR "1469-8064"[IS] OR "1049-0965"[IS] OR "1537-5935"[IS] OR "0022-1376"[IS] OR "1537-5269"[IS] OR "1468-5248"[IS] OR "1468-5248"[IS] OR "1329-878X"[IS] OR "2200-467X"[IS] OR "1728-4465"[IS] OR "0039-3665"[IS] OR "2610-3540"[IS] OR "2610-3540"[IS] OR "2324-8386"[IS] OR "2324-8378"[IS] OR "1526-9523"[IS] OR "1526-9523"[IS] OR "1584-8574"[IS] OR "1584-8574"[IS] OR "1550-5030"[IS] OR "0361-6274"[IS] OR "0163-9641"[IS] OR "1097-0355"[IS] OR "1468-2389"[IS] OR "0965-075X"[IS] OR "1438-7573"[IS] OR "1525-3961"[IS] OR "1525-1403"[IS] OR "1094-7159"[IS] OR "1385-1314"[IS] OR "1385-1314"[IS] OR "1472-6815"[IS] OR "1472-6815"[IS] OR "1567-7257"[IS] OR "1567-1348"[IS] OR "1525-3163"[IS] OR "0021-8812"[IS] OR "1748-6815"[IS] OR "1748-6815"[IS] OR "1469-9893"[IS] OR "1461-3808"[IS] OR "1096-0333"[IS] OR "0041-008X"[IS] OR "1464-5173"[IS] OR "1360-3116"[IS] OR "0935-4964"[IS] OR

"1432-2250"[IS] OR "1472-4782"[IS] OR "1476-9328"[IS] OR "2046-6390"[IS] OR "2046-6390"[IS] OR
"2194-5411"[IS] OR "2194-5403"[IS] OR "0147-1767"[IS] OR "0147-1767"[IS] OR "1560-2281"[IS] OR
"1083-3668"[IS] OR "2053-4604"[IS] OR "2053-4612"[IS] OR "1539-2570"[IS] OR "0271-6798"[IS] OR
"0889-504X"[IS] OR "1528-8900"[IS] OR "1744-8069"[IS] OR "1744-8069"[IS] OR "2150-1149"[IS] OR
"1533-3159"[IS] OR "1463-9084"[IS] OR "1463-9076"[IS] OR "0976-2817"[IS] OR "0019-5049"[IS] OR
"1050-8619"[IS] OR "1050-8619"[IS] OR "0022-4898"[IS] OR "0022-4898"[IS] OR "1432-1920"[IS] OR
"0028-3940"[IS] OR "2151-2604"[IS] OR "2190-8370"[IS] OR "2214-8450"[IS] OR "2214-8469"[IS] OR
"1671-7694"[IS] OR "1671-7694"[IS] OR "0894-3257"[IS] OR "1099-0771"[IS] OR "1067-0564"[IS] OR
"1469-9400"[IS] OR "1943-4162"[IS] OR "1943-4170"[IS] OR "1476-7724"[IS] OR "1476-7724"[IS] OR
"1474-919X"[IS] OR "0019-1019"[IS] OR "2424-8002"[IS] OR "2424-8002"[IS] OR "1744-7429"[IS] OR
"0006-3606"[IS] OR "1054-1500"[IS] OR "1089-7682"[IS] OR "0094-1298"[IS] OR "1558-0504"[IS] OR
"1468-0777"[IS] OR "1468-0777"[IS] OR "8755-9129"[IS] OR "1525-6103"[IS] OR "0228-0671"[IS] OR
"0228-0671"[IS] OR "0021-8669"[IS] OR "0021-8669"[IS] OR "2211-9132"[IS] OR "2211-9140"[IS] OR
"1470-1170"[IS] OR "0031-3831"[IS] OR "0894-1920"[IS] OR "1521-0723"[IS] OR "1475-4762"[IS] OR
"0004-0894"[IS] OR "1548-159X"[IS] OR "2163-7873"[IS] OR "1520-5045"[IS] OR "0888-5885"[IS] OR
"1532-7698"[IS] OR "1526-7431"[IS] OR "1598-9623"[IS] OR "2005-4149"[IS] OR "2470-0045"[IS] OR
"2470-0053"[IS] OR "0005-2736"[IS] OR "1879-2642"[IS] OR "0010-1958"[IS] OR "0010-1958"[IS] OR
"1936-0584"[IS] OR "1936-0592"[IS] OR "1448-2517"[IS] OR "1448-2517"[IS] OR "1096-0945"[IS] OR
"0014-4800"[IS] OR "2689-3622"[IS] OR "2689-3614"[IS] OR "1464-0694"[IS] OR "0960-2011"[IS] OR
"1179-2019"[IS] OR "1174-5878"[IS] OR "1467-9523"[IS] OR "0038-0199"[IS] OR "1435-0661"[IS] OR
"0361-5995"[IS] OR "2055-4877"[IS] OR "2055-4877"[IS] OR "1873-6351"[IS] OR "0278-6915"[IS] OR
"1553-6610"[IS] OR "1868-9884"[IS] OR "1758-857X"[IS] OR "1747-1117"[IS] OR "1874-785X"[IS] OR
"1874-7868"[IS] OR "0706-652X"[IS] OR "1205-7533"[IS] OR "1741-3540"[IS] OR "0256-307X"[IS] OR
"1077-8926"[IS] OR "1097-1440"[IS] OR "1464-7001"[IS] OR "1464-7001"[IS] OR "1446-1242"[IS] OR
"1839-3551"[IS] OR "0925-9899"[IS] OR "1572-9192"[IS] OR "2381-2354"[IS] OR "2381-2346"[IS] OR
"1029-2330"[IS] OR "1061-186X"[IS] OR "1600-0765"[IS] OR "0022-3484"[IS] OR "1941-2460"[IS] OR
"0003-0651"[IS] OR "0954-3945"[IS] OR "0954-3945"[IS] OR "2254-4372"[IS] OR "1136-1034"[IS] OR
"2666-0539"[IS] OR "2666-0539"[IS] OR "1461-7471"[IS] OR "1363-4615"[IS] OR "1365-2311"[IS] OR
"0307-6946"[IS] OR "1558-1934"[IS] OR "1083-7515"[IS] OR "2352-0132"[IS] OR "2352-0132"[IS] OR
"1476-5535"[IS] OR "1367-5435"[IS] OR "1128-045X"[IS] OR "1123-6337"[IS] OR "0010-4485"[IS] OR
"0010-4485"[IS] OR "1569-1713"[IS] OR "1569-1713"[IS] OR "1475-6374"[IS] OR "1475-6366"[IS] OR
"1547-7355"[IS] OR "1547-7355"[IS] OR "1436-3259"[IS] OR "1436-3240"[IS] OR "2050-3385"[IS] OR
"2050-3385"[IS] OR "1545-2301"[IS] OR "0731-7115"[IS] OR "2468-2020"[IS] OR "2468-2020"[IS] OR
"1421-9883"[IS] OR "0253-4886"[IS] OR "1947-5713"[IS] OR "1947-5705"[IS] OR "1942-0684"[IS] OR
"1942-0676"[IS] OR "1660-3397"[IS] OR "1660-3397"[IS] OR "2150-1211"[IS] OR "2150-1203"[IS] OR
"1872-7506"[IS] OR "0925-4927"[IS] OR "1860-0743"[IS] OR "1435-1935"[IS] OR "2590-1370"[IS] OR
"2590-1370"[IS] OR "1682-0983"[IS] OR "1680-4333"[IS] OR "1932-1465"[IS] OR "1932-1473"[IS] OR
"2057-2093"[IS] OR "2057-2093"[IS] OR "1573-4803"[IS] OR "0022-2461"[IS] OR "1933-0707"[IS] OR
"1933-0715"[IS] OR "1469-8129"[IS] OR "1354-5078"[IS] OR "2218-6581"[IS] OR "2218-6581"[IS] OR
"1432-0843"[IS] OR "0344-5704"[IS] OR "0885-6257"[IS] OR "1469-591X"[IS] OR "2079-4991"[IS] OR
"2079-4991"[IS] OR "1468-1366"[IS] OR "1468-1366"[IS] OR "1573-1642"[IS] OR "1083-8155"[IS] OR
"1297-966X"[IS] OR "1286-4560"[IS] OR "2190-3948"[IS] OR "2190-393X"[IS] OR "2053-4477"[IS] OR
"2053-4485"[IS] OR "1748-3190"[IS] OR "1748-3182"[IS] OR "1472-6939"[IS] OR "1472-6939"[IS] OR
"1874-9372"[IS] OR "0006-8101"[IS] OR "2397-8554"[IS] OR "2397-8562"[IS] OR "0168-874X"[IS] OR
"0168-874X"[IS] OR "1873-7943"[IS] OR "0005-7916"[IS] OR "0022-2011"[IS] OR "1096-0805"[IS] OR
"1529-1774"[IS] OR "1087-0024"[IS] OR "1541-5856"[IS] OR "1541-5856"[IS] OR "1321-103X"[IS] OR
"1834-5530"[IS] OR "1541-7808"[IS] OR "1541-7794"[IS] OR "2352-3441"[IS] OR "2352-3441"[IS] OR
"2590-2520"[IS] OR "2590-2520"[IS] OR "1062-7197"[IS] OR "1532-6977"[IS] OR "0385-2407"[IS] OR
"0385-2407"[IS] OR "1098-2752"[IS] OR "0738-1085"[IS] OR "0344-5607"[IS] OR "1437-2320"[IS] OR
"0039-7857"[IS] OR "1573-0964"[IS] OR "1671-2234"[IS] OR "1671-2234"[IS] OR "1933-5954"[IS] OR
"1933-5954"[IS] OR "0967-0637"[IS] OR "0967-0637"[IS] OR "1099-114X"[IS] OR "0363-907X"[IS] OR
"0928-6586"[IS] OR "1744-5086"[IS] OR "0260-3055"[IS] OR "0260-3055"[IS] OR "1420-0597"[IS] OR
"1420-0597"[IS] OR "1469-3690"[IS] OR "0954-8963"[IS] OR "0197-6664"[IS] OR "1741-3729"[IS] OR
"2666-3309"[IS] OR "2666-3309"[IS] OR "1469-9664"[IS] OR "1357-4809"[IS] OR "0161-1461"[IS] OR
"1558-9129"[IS] OR "2352-3646"[IS] OR "2352-3646"[IS] OR "0030-3755"[IS] OR "1423-0267"[IS] OR
"2352-5134"[IS] OR "2352-5134"[IS] OR "1095-8339"[IS] OR "0024-4074"[IS] OR "0007-1005"[IS] OR
"1467-8527"[IS] OR "1534-0392"[IS] OR "1534-0392"[IS] OR "1350-6307"[IS] OR "1350-6307"[IS] OR
"2192-4406"[IS] OR "2192-4414"[IS] OR "2095-0233"[IS] OR "2095-0241"[IS] OR "1747-423X"[IS] OR
"1747-4248"[IS] OR "1938-3681"[IS] OR "1527-1404"[IS] OR "2352-507X"[IS] OR "2352-507X"[IS] OR
"1744-5078"[IS] OR "0927-3948"[IS] OR "1469-5812"[IS] OR "0013-1857"[IS] OR "0019-8676"[IS] OR

	"1468-232X"[IS] OR "1051-0443"[IS] OR "1535-7732"[IS] OR "0308-8839"[IS] OR "0308-8839"[IS] OR "1073-5615"[IS] OR "1543-1916"[IS] OR "1000-3673"[IS] OR "1000-3673"[IS] OR "1532-2971"[IS] OR "1090-0233"[IS] OR "1099-0755"[IS] OR "1052-7613"[IS] OR "0044-2372"[IS] OR "0044-2372"[IS] OR "0047-2425"[IS] OR "1537-2537"[IS] OR "1252-607X"[IS] OR "1776-1042"[IS] OR "1988-3218"[IS] OR "0082-5638"[IS] OR "1937-5093"[IS] OR "1937-5077"[IS] OR "0028-8306"[IS] OR "1175-8791"[IS] OR "1532-9488"[IS] OR "1043-0679"[IS] OR "1945-8932"[IS] OR "1945-8924"[IS] OR "1469-1817"[IS] OR "0142-7164"[IS] OR "0955-7571"[IS] OR "1474-449X"[IS] OR "0256-7679"[IS] OR "1439-6203"[IS] OR "1876-1399"[IS] OR "1876-1399"[IS] OR "2198-7505"[IS] OR "2198-7491"[IS] OR "1742-3600"[IS] OR "1742-3600"[IS] OR "0960-3085"[IS] OR "1744-3571"[IS] OR "2468-0451"[IS] OR "2468-0451"[IS] OR "1368-2822"[IS] OR "1460-6984"[IS] OR "1804-1728"[IS] OR "1804-171X"[IS] OR "0219-8916"[IS] OR "0219-8916"[IS] OR "2512-2819"[IS] OR "2512-2789"[IS] OR "8756-3894"[IS] OR "8756-3894"[IS] OR "2192-6506"[IS] OR "2192-6506"[IS] OR "1532-0456"[IS] OR "1878-1659"[IS] OR "0011-3204"[IS] OR "1537-5382"[IS] OR "0001-7701"[IS] OR "1572-9532"[IS] OR "1572-9591"[IS] OR "0164-0313"[IS] OR "2168-0418"[IS] OR "2168-0396"[IS] OR "2212-9790"[IS] OR "1872-7859"[IS] OR "0032-3861"[IS] OR "0032-3861"[IS] OR "2405-805X"[IS] OR "2405-805X"[IS] OR "1467-8373"[IS] OR "1360-7456"[IS] OR "1383-4517"[IS] OR "1383-4517"[IS] OR "2516-8401"[IS] OR "2516-8401"[IS] OR "1363-2469"[IS] OR "1559-808X"[IS] OR "1938-9027"[IS] OR "1526-6133"[IS] OR "1424-8247"[IS] OR "1424-8247"[IS] OR "1573-2711"[IS] OR "1023-8883"[IS] OR "0002-7294"[IS] OR "1548-1433"[IS] OR "1553-6467"[IS] OR "0002-9459"[IS] OR "1661-3791"[IS] OR "1661-3805"[IS] OR "2042-3306"[IS] OR "0425-1644"[IS] OR "2296-2360"[IS] OR "2296-2360"[IS] OR "1581-1271"[IS] OR "1408-2616"[IS] OR "0377-8401"[IS] OR "0377-8401"[IS] OR "1469-2945"[IS] OR "1359-866X"[IS] OR "2378-8763"[IS] OR "2378-8763"[IS] OR "1945-2837"[IS] OR "1945-2829"[IS] OR "1094-6136"[IS] OR "1094-6136"[IS] OR "1741-2978"[IS] OR "1440-7833"[IS] OR "0158-037X"[IS] OR "1470-126X"[IS] OR "1588-2519"[IS] OR "1585-1923"[IS] OR "0006-3592"[IS] OR "1097-0290"[IS] OR "1534-3081"[IS] OR "1531-3433"[IS] OR "0163-853X"[IS] OR "1532-6950"[IS] OR "1368-4310"[IS] OR "1368-4310"[IS] OR "2620-1747"[IS] OR "2620-1607"[IS] OR "1229-9367"[IS] OR "1229-9367"[IS] OR "2218-1997"[IS] OR "2218-1997"[IS] OR "2368-7460"[IS] OR "2368-7460"[IS] OR "1471-227X"[IS] OR "1471-227X"[IS] OR "0010-0757"[IS] OR "0010-0757"[IS] OR "1096-987X"[IS] OR "0192-8651"[IS] OR "1932-8664"[IS] OR "1932-8575"[IS] OR "2162-3104"[IS] OR "2166-3750"[IS] OR "1520-5207"[IS] OR "1520-6106"[IS] OR "1999-4923"[IS] OR "1999-4923"[IS] OR "1467-3584"[IS] OR "1742-9692"[IS] OR "0305-7410"[IS] OR "1468-2648"[IS] OR "1845-5719"[IS] OR "1845-5719"[IS] OR "0012-365X"[IS] OR "0012-365X"[IS] OR "1001-6279"[IS] OR "1001-6279"[IS] OR "1468-2435"[IS] OR "0020-7985"[IS] OR "0885-064X"[IS] OR "1090-2708"[IS] OR "2263-8733"[IS] OR "2263-8733"[IS] OR "1936-9743"[IS] OR "1936-9735"[IS] OR "0030-8730"[IS] OR "1945-5844"[IS] OR "0304-3991"[IS] OR "1879-2723"[IS] OR "1568-5527"[IS] OR "0921-8831"[IS] OR "0016-7746"[IS] OR "0016-7746"[IS] OR "1573-6571"[IS] OR "0022-4197"[IS] OR "2049-3738"[IS] OR "2049-372X"[IS] OR "2079-6382"[IS] OR "2079-6382"[IS] OR "1873-4359"[IS] OR "0927-7757"[IS] OR "1095-998X"[IS] OR "0195-6671"[IS] OR "0937-9827"[IS] OR "1437-1596"[IS] OR "2050-3318"[IS] OR "2050-3326"[IS] OR "1541-3780"[IS] OR "0047-2816"[IS] OR "1745-641X"[IS] OR "1745-6428"[IS] OR "0001-1541"[IS] OR "1547-5905"[IS] OR "0006-291X"[IS] OR "1090-2104"[IS] OR "1612-5681"[IS] OR "1612-5681"[IS] OR "1940-1019"[IS] OR "0022-3980"[IS] OR "1545-0066"[IS] OR "1090-3127"[IS] OR "2046-6102"[IS] OR "2046-6099"[IS] OR "2095-3127"[IS] OR "2195-3597"[IS] OR "1126-5434"[IS] OR "0008-0624"[IS] OR "0010-8367"[IS] OR "1460-3691"[IS] OR "1477-9226"[IS] OR "1477-9234"[IS] OR "1465-3435"[IS] OR "0141-8211"[IS] OR "1365-2583"[IS] OR "0962-1075"[IS] OR "1559-9620"[IS] OR "1559-9612"[IS] OR "2223-7747"[IS] OR "2223-7747"[IS] OR "1357-5317"[IS] OR "1468-4519"[IS] OR "2543-2656"[IS] OR "2468-502X"[IS] OR "0003-2654"[IS] OR "1364-5528"[IS] OR "1617-7959"[IS] OR "1617-7940"[IS] OR "0705-3797"[IS] OR "0705-3797"[IS] OR "1551-3114"[IS] OR "1551-3122"[IS] OR "1536-5050"[IS] OR "1558-9439"[IS] OR "1938-971X"[IS] OR "1938-9728"[IS] OR "0888-045X"[IS] OR "0888-045X"[IS] OR "1473-3277"[IS] OR "1473-3285"[IS] OR "0890-4065"[IS] OR "1879-193X"[IS] OR "1469-7580"[IS] OR "0021-8782"[IS] OR "2631-6862"[IS] OR "1938-7806"[IS] OR "1090-2147"[IS] OR "0278-2626"[IS] OR "2772-3976"[IS] OR "2772-3976"[IS] OR "1532-2300"[IS] OR "0737-3937"[IS] OR "1724-6067"[IS] OR "1120-7000"[IS] OR "2075-4450"[IS] OR "2075-4450"[IS] OR "1923-2934"[IS] OR "1923-2926"[IS] OR "1492-3831"[IS] OR "1492-3831"[IS] OR "0033-7587"[IS] OR "1938-5404"[IS] OR "1014-9562"[IS] OR "1014-9562"[IS] OR "2287-237X"[IS] OR "2287-2388"[IS] OR "0736-9387"[IS] OR "1934-7243"[IS] OR "1536-5409"[IS] OR "0749-8047"[IS] OR "0933-7741"[IS] OR "1435-5337"[IS] OR "2095-2228"[IS] OR "2095-2236"[IS] OR "1746-4269"[IS] OR "1746-4269"[IS] OR "0733-9496"[IS] OR "0733-9496"[IS] OR "1467-6435"[IS] OR "0023-5962"[IS] OR "2667-064X"[IS] OR "2667-064X"[IS] OR "0306-3968"[IS] OR "0306-3968"[IS] OR "0036-0112"[IS] OR "1549-0831"[IS] OR "2156-8324"[IS] OR "2156-8316"[IS] OR "0017-811X"[IS] OR "0017-811X"[IS] OR "0961-5539"[IS] OR "0961-5539"[IS] OR "2325-6540"[IS] OR "1096-3758"[IS] OR "2210-9706"[IS] OR "2210-9706"[IS] OR "1095-8673"[IS] OR "0022-4804"[IS] OR "1743-9728"[IS] OR "0957-8811"[IS] OR "2005-7814"[IS] OR "2005-7806"[IS] OR "2560-6778"[IS] OR

	"2560-6921"[IS] OR "1092-7875"[IS] OR "1573-6628"[IS] OR "1076-9242"[IS] OR "1099-0909"[IS] OR "1476-8364"[IS] OR "1043-8599"[IS] OR "0021-9002"[IS] OR "0021-9002"[IS] OR "1740-1453"[IS] OR "1740-1461"[IS] OR "1529-8817"[IS] OR "0022-3646"[IS] OR "2235-3186"[IS] OR "1660-8151"[IS] OR "1070-664X"[IS] OR "1089-7674"[IS] OR "1002-0071"[IS] OR "1002-0071"[IS] OR "2366-0058"[IS] OR "2366-004X"[IS] OR "1749-4478"[IS] OR "1749-4486"[IS] OR "0301-5661"[IS] OR "1600-0528"[IS] OR "1754-7083"[IS] OR "1754-7075"[IS] OR "0179-051X"[IS] OR "1432-0460"[IS] OR "0016-7398"[IS] OR "1475-4959"[IS] OR "1090-7807"[IS] OR "1090-7807"[IS] OR "2541-9404"[IS] OR "2411-3336"[IS] OR "0740-8188"[IS] OR "0740-8188"[IS] OR "1363-254X"[IS] OR "1363-254X"[IS] OR "0954-1314"[IS] OR "1467-646X"[IS] OR "0038-4941"[IS] OR "1540-6237"[IS] OR "2291-9279"[IS] OR "2291-9279"[IS] OR "2079-7737"[IS] OR "2079-7737"[IS] OR "1056-4993"[IS] OR "1558-0490"[IS] OR "2571-6255"[IS] OR "2571-6255"[IS] OR "0966-6362"[IS] OR "0966-6362"[IS] OR "2161-7856"[IS] OR "2161-783X"[IS] OR "1477-0520"[IS] OR "1477-0539"[IS] OR "1572-9737"[IS] OR "1566-0621"[IS] OR "1612-0051"[IS] OR "1125-7865"[IS] OR "1936-6590"[IS] OR "1936-6582"[IS] OR "2228-6497"[IS] OR "2228-6497"[IS] OR "1756-6274"[IS] OR "1756-6266"[IS] OR "0959-289X"[IS] OR "1532-3374"[IS] OR "2561-6722"[IS] OR "2561-6722"[IS] OR "1069-5869"[IS] OR "1531-5851"[IS] OR "0262-1711"[IS] OR "0262-1711"[IS] OR "0143-6597"[IS] OR "1360-2241"[IS] OR "1875-9637"[IS] OR "1875-9637"[IS] OR "0261-0183"[IS] OR "1461-703X"[IS] OR "0883-6353"[IS] OR "1520-6548"[IS] OR "0952-8733"[IS] OR "1740-3863"[IS] OR "2588-8404"[IS] OR "2588-8404"[IS] OR "2194-1459"[IS] OR "2194-1467"[IS] OR "1600-0870"[IS] OR "0280-6495"[IS] OR "2666-7908"[IS] OR "2666-7908"[IS] OR "0929-8673"[IS] OR "1875-533X"[IS] OR "1550-7424"[IS] OR "1550-7424"[IS] OR "0378-5920"[IS] OR "1467-9701"[IS] OR "1006-7191"[IS] OR "1006-7191"[IS] OR "1464-5041"[IS] OR "0268-7038"[IS] OR "1556-7249"[IS] OR "1556-7249"[IS] OR "0924-4247"[IS] OR "0924-4247"[IS] OR "2193-7532"[IS] OR "2193-7532"[IS] OR "1096-0015"[IS] OR "0272-7714"[IS] OR "0268-3768"[IS] OR "1433-3015"[IS] OR "1044-5463"[IS] OR "1557-8992"[IS] OR "1537-1921"[IS] OR "0898-4921"[IS] OR "0895-562X"[IS] OR "1573-0441"[IS] OR "1477-7819"[IS] OR "1477-7819"[IS] OR "0887-4034"[IS] OR "1552-3586"[IS] OR "2666-7592"[IS] OR "2666-7592"[IS] OR "1865-1372"[IS] OR "1865-1380"[IS] OR "1866-9808"[IS] OR "1866-9859"[IS] OR "2196-436X"[IS] OR "2198-3526"[IS] OR "2210-657X"[IS] OR "2210-6561"[IS] OR "0026-4695"[IS] OR "1573-1871"[IS] OR "2352-5541"[IS] OR "2352-5541"[IS] OR "2472-1727"[IS] OR "2472-1727"[IS] OR "2374-8834"[IS] OR "2374-8834"[IS] OR "1539-6746"[IS] OR "1539-6746"[IS] OR "1309-517X"[IS] OR "1309-517X"[IS] OR "1534-6285"[IS] OR "1527-2737"[IS] OR "2666-2817"[IS] OR "2666-2825"[IS] OR "0277-6693"[IS] OR "1099-131X"[IS] OR "0023-8309"[IS] OR "1756-6053"[IS] OR "0300-3930"[IS] OR "1743-9388"[IS] OR "2304-8158"[IS] OR "2304-8158"[IS] OR "1444-1586"[IS] OR "1447-0594"[IS] OR "2164-2850"[IS] OR "2164-2850"[IS] OR "0305-8298"[IS] OR "1477-9021"[IS] OR "1573-0832"[IS] OR "0301-486X"[IS] OR "2326-3660"[IS] OR "0091-3847"[IS] OR "0020-7462"[IS] OR "0020-7462"[IS] OR "1461-5185"[IS] OR "1461-5185"[IS] OR "1073-5623"[IS] OR "1543-1940"[IS] OR "0028-1522"[IS] OR "2161-4296"[IS] OR "1472-6955"[IS] OR "1472-6955"[IS] OR "2516-1032"[IS] OR "2516-1040"[IS] OR "1128-7462"[IS] OR "1591-7398"[IS] OR "0889-3241"[IS] OR "0889-3241"[IS] OR "1644-9665"[IS] OR "1644-9665"[IS] OR "0263-9769"[IS] OR "0951-5224"[IS] OR "0020-5893"[IS] OR "0020-5893"[IS] OR "1537-5315"[IS] OR "1058-5893"[IS] OR "1783-1490"[IS] OR "0019-0829"[IS] OR "0718-1876"[IS] OR "0718-1876"[IS] OR "2452-0748"[IS] OR "2452-0748"[IS] OR "1432-198X"[IS] OR "0931-041X"[IS] OR "0742-4477"[IS] OR "1520-6297"[IS] OR "1546-9530"[IS] OR "1546-9549"[IS] OR "0095-2583"[IS] OR "1465-7295"[IS] OR "1079-0969"[IS] OR "2150-4113"[IS] OR "1944-0405"[IS] OR "1944-0391"[IS] OR "1469-9486"[IS] OR "0309-877X"[IS] OR "2197-4365"[IS] OR "2197-4365"[IS] OR "1090-2155"[IS] OR "0093-934X"[IS] OR "1754-7628"[IS] OR "1467-0100"[IS] OR "0927-0256"[IS] OR "0927-0256"[IS] OR "1749-9755"[IS] OR "1749-9755"[IS] OR "2095-0179"[IS] OR "2095-0187"[IS] OR "1552-695X"[IS] OR "1534-7354"[IS] OR "0021-9673"[IS] OR "1873-3778"[IS] OR "1044-0305"[IS] OR "1879-1123"[IS] OR "0730-7829"[IS] OR "0730-7829"[IS] OR "1936-4776"[IS] OR "1045-6767"[IS] OR "1756-8692"[IS] OR "1756-8692"[IS] OR "0937-3462"[IS] OR "1433-3023"[IS] OR "2090-262X"[IS] OR "0013-2446"[IS] OR "1756-591X"[IS] OR "1756-5901"[IS] OR "1590-1874"[IS] OR "1590-3478"[IS] OR "1873-5223"[IS] OR "1471-5953"[IS] OR "0030-6053"[IS] OR "1365-3008"[IS] OR "1939-0513"[IS] OR "1088-937X"[IS] OR "1099-0720"[IS] OR "0888-4080"[IS] OR "2169-141X"[IS] OR "2169-1401"[IS] OR "1571-0882"[IS] OR "1745-3755"[IS] OR "1432-1114"[IS] OR "0723-4864"[IS] OR "1468-0874"[IS] OR "2041-3149"[IS] OR "2699-0059"[IS] OR "2699-0016"[IS] OR "1424-3210"[IS] OR "1424-8220"[IS] OR "0093-691X"[IS] OR "1879-3231"[IS] OR "0045-2068"[IS] OR "1090-2120"[IS] OR "1744-4136"[IS] OR "0929-7049"[IS] OR "2153-2176"[IS] OR "2153-2168"[IS] OR "0013-1881"[IS] OR "1469-5847"[IS] OR "1018-3639"[IS] OR "1018-3639"[IS] OR "1936-1661"[IS] OR "1936-1653"[IS] OR "1096-6218"[IS] OR "1557-7740"[IS] OR "0142-9418"[IS] OR "0142-9418"[IS] OR "2055-5660"[IS] OR "2055-5660"[IS] OR "1674-7283"[IS] OR "1869-1862"[IS] OR "0963-8288"[IS] OR "1464-5165"[IS] OR "1748-0485"[IS] OR "1748-0493"[IS] OR "2044-3994"[IS] OR "2044-4001"[IS] OR "2192-6611"[IS] OR "2192-662X"[IS] OR "1477-3848"[IS] OR "0743-4618"[IS] OR "1369-4332"[IS] OR "1369-4332"[IS] OR "2096-4250"[IS] OR "2522-8765"[IS] OR "0966-369X"[IS] OR

	"1360-0524"[IS] OR "0964-4008"[IS] OR "0964-4008"[IS] OR "1751-763X"[IS] OR "0024-9831"[IS] OR "1478-5153"[IS] OR "1362-1017"[IS] OR "2305-6304"[IS] OR "2305-6304"[IS] OR "1355-6509"[IS] OR "1355-6509"[IS] OR "0042-207X"[IS] OR "1879-2715"[IS] OR "1687-4285"[IS] OR "2090-3278"[IS] OR "2634-3606"[IS] OR "2634-3606"[IS] OR "1867-2477"[IS] OR "1867-2485"[IS] OR "2469-7249"[IS] OR "2469-7249"[IS] OR "1758-9037"[IS] OR "1834-7649"[IS] OR "1520-6629"[IS] OR "0090-4392"[IS] OR "2376-1032"[IS] OR "2376-0540"[IS] OR "2210-464X"[IS] OR "2210-4690"[IS] OR "0148-6055"[IS] OR "0148-6055"[IS] OR "2415-6310"[IS] OR "2415-6302"[IS] OR "2055-5911"[IS] OR "2055-592X"[IS] OR "2084-879X"[IS] OR "2084-879X"[IS] OR "0953-1513"[IS] OR "1741-4857"[IS] OR "0026-9662"[IS] OR "0026-9662"[IS] OR "0129-2986"[IS] OR "1742-0911"[IS] OR "1868-6354"[IS] OR "1868-6346"[IS] OR "1839-2628"[IS] OR "1832-4274"[IS] OR "0144-6193"[IS] OR "1466-433X"[IS] OR "1064-7406"[IS] OR "1558-1926"[IS] OR "2196-7482"[IS] OR "2196-7482"[IS] OR "1748-7889"[IS] OR "1748-7870"[IS] OR "0309-8265"[IS] OR "1466-1845"[IS] OR "1942-4302"[IS] OR "1942-4310"[IS] OR "1547-6510"[IS] OR "1040-8347"[IS] OR "1552-5007"[IS] OR "1552-5015"[IS] OR "0378-4754"[IS] OR "0378-4754"[IS] OR "2666-660X"[IS] OR "2666-660X"[IS] OR "1420-9055"[IS] OR "1015-1621"[IS] OR "1873-6386"[IS] OR "0160-2527"[IS] OR "0196-206X"[IS] OR "1536-7312"[IS] OR "2666-7215"[IS] OR "2666-7215"[IS] OR "0896-9205"[IS] OR "0896-9205"[IS] OR "1573-1561"[IS] OR "0098-0331"[IS] OR "1064-7554"[IS] OR "1573-7055"[IS] OR "1588-2926"[IS] OR "1388-6150"[IS] OR "1436-2813"[IS] OR "0941-1291"[IS] OR "1380-2038"[IS] OR "1478-2294"[IS] OR "1044-4068"[IS] OR "1044-4068"[IS] OR "1139-9791"[IS] OR "0893-2174"[IS] OR "1467-6370"[IS] OR "1467-6370"[IS] OR "1466-5026"[IS] OR "1466-5034"[IS] OR "1364-5021"[IS] OR "1471-2946"[IS] OR "1873-6254"[IS] OR "0001-706X"[IS] OR "1891-1803"[IS] OR "1891-1803"[IS] OR "1749-8546"[IS] OR "1749-8546"[IS] OR "1547-3384"[IS] OR "1070-289X"[IS] OR "1947-5411"[IS] OR "1947-542X"[IS] OR "1469-9265"[IS] OR "1356-3475"[IS] OR "1038-4871"[IS] OR "1440-1738"[IS] OR "0896-8446"[IS] OR "0896-8446"[IS] OR "0375-7471"[IS] OR "0375-7471"[IS] OR "0039-6109"[IS] OR "1558-3171"[IS] OR "0741-9457"[IS] OR "0741-9457"[IS] OR "1747-3616"[IS] OR "1758-7212"[IS] OR "1467-8268"[IS] OR "1017-6772"[IS] OR "2214-9147"[IS] OR "2214-9147"[IS] OR "1749-7728"[IS] OR "1749-7728"[IS] OR "1557-8062"[IS] OR "1533-1296"[IS] OR "2523-3653"[IS] OR "2523-3661"[IS] OR "1678-7757"[IS] OR "1678-7765"[IS] OR "0898-2104"[IS] OR "1532-2394"[IS] OR "0254-0584"[IS] OR "0254-0584"[IS] OR "1522-2616"[IS] OR "0025-584X"[IS] OR "1466-2523"[IS] OR "1475-2654"[IS] OR "2052-9538"[IS] OR "2052-9538"[IS] OR "1051-0559"[IS] OR "1051-0559"[IS] OR "0147-9563"[IS] OR "1527-3288"[IS] OR "1755-1722"[IS] OR "1755-0882"[IS] OR "1469-6711"[IS] OR "1354-9839"[IS] OR "1664-221X"[IS] OR "1664-2201"[IS] OR "0263-2136"[IS] OR "1460-2229"[IS] OR "2056-5615"[IS] OR "2056-5607"[IS] OR "1097-0010"[IS] OR "0022-5142"[IS] OR "1573-0840"[IS] OR "0921-030X"[IS] OR "1000-1964"[IS] OR "1000-1964"[IS] OR "0882-2786"[IS] OR "0882-2786"[IS] OR "1073-0451"[IS] OR "1073-0451"[IS] OR "1074-9039"[IS] OR "1532-7884"[IS] OR "2645-4904"[IS] OR "2645-4904"[IS] OR "1465-3311"[IS] OR "0004-9182"[IS] OR "2535-0730"[IS] OR "2535-0730"[IS] OR "2766-1512"[IS] OR "2766-1520"[IS] OR "0379-4024"[IS] OR "0379-4024"[IS] OR "2689-6575"[IS] OR "2689-6567"[IS] OR "1536-0067"[IS] OR "1535-2188"[IS] OR "1042-0533"[IS] OR "1520-6300"[IS] OR "1869-215X"[IS] OR "1869-7534"[IS] OR "2666-8939"[IS] OR "2666-8939"[IS] OR "0255-2701"[IS] OR "0255-2701"[IS] OR "2192-8258"[IS] OR "1000-9345"[IS] OR "1478-0917"[IS] OR "1478-0917"[IS] OR "1814-2079"[IS] OR "0022-1686"[IS] OR "1749-799X"[IS] OR "1749-799X"[IS] OR "1437-210X"[IS] OR "0039-7881"[IS] OR "2325-1042"[IS] OR "2325-1042"[IS] OR "1054-6006"[IS] OR "1365-2419"[IS] OR "2509-8829"[IS] OR "2509-8829"[IS] OR "2042-6747"[IS] OR "2042-6755"[IS] OR "0273-4753"[IS] OR "1552-6550"[IS] OR "2095-3836"[IS] OR "2095-3836"[IS] OR "1438-7492"[IS] OR "1439-2054"[IS] OR "2571-550X"[IS] OR "2571-550X"[IS] OR "1664-2376"[IS] OR "1664-2384"[IS] OR "1467-629X"[IS] OR "0810-5391"[IS] OR "1471-2431"[IS] OR "1471-2431"[IS] OR "0011-3921"[IS] OR "1461-7064"[IS] OR "0304-3797"[IS] OR "1469-5898"[IS] OR "0219-4554"[IS] OR "0219-4554"[IS] OR "2169-5032"[IS] OR "1555-3434"[IS] OR "2193-2409"[IS] OR "2193-2409"[IS] OR "1615-6714"[IS] OR "1434-5293"[IS] OR "1319-6103"[IS] OR "1319-6103"[IS] OR "0022-4812"[IS] OR "0022-4812"[IS] OR "1369-3786"[IS] OR "1460-2709"[IS] OR "1467-8039"[IS] OR "1873-5495"[IS] OR "1472-6831"[IS] OR "1472-6831"[IS] OR "0265-0517"[IS] OR "1469-2104"[IS] OR "1420-8954"[IS] OR "1016-3328"[IS] OR "1873-4502"[IS] OR "0886-3350"[IS] OR "0731-9487"[IS] OR "2168-376X"[IS] OR "1349-1008"[IS] OR "1343-943X"[IS] OR "1875-9157"[IS] OR "1875-9181"[IS] OR "1538-9588"[IS] OR "1538-957X"[IS] OR "1965-0175"[IS] OR "1965-0175"[IS] OR "1654-109X"[IS] OR "1402-2001"[IS] OR "0159-6306"[IS] OR "1469-3739"[IS] OR "0379-0738"[IS] OR "1872-6283"[IS] OR "1471-0374"[IS] OR "1470-2266"[IS] OR "1559-2243"[IS] OR "1558-7894"[IS] OR "0025-3162"[IS] OR "1432-1793"[IS] OR "1473-5636"[IS] OR "0960-8931"[IS] OR "1468-4527"[IS] OR "1468-4535"[IS] OR "0301-0074"[IS] OR "0301-0074"[IS] OR "1537-7385"[IS] OR "0894-9115"[IS] OR "1477-2760"[IS] OR "1475-9551"[IS] OR "1524-8399"[IS] OR "1524-8399"[IS] OR "2352-409X"[IS] OR "2352-409X"[IS] OR "1549-3296"[IS] OR "1549-3296"[IS] OR "0894-8453"[IS] OR "1573-3548"[IS] OR "0303-6812"[IS] OR "1432-1416"[IS] OR "1466-4496"[IS] OR "0261-4367"[IS] OR "0277-2833"[IS] OR "0277-2833"[IS] OR "0334-4355"[IS] OR "2040-4786"[IS] OR "1077-8012"[IS] OR

	"1552-8448"[IS] OR "0963-0643"[IS] OR "1473-6586"[IS] OR "2151-8556"[IS] OR "0018-8646"[IS] OR "1463-4201"[IS] OR "1463-4201"[IS] OR "2220-9964"[IS] OR "2220-9964"[IS] OR "1751-3766"[IS] OR "1751-3758"[IS] OR "1547-5441"[IS] OR "1547-3341"[IS] OR "1754-9493"[IS] OR "1754-9493"[IS] OR "1476-7244"[IS] OR "0811-1146"[IS] OR "0001-6810"[IS] OR "0001-6810"[IS] OR "1868-596X"[IS] OR "1868-8551"[IS] OR "0024-4066"[IS] OR "1095-8312"[IS] OR "1465-7287"[IS] OR "1074-3529"[IS] OR "1468-7968"[IS] OR "1741-2706"[IS] OR "2297-1769"[IS] OR "2297-1769"[IS] OR "1539-3100"[IS] OR "1539-3100"[IS] OR "1528-3577"[IS] OR "1528-3585"[IS] OR "0021-8936"[IS] OR "1528-9036"[IS] OR "1572-6657"[IS] OR "1572-6657"[IS] OR "1067-1927"[IS] OR "1524-475X"[IS] OR "0001-6993"[IS] OR "1502-3869"[IS] OR "1753-8157"[IS] OR "1753-8157"[IS] OR "1464-519X"[IS] OR "0260-1370"[IS] OR "2689-8381"[IS] OR "2689-8381"[IS] OR "1095-9963"[IS] OR "0733-5210"[IS] OR "0022-1910"[IS] OR "1879-1611"[IS] OR "2199-8531"[IS] OR "2199-8531"[IS] OR "1573-3394"[IS] OR "1065-3058"[IS] OR "2666-5204"[IS] OR "2666-5204"[IS] OR "1056-7941"[IS] OR "1056-7941"[IS] OR "2590-1567"[IS] OR "2590-1567"[IS] OR "0896-1530"[IS] OR "1528-7068"[IS] OR "2524-4957"[IS] OR "2524-4965"[IS] OR "1360-8746"[IS] OR "1743-9612"[IS] OR "1754-2731"[IS] OR "1754-2731"[IS] OR "2194-7236"[IS] OR "2194-7228"[IS] OR "1444-0938"[IS] OR "0816-4622"[IS] OR "1730-6310"[IS] OR "0012-3862"[IS] OR "0020-6539"[IS] OR "1875-595X"[IS] OR "1640-5544"[IS] OR "1899-7562"[IS] OR "0026-265X"[IS] OR "0026-265X"[IS] OR "1741-0401"[IS] OR "1741-0401"[IS] OR "1366-5901"[IS] OR "0143-1161"[IS] OR "0025-5564"[IS] OR "0025-5564"[IS] OR "1684-3703"[IS] OR "1684-3703"[IS] OR "1478-1700"[IS] OR "1751-2921"[IS] OR "1002-0063"[IS] OR "1993-064X"[IS] OR "2191-5040"[IS] OR "2191-5040"[IS] OR "1572-8900"[IS] OR "1566-2543"[IS] OR "0025-5645"[IS] OR "0025-5645"[IS] OR "2048-7010"[IS] OR "2048-7010"[IS] OR "1467-839X"[IS] OR "1367-2223"[IS] OR "0936-9937"[IS] OR "1432-1386"[IS] OR "2693-9169"[IS] OR "2693-9169"[IS] OR "1940-3372"[IS] OR "1940-3372"[IS] OR "2287-903X"[IS] OR "2287-8882"[IS] OR "2737-0690"[IS] OR "2737-114X"[IS] OR "2215-0382"[IS] OR "2215-0382"[IS] OR "2379-8068"[IS] OR "2379-8068"[IS] OR "0963-2719"[IS] OR "1752-7015"[IS] OR "1434-7636"[IS] OR "1434-5021"[IS] OR "0018-9359"[IS] OR "1557-9638"[IS] OR "0364-216X"[IS] OR "1432-5241"[IS] OR "0001-8244"[IS] OR "1573-3297"[IS] OR "2096-5192"[IS] OR "2096-5192"[IS] OR "1095-922X"[IS] OR "0140-1963"[IS] OR "0891-2416"[IS] OR "1552-5414"[IS] OR "1673-1581"[IS] OR "1673-1581"[IS] OR "2405-8718"[IS] OR "2405-8726"[IS] OR "0969-594X"[IS] OR "1465-329X"[IS] OR "2524-7581"[IS] OR "2197-120X"[IS] OR "2590-2571"[IS] OR "2590-2571"[IS] OR "0013-1245"[IS] OR "1552-3535"[IS] OR "2079-3200"[IS] OR "2079-3200"[IS] OR "1749-7345"[IS] OR "0893-8849"[IS] OR "2363-6203"[IS] OR "2363-6211"[IS] OR "2243-4690"[IS] OR "2243-4690"[IS] OR "1879-0720"[IS] OR "0928-0987"[IS] OR "2589-871X"[IS] OR "2589-871X"[IS] OR "0958-6946"[IS] OR "0958-6946"[IS] OR "0342-7188"[IS] OR "1432-1319"[IS] OR "1573-045X"[IS] OR "0895-5638"[IS] OR "1464-0686"[IS] OR "0965-8211"[IS] OR "1559-0720"[IS] OR "0163-4984"[IS] OR "1556-3324"[IS] OR "1556-3316"[IS] OR "2308-3425"[IS] OR "2308-3425"[IS] OR "2093-5552"[IS] OR "2093-6214"[IS] OR "0040-0262"[IS] OR "1996-8175"[IS] OR "2329-7662"[IS] OR "2329-7670"[IS] OR "1855-6531"[IS] OR "1854-6250"[IS] OR "1096-9934"[IS] OR "0270-7314"[IS] OR "1226-9239"[IS] OR "1226-9239"[IS] OR "0022-474X"[IS] OR "0022-474X"[IS] OR "1478-9523"[IS] OR "1478-9515"[IS] OR "1598-1037"[IS] OR "1598-1037"[IS] OR "1532-6969"[IS] OR "1040-7413"[IS] OR "1350-293X"[IS] OR "1752-1807"[IS] OR "0143-7496"[IS] OR "0143-7496"[IS] OR "2073-4441"[IS] OR "2073-4441"[IS] OR "1432-0940"[IS] OR "0176-4276"[IS] OR "1001-6058"[IS] OR "1001-6058"[IS] OR "1572-9753"[IS] OR "1366-638X"[IS] OR "1478-341X"[IS] OR "0041-0020"[IS] OR "1468-4489"[IS] OR "0963-9284"[IS] OR "0010-4086"[IS] OR "1545-701X"[IS] OR "2666-6472"[IS] OR "2666-6472"[IS] OR "1833-3575"[IS] OR "1833-3583"[IS] OR "1573-5117"[IS] OR "0018-8158"[IS] OR "0305-0629"[IS] OR "1547-7444"[IS] OR "2214-8051"[IS] OR "2214-8043"[IS] OR "1435-2451"[IS] OR "1435-2443"[IS] OR "0733-2467"[IS] OR "1520-6777"[IS] OR "0378-1135"[IS] OR "1873-2542"[IS] OR "1740-8261"[IS] OR "1058-8183"[IS] OR "1362-0436"[IS] OR "1362-0436"[IS] OR "0105-1873"[IS] OR "1600-0536"[IS] OR "1432-1068"[IS] OR "1633-8065"[IS] OR "1611-2156"[IS] OR "1611-2156"[IS] OR "1096-0430"[IS] OR "0021-9045"[IS] OR "1533-2918"[IS] OR "1533-290X"[IS] OR "1380-7501"[IS] OR "1573-7721"[IS] OR "2073-4360"[IS] OR "2073-4360"[IS] OR "1533-8665"[IS] OR "0195-6086"[IS] OR "2213-6258"[IS] OR "2213-624X"[IS] OR "2666-7843"[IS] OR "2666-7843"[IS] OR "1469-3623"[IS] OR "0305-7925"[IS] OR "1477-0857"[IS] OR "1471-4175"[IS] OR "1040-6190"[IS] OR "1040-6190"[IS] OR "1932-8036"[IS] OR "1932-8036"[IS] OR "1753-0369"[IS] OR "1753-0350"[IS] OR "2041-7136"[IS] OR "2041-7136"[IS] OR "0001-6268"[IS] OR "0942-0940"[IS] OR "2562-7783"[IS] OR "2562-7775"[IS] OR "0267-3649"[IS] OR "0267-3649"[IS] OR "2406-825X"[IS] OR "2089-1490"[IS] OR "1566-1199"[IS] OR "1566-1199"[IS] OR "0032-0862"[IS] OR "1365-3059"[IS] OR "2590-1389"[IS] OR "2590-1389"[IS] OR "1591-996X"[IS] OR "2035-648X"[IS] OR "0435-3684"[IS] OR "1468-0467"[IS] OR "1943-9970"[IS] OR "1943-9962"[IS] OR "2040-8269"[IS] OR "2040-8269"[IS] OR "2367-8194"[IS] OR "2367-8194"[IS] OR "1750-8924"[IS] OR "1750-8916"[IS] OR "1936-1521"[IS] OR "1936-153X"[IS] OR "0718-9516"[IS] OR "0718-9516"[IS] OR "0270-1367"[IS] OR "0364-9857"[IS] OR "1476-5624"[IS] OR "1362-4393"[IS] OR "1476-7503"[IS] OR "1476-7503"[IS] OR "2666-0865"[IS] OR "2666-0865"[IS] OR "1600-048X"[IS] OR

	"0908-8857"[IS] OR "1467-9256"[IS] OR "0263-3957"[IS] OR "2573-0142"[IS] OR "2573-0142"[IS] OR "1469-7696"[IS] OR "1469-7688"[IS] OR "1355-2546"[IS] OR "1355-2546"[IS] OR "1088-4165"[IS] OR "1088-4165"[IS] OR "0940-9602"[IS] OR "0940-9602"[IS] OR "0305-4179"[IS] OR "1879-1409"[IS] OR "0018-0831"[IS] OR "0018-0831"[IS] OR "0219-6220"[IS] OR "0219-6220"[IS] OR "0032-8855"[IS] OR "1552-7522"[IS] OR "2079-6374"[IS] OR "2079-6374"[IS] OR "0261-2194"[IS] OR "0261-2194"[IS] OR "2053-4701"[IS] OR "2053-4701"[IS] OR "0363-2415"[IS] OR "1548-8446"[IS] OR "1527-0025"[IS] OR "1552-7794"[IS] OR "1052-3057"[IS] OR "1532-8511"[IS] OR "2504-3889"[IS] OR "2504-3889"[IS] OR "1558-1365"[IS] OR "1042-3699"[IS] OR "1878-5166"[IS] OR "1878-5158"[IS] OR "1502-3923"[IS] OR "0020-174X"[IS] OR "1520-8524"[IS] OR "0001-4966"[IS] OR "1523-0406"[IS] OR "1523-0406"[IS] OR "1109-4028"[IS] OR "1109-4028"[IS] OR "1860-7187"[IS] OR "1860-7179"[IS] OR "8755-4615"[IS] OR "8755-4615"[IS] OR "1521-0480"[IS] OR "0891-6152"[IS] OR "1558-9447"[IS] OR "1558-9455"[IS] OR "0020-8728"[IS] OR "1461-7234"[IS] OR "1085-7117"[IS] OR "1537-2693"[IS] OR "0022-1147"[IS] OR "1750-3841"[IS] OR "0951-5089"[IS] OR "1465-394X"[IS] OR "0920-1742"[IS] OR "1573-5168"[IS] OR "1728-869X"[IS] OR "1728-869X"[IS] OR "2504-4494"[IS] OR "2504-4494"[IS] OR "0898-5898"[IS] OR "0898-5898"[IS] OR "1522-8878"[IS] OR "1522-8878"[IS] OR "0304-4017"[IS] OR "1873-2550"[IS] OR "2201-1919"[IS] OR "2201-1919"[IS] OR "2164-6589"[IS] OR "2164-6570"[IS] OR "0022-2372"[IS] OR "1545-1542"[IS] OR "2158-3803"[IS] OR "2158-379X"[IS] OR "2041-7942"[IS] OR "0025-5793"[IS] OR "1751-9918"[IS] OR "1751-9918"[IS] OR "1551-3688"[IS] OR "1551-3688"[IS] OR "1740-388X"[IS] OR "1472-4790"[IS] OR "0278-4343"[IS] OR "0278-4343"[IS] OR "1642-3593"[IS] OR "1642-3593"[IS] OR "1574-0196"[IS] OR "1574-0196"[IS] OR "1470-3297"[IS] OR "1470-3300"[IS] OR "2577-4441"[IS] OR "2577-445X"[IS] OR "1757-2223"[IS] OR "1757-2231"[IS] OR "0931-2668"[IS] OR "1439-0388"[IS] OR "0167-5877"[IS] OR "1873-1716"[IS] OR "1988-2807"[IS] OR "1139-1138"[IS] OR "2093-0860"[IS] OR "1226-2617"[IS] OR "1532-818X"[IS] OR "0196-0709"[IS] OR "2059-5816"[IS] OR "2059-5816"[IS] OR "2237-9622"[IS] OR "1679-4974"[IS] OR "1445-4408"[IS] OR "1445-4416"[IS] OR "1465-3869"[IS] OR "0958-9236"[IS] OR "1532-8481"[IS] OR "8755-7223"[IS] OR "2476-9304"[IS] OR "2355-1895"[IS] OR "1095-0761"[IS] OR "1095-0761"[IS] OR "1612-4677"[IS] OR "1612-4669"[IS] OR "2662-9992"[IS] OR "2662-9992"[IS] OR "0957-4484"[IS] OR "1361-6528"[IS] OR "1573-5087"[IS] OR "0167-6903"[IS] OR "1945-5119"[IS] OR "1074-9357"[IS] OR "1476-5829"[IS] OR "1476-5810"[IS] OR "1942-7611"[IS] OR "1942-7603"[IS] OR "1389-5753"[IS] OR "1389-5753"[IS] OR "0014-1844"[IS] OR "1469-588X"[IS] OR "1064-119X"[IS] OR "1521-0618"[IS] OR "1420-3049"[IS] OR "1420-3049"[IS] OR "0263-5747"[IS] OR "1469-8668"[IS] OR "1477-2833"[IS] OR "1028-6632"[IS] OR "1674-7321"[IS] OR "1869-1900"[IS] OR "1832-8105"[IS] OR "1748-3131"[IS] OR "0017-8039"[IS] OR "0017-8039"[IS] OR "0918-9440"[IS] OR "1618-0860"[IS] OR "2474-736X"[IS] OR "2474-736X"[IS] OR "1753-5069"[IS] OR "1753-5077"[IS] OR "0161-7761"[IS] OR "1548-1492"[IS] OR "0165-7836"[IS] OR "0165-7836"[IS] OR "1742-9153"[IS] OR "1742-9145"[IS] OR "2378-8038"[IS] OR "2378-8038"[IS] OR "1095-158X"[IS] OR "1559-3126"[IS] OR "0033-5177"[IS] OR "1573-7845"[IS] OR "1948-8335"[IS] OR "1948-8327"[IS] OR "0015-752X"[IS] OR "1464-3626"[IS] OR "1528-3488"[IS] OR "1532-706X"[IS] OR "1029-8649"[IS] OR "2045-4147"[IS] OR "2524-4442"[IS] OR "2096-496X"[IS] OR "2666-6820"[IS] OR "2666-6820"[IS] OR "1873-2380"[IS] OR "0021-9290"[IS] OR "1296-2074"[IS] OR "1296-2074"[IS] OR "2045-0893"[IS] OR "2045-0885"[IS] OR "0033-3085"[IS] OR "1520-6807"[IS] OR "0020-1383"[IS] OR "1879-0267"[IS] OR "1568-4156"[IS] OR "1568-4156"[IS] OR "1052-9284"[IS] OR "1099-1298"[IS] OR "0022-0493"[IS] OR "0022-0493"[IS] OR "0360-5310"[IS] OR "1744-5019"[IS] OR "2667-1433"[IS] OR "2667-1433"[IS] OR "1467-2715"[IS] OR "1472-6033"[IS] OR "2661-8974"[IS] OR "2661-8974"[IS] OR "2225-4110"[IS] OR "2225-4110"[IS] OR "1464-3839"[IS] OR "0013-0915"[IS] OR "0737-1209"[IS] OR "1525-1446"[IS] OR "1613-7159"[IS] OR "1866-749X"[IS] OR "0035-6883"[IS] OR "0035-6883"[IS] OR "0358-5522"[IS] OR "1750-2837"[IS] OR "0038-2876"[IS] OR "0038-2876"[IS] OR "0045-5067"[IS] OR "1208-6037"[IS] OR "0963-8008"[IS] OR "1468-0416"[IS] OR "1462-6004"[IS] OR "1462-6004"[IS] OR "1748-6963"[IS] OR "1743-5889"[IS] OR "2167-8359"[IS] OR "2167-8359"[IS] OR "1381-5148"[IS] OR "1381-5148"[IS] OR "1319-562X"[IS] OR "1319-562X"[IS] OR "2470-1343"[IS] OR "2470-1343"[IS] OR "1732-2421"[IS] OR "0567-7920"[IS] OR "0955-7997"[IS] OR "0955-7997"[IS] OR "0022-2585"[IS] OR "0022-2585"[IS] OR "2520-8128"[IS] OR "2520-811X"[IS] OR "1873-2704"[IS] OR "1540-7489"[IS] OR "1742-481X"[IS] OR "1742-4801"[IS] OR "1479-5752"[IS] OR "0090-9882"[IS] OR "2052-1707"[IS] OR "2052-1707"[IS] OR "1060-586X"[IS] OR "1060-586X"[IS] OR "1841-5261"[IS] OR "1841-5261"[IS] OR "2205-0795"[IS] OR "2205-0795"[IS] OR "1931-3918"[IS] OR "1931-3918"[IS] OR "1614-3116"[IS] OR "0567-7718"[IS] OR "1467-8284"[IS] OR "0003-2638"[IS] OR "1432-0665"[IS] OR "0933-5846"[IS] OR "0005-0067"[IS] OR "1742-9544"[IS] OR "1755-6171"[IS] OR "1755-618X"[IS] OR "2212-0548"[IS] OR "2212-0548"[IS] OR "0380-1330"[IS] OR "0380-1330"[IS] OR "1471-8731"[IS] OR "1471-8847"[IS] OR "2048-7177"[IS] OR "2048-7177"[IS] OR "2044-2041"[IS] OR "2044-205X"[IS] OR "1557-9336"[IS] OR "1557-9336"[IS] OR "0887-4417"[IS] OR "0887-4417"[IS] OR "0885-7482"[IS] OR "1573-2851"[IS] OR "0952-8369"[IS] OR "1469-7998"[IS] OR "1349-8029"[IS] OR "0387-8105"[IS] OR "1138-5820"[IS] OR "1138-5820"[IS] OR "1744-5302"[IS] OR

	"1754-212X"[IS] OR "0003-4894"[IS] OR "1943-572X"[IS] OR "1177-3928"[IS] OR "1177-3928"[IS] OR "1470-3610"[IS] OR "1352-7258"[IS] OR "2471-5646"[IS] OR "2471-5638"[IS] OR "1879-291X"[IS] OR "0301-5629"[IS] OR "0963-7486"[IS] OR "1465-3478"[IS] OR "1435-5949"[IS] OR "1435-5930"[IS] OR "0022-3727"[IS] OR "1361-6463"[IS] OR "1752-6116"[IS] OR "1476-3141"[IS] OR "1313-2970"[IS] OR "1313-2989"[IS] OR "0951-5666"[IS] OR "1435-5655"[IS] OR "0253-4827"[IS] OR "1573-2754"[IS] OR "2662-7671"[IS] OR "2662-7671"[IS] OR "1740-2344"[IS] OR "0142-7237"[IS] OR "1773-2247"[IS] OR "2588-8943"[IS] OR "1747-5090"[IS] OR "1363-6820"[IS] OR "1369-8001"[IS] OR "1369-8001"[IS] OR "1453-8245"[IS] OR "1453-8245"[IS] OR "1877-5845"[IS] OR "1877-5853"[IS] OR "1947-6035"[IS] OR "1947-6043"[IS] OR "0893-4215"[IS] OR "0893-4215"[IS] OR "1758-7093"[IS] OR "2040-7149"[IS] OR "1095-6840"[IS] OR "0016-6480"[IS] OR "1758-8251"[IS] OR "1758-826X"[IS] OR "1179-1411"[IS] OR "1179-1411"[IS] OR "2369-2529"[IS] OR "2369-2529"[IS] OR "2192-2209"[IS] OR "2192-2195"[IS] OR "0031-9422"[IS] OR "1873-3700"[IS] OR "2168-0450"[IS] OR "2168-0450"[IS] OR "1098-2353"[IS] OR "0897-3806"[IS] OR "2150-6779"[IS] OR "2150-6787"[IS] OR "1758-5899"[IS] OR "1758-5880"[IS] OR "1877-7503"[IS] OR "1877-7503"[IS] OR "2042-7158"[IS] OR "0022-3573"[IS] OR "1744-6198"[IS] OR "0029-6473"[IS] OR "1461-4103"[IS] OR "1461-4103"[IS] OR "2246-2929"[IS] OR "2246-2929"[IS] OR "1465-3877"[IS] OR "0305-7240"[IS] OR "0168-9002"[IS] OR "0168-9002"[IS] OR "1467-9450"[IS] OR "0036-5564"[IS] OR "1522-1709"[IS] OR "1520-9512"[IS] OR "2076-2615"[IS] OR "2076-2615"[IS] OR "1030-1887"[IS] OR "1030-1887"[IS] OR "1572-8404"[IS] OR "0169-3867"[IS] OR "1558-4046"[IS] OR "1540-7993"[IS] OR "0802-6106"[IS] OR "1473-4192"[IS] OR "1460-3667"[IS] OR "0951-6298"[IS] OR "1834-7819"[IS] OR "0045-0421"[IS] OR "0143-7208"[IS] OR "1873-3743"[IS] OR "1568-4474"[IS] OR "1568-4474"[IS] OR "0197-7261"[IS] OR "2051-6185"[IS] OR "0279-0750"[IS] OR "1468-0114"[IS] OR "1469-8161"[IS] OR "0031-1820"[IS] OR "1747-5341"[IS] OR "1747-5341"[IS] OR "2411-1236"[IS] OR "2313-2299"[IS] OR "2666-8335"[IS] OR "2666-8335"[IS] OR "1097-0363"[IS] OR "0271-2091"[IS] OR "1750-6182"[IS] OR "1750-6182"[IS] OR "1178-2390"[IS] OR "1178-2390"[IS] OR "0377-0257"[IS] OR "0377-0257"[IS] OR "1479-3261"[IS] OR "1476-4431"[IS] OR "2452-2198"[IS] OR "2452-2198"[IS] OR "1477-8211"[IS] OR "1477-822X"[IS] OR "1103-3088"[IS] OR "1103-3088"[IS] OR "1751-9578"[IS] OR "1751-956X"[IS] OR "0167-6318"[IS] OR "1613-3676"[IS] OR "1541-132X"[IS] OR "1541-1338"[IS] OR "1532-2122"[IS] OR "1462-3889"[IS] OR "1745-4832"[IS] OR "1745-4840"[IS] OR "2251-7715"[IS] OR "2195-3228"[IS] OR "1864-1105"[IS] OR "2151-2388"[IS] OR "0377-8398"[IS] OR "0377-8398"[IS] OR "2332-8894"[IS] OR "2332-8908"[IS] OR "1421-9964"[IS] OR "1015-3837"[IS] OR "1741-3087"[IS] OR "0305-7356"[IS] OR "1435-6066"[IS] OR "0934-9839"[IS] OR "1877-9174"[IS] OR "1877-9166"[IS] OR "1750-4813"[IS] OR "1750-4821"[IS] OR "0172-9179"[IS] OR "1612-4820"[IS] OR "1536-1241"[IS] OR "1558-2639"[IS] OR "1092-6771"[IS] OR "1545-083X"[IS] OR "1748-7692"[IS] OR "0824-0469"[IS] OR "1573-6717"[IS] OR "0146-1044"[IS] OR "0039-3746"[IS] OR "0039-3746"[IS] OR "1741-2889"[IS] OR "1367-4935"[IS] OR "0022-1309"[IS] OR "1940-0888"[IS] OR "0191-2917"[IS] OR "0191-2917"[IS] OR "0486-6134"[IS] OR "0486-6134"[IS] OR "1475-682X"[IS] OR "0038-0245"[IS] OR "1424-7755"[IS] OR "1662-6370"[IS] OR "2186-6953"[IS] OR "2186-3342"[IS] OR "1460-3624"[IS] OR "0957-9265"[IS] OR "0014-0139"[IS] OR "1366-5847"[IS] OR "0921-2728"[IS] OR "1573-0417"[IS] OR "1615-6102"[IS] OR "0033-183X"[IS] OR "1863-1959"[IS] OR "1863-2378"[IS] OR "1572-9877"[IS] OR "1205-8629"[IS] OR "1478-7431"[IS] OR "0022-0620"[IS] OR "1552-5481"[IS] OR "0192-513X"[IS] OR "0963-8199"[IS] OR "1469-9559"[IS] OR "2572-3170"[IS] OR "2572-3170"[IS] OR "1573-7624"[IS] OR "1384-6175"[IS] OR "1875-5364"[IS] OR "1672-3651"[IS] OR "1462-7264"[IS] OR "1365-8050"[IS] OR "0950-5423"[IS] OR "1365-2621"[IS] OR "1615-7109"[IS] OR "1203-4754"[IS] OR "2469-0228"[IS] OR "2469-0228"[IS] OR "1551-3793"[IS] OR "1551-3777"[IS] OR "0272-8397"[IS] OR "1548-0569"[IS] OR "2444-8656"[IS] OR "2444-8656"[IS] OR "1674-5507"[IS] OR "1674-5507"[IS] OR "1875-0281"[IS] OR "1875-0281"[IS] OR "1682-3141"[IS] OR "1682-3141"[IS] OR "2578-2703"[IS] OR "2578-2703"[IS] OR "2212-4284"[IS] OR "2212-4284"[IS] OR "1573-3319"[IS] OR "1053-1890"[IS] OR "0950-4230"[IS] OR "0950-4230"[IS] OR "0731-6844"[IS] OR "1530-7964"[IS] OR "1442-2018"[IS] OR "1441-0745"[IS] OR "1470-8914"[IS] OR "1476-9336"[IS] OR "0011-2240"[IS] OR "1090-2392"[IS] OR "1861-2784"[IS] OR "1861-2776"[IS] OR "2666-142X"[IS] OR "2666-142X"[IS] OR "0021-9924"[IS] OR "1873-7994"[IS] OR "0974-0430"[IS] OR "0971-5894"[IS] OR "1477-2000"[IS] OR "1478-0933"[IS] OR "1746-6148"[IS] OR "1746-6148"[IS] OR "1674-0750"[IS] OR "2198-2600"[IS] OR "1942-7786"[IS] OR "2633-674X"[IS] OR "1573-7772"[IS] OR "1566-4910"[IS] OR "2058-458X"[IS] OR "2058-458X"[IS] OR "2050-0386"[IS] OR "2050-0394"[IS] OR "0379-6779"[IS] OR "0379-6779"[IS] OR "1396-0296"[IS] OR "1529-8019"[IS] OR "2213-8862"[IS] OR "1991-7902"[IS] OR "1467-9302"[IS] OR "0954-0962"[IS] OR "2329-4523"[IS] OR "2329-4515"[IS] OR "0002-5232"[IS] OR "0002-5232"[IS] OR "0007-0904"[IS] OR "1468-2842"[IS] OR "2374-5126"[IS] OR "2374-5118"[IS] OR "1753-1055"[IS] OR "1753-1063"[IS] OR "2077-1886"[IS] OR "2218-0648"[IS] OR "0022-1155"[IS] OR "0975-8402"[IS] OR "1751-1437"[IS] OR "1751-1437"[IS] OR "1696-4713"[IS] OR "1696-4713"[IS] OR "1467-2995"[IS] OR "1467-2987"[IS] OR "0959-2709"[IS] OR "1474-0001"[IS] OR "1472-4049"[IS] OR "1747-7638"[IS] OR "1752-9921"[IS] OR "1752-993X"[IS] OR "2666-4496"[IS] OR

	"2096-7527"[IS] OR "0196-8092"[IS] OR "1096-9101"[IS] OR "2544-7459"[IS] OR "2544-7459"[IS] OR "1469-3593"[IS] OR "1362-1025"[IS] OR "1744-3881"[IS] OR "1744-3881"[IS] OR "1435-8921"[IS] OR "0377-7332"[IS] OR "1369-1457"[IS] OR "1369-1457"[IS] OR "0961-0006"[IS] OR "1741-6477"[IS] OR "2071-1050"[IS] OR "2071-1050"[IS] OR "2073-4395"[IS] OR "2073-4395"[IS] OR "1572-8420"[IS] OR "0165-0106"[IS] OR "0022-1201"[IS] OR "1938-3746"[IS] OR "2093-7482"[IS] OR "1976-1317"[IS] OR "2297-7775"[IS] OR "2297-7775"[IS] OR "0161-9268"[IS] OR "1550-5014"[IS] OR "0714-9808"[IS] OR "1710-1107"[IS] OR "1469-4433"[IS] OR "0967-0262"[IS] OR "1754-9507"[IS] OR "1754-9515"[IS] OR "2334-5985"[IS] OR "0738-1360"[IS] OR "1744-9006"[IS] OR "1573-062X"[IS] OR "1572-9680"[IS] OR "0167-4366"[IS] OR "2151-2485"[IS] OR "1976-8354"[IS] OR "2045-435X"[IS] OR "2045-4368"[IS] OR "1598-818X"[IS] OR "1598-8198"[IS] OR "2095-2430"[IS] OR "2095-2449"[IS] OR "1043-6596"[IS] OR "1552-7832"[IS] OR "1086-671X"[IS] OR "1086-671X"[IS] OR "1573-9104"[IS] OR "0921-9668"[IS] OR "1747-0161"[IS] OR "2047-6094"[IS] OR "1469-9680"[IS] OR "1367-6261"[IS] OR "1946-2166"[IS] OR "1946-2174"[IS] OR "1613-365X"[IS] OR "1612-295X"[IS] OR "1461-7382"[IS] OR "1363-4607"[IS] OR "1552-3020"[IS] OR "0886-1099"[IS] OR "0268-9146"[IS] OR "1365-2052"[IS] OR "0959-7743"[IS] OR "1474-0540"[IS] OR "0268-5809"[IS] OR "1461-7242"[IS] OR "2168-1376"[IS] OR "2168-1376"[IS] OR "0195-5616"[IS] OR "1878-1306"[IS] OR "2213-7467"[IS] OR "2213-7467"[IS] OR "2000-4508"[IS] OR "2000-4508"[IS] OR "1570-7458"[IS] OR "0013-8703"[IS] OR "1878-8181"[IS] OR "1878-8181"[IS] OR "1752-0762"[IS] OR "1010-6049"[IS] OR "2054-0892"[IS] OR "2054-0906"[IS] OR "2330-0574"[IS] OR "2330-0574"[IS] OR "1470-8477"[IS] OR "1470-8477"[IS] OR "0042-6601"[IS] OR "0042-6601"[IS] OR "1545-5815"[IS] OR "0898-9621"[IS] OR "1305-7464"[IS] OR "1305-7456"[IS] OR "1557-1858"[IS] OR "1557-1866"[IS] OR "1613-964X"[IS] OR "0020-5346"[IS] OR "1094-8074"[IS] OR "1935-3952"[IS] OR "1561-4263"[IS] OR "1561-4263"[IS] OR "1793-7078"[IS] OR "1793-7078"[IS] OR "2329-4973"[IS] OR "2329-4965"[IS] OR "1476-6825"[IS] OR "1476-6825"[IS] OR "1873-6963"[IS] OR "0965-2299"[IS] OR "1881-1361"[IS] OR "0287-4547"[IS] OR "2633-8076"[IS] OR "2633-8084"[IS] OR "0947-5745"[IS] OR "1439-0469"[IS] OR "1176-6336"[IS] OR "1176-6336"[IS] OR "1096-8644"[IS] OR "0002-9483"[IS] OR "1469-3615"[IS] OR "1366-8803"[IS] OR "1099-0542"[IS] OR "1061-3773"[IS] OR "1740-5912"[IS] OR "1740-5904"[IS] OR "1529-7470"[IS] OR "1533-6239"[IS] OR "1280-9659"[IS] OR "1638-9395"[IS] OR "1096-0481"[IS] OR "0889-1575"[IS] OR "0885-9701"[IS] OR "1550-509X"[IS] OR "0193-0826"[IS] OR "0193-0826"[IS] OR "2014-2862"[IS] OR "2014-2862"[IS] OR "0003-813X"[IS] OR "1475-4754"[IS] OR "2470-4024"[IS] OR "2095-4816"[IS] OR "1999-4907"[IS] OR "1999-4907"[IS] OR "1471-0307"[IS] OR "1364-727X"[IS] OR "1468-0173"[IS] OR "1468-0173"[IS] OR "0094-582X"[IS] OR "1552-678X"[IS] OR "1467-923X"[IS] OR "0032-3179"[IS] OR "1297-9678"[IS] OR "0044-8435"[IS] OR "2399-1402"[IS] OR "2399-1399"[IS] OR "2631-7680"[IS] OR "2631-7680"[IS] OR "1648-5831"[IS] OR "1648-5831"[IS] OR "1945-0079"[IS] OR "1057-0837"[IS] OR "2055-298X"[IS] OR "2055-298X"[IS] OR "1435-0653"[IS] OR "0011-183X"[IS] OR "2282-4324"[IS] OR "2282-4324"[IS] OR "0304-2294"[IS] OR "2295-5739"[IS] OR "2371-1671"[IS] OR "2371-1671"[IS] OR "0144-8188"[IS] OR "0144-8188"[IS] OR "0022-0078"[IS] OR "1745-6606"[IS] OR "1752-4512"[IS] OR "1752-4520"[IS] OR "0065-2156"[IS] OR "0065-2156"[IS] OR "2199-9260"[IS] OR "2199-9279"[IS] OR "0951-3558"[IS] OR "0951-3558"[IS] OR "0162-0134"[IS] OR "1873-3344"[IS] OR "2048-6790"[IS] OR "2048-6790"[IS] OR "0022-4901"[IS] OR "1745-4603"[IS] OR "1388-0209"[IS] OR "1744-5116"[IS] OR "0007-070X"[IS] OR "0007-070X"[IS] OR "1873-8052"[IS] OR "0911-6044"[IS] OR "1836-2206"[IS] OR "0817-8542"[IS] OR "2251-7308"[IS] OR "2228-5881"[IS] OR "2043-6238"[IS] OR "2043-6246"[IS] OR "0269-283X"[IS] OR "1365-2915"[IS] OR "1743-4777"[IS] OR "0043-9339"[IS] OR "0740-2007"[IS] OR "2154-4689"[IS] OR "1099-8004"[IS] OR "1099-8004"[IS] OR "2054-8923"[IS] OR "2054-8923"[IS] OR "1470-1375"[IS] OR "0043-8243"[IS] OR "2666-9129"[IS] OR "2666-9129"[IS] OR "1365-893X"[IS] OR "1365-893X"[IS] OR "1174-1740"[IS] OR "1177-1801"[IS] OR "1348-8678"[IS] OR "2331-2548"[IS] OR "2053-7166"[IS] OR "2053-7166"[IS] OR "1344-7610"[IS] OR "1347-3735"[IS] OR "1424-2818"[IS] OR "1424-2818"[IS] OR "1435-0211"[IS] OR "1611-4663"[IS] OR "0950-5431"[IS] OR "0950-5431"[IS] OR "1873-6297"[IS] OR "0001-6918"[IS] OR "1552-3578"[IS] OR "1069-3971"[IS] OR "0148-558X"[IS] OR "0148-558X"[IS] OR "1864-063X"[IS] OR "1864-063X"[IS] OR "1432-136X"[IS] OR "0174-1578"[IS] OR "1477-8238"[IS] OR "1477-8246"[IS] OR "1941-9902"[IS] OR "1941-9899"[IS] OR "0167-6393"[IS] OR "0167-6393"[IS] OR "1573-143X"[IS] OR "0967-6120"[IS] OR "1744-117X"[IS] OR "1744-117X"[IS] OR "1470-1227"[IS] OR "0261-5479"[IS] OR "1369-1481"[IS] OR "1369-1481"[IS] OR "0920-203X"[IS] OR "1741-590X"[IS] OR "0020-7144"[IS] OR "1744-5183"[IS] OR "0020-7152"[IS] OR "0020-7152"[IS] OR "1608-1021"[IS] OR "2617-0795"[IS] OR "0043-1745"[IS] OR "1550-2759"[IS] OR "2071-2936"[IS] OR "2071-2928"[IS] OR "2374-3689"[IS] OR "2374-3670"[IS] OR "1439-0396"[IS] OR "0931-2439"[IS] OR "1471-2962"[IS] OR "1364-503X"[IS] OR "1543-2785"[IS] OR "0741-1235"[IS] OR "1532-4400"[IS] OR "1532-4400"[IS] OR "0172-2190"[IS] OR "0172-2190"[IS] OR "1941-3300"[IS] OR "0022-4561"[IS] OR "2054-1058"[IS] OR "2054-1058"[IS] OR "1467-8500"[IS] OR "0313-6647"[IS] OR "1601-5029"[IS] OR "1601-5037"[IS] OR "2227-5223"[IS] OR "2095-2899"[IS] OR "1745-4514"[IS] OR "0145-8884"[IS] OR "1528-3976"[IS] OR "1071-5754"[IS] OR "0716-1417"[IS] OR

	"0716-1417"[IS] OR "0098-1389"[IS] OR "1541-034X"[IS] OR "1556-7125"[IS] OR "1535-3141"[IS] OR "1464-0678"[IS] OR "1357-650X"[IS] OR "0047-5394"[IS] OR "0047-5394"[IS] OR "2168-3565"[IS] OR "2168-3573"[IS] OR "0971-8524"[IS] OR "0973-0656"[IS] OR "2352-4588"[IS] OR "2352-4588"[IS] OR "2095-4964"[IS] OR "2095-4964"[IS] OR "2300-7648"[IS] OR "2353-5636"[IS] OR "0013-8746"[IS] OR "0013-8746"[IS] OR "2666-061X"[IS] OR "2666-061X"[IS] OR "1871-014X"[IS] OR "1871-0131"[IS] OR "1996-1073"[IS] OR "1996-1073"[IS] OR "1461-9571"[IS] OR "1741-2722"[IS] OR "1552-146X"[IS] OR "0093-0334"[IS] OR "0951-2748"[IS] OR "1470-1332"[IS] OR "2586-6060"[IS] OR "2586-6052"[IS] OR "1744-7348"[IS] OR "0003-4746"[IS] OR "0926-2644"[IS] OR "1572-9907"[IS] OR "1098-240X"[IS] OR "0160-6891"[IS] OR "0168-1591"[IS] OR "0168-1591"[IS] OR "1357-034X"[IS] OR "1460-3632"[IS] OR "1042-9247"[IS] OR "1042-9247"[IS] OR "1572-9915"[IS] OR "0300-7839"[IS] OR "1931-762X"[IS] OR "1931-7611"[IS] OR "1752-7724"[IS] OR "1752-7716"[IS] OR "1363-951X"[IS] OR "1363-951X"[IS] OR "1530-7131"[IS] OR "1531-2542"[IS] OR "2057-0198"[IS] OR "2057-0201"[IS] OR "2196-3010"[IS] OR "2196-3010"[IS] OR "1526-9914"[IS] OR "1526-9914"[IS] OR "0951-6328"[IS] OR "1471-6925"[IS] OR "2686-8024"[IS] OR "2687-0088"[IS] OR "0931-1890"[IS] OR "1432-2285"[IS] OR "2666-5158"[IS] OR "2666-5158"[IS] OR "1468-0408"[IS] OR "0267-4424"[IS] OR "1527-3342"[IS] OR "1527-3342"[IS] OR "1747-9541"[IS] OR "1747-9541"[IS] OR "2314-4599"[IS] OR "2314-4580"[IS] OR "0148-4834"[IS] OR "0148-4834"[IS] OR "1099-1476"[IS] OR "0170-4214"[IS] OR "0168-647X"[IS] OR "0168-647X"[IS] OR "2213-2244"[IS] OR "2213-2244"[IS] OR "1752-0606"[IS] OR "0194-472X"[IS] OR "1047-3203"[IS] OR "1047-3203"[IS] OR "1872-9185"[IS] OR "0964-7775"[IS] OR "2304-6775"[IS] OR "2304-6775"[IS] OR "1742-1780"[IS] OR "1742-1772"[IS] OR "2072-0815"[IS] OR "2072-0815"[IS] OR "2337-0173"[IS] OR "2336-9744"[IS] OR "1938-7636"[IS] OR "1938-6400"[IS] OR "0946-672X"[IS] OR "0946-672X"[IS] OR "0004-8038"[IS] OR "2732-4613"[IS] OR "2624-9367"[IS] OR "2624-9367"[IS] OR "1876-4053"[IS] OR "1876-4045"[IS] OR "1468-3857"[IS] OR "1468-3857"[IS] OR "1613-4079"[IS] OR "0921-4771"[IS] OR "0272-9490"[IS] OR "0272-9490"[IS] OR "1226-5934"[IS] OR "2161-6779"[IS] OR "1365-2761"[IS] OR "0140-7775"[IS] OR "1466-7681"[IS] OR "1466-769X"[IS] OR "1617-4909"[IS] OR "1617-4917"[IS] OR "2254-3139"[IS] OR "2254-3139"[IS] OR "2541-9595"[IS] OR "2221-2701"[IS] OR "1752-7589"[IS] OR "1745-2007"[IS] OR "0045-3102"[IS] OR "1468-263X"[IS] OR "1465-3982"[IS] OR "0953-8259"[IS] OR "1558-0954"[IS] OR "1097-1475"[IS] OR "1745-6673"[IS] OR "1745-6673"[IS] OR "1988-2904"[IS] OR "1138-7416"[IS] OR "0092-055X"[IS] OR "1939-862X"[IS] OR "1351-847X"[IS] OR "1466-4364"[IS] OR "1529-3785"[IS] OR "1557-945X"[IS] OR "1753-8963"[IS] OR "1753-8971"[IS] OR "2164-5701"[IS] OR "2164-5698"[IS] OR "1871-4528"[IS] OR "0014-3065"[IS] OR "1572-9842"[IS] OR "0263-0338"[IS] OR "2192-9521"[IS] OR "2192-953X"[IS] OR "1878-4518"[IS] OR "1878-450X"[IS] OR "0029-4527"[IS] OR "1939-0726"[IS] OR "2328-9252"[IS] OR "2328-9260"[IS] OR "2326-7852"[IS] OR "2326-7836"[IS] OR "1680-2012"[IS] OR "1680-2012"[IS] OR "1043-9862"[IS] OR "1552-5406"[IS] OR "1742-1713"[IS] OR "1742-1705"[IS] OR "1750-8533"[IS] OR "1750-8614"[IS] OR "0167-7411"[IS] OR "1572-8749"[IS] OR "1474-4740"[IS] OR "1477-0881"[IS] OR "0176-4268"[IS] OR "1432-1343"[IS] OR "0022-4510"[IS] OR "1748-5827"[IS] OR "1072-4745"[IS] OR "1468-2893"[IS] OR "1322-7114"[IS] OR "1440-172X"[IS] OR "1556-181X"[IS] OR "1556-1801"[IS] OR "1556-4886"[IS] OR "1556-4991"[IS] OR "2765-0235"[IS] OR "2765-0189"[IS] OR "1573-8604"[IS] OR "0164-0291"[IS] OR "2772-5669"[IS] OR "2772-5669"[IS] OR "0922-1565"[IS] OR "1478-9698"[IS] OR "2041-3335"[IS] OR "0212-6109"[IS] OR "0044-5231"[IS] OR "0044-5231"[IS] OR "2590-2598"[IS] OR "2096-4501"[IS] OR "1467-6427"[IS] OR "0163-4445"[IS] OR "1877-5764"[IS] OR "1877-5756"[IS] OR "1471-678X"[IS] OR "1471-6798"[IS] OR "2520-1018"[IS] OR "2520-1018"[IS] OR "1936-6469"[IS] OR "0882-0783"[IS] OR "0193-7235"[IS] OR "1552-7638"[IS] OR "0023-6772"[IS] OR "1758-1117"[IS] OR "0169-4286"[IS] OR "1573-5095"[IS] OR "0925-3467"[IS] OR "0925-3467"[IS] OR "1477-2817"[IS] OR "1745-039X"[IS] OR "0012-2017"[IS] OR "1746-8361"[IS] OR "1386-4564"[IS] OR "1573-7659"[IS] OR "2095-0357"[IS] OR "2095-0357"[IS] OR "2405-8440"[IS] OR "2405-8440"[IS] OR "2515-964X"[IS] OR "2515-964X"[IS] OR "2199-4641"[IS] OR "2199-465X"[IS] OR "1544-2217"[IS] OR "0300-9858"[IS] OR "0002-0397"[IS] OR "0002-0397"[IS] OR "0307-9457"[IS] OR "1465-3338"[IS] OR "0966-8373"[IS] OR "0966-8373"[IS] OR "1098-612X"[IS] OR "1532-2750"[IS] OR "1177-889X"[IS] OR "1177-889X"[IS] OR "0722-4060"[IS] OR "1432-2056"[IS] OR "1521-0693"[IS] OR "1057-3569"[IS] OR "1015-3802"[IS] OR "1015-3802"[IS] OR "1532-6934"[IS] OR "1040-0419"[IS] OR "0730-9295"[IS] OR "2163-5226"[IS] OR "0075-4358"[IS] OR "0075-4358"[IS] OR "1470-1138"[IS] OR "0263-5143"[IS] OR "2666-7193"[IS] OR "2666-7193"[IS] OR "0959-4493"[IS] OR "1365-3164"[IS] OR "2514-8362"[IS] OR "2514-8362"[IS] OR "0268-6902"[IS] OR "0268-6902"[IS] OR "1869-5450"[IS] OR "1869-5469"[IS] OR "2075-5309"[IS] OR "2075-5309"[IS] OR "1531-4332"[IS] OR "1095-6433"[IS] OR "1760-2734"[IS] OR "1875-0834"[IS] OR "1447-2554"[IS] OR "1447-2546"[IS] OR "1072-0847"[IS] OR "1099-078X"[IS] OR "1366-8250"[IS] OR "1469-9532"[IS] OR "1552-6917"[IS] OR "1055-3290"[IS] OR "2058-5543"[IS] OR "2058-5543"[IS] OR "2673-1665"[IS] OR "2673-1673"[IS] OR "1618-1247"[IS] OR "1618-1255"[IS] OR "0080-6757"[IS] OR "1467-9477"[IS] OR "2364-8228"[IS] OR "2364-8228"[IS] OR "2183-2463"[IS] OR "2183-2463"[IS] OR "2197-0025"[IS] OR "2197-0025"[IS] OR "0011-5266"[IS] OR

	"1548-6192"[IS] OR "1439-0310"[IS] OR "0179-1613"[IS] OR "1793-6551"[IS] OR "0218-1274"[IS] OR "1744-795X"[IS] OR "0255-7614"[IS] OR "1007-662X"[IS] OR "1993-0607"[IS] OR "0276-3877"[IS] OR "0276-3877"[IS] OR "0176-1714"[IS] OR "1432-217X"[IS] OR "2078-0389"[IS] OR "2078-0397"[IS] OR "1793-6543"[IS] OR "0218-348X"[IS] OR "1735-1472"[IS] OR "1735-1472"[IS] OR "2229-0443"[IS] OR "2229-0443"[IS] OR "1750-4716"[IS] OR "1750-4708"[IS] OR "1460-2482"[IS] OR "0031-2290"[IS] OR "2336-2839"[IS] OR "2336-2839"[IS] OR "0268-2575"[IS] OR "1097-4660"[IS] OR "2158-9119"[IS] OR "0735-648X"[IS] OR "0362-2525"[IS] OR "1097-4687"[IS] OR "0002-0443"[IS] OR "1539-0721"[IS] OR "1868-6303"[IS] OR "1868-6311"[IS] OR "0365-0340"[IS] OR "1476-3567"[IS] OR "1365-2206"[IS] OR "1356-7500"[IS] OR "1994-036X"[IS] OR "1991-3761"[IS] OR "1068-5502"[IS] OR "1068-5502"[IS] OR "1432-1351"[IS] OR "0340-7594"[IS] OR "0161-6846"[IS] OR "1541-1540"[IS] OR "1473-3250"[IS] OR "1473-3250"[IS] OR "0936-5907"[IS] OR "0936-5907"[IS] OR "1748-8966"[IS] OR "1748-8958"[IS] OR "0144-333X"[IS] OR "1758-6720"[IS] OR "1880-6791"[IS] OR "1880-6805"[IS] OR "0929-6174"[IS] OR "0929-6174"[IS] OR "1099-1123"[IS] OR "1090-6738"[IS] OR "1537-7857"[IS] OR "1537-7865"[IS] OR "1042-8232"[IS] OR "1042-8232"[IS] OR "1573-6555"[IS] OR "0090-6905"[IS] OR "1559-6109"[IS] OR "1559-6109"[IS] OR "0026-1270"[IS] OR "2511-705X"[IS] OR "1432-1955"[IS] OR "0932-0113"[IS] OR "0887-8013"[IS] OR "1098-2825"[IS] OR "0360-4918"[IS] OR "0360-4918"[IS] OR "1469-5790"[IS] OR "0952-3987"[IS] OR "1938-8160"[IS] OR "1938-8179"[IS] OR "2116-7052"[IS] OR "1246-0125"[IS] OR "1408-6980"[IS] OR "1581-1980"[IS] OR "1557-055X"[IS] OR "1557-0541"[IS] OR "2559-8201"[IS] OR "2559-8201"[IS] OR "2095-2635"[IS] OR "2095-2635"[IS] OR "1361-4533"[IS] OR "1740-7834"[IS] OR "0092-8240"[IS] OR "1522-9602"[IS] OR "1469-3631"[IS] OR "1356-9775"[IS] OR "0743-5584"[IS] OR "1552-6895"[IS] OR "1018-2101"[IS] OR "1018-2101"[IS] OR "1573-7101"[IS] OR "0048-5829"[IS] OR "1063-6161"[IS] OR "1938-1581"[IS] OR "1556-3839"[IS] OR "0734-0168"[IS] OR "1558-3066"[IS] OR "1558-3058"[IS] OR "0889-4655"[IS] OR "1550-5049"[IS] OR "2154-3348"[IS] OR "2154-3348"[IS] OR "2515-690X"[IS] OR "2515-690X"[IS] OR "1532-7817"[IS] OR "1048-9223"[IS] OR "0002-9300"[IS] OR "0002-9300"[IS] OR "1071-4421"[IS] OR "1071-4421"[IS] OR "2059-5794"[IS] OR "2059-5794"[IS] OR "1110-1768"[IS] OR "1110-1768"[IS] OR "1934-9491"[IS] OR "1934-9556"[IS] OR "2666-1543"[IS] OR "2666-1543"[IS] OR "0964-6639"[IS] OR "1461-7390"[IS] OR "1367-0069"[IS] OR "1367-0069"[IS] OR "0916-8818"[IS] OR "1348-4400"[IS] OR "0002-1962"[IS] OR "1435-0645"[IS] OR "1944-7515"[IS] OR "1944-7558"[IS] OR "0250-5991"[IS] OR "0250-5991"[IS] OR "1049-7315"[IS] OR "1552-7581"[IS] OR "1074-8407"[IS] OR "1552-549X"[IS] OR "1522-2152"[IS] OR "1537-5293"[IS] OR "1076-8971"[IS] OR "1076-8971"[IS] OR "1084-4791"[IS] OR "1936-4830"[IS] OR "2050-2680"[IS] OR "2050-2680"[IS] OR "2589-1375"[IS] OR "2588-994X"[IS] OR "0368-0762"[IS] OR "2046-0481"[IS] OR "1470-1006"[IS] OR "0144-2872"[IS] OR "1476-8267"[IS] OR "1024-2694"[IS] OR "1360-6743"[IS] OR "1469-4379"[IS] OR "0157-6321"[IS] OR "0157-6321"[IS] OR "1351-1610"[IS] OR "1469-8412"[IS] OR "1868-4882"[IS] OR "1868-1034"[IS] OR "2059-7991"[IS] OR "2059-7991"[IS] OR "1890-2146"[IS] OR "1890-2138"[IS] OR "2046-1488"[IS] OR "2046-147X"[IS] OR "2769-3600"[IS] OR "2769-3589"[IS] OR "1940-1507"[IS] OR "1940-1493"[IS] OR "1340-3443"[IS] OR "1340-3443"[IS] OR "1083-446X"[IS] OR "1525-9153"[IS] OR "2075-4701"[IS] OR "2075-4701"[IS] OR "1751-1321"[IS] OR "1751-133X"[IS] OR "1879-0054"[IS] OR "0739-7240"[IS] OR "1023-7267"[IS] OR "1023-7267"[IS] OR "1570-1727"[IS] OR "1570-1727"[IS] OR "0038-0253"[IS] OR "1533-8525"[IS] OR "1467-8519"[IS] OR "0269-9702"[IS] OR "1386-1999"[IS] OR "1386-1999"[IS] OR "2673-3145"[IS] OR "2673-3145"[IS] OR "1549-8425"[IS] OR "1549-8417"[IS] OR "1469-8110"[IS] OR "1351-3249"[IS] OR "1086-3303"[IS] OR "1071-6076"[IS] OR "2045-709X"[IS] OR "2045-709X"[IS] OR "1535-1025"[IS] OR "1535-1025"[IS] OR "2153-3687"[IS] OR "2153-3687"[IS] OR "1532-2661"[IS] OR "0034-5288"[IS] OR "1322-7130"[IS] OR "1755-0238"[IS] OR "2095-7505"[IS] OR "2095-977X"[IS] OR "1741-4350"[IS] OR "1741-4350"[IS] OR "1743-9418"[IS] OR "1362-9395"[IS] OR "0334-5114"[IS] OR "1878-7665"[IS] OR "1099-1743"[IS] OR "1092-7026"[IS] OR "1878-1667"[IS] OR "0147-9571"[IS] OR "0276-2374"[IS] OR "0276-2374"[IS] OR "1057-5677"[IS] OR "1057-5677"[IS] OR "1475-4843"[IS] OR "1475-4835"[IS] OR "1545-696X"[IS] OR "0897-6546"[IS] OR "1309-422X"[IS] OR "2147-429X"[IS] OR "2052-3211"[IS] OR "2052-3211"[IS] OR "1527-6988"[IS] OR "1527-6988"[IS] OR "1610-2878"[IS] OR "1610-2878"[IS] OR "1550-1558"[IS] OR "1054-8289"[IS] OR "2475-0158"[IS] OR "2475-0166"[IS] OR "1479-9855"[IS] OR "1479-9855"[IS] OR "1939-2400"[IS] OR "1939-2397"[IS] OR "2297-900X"[IS] OR "2297-900X"[IS] OR "1469-9427"[IS] OR "1350-178X"[IS] OR "1744-988X"[IS] OR "1744-9871"[IS] OR "1879-0992"[IS] OR "0306-4565"[IS] OR "0013-7006"[IS] OR "0013-7006"[IS] OR "2338-557X"[IS] OR "0126-012X"[IS] OR "2146-7757"[IS] OR "2146-7757"[IS] OR "1099-0798"[IS] OR "0735-3936"[IS] OR "0306-624X"[IS] OR "1552-6933"[IS] OR "1594-4077"[IS] OR "1594-4077"[IS] OR "1558-7878"[IS] OR "1558-7878"[IS] OR "2618-8171"[IS] OR "2618-8163"[IS] OR "1521-0456"[IS] OR "0163-9625"[IS] OR "1878-013X"[IS] OR "1755-599X"[IS] OR "0915-1559"[IS] OR "0915-1559"[IS] OR "1931-437X"[IS] OR "1931-4361"[IS] OR "0944-2006"[IS] OR "0944-2006"[IS] OR "2633-4682"[IS] OR "2633-4690"[IS] OR "0010-096X"[IS] OR "0010-096X"[IS] OR "2059-4372"[IS] OR "2059-4372"[IS] OR "1751-9861"[IS] OR "1071-2089"[IS] OR "0165-5515"[IS] OR "1741-6485"[IS] OR "2059-5891"[IS] OR

	"2059-5891"[IS] OR "0003-8628"[IS] OR "1758-9622"[IS] OR "2673-3218"[IS] OR "2673-3218"[IS] OR
	"0021-8375"[IS] OR "1439-0361"[IS] OR "0967-5426"[IS] OR "0967-5426"[IS] OR "1540-3556"[IS] OR
	"1091-1359"[IS] OR "1552-650X"[IS] OR "0022-1678"[IS] OR "0194-2638"[IS] OR "1541-3144"[IS] OR
	"0002-0206"[IS] OR "0002-0206"[IS] OR "1745-1035"[IS] OR "1051-0974"[IS] OR "1666-2105"[IS] OR
	"1850-373X"[IS] OR "1532-8449"[IS] OR "0882-5963"[IS] OR "1095-8541"[IS] OR "0022-5193"[IS] OR
	"1611-3683"[IS] OR "1611-3683"[IS] OR "1689-765X"[IS] OR "2353-3293"[IS] OR "1741-2862"[IS] OR
	"0047-1178"[IS] OR "1740-5599"[IS] OR "0032-258X"[IS] OR "1432-119X"[IS] OR "0948-6143"[IS] OR
	"0217-2445"[IS] OR "0217-2445"[IS] OR "1469-901X"[IS] OR "0034-4125"[IS] OR "1768-1448"[IS] OR
	"1314-2615"[IS] OR "0008-4301"[IS] OR "1480-3283"[IS] OR "1478-6028"[IS] OR "1478-601X"[IS] OR
	"2190-5142"[IS] OR "1618-3169"[IS] OR "1088-7679"[IS] OR "1088-7679"[IS] OR "1476-7430"[IS] OR
	"1572-5138"[IS] OR "1638-9387"[IS] OR "1280-9551"[IS] OR "1741-6590"[IS] OR "1741-6604"[IS] OR
	"1479-1854"[IS] OR "1472-3891"[IS] OR "1740-7931"[IS] OR "0141-6200"[IS] OR "1479-5809"[IS] OR
	"1529-5036"[IS] OR "0955-792X"[IS] OR "1465-363X"[IS] OR "1467-9264"[IS] OR "0066-7374"[IS] OR
	"1477-3708"[IS] OR "1477-3708"[IS] OR "1474-2659"[IS] OR "1474-2640"[IS] OR "1556-2956"[IS] OR
	"1556-2948"[IS] OR "1463-4236"[IS] OR "1477-1128"[IS] OR "2452-2929"[IS] OR "2452-2929"[IS] OR
	"0378-4320"[IS] OR "1873-2232"[IS] OR "1572-8773"[IS] OR "0966-0844"[IS] OR "1879-1107"[IS] OR
	"1096-4959"[IS] OR "1525-822X"[IS] OR "1525-822X"[IS] OR "1741-6469"[IS] OR "0266-6669"[IS] OR
	"1461-9555"[IS] OR "1461-9563"[IS] OR "0043-7719"[IS] OR "1432-5225"[IS] OR "1940-4395"[IS] OR
	"1063-2913"[IS] OR "0920-1297"[IS] OR "1558-5263"[IS] OR "0278-0771"[IS] OR "0278-0771"[IS] OR
	"1755-0211"[IS] OR "1755-0203"[IS] OR "1035-0330"[IS] OR "1470-1219"[IS] OR "1096-4762"[IS] OR
	"1096-4762"[IS] OR "0140-2390"[IS] OR "1743-937X"[IS] OR "1751-0147"[IS] OR "0044-605X"[IS] OR
	"2383-3866"[IS] OR "2383-3572"[IS] OR "1744-7143"[IS] OR "1747-6615"[IS] OR "1572-8633"[IS] OR
	"1386-7423"[IS] OR "1573-5044"[IS] OR "0167-6857"[IS] OR "1552-6364"[IS] OR "0739-9863"[IS] OR
	"1432-1882"[IS] OR "0942-4962"[IS] OR "2472-5390"[IS] OR "2472-5390"[IS] OR "1522-2179"[IS] OR
	"1539-0705"[IS] OR "1097-0029"[IS] OR "1059-910X"[IS] OR "1474-7456"[IS] OR "1475-3138"[IS] OR
	"0891-2963"[IS] OR "1029-2381"[IS] OR "2311-8571"[IS] OR "2589-2894"[IS] OR "0922-1425"[IS] OR
	"0922-1425"[IS] OR "1529-9716"[IS] OR "1529-9716"[IS] OR "1350-4126"[IS] OR "1475-6811"[IS] OR
	"0167-9848"[IS] OR "0167-9848"[IS] OR "2398-4163"[IS] OR "2398-4155"[IS] OR "2322-4835"[IS] OR
	"2322-2476"[IS] OR "1467-9779"[IS] OR "1097-3923"[IS] OR "0023-8791"[IS] OR "1542-4278"[IS] OR
	"0002-8762"[IS] OR "1937-5239"[IS] OR "1389-0166"[IS] OR "1389-0166"[IS] OR "2190-8249"[IS] OR
	"1867-299X"[IS] OR "0263-2772"[IS] OR "0263-2772"[IS] OR "2050-7445"[IS] OR "2050-7445"[IS] OR
	"1935-4932"[IS] OR "1935-4940"[IS] OR "2326-4519"[IS] OR "2326-4500"[IS] OR "0034-527X"[IS] OR
	"0034-527X"[IS] OR "1469-2953"[IS] OR "1463-1369"[IS] OR "0966-8136"[IS] OR "1465-3427"[IS] OR
	"1477-2744"[IS] OR "1068-316X"[IS] OR "1527-2133"[IS] OR "0003-1283"[IS] OR "2049-7636"[IS] OR
	"1528-8870"[IS] OR "0967-0769"[IS] OR "1464-3693"[IS] OR "2352-6181"[IS] OR "2352-6181"[IS] OR
	"0888-0395"[IS] OR "0888-0395"[IS] OR "2059-2043"[IS] OR "2059-2043"[IS] OR "1614-2942"[IS] OR
	"1614-2950"[IS] OR "1533-7731"[IS] OR "0893-5394"[IS] OR "2515-1096"[IS] OR "2515-1088"[IS] OR
	"2047-7414"[IS] OR "2047-7422"[IS] OR "1703-4949"[IS] OR "1703-4949"[IS] OR "0896-6346"[IS] OR
	"1305-3299"[IS] OR "2398-628X"[IS] OR "2398-6298"[IS] OR "2353-883X"[IS] OR "2353-8821"[IS] OR
	"1556-4711"[IS] OR "1556-4673"[IS] OR "0008-4506"[IS] OR "0008-4506"[IS] OR "1461-6742"[IS] OR
	"1461-6742"[IS] OR "1018-3647"[IS] OR "1018-3647"[IS] OR "1174-5398"[IS] OR "2159-6816"[IS] OR
	"2044-0847"[IS] OR "2044-0839"[IS] OR "1741-3079"[IS] OR "0264-5505"[IS] OR "1748-2631"[IS] OR
	"1748-2623"[IS] OR "0963-9314"[IS] OR "1573-1367"[IS] OR "2373-700X"[IS] OR "2373-6992"[IS] OR
	"2044-124X"[IS] OR "2044-1258"[IS] OR "2632-7627"[IS] OR "2632-7627"[IS] OR "1572-8374"[IS] OR
	"0920-427X"[IS] OR "2324-8823"[IS] OR "2324-8831"[IS] OR "2199-9023"[IS] OR "2199-9031"[IS] OR
	"0266-0784"[IS] OR "0266-0784"[IS] OR "1611-8014"[IS] OR "1611-8014"[IS] OR "0015-7120"[IS] OR
	"0015-7120"[IS] OR "1530-9177"[IS] OR "0163-660X"[IS] OR "1741-2730"[IS] OR "1474-8851"[IS] OR
	"2211-3770"[IS] OR "2211-3789"[IS] OR "0958-0344"[IS] OR "1099-1565"[IS] OR "0887-3631"[IS] OR
	"0887-3631"[IS] OR "0925-8531"[IS] OR "0925-8531"[IS] OR "1572-8730"[IS] OR "0039-3215"[IS] OR
	"1536-1098"[IS] OR "1536-1098"[IS] OR "0304-4092"[IS] OR "1573-0786"[IS] OR "1943-6149"[IS] OR
	"1943-6157"[IS] OR "2049-5838"[IS] OR "2049-5846"[IS] OR "0028-7881"[IS] OR "0028-7881"[IS] OR
	"2014-3680"[IS] OR "2014-3680"[IS] OR "1064-5578"[IS] OR "1934-7715"[IS] OR "0067-270X"[IS] OR
	"1945-5534"[IS] OR "2158-205X"[IS] OR "2158-2041"[IS] OR "2573-5586"[IS] OR "0738-2898"[IS] OR
	"1871-1413"[IS] OR "1871-1413"[IS] OR "0100-879X"[IS] OR "1414-431X"[IS] OR "2187-9036"[IS] OR
	"2187-9036"[IS] OR "1710-1115"[IS] OR "0008-4131"[IS] OR "0929-1873"[IS] OR "1573-8469"[IS] OR
	"0144-3585"[IS] OR "0144-3585"[IS] OR "0001-9720"[IS] OR "1750-0184"[IS] OR "2165-8692"[IS] OR
	"2165-8684"[IS] OR "0019-042X"[IS] OR "0019-042X"[IS] OR "2165-0357"[IS] OR "2165-0349"[IS] OR
	"1879-9817"[IS] OR "1879-9817"[IS] OR "1750-2977"[IS] OR "1750-2985"[IS] OR "1573-7446"[IS] OR
	"0165-7380"[IS] OR "2494-1271"[IS] OR "2494-1271"[IS] OR "0025-1003"[IS] OR "0025-1003"[IS] OR
	"2306-7381"[IS] OR "2306-7381"[IS] OR "0206-3735"[IS] OR "0206-3735"[IS] OR "1187-7863"[IS] OR

	"1187-7863"[IS] OR "2051-2201"[IS] OR "1355-8250"[IS] OR "1532-771X"[IS] OR "1534-8431"[IS] OR "2195-7819"[IS] OR "2195-7819"[IS] OR "1944-8260"[IS] OR "0809-8131"[IS] OR "1439-0221"[IS] OR "0032-0943"[IS] OR "2332-2705"[IS] OR "2332-2713"[IS] OR "1086-3168"[IS] OR "0002-9475"[IS] OR "1360-3124"[IS] OR "1464-5092"[IS] OR "1749-6039"[IS] OR "1749-6020"[IS] OR "1750-6352"[IS] OR "1750-6360"[IS] OR "2161-2234"[IS] OR "2161-2234"[IS] OR "2639-3832"[IS] OR "2639-3832"[IS] OR "1752-928X"[IS] OR "1752-928X"[IS] OR "1478-2189"[IS] OR "1478-2189"[IS] OR "1552-7441"[IS] OR "0048-3931"[IS] OR "1854-2492"[IS] OR "1408-967X"[IS] OR "1476-8275"[IS] OR "0300-4430"[IS] OR "1488-3473"[IS] OR "1488-3473"[IS] OR "2752-7549"[IS] OR "2752-7530"[IS] OR "1470-5958"[IS] OR "1741-2838"[IS] OR "1612-1651"[IS] OR "2567-6911"[IS] OR "0932-0814"[IS] OR "1032-3732"[IS] OR "1749-3374"[IS] OR "0066-1546"[IS] OR "0066-1546"[IS] OR "2300-9802"[IS] OR "1425-3305"[IS] OR "0960-3182"[IS] OR "1573-1529"[IS] OR "2322-1291"[IS] OR "2322-1291"[IS] OR "1942-258X"[IS] OR "1476-6086"[IS] OR "1537-5366"[IS] OR "0047-2530"[IS] OR "1060-8265"[IS] OR "1933-0251"[IS] OR "2326-8743"[IS] OR "2326-8751"[IS] OR "1359-1835"[IS] OR "1460-3586"[IS] OR "1359-7566"[IS] OR "1359-7566"[IS] OR "0883-024X"[IS] OR "0883-024X"[IS] OR "2280-1855"[IS] OR "2280-1855"[IS] OR "1469-5936"[IS] OR "0967-2567"[IS] OR "2199-174X"[IS] OR "2199-174X"[IS] OR "0031-8868"[IS] OR "1568-5284"[IS] OR "0267-0844"[IS] OR "0267-0844"[IS] OR "0042-2533"[IS] OR "0042-2533"[IS] OR "1468-1099"[IS] OR "1474-0060"[IS] OR "1081-6976"[IS] OR "1555-2934"[IS] OR "2468-2276"[IS] OR "2468-2276"[IS] OR "2399-2050"[IS] OR "2052-6113"[IS] OR "1079-8986"[IS] OR "1079-8986"[IS] OR "1573-5060"[IS] OR "0014-2336"[IS] OR "1879-9272"[IS] OR "1879-9264"[IS] OR "1533-6263"[IS] OR "0302-1475"[IS] OR "0008-1221"[IS] OR "0008-1221"[IS] OR "1465-3885"[IS] OR "0265-0533"[IS] OR "2375-9240"[IS] OR "2375-9267"[IS] OR "2206-3374"[IS] OR "2206-3374"[IS] OR "0075-8914"[IS] OR "1756-3801"[IS] OR "1735-5362"[IS] OR "1735-9414"[IS] OR "0019-4964"[IS] OR "0970-4140"[IS] OR "0018-9855"[IS] OR "2195-0237"[IS] OR "1528-6916"[IS] OR "0885-4726"[IS] OR "1357-2334"[IS] OR "1743-9337"[IS] OR "1463-5224"[IS] OR "1463-5216"[IS] OR "0007-9871"[IS] OR "0007-9871"[IS] OR "1755-0483"[IS] OR "1755-0483"[IS] OR "0734-578X"[IS] OR "0734-578X"[IS] OR "1827-4765"[IS] OR "2037-0644"[IS] OR "0048-0169"[IS] OR "1176-0710"[IS] OR "0742-1656"[IS] OR "0742-1656"[IS] OR "1885-642X"[IS] OR "1886-3655"[IS] OR "1558-0989"[IS] OR "1099-9922"[IS] OR "1462-3943"[IS] OR "1470-1103"[IS] OR "0884-2175"[IS] OR "1552-6909"[IS] OR "0045-5091"[IS] OR "1911-0820"[IS] OR "1574-020X"[IS] OR "1574-020X"[IS] OR "0963-7494"[IS] OR "0963-7494"[IS] OR "2151-6952"[IS] OR "2151-6952"[IS] OR "1569-7274"[IS] OR "1540-3009"[IS] OR "2672-0191"[IS] OR "2055-0391"[IS] OR "2152-906X"[IS] OR "2152-9078"[IS] OR "2242-4075"[IS] OR "0037-5330"[IS] OR "2210-7711"[IS] OR "2210-7703"[IS] OR "0269-1728"[IS] OR "1464-5297"[IS] OR "1573-6725"[IS] OR "0885-7466"[IS] OR "1469-6053"[IS] OR "1469-6053"[IS] OR "0022-5053"[IS] OR "1538-4586"[IS] OR "0024-3949"[IS] OR "1613-396X"[IS] OR "1740-0228"[IS] OR "1740-0236"[IS] OR "0003-1062"[IS] OR "0003-1062"[IS] OR "1470-594X"[IS] OR "1470-594X"[IS] OR "1600-0498"[IS] OR "0008-8994"[IS] OR "0196-8599"[IS] OR "0196-8599"[IS] OR "0303-6758"[IS] OR "0303-6758"[IS] OR "1743-9590"[IS] OR "1466-0970"[IS] OR "2056-9017"[IS] OR "2056-9017"[IS] OR "2311-7524"[IS] OR "2311-7524"[IS] OR "0269-7580"[IS] OR "0269-7580"[IS] OR "0892-7936"[IS] OR "0892-7936"[IS] OR "2610-9182"[IS] OR "2610-9182"[IS] OR "2548-0839"[IS] OR "2548-0839"[IS] OR "1524-9042"[IS] OR "1532-8635"[IS] OR "1744-6880"[IS] OR "1744-6872"[IS] OR "1557-3087"[IS] OR "1557-2013"[IS] OR "2326-3768"[IS] OR "2326-3768"[IS] OR "0958-4935"[IS] OR "1469-364X"[IS] OR "1572-8552"[IS] OR "1383-4924"[IS] OR "0569-9878"[IS] OR "1988-8325"[IS] OR "2045-3825"[IS] OR "2045-3817"[IS] OR "1532-7868"[IS] OR "1092-6488"[IS] OR "2049-9434"[IS] OR "2049-9442"[IS] OR "1707-7753"[IS] OR "1911-0219"[IS] OR "0270-2711"[IS] OR "1521-0685"[IS] OR "2215-0390"[IS] OR "2215-0390"[IS] OR "1741-6205"[IS] OR "1566-7529"[IS] OR "1871-191X"[IS] OR "1871-1901"[IS] OR "0034-0561"[IS] OR "0034-0561"[IS] OR "2333-4509"[IS] OR "1607-8055"[IS] OR "2211-3460"[IS] OR "2211-3452"[IS] OR "2191-4281"[IS] OR "2193-567X"[IS] OR "0045-0766"[IS] OR "1440-1630"[IS] OR "1362-704X"[IS] OR "1362-704X"[IS] OR "0266-2671"[IS] OR "1474-0028"[IS] OR "0024-3841"[IS] OR "0024-3841"[IS] OR "2355-6994"[IS] OR "1978-6301"[IS] OR "1108-9628"[IS] OR "1108-9628"[IS] OR "1550-3259"[IS] OR "0271-8294"[IS] OR "0003-0481"[IS] OR "0003-0481"[IS] OR "1613-3668"[IS] OR "0165-2516"[IS] OR "2588-9338"[IS] OR "2588-9338"[IS] OR "2246-8498"[IS] OR "2246-8498"[IS] OR "0031-8388"[IS] OR "1423-0321"[IS] OR "1743-0445"[IS] OR "1743-0437"[IS] OR "0312-2417"[IS] OR "0312-2417"[IS] OR "1736-7484"[IS] OR "1406-2933"[IS] OR "1477-111X"[IS] OR "0267-6591"[IS] OR "1983-3121"[IS] OR "0034-7329"[IS] OR "1475-3073"[IS] OR "1474-7464"[IS] OR "1879-4920"[IS] OR "1879-4912"[IS] OR "1939-795X"[IS] OR "1939-795X"[IS] OR "1044-2073"[IS] OR "1044-2073"[IS] OR "1836-9324"[IS] OR "1836-9324"[IS] OR "1569-9811"[IS] OR "1384-6655"[IS] OR "1538-9855"[IS] OR "0363-3624"[IS] OR "0976-3457"[IS] OR "0973-9572"[IS] OR "1872-0218"[IS] OR "1872-0226"[IS] OR "1613-3722"[IS] OR "0933-1719"[IS] OR "1573-1782"[IS] OR "1873-0388"[IS] OR "0002-9114"[IS] OR "0002-9114"[IS] OR "1746-1545"[IS] OR "0966-2839"[IS] OR "2051-5553"[IS] OR "2051-5561"[IS] OR "1569-9862"[IS] OR "1569-2159"[IS] OR "1753-9129"[IS] OR "1753-9137"[IS] OR "1742-058X"[IS] OR "1742-058X"[IS] OR "1868-4890"[IS] OR "1866-802X"[IS] OR

	"1989-9947"[IS] OR "1989-9947"[IS] OR "1578-4118"[IS] OR "1135-6405"[IS] OR "2589-207X"[IS] OR "2589-2053"[IS] OR "0728-4896"[IS] OR "1834-4453"[IS] OR "1535-0770"[IS] OR "1535-0770"[IS] OR "2352-2208"[IS] OR "2352-2216"[IS] OR "2230-6862"[IS] OR "2230-6862"[IS] OR "1741-2633"[IS] OR "1059-7123"[IS] OR "1079-2082"[IS] OR "1535-2900"[IS] OR "1466-8203"[IS] OR "1466-8203"[IS] OR "1556-2646"[IS] OR "1556-2654"[IS] OR "2156-7093"[IS] OR "0003-6390"[IS] OR "0022-2917"[IS] OR "0022-2917"[IS] OR "1467-9752"[IS] OR "0309-8249"[IS] OR "0029-3652"[IS] OR "0029-3652"[IS] OR "1932-2798"[IS] OR "1876-2700"[IS] OR "1034-5329"[IS] OR "2206-9542"[IS] OR "2198-0802"[IS] OR "2198-0802"[IS] OR "1445-6354"[IS] OR "1445-6354"[IS] OR "2212-5892"[IS] OR "2212-5884"[IS] OR "1744-9359"[IS] OR "1744-9367"[IS] OR "0029-8115"[IS] OR "0029-8115"[IS] OR "0034-673X"[IS] OR "0034-673X"[IS] OR "2167-6437"[IS] OR "0022-2984"[IS] OR "1086-3249"[IS] OR "1054-6863"[IS] OR "2468-6042"[IS] OR "2468-6034"[IS] OR "0928-1371"[IS] OR "0928-1371"[IS] OR "1715-2593"[IS] OR "1715-2593"[IS] OR "2405-6618"[IS] OR "2405-6618"[IS] OR "2195-2701"[IS] OR "2195-2701"[IS] OR "0032-0447"[IS] OR "0032-0447"[IS] OR "1931-7743"[IS] OR "1557-0274"[IS] OR "1698-7454"[IS] OR "1698-7454"[IS] OR "1544-3191"[IS] OR "1544-3450"[IS] OR "0029-8077"[IS] OR "0029-8077"[IS] OR "0031-9465"[IS] OR "1593-2095"[IS] OR "0967-201X"[IS] OR "1752-2285"[IS] OR "2387-4562"[IS] OR "2387-4562"[IS] OR "1878-6553"[IS] OR "1878-6561"[IS] OR "0161-4754"[IS] OR "1532-6586"[IS] OR "0952-9136"[IS] OR "0952-9136"[IS] OR "2363-5150"[IS] OR "2363-5142"[IS] OR "2157-6475"[IS] OR "0192-4036"[IS] OR "1045-6635"[IS] OR "2325-5080"[IS] OR "2359-5639"[IS] OR "2359-5639"[IS] OR "1537-2073"[IS] OR "1537-2073"[IS] OR "2191-9216"[IS] OR "2191-933X"[IS] OR "2059-1101"[IS] OR "2059-1098"[IS] OR "1035-7823"[IS] OR "1467-8403"[IS] OR "1382-4554"[IS] OR "1382-4554"[IS] OR "1740-4681"[IS] OR "1745-5243"[IS] OR "1578-7559"[IS] OR "1578-7559"[IS] OR "0973-0796"[IS] OR "0971-7218"[IS] OR "1899-5276"[IS] OR "2451-2680"[IS] OR "1099-1212"[IS] OR "1047-482X"[IS] OR "1469-2910"[IS] OR "1364-8470"[IS] OR "1877-1297"[IS] OR "1877-1297"[IS] OR "1478-1603"[IS] OR "1461-3557"[IS] OR "0047-2328"[IS] OR "0047-2328"[IS] OR "2514-3298"[IS] OR "2514-3298"[IS] OR "0091-4509"[IS] OR "0091-4509"[IS] OR "1754-8845"[IS] OR "0425-0494"[IS] OR "1464-374X"[IS] OR "0952-8873"[IS] OR "0039-3681"[IS] OR "1879-2510"[IS] OR "0886-6708"[IS] OR "0886-6708"[IS] OR "1470-3572"[IS] OR "1741-3214"[IS] OR "2363-5169"[IS] OR "2363-5169"[IS] OR "0269-9206"[IS] OR "1464-5076"[IS] OR "1747-633X"[IS] OR "1747-6321"[IS] OR "2542-3851"[IS] OR "2542-386X"[IS] OR "1938-8071"[IS] OR "1938-8063"[IS] OR "1692-6013"[IS] OR "2500-8692"[IS] OR "2014-7910"[IS] OR "2014-7910"[IS] OR "0018-098X"[IS] OR "1553-5622"[IS] OR "1746-6660"[IS] OR "1746-6660"[IS] OR "2045-0044"[IS] OR "0263-3264"[IS] OR "0323-1267"[IS] OR "0323-1267"[IS] OR "1741-2714"[IS] OR "1466-1381"[IS] OR "1750-6999"[IS] OR "1750-6980"[IS] OR "0026-749X"[IS] OR "1469-8099"[IS] OR "1642-1027"[IS] OR "1642-1027"[IS] OR "1754-3266"[IS] OR "1754-3274"[IS] OR "2159-4953"[IS] OR "2159-4953"[IS] OR "2470-1483"[IS] OR "2470-1475"[IS] OR "2376-4538"[IS] OR "0264-6811"[IS] OR "2334-3745"[IS] OR "2334-3745"[IS] OR "1406-4243"[IS] OR "1736-7409"[IS] OR "1465-7260"[IS] OR "1465-7252"[IS] OR "0084-6724"[IS] OR "1573-6121"[IS] OR "0147-8257"[IS] OR "0147-8257"[IS] OR "2166-3548"[IS] OR "2166-3556"[IS] OR "1755-6678"[IS] OR "1755-6686"[IS] OR "0276-2080"[IS] OR "2154-154X"[IS] OR "1000-131X"[IS] OR "1000-131X"[IS] OR "1563-0110"[IS] OR "1563-0110"[IS] OR "1758-8871"[IS] OR "1477-996X"[IS] OR "1473-4265"[IS] OR "1468-215X"[IS] OR "2051-364X"[IS] OR "2051-3658"[IS] OR "1748-1317"[IS] OR "1748-1317"[IS] OR "1759-0450"[IS] OR "1759-0442"[IS] OR "1095-5143"[IS] OR "1095-5143"[IS] OR "2165-2627"[IS] OR "2165-2627"[IS] OR "0004-7554"[IS] OR "1549-4950"[IS] OR "1421-9972"[IS] OR "1021-7762"[IS] OR "1743-1301"[IS] OR "0031-0328"[IS] OR "1839-339X"[IS] OR "0814-3021"[IS] OR "1750-3280"[IS] OR "1750-3299"[IS] OR "1566-7170"[IS] OR "1573-7098"[IS] OR "2255-3924"[IS] OR "1133-4525"[IS] OR "0003-5491"[IS] OR "1534-1518"[IS] OR "0121-5612"[IS] OR "0121-5612"[IS] OR "1946-6560"[IS] OR "1946-6579"[IS] OR "2215-1354"[IS] OR "2215-1362"[IS] OR "1360-0869"[IS] OR "1364-6885"[IS] OR "0007-6805"[IS] OR "2044-768X"[IS] OR "2386-7876"[IS] OR "0214-0039"[IS] OR "1461-7056"[IS] OR "1354-067X"[IS] OR "1476-8062"[IS] OR "1476-8070"[IS] OR "1799-649X"[IS] OR "1799-649X"[IS] OR "1878-9722"[IS] OR "1878-9714"[IS] OR "1743-1700"[IS] OR "0952-7648"[IS] OR "0378-4177"[IS] OR "0378-4177"[IS] OR "0161-6463"[IS] OR "0161-6463"[IS] OR "0021-9118"[IS] OR "0021-9118"[IS] OR "2364-6861"[IS] OR "2364-6861"[IS] OR "2049-1115"[IS] OR "2575-1433"[IS] OR "1369-8249"[IS] OR "1743-968X"[IS] OR "1076-0431"[IS] OR "1943-5568"[IS] OR "0041-5650"[IS] OR "0041-5650"[IS] OR "2407-8646"[IS] OR "1412-4734"[IS] OR "1746-918X"[IS] OR "1746-9198"[IS] OR "0959-2695"[IS] OR "0959-2695"[IS] OR "1068-8471"[IS] OR "1068-8471"[IS] OR "1539-0683"[IS] OR "0361-929X"[IS] OR "1358-684X"[IS] OR "1358-684X"[IS] OR "0363-6550"[IS] OR "1475-4975"[IS] OR "1467-9329"[IS] OR "0034-0006"[IS] OR "0277-5395"[IS] OR "0277-5395"[IS] OR "1369-8230"[IS] OR "1743-8772"[IS] OR "1081-602X"[IS] OR "1081-602X"[IS] OR "0003-0082"[IS] OR "0003-0082"[IS] OR "1572-851X"[IS] OR "0163-8548"[IS] OR "2064-5147"[IS] OR "2064-5031"[IS] OR "1206-3312"[IS] OR "1206-3312"[IS] OR "1743-7938"[IS] OR "0007-6791"[IS] OR "1027-6084"[IS] OR "1027-6084"[IS] OR "0271-5309"[IS] OR "0271-5309"[IS] OR "1938-2383"[IS] OR "1539-4492"[IS] OR "0016-8076"[IS] OR "0016-8076"[IS] OR "1381-2416"[IS] OR "1572-8110"[IS] OR

	"1537-7938"[IS] OR "1537-7938"[IS] OR "1744-5213"[IS] OR "0964-704X"[IS] OR "0265-9646"[IS] OR "1879-338X"[IS] OR "2090-3995"[IS] OR "2090-3987"[IS] OR "1958-5780"[IS] OR "1166-3081"[IS] OR "1989-7553"[IS] OR "1135-5948"[IS] OR "2071-8322"[IS] OR "2071-8322"[IS] OR "2042-1818"[IS] OR "0025-8024"[IS] OR "2476-1028"[IS] OR "2476-1036"[IS] OR "0075-4242"[IS] OR "1552-5457"[IS] OR "2153-9588"[IS] OR "2153-957X"[IS] OR "1555-5550"[IS] OR "1555-5542"[IS] OR "0091-2131"[IS] OR "1548-1352"[IS] OR "2325-4815"[IS] OR "2325-4823"[IS] OR "2559-9283"[IS] OR "2559-9283"[IS] OR "2180-2483"[IS] OR "1675-8110"[IS] OR "2153-960X"[IS] OR "2153-9596"[IS] OR "1942-8537"[IS] OR "0041-9907"[IS] OR "0036-6803"[IS] OR "1949-8594"[IS] OR "1468-3849"[IS] OR "1468-3849"[IS] OR "1057-0314"[IS] OR "1057-0314"[IS] OR "2325-3290"[IS] OR "2325-3290"[IS] OR "0883-8534"[IS] OR "0883-8534"[IS] OR "0026-2234"[IS] OR "0026-2234"[IS] OR "1875-1342"[IS] OR "1875-1350"[IS] OR "1368-9894"[IS] OR "1367-0751"[IS] OR "1470-1111"[IS] OR "1464-7893"[IS] OR "1473-2254"[IS] OR "1473-2254"[IS] OR "1467-5986"[IS] OR "1469-8439"[IS] OR "1063-4304"[IS] OR "1047-7594"[IS] OR "1229-1374"[IS] OR "1229-1374"[IS] OR "1464-3820"[IS] OR "0143-6503"[IS] OR "0938-5428"[IS] OR "1464-3596"[IS] OR "2363-9075"[IS] OR "2363-9075"[IS] OR "1876-0104"[IS] OR "1613-7272"[IS] OR "0001-8368"[IS] OR "0001-8368"[IS] OR "1740-469X"[IS] OR "0308-5759"[IS] OR "0037-7686"[IS] OR "1461-7404"[IS] OR "2192-4007"[IS] OR "2192-4007"[IS] OR "1461-7447"[IS] OR "0959-3543"[IS] OR "0739-7046"[IS] OR "2153-3393"[IS] OR "2160-486X"[IS] OR "2160-4851"[IS] OR "1744-0572"[IS] OR "1744-0572"[IS] OR "1538-974X"[IS] OR "1063-3685"[IS] OR "2153-599X"[IS] OR "2153-5981"[IS] OR "2190-3387"[IS] OR "2190-3387"[IS] OR "2408-0071"[IS] OR "2407-9529"[IS] OR "0388-0001"[IS] OR "0388-0001"[IS] OR "2044-1274"[IS] OR "2044-1266"[IS] OR "1934-5267"[IS] OR "1934-5267"[IS] OR "1744-2222"[IS] OR "1744-2230"[IS] OR "0716-1530"[IS] OR "0718-6894"[IS] OR "1360-0834"[IS] OR "1360-0834"[IS] OR "2210-2116"[IS] OR "2210-2124"[IS] OR "1989-9904"[IS] OR "0213-2338"[IS] OR "2042-5961"[IS] OR "2042-597X"[IS] OR "1933-8139"[IS] OR "0066-6939"[IS] OR "0960-8788"[IS] OR "0960-8788"[IS] OR "1461-7331"[IS] OR "0031-322X"[IS] OR "1573-5664"[IS] OR "1573-5664"[IS] OR "1577-4015"[IS] OR "2014-0983"[IS] OR "1099-0763"[IS] OR "1075-2196"[IS] OR "1361-7672"[IS] OR "1469-9362"[IS] OR "1657-0790"[IS] OR "2256-5760"[IS] OR "1875-8533"[IS] OR "1570-5838"[IS] OR "1092-7697"[IS] OR "1573-7748"[IS] OR "0264-3758"[IS] OR "0264-3758"[IS] OR "0718-3437"[IS] OR "0716-0747"[IS] OR "2050-5728"[IS] OR "2050-571X"[IS] OR "1175-8708"[IS] OR "1175-8708"[IS] OR "1752-6302"[IS] OR "1752-6299"[IS] OR "1465-3923"[IS] OR "0090-5992"[IS] OR "0942-2919"[IS] OR "0942-2919"[IS] OR "0012-7086"[IS] OR "0012-7086"[IS] OR "0020-7047"[IS] OR "0020-7047"[IS] OR "1476-6728"[IS] OR "0309-0892"[IS] OR "1758-7751"[IS] OR "1751-1348"[IS] OR "1934-1687"[IS] OR "1321-8719"[IS] OR "1131-6993"[IS] OR "1988-2327"[IS] OR "2574-9870"[IS] OR "2574-9870"[IS] OR "1934-3876"[IS] OR "0021-9231"[IS] OR "0265-8240"[IS] OR "1467-9930"[IS] OR "1756-7505"[IS] OR "1756-7513"[IS] OR "2009-7379"[IS] OR "2009-7379"[IS] OR "0039-3193"[IS] OR "0039-3193"[IS] OR "1539-7858"[IS] OR "0093-1896"[IS] OR "1467-2227"[IS] OR "1467-2235"[IS] OR "0734-6875"[IS] OR "2053-7387"[IS] OR "1468-5914"[IS] OR "0021-8308"[IS] OR "1369-6815"[IS] OR "1369-6815"[IS] OR "2666-5557"[IS] OR "2666-5557"[IS] OR "2325-8039"[IS] OR "0039-3541"[IS] OR "0066-6742"[IS] OR "1988-3110"[IS] OR "1751-0708"[IS] OR "1751-0694"[IS] OR "1095-8614"[IS] OR "0305-7488"[IS] OR "1751-3065"[IS] OR "1751-3057"[IS] OR "1479-2451"[IS] OR "1479-2443"[IS] OR "0065-4124"[IS] OR "0065-4124"[IS] OR "1365-7127"[IS] OR "1740-5572"[IS] OR "2149-1291"[IS] OR "2149-1291"[IS] OR "1096-1151"[IS] OR "0048-721X"[IS] OR "2152-2820"[IS] OR "2152-2820"[IS] OR "2466-1511"[IS] OR "1738-3102"[IS] OR "2159-1229"[IS] OR "0886-5655"[IS] OR "1557-2293"[IS] OR "1557-2285"[IS] OR "2542-3835"[IS] OR "2542-3843"[IS] OR "0514-7336"[IS] OR "0514-7336"[IS] OR "1038-1562"[IS] OR "1038-1562"[IS] OR "2334-9050"[IS] OR "2334-9050"[IS] OR "2514-9369"[IS] OR "2514-9369"[IS] OR "1055-1360"[IS] OR "1055-1360"[IS] OR "2162-2752"[IS] OR "1948-9137"[IS] OR "2153-9561"[IS] OR "2153-9553"[IS] OR "1386-2820"[IS] OR "1572-8447"[IS] OR "2032-6904"[IS] OR "2032-6912"[IS] OR "1552-8014"[IS] OR "1552-8014"[IS] OR "1095-9270"[IS] OR "1057-2414"[IS] OR "1556-3693"[IS] OR "1556-3693"[IS] OR "1067-3040"[IS] OR "1067-3040"[IS] OR "0262-5253"[IS] OR "1468-0092"[IS] OR "0886-571X"[IS] OR "0886-571X"[IS] OR "1077-727X"[IS] OR "1552-3934"[IS] OR "0968-5650"[IS] OR "1474-0052"[IS] OR "2056-6530"[IS] OR "2056-6522"[IS] OR "2210-5832"[IS] OR "2210-5824"[IS] OR "1052-0147"[IS] OR "1052-0147"[IS] OR "2210-4097"[IS] OR "2210-4070"[IS] OR "2211-4742"[IS] OR "2211-4750"[IS] OR "0964-9069"[IS] OR "0964-9069"[IS] OR "2192-7669"[IS] OR "0075-6334"[IS] OR "1575-9563"[IS] OR "1575-9563"[IS] OR "1853-8126"[IS] OR "0327-5159"[IS] OR "0155-0640"[IS] OR "0155-0640"[IS] OR "1468-0386"[IS] OR "1351-5993"[IS] OR "0440-9213"[IS] OR "0440-9213"[IS] OR "0929-1261"[IS] OR "1572-9990"[IS] OR "1934-9645"[IS] OR "1934-9637"[IS] OR "2300-6560"[IS] OR "2300-6560"[IS] OR "2055-7671"[IS] OR "2055-768X"[IS] OR "1916-5781"[IS] OR "1916-5781"[IS] OR "0023-656X"[IS] OR "1469-9702"[IS] OR "0145-5532"[IS] OR "1527-8034"[IS] OR "1516-3210"[IS] OR "1984-4182"[IS] OR "1864-1385"[IS] OR "1864-1385"[IS] OR "1094-2076"[IS] OR "2325-5404"[IS] OR "0165-0750"[IS] OR "0165-0750"[IS] OR "1540-8558"[IS] OR "1050-9674"[IS] OR "2011-799X"[IS] OR "2011-799X"[IS] OR "1429-2173"[IS] OR "2545-3971"[IS] OR "2329-2210"[IS] OR "2329-2210"[IS] OR

	"1600-0471"[IS] OR "0905-7196"[IS] OR "0165-4004"[IS] OR "1614-7308"[IS] OR "1527-1919"[IS] OR "0018-2702"[IS] OR "2196-9663"[IS] OR "2196-9663"[IS] OR "0002-1482"[IS] OR "0002-1482"[IS] OR "0003-5521"[IS] OR "0003-5521"[IS] OR "1900-5407"[IS] OR "2011-4273"[IS] OR "0326-7911"[IS] OR "2250-7728"[IS] OR "1988-3056"[IS] OR "1137-0734"[IS] OR "2002-0104"[IS] OR "2002-0104"[IS] OR "2355-6145"[IS] OR "0215-0492"[IS] OR "1879-5382"[IS] OR "1879-5382"[IS] OR "1474-0222"[IS] OR "1474-0222"[IS] OR "2054-7668"[IS] OR "2054-7676"[IS] OR "1989-872X"[IS] OR "2530-0024"[IS] OR "1745-8927"[IS] OR "1745-8935"[IS] OR "1072-4303"[IS] OR "1072-4303"[IS] OR "0955-5803"[IS] OR "0955-5803"[IS] OR "2392-2397"[IS] OR "2392-2397"[IS] OR "1612-3093"[IS] OR "1863-9038"[IS] OR "1877-6531"[IS] OR "1877-6531"[IS] OR "0029-3571"[IS] OR "0029-3571"[IS] OR "2549-3167"[IS] OR "2549-3132"[IS] OR "2053-6712"[IS] OR "2053-6712"[IS] OR "1697-0381"[IS] OR "1697-0381"[IS] OR "0006-8047"[IS] OR "0006-8047"[IS] OR "0010-4175"[IS] OR "1475-2999"[IS] OR "2053-5368"[IS] OR "2053-535X"[IS] OR "1573-4994"[IS] OR "1053-0509"[IS] OR "1889-4178"[IS] OR "1989-9335"[IS] OR "0090-8320"[IS] OR "1521-0642"[IS] OR "0718-3399"[IS] OR "0718-3399"[IS] OR "1652-8670"[IS] OR "1652-8670"[IS] OR "1387-6740"[IS] OR "1387-6740"[IS] OR "0258-3690"[IS] OR "2049-1999"[IS] OR "0725-6868"[IS] OR "1469-9540"[IS] OR "1364-2529"[IS] OR "1364-2529"[IS] OR "2040-8072"[IS] OR "0038-7134"[IS] OR "1388-364X"[IS] OR "1571-8166"[IS] OR "0735-0198"[IS] OR "1532-7981"[IS] OR "2618-7213"[IS] OR "2405-4739"[IS] OR "1879-4750"[IS] OR "0924-0608"[IS] OR "2082-7571"[IS] OR "2084-3763"[IS] OR "0021-9347"[IS] OR "0021-9347"[IS] OR "0002-7766"[IS] OR "0002-7766"[IS] OR "1535-8283"[IS] OR "0066-8435"[IS] OR "2514-9407"[IS] OR "2514-9407"[IS] OR "1819-5644"[IS] OR "1819-5644"[IS] OR "2451-0327"[IS] OR "1641-9278"[IS] OR "1540-6245"[IS] OR "0021-8529"[IS] OR "1930-5044"[IS] OR "1932-4421"[IS] OR "0065-101X"[IS] OR "1600-0390"[IS] OR "0892-2764"[IS] OR "1573-3335"[IS] OR "1826-9745"[IS] OR "2283-2998"[IS] OR "1573-1731"[IS] OR "1567-6617"[IS] OR "2155-2851"[IS] OR "2155-286X"[IS] OR "0066-1619"[IS] OR "1783-1334"[IS] OR "0969-725X"[IS] OR "1469-2899"[IS] OR "1051-144X"[IS] OR "2379-6529"[IS] OR "0003-0678"[IS] OR "1080-6490"[IS] OR "1988-2408"[IS] OR "1131-5598"[IS] OR "1102-7355"[IS] OR "1102-7355"[IS] OR "0047-2786"[IS] OR "1467-9833"[IS] OR "1467-9981"[IS] OR "0815-0796"[IS] OR "1568-5330"[IS] OR "0042-4935"[IS] OR "1471-6380"[IS] OR "0020-7438"[IS] OR "0191-4537"[IS] OR "0191-4537"[IS] OR "0195-6167"[IS] OR "1533-8339"[IS] OR "0022-5061"[IS] OR "1520-6696"[IS] OR "1743-2197"[IS] OR "1743-2197"[IS] OR "1139-7489"[IS] OR "1139-7489"[IS] OR "1137-8654"[IS] OR "2174-5382"[IS] OR "2340-5236"[IS] OR "2340-5236"[IS] OR "2395-9134"[IS] OR "2395-9134"[IS] OR "2332-0486"[IS] OR "0007-1773"[IS] OR "1572-0381"[IS] OR "1572-0373"[IS] OR "1475-3014"[IS] OR "1470-5427"[IS] OR "1529-0824"[IS] OR "2158-074X"[IS] OR "1502-7570"[IS] OR "1502-7589"[IS] OR "0922-6567"[IS] OR "1573-0573"[IS] OR "1369-8486"[IS] OR "1369-8486"[IS] OR "2571-9408"[IS] OR "2571-9408"[IS] OR "0022-5266"[IS] OR "1759-3999"[IS] OR "1741-5918"[IS] OR "1386-9795"[IS] OR "2079-9713"[IS] OR "2079-9705"[IS] OR "1532-8473"[IS] OR "1089-9472"[IS] OR "0718-1299"[IS] OR "0718-8358"[IS] OR "2328-1162"[IS] OR "0091-6471"[IS] OR "1576-4737"[IS] OR "1576-4737"[IS] OR "2514-9326"[IS] OR "2514-9326"[IS] OR "2226-471X"[IS] OR "2226-471X"[IS] OR "2164-0661"[IS] OR "1948-4992"[IS] OR "0039-7679"[IS] OR "1502-7805"[IS] OR "1744-6716"[IS] OR "1744-6716"[IS] OR "1578-7044"[IS] OR "1578-7044"[IS] OR "0028-6087"[IS] OR "1080-661X"[IS] OR "2461-0275"[IS] OR "2355-2794"[IS] OR "1741-6183"[IS] OR "0953-8208"[IS] OR "0353-5150"[IS] OR "1874-6349"[IS] OR "1352-3252"[IS] OR "1469-8048"[IS] OR "1874-5490"[IS] OR "1874-5504"[IS] OR "1750-1245"[IS] OR "1755-2036"[IS] OR "0129-797X"[IS] OR "0129-797X"[IS] OR "1532-7892"[IS] OR "1521-0960"[IS] OR "1813-5145"[IS] OR "1015-5104"[IS] OR "2279-7335"[IS] OR "2279-7327"[IS] OR "1479-1420"[IS] OR "1479-4233"[IS] OR "0929-8215"[IS] OR "0929-8215"[IS] OR "1932-4502"[IS] OR "1936-3567"[IS] OR "1132-6891"[IS] OR "1132-6891"[IS] OR "1464-9373"[IS] OR "1469-8447"[IS] OR "0869-3617"[IS] OR "2072-0459"[IS] OR "1469-218X"[IS] OR "0268-4160"[IS] OR "1747-5759"[IS] OR "1747-5767"[IS] OR "2041-6962"[IS] OR "0038-4283"[IS] OR "1860-7330"[IS] OR "1860-7349"[IS] OR "2310-0524"[IS] OR "2079-8555"[IS] OR "1469-3666"[IS] OR "1030-4312"[IS] OR "2321-7081"[IS] OR "0976-3996"[IS] OR "1470-4994"[IS] OR "2042-1273"[IS] OR "1097-3729"[IS] OR "0040-165X"[IS] OR "0265-6590"[IS] OR "1477-0865"[IS] OR "1934-5275"[IS] OR "1934-5275"[IS] OR "0103-4979"[IS] OR "0103-4979"[IS] OR "0038-1969"[IS] OR "0038-1969"[IS] OR "0955-2359"[IS] OR "1477-4674"[IS] OR "2333-7486"[IS] OR "2333-7494"[IS] OR "0892-6794"[IS] OR "0892-6794"[IS] OR "0887-5367"[IS] OR "1527-2001"[IS] OR "1547-3201"[IS] OR "0034-4087"[IS] OR "2049-6788"[IS] OR "2049-677X"[IS] OR "2340-3373"[IS] OR "1698-6989"[IS] OR "1059-8650"[IS] OR "2051-6169"[IS] OR "1121-7081"[IS] OR "1467-9914"[IS] OR "1879-3665"[IS] OR "1879-3665"[IS] OR "1476-4504"[IS] OR "2040-1388"[IS] OR "1838-0743"[IS] OR "2201-473X"[IS] OR "2162-7223"[IS] OR "0010-9894"[IS] OR "1547-3465"[IS] OR "1547-3465"[IS] OR "1478-2790"[IS] OR "1478-2804"[IS] OR "0042-8736"[IS] OR "0042-8736"[IS] OR "2325-5161"[IS] OR "0004-3125"[IS] OR "2153-7828"[IS] OR "0277-2027"[IS] OR "1527-2079"[IS] OR "0031-8213"[IS] OR "2353-7396"[IS] OR "2353-7396"[IS] OR "1988-2696"[IS] OR "1134-1629"[IS] OR "0018-2680"[IS] OR "0018-2680"[IS] OR "2509-9965"[IS] OR "2509-9965"[IS] OR "1552-8030"[IS] OR "1552-8049"[IS] OR "2341-0833"[IS] OR "1138-5774"[IS] OR

	"0333-5372"[IS] OR "0333-5372"[IS] OR "0039-3630"[IS] OR "2047-0584"[IS] OR "1557-4660"[IS] OR "1557-4660"[IS] OR "1741-1920"[IS] OR "1741-1912"[IS] OR "1130-2402"[IS] OR "1130-2402"[IS] OR "0031-0506"[IS] OR "0031-0506"[IS] OR "2077-1444"[IS] OR "2077-1444"[IS] OR "1900-5180"[IS] OR "0123-885X"[IS] OR "0068-2454"[IS] OR "0068-2454"[IS] OR "1996-4617"[IS] OR "2070-3449"[IS] OR "0391-9714"[IS] OR "1742-6316"[IS] OR "1811-7465"[IS] OR "2071-0437"[IS] OR "1570-0631"[IS] OR "0047-2212"[IS] OR "2451-8913"[IS] OR "2451-8921"[IS] OR "0147-1694"[IS] OR "0147-1694"[IS] OR "1744-9642"[IS] OR "1744-9650"[IS] OR "2159-9785"[IS] OR "2159-9793"[IS] OR "0940-7391"[IS] OR "0940-7391"[IS] OR "1357-6275"[IS] OR "1469-9885"[IS] OR "0951-631X"[IS] OR "1477-4666"[IS] OR "1470-112X"[IS] OR "1356-9783"[IS] OR "2615-3386"[IS] OR "2614-6584"[IS] OR "0725-5136"[IS] OR "0725-5136"[IS] OR "1871-9791"[IS] OR "1871-9805"[IS] OR "2044-0251"[IS] OR "2044-0243"[IS] OR "0007-5108"[IS] OR "0007-5108"[IS] OR "0176-4225"[IS] OR "1569-9714"[IS] OR "1749-6535"[IS] OR "1749-6543"[IS] OR "0925-4560"[IS] OR "1572-8587"[IS] OR "1478-2715"[IS] OR "1478-2715"[IS] OR "1552-6836"[IS] OR "0097-7004"[IS] OR "0031-8191"[IS] OR "1469-817X"[IS] OR "0955-2367"[IS] OR "1469-2961"[IS] OR "2072-8050"[IS] OR "0259-9422"[IS] OR "1573-6679"[IS] OR "0031-2789"[IS] OR "0184-7783"[IS] OR "0184-7783"[IS] OR "1852-4826"[IS] OR "1852-060X"[IS] OR "1338-5615"[IS] OR "2453-7829"[IS] OR "1469-8455"[IS] OR "1364-436X"[IS] OR "1467-9973"[IS] OR "0026-1068"[IS] OR "0556-8641"[IS] OR "1996-8523"[IS] OR "1988-4192"[IS] OR "0034-849X"[IS] OR "2442-3084"[IS] OR "1907-591X"[IS] OR "1849-0514"[IS] OR "1845-8475"[IS] OR "2213-3038"[IS] OR "2213-302X"[IS] OR "1943-4618"[IS] OR "1552-6100"[IS] OR "1467-8047"[IS] OR "1467-8047"[IS] OR "1572-9966"[IS] OR "1043-4062"[IS] OR "1944-2890"[IS] OR "1944-2904"[IS] OR "2051-820X"[IS] OR "2051-8196"[IS] OR "0021-8723"[IS] OR "1945-2314"[IS] OR "1728-192X"[IS] OR "1728-1938"[IS] OR "1361-9462"[IS] OR "1361-9462"[IS] OR "2345-0487"[IS] OR "2345-0479"[IS] OR "0014-1836"[IS] OR "0014-1836"[IS] OR "1675-8021"[IS] OR "1675-8021"[IS] OR "1680-0982"[IS] OR "1680-0982"[IS] OR "1581-2987"[IS] OR "0006-5722"[IS] OR "2631-6900"[IS] OR "2631-6897"[IS] OR "2168-9105"[IS] OR "2168-9113"[IS] OR "2067-8223"[IS] OR "2067-533X"[IS] OR "2288-9930"[IS] OR "2234-7224"[IS] OR "0813-0426"[IS] OR "0813-0426"[IS] OR "1873-930X"[IS] OR "0038-1527"[IS] OR "2074-7705"[IS] OR "1609-9982"[IS] OR "2224-8935"[IS] OR "1816-5435"[IS] OR "0824-2577"[IS] OR "0824-2577"[IS] OR "1944-8953"[IS] OR "1944-8961"[IS] OR "1972-5671"[IS] OR "1120-6462"[IS] OR "2658-6975"[IS] OR "2587-5566"[IS] OR "1477-4569"[IS] OR "1363-3554"[IS] OR "1885-446X"[IS] OR "1885-446X"[IS] OR "2151-7290"[IS] OR "2151-7290"[IS] OR "0718-1043"[IS] OR "0716-0925"[IS] OR "2157-930X"[IS] OR "2157-9318"[IS] OR "2164-4543"[IS] OR "2164-4551"[IS] OR "1975-3586"[IS] OR "1975-4019"[IS] OR "1448-2940"[IS] OR "1448-2940"[IS] OR "1756-9370"[IS] OR "1756-9389"[IS] OR "2468-8800"[IS] OR "2468-8797"[IS] OR "1529-1898"[IS] OR "0031-8221"[IS] OR "2451-3202"[IS] OR "2543-702X"[IS] OR "1881-8153"[IS] OR "1340-4202"[IS] OR "1470-2436"[IS] OR "1743-9698"[IS] OR "1745-7823"[IS] OR "1745-7831"[IS] OR "0960-7773"[IS] OR "1469-2171"[IS] OR "1469-5103"[IS] OR "0018-246X"[IS] OR "1943-2704"[IS] OR "1044-7946"[IS] OR "2533-2295"[IS] OR "2281-2881"[IS] OR "1871-4765"[IS] OR "1871-4757"[IS] OR "0860-2395"[IS] OR "2544-8870"[IS] OR "1469-3542"[IS] OR "1353-0194"[IS] OR "2472-9884"[IS] OR "2472-9876"[IS] OR "0819-8691"[IS] OR "0311-3248"[IS] OR "1750-4902"[IS] OR "2048-4887"[IS] OR "2541-9390"[IS] OR "1812-9323"[IS] OR "1754-6559"[IS] OR "1754-6567"[IS] OR "2156-7697"[IS] OR "2156-7689"[IS] OR "1732-4254"[IS] OR "2083-8298"[IS] OR "2674-4600"[IS] OR "2674-4619"[IS] OR "0103-3786"[IS] OR "0103-3786"[IS] OR "0100-1574"[IS] OR "0100-1574"[IS] OR "1532-6578"[IS] OR "1062-0303"[IS] OR "1818-7919"[IS] OR "1818-7919"[IS] OR "1465-4466"[IS] OR "1569-206X"[IS] OR "1347-2852"[IS] OR "1346-7581"[IS] OR "0003-0139"[IS] OR "1547-3848"[IS] OR "1745-3062"[IS] OR "1745-3054"[IS] OR "1503-111X"[IS] OR "0800-3831"[IS] OR "1527-2117"[IS] OR "0002-9831"[IS] OR "0002-0184"[IS] OR "1469-2872"[IS] OR "1939-6716"[IS] OR "1942-1273"[IS] OR "1572-8668"[IS] OR "0028-2677"[IS] OR "2373-2288"[IS] OR "0066-5983"[IS] OR "1811-833X"[IS] OR "2311-7133"[IS] OR "0957-1558"[IS] OR "1740-2352"[IS] OR "1745-5294"[IS] OR "0142-064X"[IS] OR "1462-3528"[IS] OR "1469-9494"[IS] OR "1756-4263"[IS] OR "1756-4255"[IS] OR "1857-3533"[IS] OR "1608-9057"[IS] OR "1461-720X"[IS] OR "0952-6951"[IS] OR "1469-5138"[IS] OR "0021-8537"[IS] OR "2204-9207"[IS] OR "2204-9193"[IS] OR "1479-8360"[IS] OR "1479-8360"[IS] OR "0128-5157"[IS] OR "2550-2247"[IS] OR "0021-8715"[IS] OR "1535-1882"[IS] OR "1522-4600"[IS] OR "1522-4600"[IS] OR "0029-5132"[IS] OR "0029-5132"[IS] OR "2720-1481"[IS] OR "0137-6985"[IS] OR "0015-587X"[IS] OR "1469-8315"[IS] OR "2052-3998"[IS] OR "2052-4005"[IS] OR "0486-6525"[IS] OR "0486-6525"[IS] OR "1598-7647"[IS] OR "2451-909X"[IS] OR "0035-0451"[IS] OR "0718-0934"[IS] OR "0869-6063"[IS] OR "0869-6063"[IS] OR "2354-0036"[IS] OR "2354-0036"[IS] OR "1479-0270"[IS] OR "0085-6401"[IS] OR "1728-9718"[IS] OR "1728-9718"[IS] OR "1988-4168"[IS] OR "0436-029X"[IS] OR "1593-7879"[IS] OR "1593-7879"[IS] OR "2081-2221"[IS] OR "1899-4849"[IS] OR "1944-3919"[IS] OR "1944-3927"[IS] OR "2380-1883"[IS] OR "2380-1891"[IS] OR "0963-9470"[IS] OR "1461-7293"[IS] OR "2009-0048"[IS] OR "0035-8991"[IS] OR "0045-6713"[IS] OR "0045-6713"[IS] OR "2455-328X"[IS] OR "2456-0502"[IS] OR "2371-4549"[IS] OR "2371-4549"[IS] OR "2056-6700"[IS] OR "2056-6700"[IS] OR "1920-7336"[IS] OR "0229-5113"[IS] OR

	"2406-0739"[IS] OR "0350-0241"[IS] OR "0211-1608"[IS] OR "2530-3589"[IS] OR "2014-8860"[IS] OR "2014-8860"[IS] OR "1745-817X"[IS] OR "0076-6097"[IS] OR "1581-128X"[IS] OR "1408-6271"[IS] OR "1810-6374"[IS] OR "2618-9844"[IS] OR "1834-6049"[IS] OR "1834-6057"[IS] OR "2050-3032"[IS] OR "2050-3040"[IS] OR "0191-6599"[IS] OR "1873-541X"[IS] OR "0123-3432"[IS] OR "0123-3432"[IS] OR "0976-3465"[IS] OR "0975-0878"[IS] OR "2046-6757"[IS] OR "2046-6749"[IS] OR "1744-9057"[IS] OR "1744-9057"[IS] OR "1542-6440"[IS] OR "1542-6432"[IS] OR "2254-9307"[IS] OR "1133-3634"[IS] OR "1855-8399"[IS] OR "0353-6483"[IS] OR "0042-8639"[IS] OR "0042-8639"[IS] OR "0130-2620"[IS] OR "0130-2620"[IS] OR "0263-7189"[IS] OR "2052-6148"[IS] OR "1466-1888"[IS] OR "1368-8790"[IS] OR "2500-1809"[IS] OR "2500-1809"[IS] OR "2451-280X"[IS] OR "1644-8561"[IS] OR "1901-9726"[IS] OR "1901-9726"[IS] OR "1571-0718"[IS] OR "1571-0726"[IS] OR "0011-3530"[IS] OR "0011-3530"[IS] OR "2174-1859"[IS] OR "2444-3239"[IS] OR "0967-0882"[IS] OR "0967-0882"[IS] OR "1745-9737"[IS] OR "1745-9745"[IS] OR "1361-7362"[IS] OR "1476-6787"[IS] OR "1542-3484"[IS] OR "0740-9710"[IS] OR "1583-0039"[IS] OR "1583-0039"[IS] OR "1071-7641"[IS] OR "1071-7641"[IS] OR "2500-1523"[IS] OR "2712-8059"[IS] OR "0719-4994"[IS] OR "0719-4994"[IS] OR "2587-9766"[IS] OR "2220-4156"[IS] OR "1353-2944"[IS] OR "1469-9877"[IS] OR "2156-7263"[IS] OR "2156-695X"[IS] OR "2153-8441"[IS] OR "1051-3558"[IS] OR "0197-1360"[IS] OR "1945-2330"[IS] OR "2376-1199"[IS] OR "2376-1202"[IS] OR "1477-2612"[IS] OR "0275-7206"[IS] OR "1743-4521"[IS] OR "1755-1714"[IS] OR "1569-9773"[IS] OR "1568-1475"[IS] OR "2255-8640"[IS] OR "2255-8632"[IS] OR "1556-3022"[IS] OR "1071-4413"[IS] OR "2213-4905"[IS] OR "2213-4913"[IS] OR "2096-3041"[IS] OR "2662-6802"[IS] OR "0008-3968"[IS] OR "0008-3968"[IS] OR "1353-7121"[IS] OR "1353-7121"[IS] OR "1751-942X"[IS] OR "1751-9411"[IS] OR "2578-4919"[IS] OR "2578-4900"[IS] OR "1337-8740"[IS] OR "1337-8740"[IS] OR "0719-4404"[IS] OR "0717-036X"[IS] OR "0334-4509"[IS] OR "0334-4509"[IS] OR "2326-8271"[IS] OR "2326-8263"[IS] OR "1567-7095"[IS] OR "1568-5373"[IS] OR "1477-4585"[IS] OR "0002-7189"[IS] OR "2311-911X"[IS] OR "2313-6871"[IS] OR "2312-4628"[IS] OR "2312-461X"[IS] OR "0350-6894"[IS] OR "0350-6894"[IS] OR "2410-4531"[IS] OR "2073-6606"[IS] OR "0963-9268"[IS] OR "1469-8706"[IS] OR "1472-5843"[IS] OR "1472-5851"[IS] OR "1086-3176"[IS] OR "0007-5140"[IS] OR "1886-4945"[IS] OR "1886-4945"[IS] OR "1743-9329"[IS] OR "0308-6534"[IS] OR "2079-8482"[IS] OR "2079-8482"[IS] OR "1534-6714"[IS] OR "0799-0537"[IS] OR "2541-9366"[IS] OR "2541-9358"[IS] OR "1740-9314"[IS] OR "1444-2213"[IS] OR "0015-1386"[IS] OR "1533-8630"[IS] OR "2548-8457"[IS] OR "2548-8465"[IS] OR "2040-0934"[IS] OR "1479-4713"[IS] OR "1474-001X"[IS] OR "0007-0874"[IS] OR "0380-1489"[IS] OR "0380-1489"[IS] OR "1823-7517"[IS] OR "2289-2672"[IS] OR "1355-7718"[IS] OR "1469-8153"[IS] OR "0393-3415"[IS] OR "2612-1026"[IS] OR "0719-2681"[IS] OR "0716-2278"[IS] OR "1467-7709"[IS] OR "0145-2096"[IS] OR "2048-8343"[IS] OR "0025-7273"[IS] OR "1330-0644"[IS] OR "1330-0644"[IS] OR "1357-1559"[IS] OR "1558-5476"[IS] OR "0037-1998"[IS] OR "1613-3692"[IS] OR "1098-304X"[IS] OR "1098-304X"[IS] OR "0018-2141"[IS] OR "1988-8368"[IS] OR "2450-7059"[IS] OR "1689-5150"[IS] OR "2326-4462"[IS] OR "2326-4470"[IS] OR "1473-5776"[IS] OR "1473-5784"[IS] OR "0021-325X"[IS] OR "2254-6219"[IS] OR "0346-8755"[IS] OR "1502-7716"[IS] OR "1472-586X"[IS] OR "1472-5878"[IS] OR "1464-5149"[IS] OR "0144-5340"[IS] OR "0718-5049"[IS] OR "0718-5049"[IS] OR "0022-1791"[IS] OR "0022-1791"[IS] OR "1941-7497"[IS] OR "0022-2909"[IS] OR "0160-7960"[IS] OR "2161-007X"[IS] OR "0016-7282"[IS] OR "2300-7362"[IS] OR "2210-4127"[IS] OR "2210-4119"[IS] OR "1753-5522"[IS] OR "1350-5033"[IS] OR "1576-0162"[IS] OR "1576-0162"[IS] OR "1943-8184"[IS] OR "1943-8192"[IS] OR "1681-5564"[IS] OR "1681-5564"[IS] OR "1580-8122"[IS] OR "1580-8122"[IS] OR "1532-7086"[IS] OR "1532-7086"[IS] OR "0210-1610"[IS] OR "0210-1610"[IS] OR "1751-7877"[IS] OR "0162-9778"[IS] OR "0955-2340"[IS] OR "1471-6917"[IS] OR "0131-1441"[IS] OR "0131-1441"[IS] OR "1777-5469"[IS] OR "0037-8984"[IS] OR "2619-1008"[IS] OR "2619-0990"[IS] OR "0718-9303"[IS] OR "0067-9674"[IS] OR "1757-1898"[IS] OR "1757-1901"[IS] OR "0268-4527"[IS] OR "0268-4527"[IS] OR "1570-9256"[IS] OR "0922-2936"[IS] OR "1556-4908"[IS] OR "1556-5009"[IS] OR "1944-2858"[IS] OR "1944-2858"[IS] OR "1467-8446"[IS] OR "0004-8992"[IS] OR "1469-9419"[IS] OR "1353-7903"[IS] OR "2572-6544"[IS] OR "2572-6544"[IS] OR "1741-2994"[IS] OR "1470-4129"[IS] OR "1406-992X"[IS] OR "1406-9938"[IS] OR "2232-5131"[IS] OR "2350-4226"[IS] OR "1474-0680"[IS] OR "0022-4634"[IS] OR "1045-6007"[IS] OR "1527-8050"[IS] OR "0718-2309"[IS] OR "0718-2309"[IS] OR "1939-0610"[IS] OR "1093-4510"[IS] OR "2391-4815"[IS] OR "0464-1086"[IS] OR "2521-442X"[IS] OR "2520-2073"[IS] OR "0042-8744"[IS] OR "0042-8744"[IS] OR "2340-4981"[IS] OR "1697-7750"[IS] OR "1469-9311"[IS] OR "0959-6410"[IS] OR "1548-1409"[IS] OR "1559-9167"[IS] OR "2055-7760"[IS] OR "2055-7760"[IS] OR "0921-5891"[IS] OR "1875-0257"[IS] OR "2453-9759"[IS] OR "1339-2204"[IS] OR "2339-9171"[IS] OR "0079-8215"[IS] OR "1573-0948"[IS] OR "0925-9392"[IS] OR "1527-2052"[IS] OR "0042-5222"[IS] OR "1841-0464"[IS] OR "1842-8517"[IS] OR "1350-7486"[IS] OR "1469-8293"[IS] OR "1469-9583"[IS] OR "1354-571X"[IS] OR "2644-4844"[IS] OR "1336-1635"[IS] OR "1877-7031"[IS] OR "1877-8798"[IS] OR "1045-0300"[IS] OR "1045-0300"[IS] OR "1063-2921"[IS] OR "1930-7799"[IS] OR "0075-4269"[IS] OR "0075-4269"[IS] OR "1752-6272"[IS] OR "1752-6280"[IS] OR "1460-8944"[IS] OR "1469-9907"[IS] OR "1998-6645"[IS] OR "2310-5046"[IS] OR

	"2340-8472"[IS] OR "2340-7743"[IS] OR "0929-077X"[IS] OR "1570-0577"[IS] OR "1575-0361"[IS] OR "1989-063X"[IS] OR "1536-3759"[IS] OR "1539-4107"[IS] OR "0021-1427"[IS] OR "2047-2153"[IS] OR "1744-9863"[IS] OR "1744-9855"[IS] OR "0046-208X"[IS] OR "0046-208X"[IS] OR "2470-2048"[IS] OR "2470-2048"[IS] OR "0011-1619"[IS] OR "1939-9138"[IS] OR "2174-8063"[IS] OR "2659-9295"[IS] OR "2405-6472"[IS] OR "2405-6480"[IS] OR "0921-0326"[IS] OR "0732-2992"[IS] OR "0068-2462"[IS] OR "0068-2462"[IS] OR "2444-0043"[IS] OR "2444-0043"[IS] OR "2203-7543"[IS] OR "2203-7543"[IS] OR "0039-4238"[IS] OR "0039-4238"[IS] OR "0591-2385"[IS] OR "1467-9744"[IS] OR "2254-2035"[IS] OR "2254-2035"[IS] OR "0332-4893"[IS] OR "2050-4918"[IS] OR "2049-6737"[IS] OR "2049-6729"[IS] OR "1139-7365"[IS] OR "1139-7365"[IS] OR "2573-573X"[IS] OR "1323-238X"[IS] OR "1334-2312"[IS] OR "1334-2312"[IS] OR "2414-3324"[IS] OR "2414-3324"[IS] OR "1877-8127"[IS] OR "1877-8127"[IS] OR "1745-5286"[IS] OR "0951-8207"[IS] OR "1935-0236"[IS] OR "0034-4338"[IS] OR "2713-2021"[IS] OR "2713-2021"[IS] OR "1846-8721"[IS] OR "0032-3241"[IS] OR "1572-1701"[IS] OR "1572-1701"[IS] OR "1750-2705"[IS] OR "2047-993X"[IS] OR "1448-4528"[IS] OR "1751-2964"[IS] OR "2007-3496"[IS] OR "1405-2253"[IS] OR "0119-8386"[IS] OR "0119-8386"[IS] OR "2373-7530"[IS] OR "2373-7530"[IS] OR "0361-5413"[IS] OR "1558-2744"[IS] OR "0068-113X"[IS] OR "0068-113X"[IS] OR "2415-7422"[IS] OR "2415-7430"[IS] OR "1783-1512"[IS] OR "1016-5584"[IS] OR "1748-5819"[IS] OR "0740-770X"[IS] OR "2051-7106"[IS] OR "2051-7114"[IS] OR "2560-3647"[IS] OR "0352-3160"[IS] OR "0022-7498"[IS] OR "1613-0723"[IS] OR "1930-8892"[IS] OR "1084-5453"[IS] OR "2697-6056"[IS] OR "1686-6541"[IS] OR "1744-8727"[IS] OR "1744-8735"[IS] OR "1949-0755"[IS] OR "0190-2407"[IS] OR "1468-4365"[IS] OR "0896-7148"[IS] OR "2058-1831"[IS] OR "2058-184X"[IS] OR "1468-2303"[IS] OR "0018-2656"[IS] OR "1749-6977"[IS] OR "1749-6985"[IS] OR "1479-0726"[IS] OR "1943-3107"[IS] OR "1541-8480"[IS] OR "1092-6690"[IS] OR "2633-4216"[IS] OR "2042-7867"[IS] OR "2500-4247"[IS] OR "2541-8564"[IS] OR "2550-1755"[IS] OR "1511-8819"[IS] OR "2336-4386"[IS] OR "0231-5823"[IS] OR "2040-4697"[IS] OR "2040-4689"[IS] OR "0893-7400"[IS] OR "1547-1896"[IS] OR "0022-4200"[IS] OR "0022-4200"[IS] OR "2309-7973"[IS] OR "2219-9659"[IS] OR "0008-4298"[IS] OR "2042-0587"[IS] OR "2039-2362"[IS] OR "2039-2362"[IS] OR "1139-1472"[IS] OR "1139-1472"[IS] OR "1529-8094"[IS] OR "1529-8094"[IS] OR "1369-801X"[IS] OR "1369-801X"[IS] OR "0023-1940"[IS] OR "0023-1940"[IS] OR "2219-3111"[IS] OR "2219-3111"[IS] OR "1462-317X"[IS] OR "1462-317X"[IS] OR "2660-891X"[IS] OR "1137-8905"[IS] OR "2014-5748"[IS] OR "2014-5748"[IS] OR "2619-9890"[IS] OR "1303-9407"[IS] OR "1988-8430"[IS] OR "1988-8430"[IS] OR "2542-2278"[IS] OR "2541-9382"[IS] OR "1478-8810"[IS] OR "1740-4649"[IS] OR "1044-2057"[IS] OR "1044-2057"[IS] OR "1983-9928"[IS] OR "1983-9928"[IS] OR "2056-998X"[IS] OR "2056-9971"[IS] OR "0717-6260"[IS] OR "0716-5455"[IS] OR "1743-9523"[IS] OR "0144-039X"[IS] OR "2385-8753"[IS] OR "1855-6302"[IS] OR "0307-1022"[IS] OR "1470-1200"[IS] OR "2083-2222"[IS] OR "2451-2168"[IS] OR "1757-9619"[IS] OR "1757-9627"[IS] OR "1368-8804"[IS] OR "1469-9729"[IS] OR "0029-5973"[IS] OR "1568-5276"[IS] OR "1474-6719"[IS] OR "1474-6700"[IS] OR "0071-1675"[IS] OR "0071-1675"[IS] OR "2212-9758"[IS] OR "2212-974X"[IS] OR "0073-2435"[IS] OR "0073-2435"[IS] OR "1133-598X"[IS] OR "2341-1112"[IS] OR "1988-4230"[IS] OR "0066-5061"[IS] OR "0014-2182"[IS] OR "0014-2182"[IS] OR "0172-6404"[IS] OR "0172-6404"[IS] OR "1794-7111"[IS] OR "1794-7111"[IS] OR "1543-5326"[IS] OR "1050-9208"[IS] OR "1130-8354"[IS] OR "2445-3587"[IS] OR "1745-5278"[IS] OR "1740-3553"[IS] OR "2008-0387"[IS] OR "2008-0387"[IS] OR "2307-1737"[IS] OR "2307-1753"[IS] OR "2366-0465"[IS] OR "1610-3181"[IS] OR "2254-6103"[IS] OR "1133-6137"[IS] OR "1995-1272"[IS] OR "1995-1272"[IS] OR "1138-6363"[IS] OR "1138-6363"[IS] OR "0213-2370"[IS] OR "0213-2370"[IS] OR "0192-2882"[IS] OR "1086-332X"[IS] OR "1537-5358"[IS] OR "0022-2801"[IS] OR "1752-7074"[IS] OR "1752-7066"[IS] OR "1741-4113"[IS] OR "1741-4113"[IS] OR "1046-2937"[IS] OR "1046-2937"[IS] OR "1478-5722"[IS] OR "1478-5730"[IS] OR "2339-7500"[IS] OR "2014-9298"[IS] OR "2685-5909"[IS] OR "2614-1566"[IS] OR "1989-6425"[IS] OR "1989-6425"[IS] OR "2003-0924"[IS] OR "2003-0924"[IS] OR "2405-836X"[IS] OR "2405-8351"[IS] OR "1467-9795"[IS] OR "0384-9694"[IS] OR "1995-4336"[IS] OR "1995-4328"[IS] OR "2067-7812"[IS] OR "1843-1348"[IS] OR "1998-9938"[IS] OR "2312-8704"[IS] OR "1710-1093"[IS] OR "0008-3755"[IS] OR "2313-0660"[IS] OR "2313-0679"[IS] OR "2073-7564"[IS] OR "2073-7564"[IS] OR "2386-8864"[IS] OR "1136-2464"[IS] OR "0101-4714"[IS] OR "1982-0267"[IS] OR "0928-0731"[IS] OR "0928-0731"[IS] OR "1067-8344"[IS] OR "0278-6656"[IS] OR "0103-2186"[IS] OR "2178-1494"[IS] OR "2172-0150"[IS] OR "2172-0150"[IS] OR "1411-5115"[IS] OR "2541-2426"[IS] OR "2150-4857"[IS] OR "2150-4865"[IS] OR "0130-3864"[IS] OR "0130-3864"[IS] OR "1085-7931"[IS] OR "0065-860X"[IS] OR "2328-1308"[IS] OR "2328-1308"[IS] OR "2165-9214"[IS] OR "2588-8099"[IS] OR "0036-9764"[IS] OR "0036-9764"[IS] OR "2462-6813"[IS] OR "1988-1088"[IS] OR "0869-1908"[IS] OR "0869-1908"[IS] OR "0043-5597"[IS] OR "0043-5597"[IS] OR "0739-8913"[IS] OR "2378-525X"[IS] OR "2320-2661"[IS] OR "2738-2222"[IS] OR "2161-9441"[IS] OR "2161-9468"[IS] OR "2313-8912"[IS] OR "2313-8912"[IS] OR "0360-5949"[IS] OR "1533-0699"[IS] OR "2325-7989"[IS] OR "2325-7962"[IS] OR "2708-2644"[IS] OR "0567-6002"[IS] OR "2073-9745"[IS] OR "2073-9745"[IS] OR "1754-8861"[IS] OR "1754-8853"[IS] OR "1740-0309"[IS] OR "1740-7923"[IS] OR "0007-7704"[IS] OR "1868-9027"[IS] OR

	"1569-7320"[IS] OR "1872-5171"[IS] OR "1934-9688"[IS] OR "1934-9696"[IS] OR "1749-8171"[IS] OR "1749-8171"[IS] OR "1130-3611"[IS] OR "1130-3611"[IS] OR "2593-0265"[IS] OR "2593-0265"[IS] OR "1759-8931"[IS] OR "0265-3788"[IS] OR "2008-0867"[IS] OR "2345-3117"[IS] OR "1471-6445"[IS] OR "0147-5479"[IS] OR "0258-2279"[IS] OR "0258-2279"[IS] OR "0026-7694"[IS] OR "1712-5286"[IS] OR "0026-7929"[IS] OR "0026-7929"[IS] OR "1469-8145"[IS] OR "0028-6885"[IS] OR "2150-9298"[IS] OR "2150-9301"[IS] OR "0956-7933"[IS] OR "1474-0656"[IS] OR "0321-0391"[IS] OR "0321-0391"[IS] OR "2237-2717"[IS] OR "2237-2717"[IS] OR "1754-1018"[IS] OR "1479-4012"[IS] OR "2333-9934"[IS] OR "0095-280X"[IS] OR "0974-4061"[IS] OR "0974-4053"[IS] OR "0255-6812"[IS] OR "0255-6812"[IS] OR "1037-1397"[IS] OR "1037-1397"[IS] OR "1994-6953"[IS] OR "2304-8069"[IS] OR "1728-2756"[IS] OR "1811-2773"[IS] OR "0030-8129"[IS] OR "0030-8129"[IS] OR "2333-262X"[IS] OR "2154-0993"[IS] OR "2331-2521"[IS] OR "2331-253X"[IS] OR "0716-7520"[IS] OR "0719-3262"[IS] OR "1422-6960"[IS] OR "1422-6944"[IS] OR "2475-1448"[IS] OR "2475-143X"[IS] OR "2618-8600"[IS] OR "2687-0789"[IS] OR "0021-6933"[IS] OR "1581-3754"[IS] OR "0022-0094"[IS] OR "1461-7250"[IS] OR "1751-3480"[IS] OR "1751-3472"[IS] OR "1573-3831"[IS] OR "0168-9789"[IS] OR "0013-8282"[IS] OR "0013-8282"[IS] OR "1480-8986"[IS] OR "1480-8986"[IS] OR "2450-050X"[IS] OR "2391-7997"[IS] OR "0213-2060"[IS] OR "0213-2060"[IS] OR "2532-3997"[IS] OR "2532-3997"[IS] OR "0008-3496"[IS] OR "0008-3496"[IS] OR "1475-5610"[IS] OR "1475-5610"[IS] OR "0973-0893"[IS] OR "0019-4646"[IS] OR "2079-8784"[IS] OR "2079-8784"[IS] OR "1552-6771"[IS] OR "0096-1442"[IS] OR "1060-1503"[IS] OR "1470-1553"[IS] OR "1874-656X"[IS] OR "1807-9326"[IS] OR "0021-1214"[IS] OR "0021-1214"[IS] OR "1527-6457"[IS] OR "1527-6457"[IS] OR "0255-0539"[IS] OR "0255-0539"[IS] OR "0037-783X"[IS] OR "0037-783X"[IS] OR "1869-2737"[IS] OR "1869-2729"[IS] OR "2068-6633"[IS] OR "2068-651X"[IS] OR "1581-8918"[IS] OR "2386-0316"[IS] OR "2306-0646"[IS] OR "2306-9910"[IS] OR "1467-6443"[IS] OR "0952-1909"[IS] OR "1534-8423"[IS] OR "1534-8415"[IS] OR "0973-1741"[IS] OR "0973-1733"[IS] OR "2300-6579"[IS] OR "2300-6579"[IS] OR "1550-7394"[IS] OR "1949-8381"[IS] OR "1337-9275"[IS] OR "1337-9690"[IS] OR "2405-5093"[IS] OR "1741-0819"[IS] OR "0590-9597"[IS] OR "1848-9079"[IS] OR "1478-0038"[IS] OR "1478-0046"[IS] OR "1689-8311"[IS] OR "1689-8311"[IS] OR "1407-3110"[IS] OR "1407-3110"[IS] OR "1469-9869"[IS] OR "0963-9489"[IS] OR "2398-9777"[IS] OR "2398-9785"[IS] OR "2159-340X"[IS] OR "0020-9643"[IS] OR "0307-0131"[IS] OR "0307-0131"[IS] OR "0009-8388"[IS] OR "1471-6844"[IS] OR "0046-385X"[IS] OR "0046-385X"[IS] OR "0143-3768"[IS] OR "2160-2506"[IS] OR "1080-6547"[IS] OR "0013-8304"[IS] OR "2072-8662"[IS] OR "2712-7575"[IS] OR "0865-4824"[IS] OR "2226-1877"[IS] OR "1604-9411"[IS] OR "0106-5815"[IS] OR "2040-4425"[IS] OR "2040-4417"[IS] OR "1076-0962"[IS] OR "1759-1090"[IS] OR "2643-8941"[IS] OR "2643-895X"[IS] OR "2255-5889"[IS] OR "2255-5889"[IS] OR "1612-9768"[IS] OR "1430-6921"[IS] OR "2040-0616"[IS] OR "1468-2761"[IS] OR "2006-4241"[IS] OR "2714-4321"[IS] OR "2058-6310"[IS] OR "2058-6310"[IS] OR "0314-769X"[IS] OR "1444-3058"[IS] OR "2590-3268"[IS] OR "2542-6583"[IS] OR "2385-8907"[IS] OR "2335-4127"[IS] OR "1360-2365"[IS] OR "1466-4410"[IS] OR "2356-4644"[IS] OR "2086-2636"[IS] OR "2211-792X"[IS] OR "2211-7954"[IS] OR "1744-4195"[IS] OR "1380-3603"[IS] OR "1783-1423"[IS] OR "0013-9513"[IS] OR "1336-7811"[IS] OR "1336-7811"[IS] OR "0003-5688"[IS] OR "0003-5688"[IS] OR "2154-6711"[IS] OR "1949-8519"[IS] OR "1749-4915"[IS] OR "1749-4907"[IS] OR "1016-2283"[IS] OR "2465-8545"[IS] OR "1520-9857"[IS] OR "2328-966X"[IS] OR "1327-9556"[IS] OR "1327-9556"[IS] OR "0006-0887"[IS] OR "0006-0887"[IS] OR "0017-1298"[IS] OR "0017-1298"[IS] OR "2508-5921"[IS] OR "1598-2041"[IS] OR "2040-5979"[IS] OR "2040-5960"[IS] OR "2449-8378"[IS] OR "2084-140X"[IS] OR "1874-8775"[IS] OR "1874-8767"[IS] OR "1524-4113"[IS] OR "1554-611X"[IS] OR "0035-0907"[IS] OR "0035-0907"[IS] OR "1022-4556"[IS] OR "1574-9282"[IS] OR "0021-0870"[IS] OR "1783-1482"[IS] OR "2687-8011"[IS] OR "2687-8003"[IS] OR "2163-3088"[IS] OR "0748-0814"[IS] OR "1132-8304"[IS] OR "2173-0687"[IS] OR "2408-1213"[IS] OR "2228-8279"[IS] OR "0026-7074"[IS] OR "1568-525X"[IS] OR "0049-8661"[IS] OR "0049-8661"[IS] OR "0891-3811"[IS] OR "0891-3811"[IS] OR "1579-9794"[IS] OR "1579-9794"[IS] OR "2708-5953"[IS] OR "2708-5945"[IS] OR "1528-4204"[IS] OR "0009-2002"[IS] OR "0024-094X"[IS] OR "0024-094X"[IS] OR "2044-3706"[IS] OR "2044-1983"[IS] OR "2532-6848"[IS] OR "2532-6848"[IS] OR "1699-4949"[IS] OR "1699-4949"[IS] OR "2054-1996"[IS] OR "2054-1988"[IS] OR "0254-9239"[IS] OR "2223-3768"[IS] OR "2000-0898"[IS] OR "2000-0898"[IS] OR "1741-1548"[IS] OR "2040-0594"[IS] OR "2042-5937"[IS] OR "2042-5937"[IS] OR "0026-8232"[IS] OR "1545-6951"[IS] OR "2352-1813"[IS] OR "2352-1805"[IS] OR "0004-0428"[IS] OR "1988-8511"[IS] OR "2171-861X"[IS] OR "0214-4808"[IS] OR "2225-5346"[IS] OR "2686-8989"[IS] OR "2054-4170"[IS] OR "2056-7790"[IS] OR "2055-2106"[IS] OR "2055-2114"[IS] OR "1695-5951"[IS] OR "1695-5951"[IS] OR "1475-3839"[IS] OR "1478-3398"[IS] OR "0257-2117"[IS] OR "0257-2117"[IS] OR "2040-350X"[IS] OR "2040-3518"[IS] OR "0003-8504"[IS] OR "0003-8504"[IS] OR "1755-6198"[IS] OR "1755-6201"[IS] OR "2724-1564"[IS] OR "2724-1564"[IS] OR "1527-8271"[IS] OR "1067-9847"[IS] OR "0018-2311"[IS] OR "0018-2311"[IS] OR "2014-1130"[IS] OR "2014-1130"[IS] OR "1471-6968"[IS] OR "0034-6551"[IS] OR "0065-1044"[IS] OR "0065-1044"[IS] OR "2000-4214"[IS] OR "2000-4214"[IS] OR "1656-152X"[IS] OR "1656-152X"[IS] OR "0716-5811"[IS] OR "0716-5811"[IS] OR

	"1575-1430"[IS] OR "1575-1430"[IS] OR "1812-5441"[IS] OR "1753-5409"[IS] OR "1018-5011"[IS] OR "1018-5011"[IS] OR "1502-7694"[IS] OR "1502-7694"[IS] OR "0036-9543"[IS] OR "1460-2474"[IS] OR "2326-4497"[IS] OR "2326-4489"[IS] OR "0066-4774"[IS] OR "0066-4774"[IS] OR "2288-2987"[IS] OR "1226-8046"[IS] OR "1828-5961"[IS] OR "1828-5961"[IS] OR "0071-1713"[IS] OR "0717-6171"[IS] OR "1741-6442"[IS] OR "0021-9894"[IS] OR "2375-6527"[IS] OR "2327-5731"[IS] OR "1003-7519"[IS] OR "1003-7519"[IS] OR "1553-0981"[IS] OR "1756-2597"[IS] OR "0277-1322"[IS] OR "0277-1322"[IS] OR "1450-569X"[IS] OR "2217-8066"[IS] OR "0001-9593"[IS] OR "1827-787X"[IS] OR "0570-6084"[IS] OR "0570-6084"[IS] OR "2346-691X"[IS] OR "2346-6901"[IS] OR "0003-5815"[IS] OR "0003-5815"[IS] OR "2057-035X"[IS] OR "2057-0341"[IS] OR "2042-7891"[IS] OR "2042-7905"[IS] OR "1353-4645"[IS] OR "1460-700X"[IS] OR "0046-1628"[IS] OR "0046-1628"[IS] OR "2171-0147"[IS] OR "2254-9633"[IS] OR "1750-807X"[IS] OR "1750-8061"[IS] OR "2329-0056"[IS] OR "2329-0048"[IS] OR "1825-263X"[IS] OR "1825-263X"[IS] OR "1989-8568"[IS] OR "1989-8568"[IS] OR "1754-0208"[IS] OR "1754-0194"[IS] OR "0716-6346"[IS] OR "0719-5176"[IS] OR "0858-1975"[IS] OR "2586-8721"[IS] OR "2183-3869"[IS] OR "2183-9956"[IS] OR "1882-6865"[IS] OR "1882-6865"[IS] OR "1079-3453"[IS] OR "1755-165X"[IS] OR "0974-9276"[IS] OR "0976-352X"[IS] OR "0315-0836"[IS] OR "0315-0836"[IS] OR "0009-837X"[IS] OR "1546-072X"[IS] OR "2272-9852"[IS] OR "0247-381X"[IS] OR "0012-8163"[IS] OR "1534-147X"[IS] OR "0869-5377"[IS] OR "2499-9628"[IS] OR "0210-6124"[IS] OR "0210-6124"[IS] OR "0014-1291"[IS] OR "0014-1291"[IS] OR "1740-4657"[IS] OR "1050-9585"[IS] OR "0891-9356"[IS] OR "1067-8352"[IS] OR "2532-5353"[IS] OR "2532-5353"[IS] OR "0029-4500"[IS] OR "0029-4500"[IS] OR "1813-7083"[IS] OR "1813-7083"[IS] OR "0818-8149"[IS] OR "0818-8149"[IS] OR "2345-1149"[IS] OR "1857-2685"[IS] OR "0013-8398"[IS] OR "0013-8398"[IS] OR "0019-0993"[IS] OR "0019-0993"[IS] OR "0950-236X"[IS] OR "1470-1308"[IS] OR "1579-7422"[IS] OR "1579-7422"[IS] OR "1988-2955"[IS] OR "0211-3589"[IS] OR "1478-7318"[IS] OR "1478-7318"[IS] OR "0044-216X"[IS] OR "0044-216X"[IS] OR "0066-7668"[IS] OR "0066-7668"[IS] OR "0008-5006"[IS] OR "0008-5006"[IS] OR "1989-7383"[IS] OR "1989-7383"[IS] OR "1543-4273"[IS] OR "1559-0895"[IS] OR "1382-5577"[IS] OR "1382-5577"[IS] OR "1989-614X"[IS] OR "0210-4911"[IS] OR "2336-4416"[IS] OR "1803-7399"[IS] OR "0585-5292"[IS] OR "1958-5705"[IS] OR "0001-6446"[IS] OR "1588-2667"[IS] OR "0885-0429"[IS] OR "1553-1201"[IS] OR "1759-7145"[IS] OR "1759-7137"[IS] OR "2405-5050"[IS] OR "2405-5069"[IS] OR "1989-3302"[IS] OR "1989-3302"[IS] OR "0127-046X"[IS] OR "0127-046X"[IS] OR "0043-2520"[IS] OR "0043-2520"[IS] OR "0858-0855"[IS] OR "0858-0855"[IS] OR "0894-8410"[IS] OR "0894-8410"[IS] OR "0190-0013"[IS] OR "1086-329X"[IS] OR "2204-0064"[IS] OR "1320-0968"[IS] OR "2160-5076"[IS] OR "0197-6745"[IS] OR "0036-5637"[IS] OR "0036-5637"[IS] OR "0364-0094"[IS] OR "1475-4541"[IS] OR "0093-3139"[IS] OR "1542-4286"[IS] OR "2151-9668"[IS] OR "1326-0219"[IS] OR "1394-9330"[IS] OR "1985-8353"[IS] OR "2175-7968"[IS] OR "1414-526X"[IS] OR "1744-4217"[IS] OR "0013-838X"[IS] OR "2415-3613"[IS] OR "1238-5018"[IS] OR "0582-9399"[IS] OR "0582-9399"[IS] OR "2640-7310"[IS] OR "0043-8006"[IS])
